# Lipophilic Constituents in *Salvia miltiorrhiza* Inhibit Activation of the Hepatic Stellate Cells by Suppressing the JAK1/STAT3 Signaling Pathway: A Network Pharmacology Study and Experimental Validation

**DOI:** 10.3389/fphar.2022.770344

**Published:** 2022-04-20

**Authors:** Ya-Xin Tang, Mingming Liu, Long Liu, Bo-Rui Zhen, Tian-Tian Wang, Na Li, Nanning Lv, Zhenyu Zhu, Guoquan Sun, Xiaobo Wang, Si Chen

**Affiliations:** ^1^ Key Laboratory of Computational Chemistry-Based Natural Antitumor Drug Research & Development, School of Traditional Chinese Materia Medica, Shenyang Pharmaceutical University, Shenyang, China; ^2^ School of Medicine, Shanghai University, Shanghai, China; ^3^ GongQing Institute of Science and Technology, Gong Qing, China; ^4^ Lianyungang Second People’s Hospital, Lianyungang, China; ^5^ School of Pharmacy, Second Military Medical University, Shanghai, China; ^6^ Xinhua Hospital, School of Medicine, Shanghai Jiao Tong University, Shanghai, China; ^7^ The 967th Hospital of the Chinese People’s Liberation Army Joint Logistics Support Force, Dalian, China

**Keywords:** *Salvia miltiorrhiza*, hepatic stellate cells, liver fibrosis, network pharmacology, JAK1/STAT3 signaling pathway

## Abstract

Liver fibrosis is currently a global health challenge with no approved therapy, with the activation of hepatic stellate cells being a principal factor. Lipophilic constituents in *Salvia miltiorrhiza* (LS) have been reported to improve liver function and reduce the indicators of liver fibrosis for patients with chronic hepatitis B induced hepatic fibrosis. However, the pharmacological mechanisms of LS on liver fibrosis have not been clarified. In this study, 71 active compounds, 342 potential target proteins and 22 signaling pathways of LS were identified through a network pharmacology strategy. Through text mining and data analysis, the JAK1/STAT3 signaling pathway was representatively selected for further experimental validation. We firstly confirmed the protective effect of LS on liver fibrosis *in vivo* by animal experiments. Hepatic stellate cells, which proliferated and displayed a fibroblast-like morphology similar to activated primary stellate cells, were applied to evaluate its underlying mechanisms. The results showed that LS could inhibit the cell viability, promote the cell apoptosis, decrease the expression of liver fibrosis markers, and downregulate the JAK1/STAT3 signaling pathway. These results demonstrated that LS could exert anti-liver-fibrosis effects by inhibiting the activation of HSCs and regulating the JAK1/STAT3 signaling pathway, which is expected to benefit its clinical application.

## Introduction

Liver fibrosis is a reversible wound healing reaction resulting from numerous chronic injuries (Aydin and Akcali, 2018). If the injuries persist, liver fibrosis will develop into cirrhosis, hepatocellular carcinoma, and death (Xu et al., 2019). At present, no clinically effective, specific, anti-liver-fibrosis biological or chemical therapy is available (Trivella et al., 2020). Therefore, there is an urgent need to identify effective anti-liver fibrosis agents. However, the liver heterogeneity causes the pathogenesis of hepatic fibrosis to be complex and diverse, which further complicates drug discovery in treating liver fibrosis (Aydin and Akcali, 2018).

Traditional Chinese medicine (TCM) herbs have unique advantages in the treatment of complex diseases, such as liver fibrosis, as they generally contain multiple ingredients that act by targeting multiple proteins and regulating numerous pathways (Xing et al., 2018; Chen et al., 2019; Yang et al., 2021). *Salvia miltiorrhiza* Bunge, commonly called Danshen, is the principal herb in prescriptions (e.g., Fuzheng Huayu Recipe and Compound 861) that have been widely used to treat liver fibrosis clinically (Xing et al., 2018). Further, clinical studies have shown that the lipophilic constituents in LS can improve the liver function and reduce the indicators of hepatitis-B-induced liver fibrosis (Liang, 2018). Many works have reported the mechanism of action of several active compounds in LS toward treating liver fibrosis (Ge et al., 2017; Wang R. et al., 2018; Shi et al., 2020). However, the pharmacological mechanisms of LS toward liver fibrosis have not been clarified.

**FIGURE 1:**
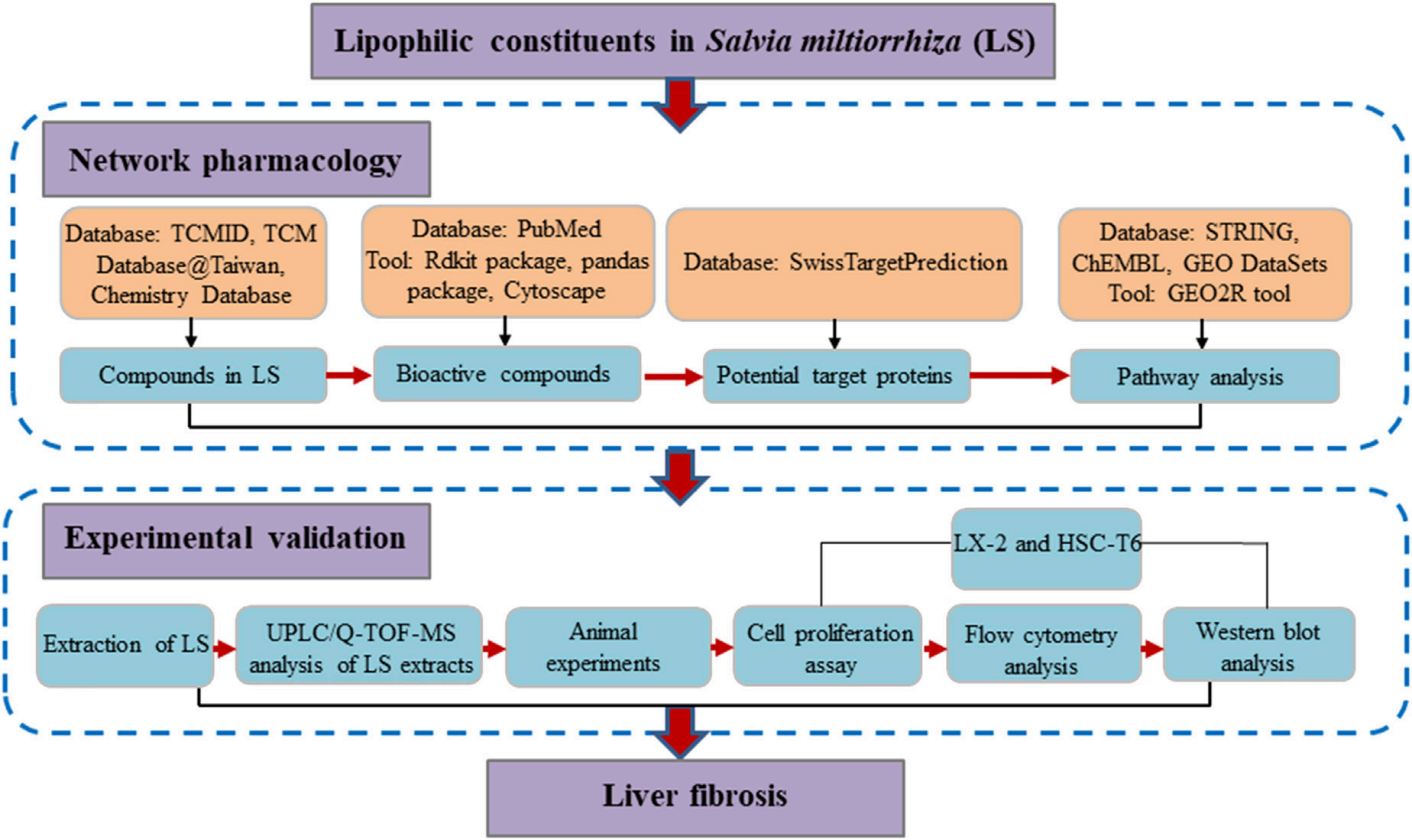
The flowchart of the whole study.

Network pharmacology, with the concept of “multi-compound-multi-target,” shares much with the TCM holistic concept (Chen et al., 2014). Thus, network pharmacology could be a suitable approach to investigate the molecular mechanisms of LS from a systemic perspective. Previous studies by our team have also confirmed the practicality of network pharmacology in investigating the mechanism of action of TCM (Chen et al., 2014; Chen et al., 2016; Xing et al., 2018). In addition, hepatic stellate cells (HSCs) are the main source of liver extracellular matrix (ECM), and the enzymes that regulate the degradation of ECM mainly exist in HSCs. Thus, the activation of HSCs is the central link of liver fibrosis (Aydin and Akcali, 2018). In this study, network pharmacology, a computational approach was applied to identify the underlying mechanisms by which LS exerts anti-liver-fibrosis effects. Subsequent pharmacological experiments were conducted to explore the inhibition activity of LS on HSCs and validate the network pharmacology results. A detailed flowchart is depicted in Figure 1. To our knowledge, this is the first study to investigate the potential active compounds and pharmacological mechanisms of LS in inhibiting the activation of HSCs for the treatment of liver fibrosis.

## Materials and Methods

### Materials

Salvia miltiorrhiza (LS; Place of Origin: Henan, China) was purchased from Shanghai Leiyunshang Pharmaceutical Co., Ltd. The HSC-T6 and LX-2 cell lines were purchased from Shanghai Fudan IBS Cell Resource Center. Phosphate buffer solution (PBS) was obtained from Chinese manufacturer Servicebio. Dulbecco’s Modified Eagle’s Medium (DMEM), Fetal bovine serum (FBS), Penicillin-Streptomycin, and trypsin (0.25%) were purchased from GIBCO (United States). NP-40 Lysis Buffer was procured from Beyotime (China). An EDTA-free protease inhibitor cocktail and PhosSTOP™ were purchased from Roche. Antibodies (Signal transducer and activator of transcription 3 [STAT3], phosphate signal transducer and activator of transcription [P-STAT3], tyrosine-protein kinase [JAK1], alpha-smooth muscle actin A [α-SMA], transforming growth factor β1 [TGFβ1], and GAPDH) were obtained from Abcam (United States). The polyclonal antibody P-JAK1 was purchased from Invitrogen. Recombinant human TGFβ1 was ordered from PeproTech. The IRDye® 800CW goat anti-Rabbit IgG secondary antibody was purchased from LI-COR Biotechnology (United States).An Annexin V-FITC apoptosis kit was purchased from Biolegend (United States). Cell Counting Kit-8 (CCK-8) was purchased from Beyotime Biotechnology Co., Ltd. (China).

### Construction of a Chemical Database of LS

TCMID (http://www.megabionet.org/tcmid), TCM Database@Taiwan (http://tcm.cmu.edu.tw), and Chemistry Database (http://www.chemcpd.csdb.cn/scdb) were searched to collect the names, structures, CAS numbers, and classifications of compounds in LS, thus constructing an in-house chemical database. Then, literature, books, the Encyclopedia of Chinese Medicine, and the PubChem database (https://pubchem.ncbi.nlm.nih.gov) were reviewed to confirm, merge, correct, and supplement the chemical information in our database.

### Construction of a Database With Known Anti-Hepatic Fibrosis Compounds in LS

We searched the PubMed database (https://pubmed.ncbi.nlm.nih.gov) with keywords, including the lipophilic compound names in LS in conjunction with liver fibrosis, hepatic fibrosis, or hepatic stellate cells. Compounds with anti-liver-fibrosis activity were screened and summarized.

### Construction of a Lipophilic Potential Active Compound Interaction Network Based on Molecular Similarity Calculation

Open Babel was applied to convert the SMILES format of compounds in the chemical database to SDF format (O’Boyle et al., 2011). Then the Rdkit package (https://www.rdkit.org/) in Python (https://www.python.org/) was used to calculate the Tanimoto similarity between compounds with anti-liver-fibrosis activity and compounds in LS based on the ECFP4 circular topological fingerprints. The pandas package was used to save the calculated value to an Excel table. Previous researchers conducted retrospective analysis on the data in ChEML and ZINC, suggesting that the tanimoto similarity thresholds recommended for ECFP4 fingerprints should be set to 0.4 (Muegge and Mukherjee, 2016), meaning compounds with similarity greater than 0.4 were more likely to have the same activity. In order to improve the accuracy, the similarity threshold in this study was set to 0.5. A potential active compound–compound interaction network was constructed with Cytoscape 3.7.2, where compounds with a Tanimoto similarity greater than 0.5 were linked with edges.

### Prediction and Enrichment Analysis of Potential Target Proteins

SwissTargetPrediction (http://www.swisstargetprediction.ch) was used to predict the potential target proteins of the compounds, which compares a query molecule with a library of 280,000 active compounds containing more than 2,000 targets. Then, a comprehensive cross-validation analysis was used to rank the predicted target proteins and analyze the accuracy of the target prediction. According to the distribution of target prediction related scores, we selected proteins with an average score greater than 0.1 as potential targets. In addition, because more than 99% of compounds in LS have more than two similar compounds (with a similarity threshold of 0.5), we set the number of target protein-related compounds to greater than two to improve the accuracy of the target prediction. Then, the STRING (https://string-db.org/) database was used to enrich and analyze the pathways and diseases related to the potential target proteins. Pathways or diseases with a false discovery rate of less than 0.05 and the number of related target proteins greater than two were considered to be statistically significant.

### Validation of the Interaction Between the Potential Active Compounds and the Potential Target Proteins

For direct verification, the SMILES representations of compounds were used as inputs to search the ChEMBL database (https://www.ebi.ac.uk/chembl/), and the known target proteins of these compounds were summarized. For indirect verification, the transcriptome data (GSE85871) describing the reference cell line MCF7 treated with tanshinone IIA and oleanolic acid from GEO DataSets (https://www.ncbi.nlm.nih.gov/gds/) were downloaded. It is worth noting that only the above two compounds of the LS have related transcriptome data. The GEO2R tool in GEO DataSets was used to screen the differential genes regulated by tanshinone IIA and oleanolic acid. Genes that differed significantly between the control and compound treated group (*p* < 0.05) were declared differentially expressed. An additional criterion requiring genes to have a two-fold average intensity difference of between the control and compound-treated group was also applied.

### Extraction of LS

Two hundred and fifty grams of LS root was crushed into a coarse powder. The lipophilic constituents were extracted via three rounds of ethanol-based heat reflux extraction at 70°C. After filtering and merging the filtrate, ethanol was reclaimed under a reduced pressure to concentrate it into extractum with a relative density of 1.35 (60°C), which was washed with hot water until it is colorless, freeze-dried and crushed it into a fine powder to afford the extraction of LS (1.5 g).

### Quality Control of LS Extracts

3 mg of powdered sample was weighed accurately in the eppendorf tube, and 500 μl of ethanol was added. The tube was sealed and placed in the ultrasound system. Once the powder dissolved, centrifuge at 5,000 rpm for 3 min. Two hundred microliter solution was taken and filtered with a 0.22 μm filter membrane, during which the continuous filtrate was collected. Chromatography was performed on Agilent 1290 Infinity UPLC system. A C18 column (2.1 mm × 150 mm, 1.8 μm, Waters, Milford, MA) was used for the separation. The column temperature was set at 25°C. The mobile phase consisted of 0.1% formic acid (A) and acetonitrile (B), using a gradient elution of 35%–60% B at 0–20 min, 60%–80% B at 20–25 min, 80%–95% B at 25–26 min, 95% B at 26–30 min. The flow rate was 0.4 ml•min^−1^ and the injection volume was 2 μl. The mass spectra were obtained by an Agilent 6530 Accurate-Mass Q-TOF mass spectrometer connected to the UPLC system via an ESI interface. The mass spectrometer was operated in positive ion mode and negative ion mode both with a capillary voltage of 3.5 kV, drying gas flow of 5 L/min, and a gas temperature of 325°C. The nebulizer pressure was set at 30 psig. The fragmentor voltage was set at 135 V and skimmer voltage was set at 65 V. Data were collected in centroid mode and the mass range was set at m/z 100–1,500 using extended dynamic range. The collision energy (CE) was optimized for the target derivatives from 10 to 30 eV. Then, identification of LS based on the acquired TIC chromatogram was conducted. The formulas were proposed based on the mass spectra and other rules, such as the general rule for the number of nitrogen atoms, the double bond equivalent (DBE) index and the ‘show isotopic’ function.

### Animal Experiments

C57BL/6 male mice (7 weeks old, 18–20 g) were obtained from Shanghai Slac Laboratory Animal Company (Shanghai, China). In the experiments, mice were randomly assigned to five groups (control, model, LS low-dose (LS-L), LS high-dose (LS-H) and LS-safety groups). In the liver fibrosis model, they were administered with carbon tetrachloride (CCl_4_) (5% olive oil dilution, 10 ml/kg) through intraperitoneal injection by twice weekly for nine consecutive weeks. The control (0.5% CMC-Na), model (0.5% CMC-Na), LS-L (18 mg/kg) and LS-H (180 mg/kg) group were administered via oral gavage every day for nine consecutive weeks. The LS-safety group was gavage with LS (180 mg/kg/day) for nine consecutive weeks to assess its safety. All animal experiments were conducted in the Animal Experiment Center of Second Military Medical University in accordance with the standard operating procedures.

### Serum Biochemical and Cytokine Analysis

After 24 h following the final injection, the peripheral blood was obtained from every mouse through eyeball enucleation. After 1 h of incubation at room temperature, the blood samples were centrifuged for 10 min at 3,000 rpm and 4°C for separation. The serum levels of laminin (LN; Langdun, cat no. BPE20195), hyaluronic acid (HA; Langdun, cat no. BPE20516), AST (Leidu, cat no. S03030), ALT (Leidu, cat no. S03040) were determined by serum ELISA kits, according to the manufacturer protocols. The optical density was read at specific wavelengths using the BioTek Synergy instrument.

### Examination of Hydroxyproline (Hyp) in Liver

Ice-cold lysis buffer was used to prepare tissue homogenates. The mixture was centrifuged for 15 min at 13,000 rpm and 4°C to collect the supernatant fractions, which were stored at −20°C for the quantification of protein levels. Then ELISA kits were used to determine the tissue content of Hyp (Langdun cat no. BPE20231).

### Histomorphology Assay

Hepatic tissues were processed with 4% paraformaldehyde fixation before embedding were embedded by paraffin, slicing into 5-µm sections. Hematoxylin-eosin (HE) staining was used to observe the inflammatory cell infiltration of livers. Liver fibrosis was estimated by Sirius Red staining. Liver fibrosis was graded using the Metavir fibrosis scoring.

### Cell Proliferation Assay

The HSC-T6 and LX-2 cells were seeded in 96-well plates with 8 × 10^3^ cells per well and cultivated for 12 h. Then cells were exposed to LS at various concentrations for 24 h. In addition to the administration groups, a control group (without treatment) and a blank group (without cells) were also set up. The cell viability was evaluated by CCK-8. The absorbance was detected using a Bio-Rad microplate reader (SynergyTM 4, BioTek, United States) at 450 nm. The percentage inhibition of cytotoxicity was calculated as follows: [(OD_administration group_-OD_blank group_)/(OD_control group_-OD_blank group_)] × 100%. All experiments were repeated three times.

### Flow Cytometry Analysis

The HSC-T6 and LX-2 cells were cultured in DMEM containing 10% FBS and 1% penicillin/ streptomycin at 37°C in a humidified atmosphere (5% CO_2_). Cells in the exponential growth phase were seeded in a 6-well plate (6 × 10^5^ cells per well) and grown overnight. After being treated with LS at different concentrations for 24 h, cells (both floating and adherent) were harvested and washed twice in PBS. Then the cells were resuspended in PBS and stained with Annexin V/FITC and propidium iodide for 15 min in the dark at room temperature. The cells were analyzed using a FACSCalibur instrument (Becton Dickinson, Mountain View, CA, United States).

### Western Blot Analysis

The HSC-T6 and LX-2 cells were seeded in a 6-well plate (6 × 10^5^ cells per well) and grown overnight. After being treated with gradient concentrations of LS for 2 h and subsequent 10 ng/ml recombinant human TGFβ1 for 22 h, the cells were collected and washed with PBS. The total protein was extracted in NP-40 buffer on ice and then centrifuged at 12,000 r/min at 4°C for 20 min to remove insoluble materials. The total protein was quantitated by bicinchoninic acid protein assay kit and retained for subsequent analysis. The total protein was electrophoresed by a 4%–20% SDS-PAGE gradient gel and electrotransferred to a polyvinylidene fluoride membrane. The membranes were blocked with BlockPRO^TM^ 1 Min Protein-Free Blocking Buffer at room temperature for 15 min and then immunoblotted overnight at 4°C with the following primary antibodies: STAT3 (1:2,000), P-STAT3 (1:1,000), α-SMA (1:10,000), JAK1 (1:500), P-JAK1 (1:1,000), TGFβ1 (1:1,000) and GADPH (1:10,000). After being washed in Tris-Buffered Saline and Tween 20 for 5 min three times, the membranes were subsequently incubated with IRDye® 800CW Goat anti-Rabbit IgG Secondary Antibody (1:3,000) for 1 h at room temperature. The membranes were washed in TBST three times for 5 min each again. Finally, protein gray-scale bands were scanned and analyzed by an Odyssey infrared imaging system (LI-COR, United States), GADPH was used as an internal control.

### Statistical Analysis

The experimental data in this study were analyzed statistically using GraphPad Prism software (version 8.0.1 GraphPad, Inc, San Diego, CA, United States). The results are expressed as the mean ± standard error of the mean. The significant differences between the groups were explored using one-way ANOVA followed by the LSD or Tukey’s test. A *p*-value less than 0.05 is considered statistically significant.

## Results

Network pharmacology analysis to identify the potential active compounds and action mechanisms of LS against liver fibrosis

### Known Anti-Hepatic Fibrosis Lipophilic Compounds in *Salvia miltiorrhiza*

We collected a total of 138 lipophilic compounds in *Salvia miltiorrhiza*, with no repetitive structures, including 102 diterpenoids, 16 triterpenoids, six steroids, and 14 other compounds (Supplementary Table S1). As there were few reports on the liver-protecting activity of volatile oil in *Salvia miltiorrhiza*, we did not summarize this kind of lipophilic compound. At present, eight lipophilic compounds in *Salvia miltiorrhiza* are reported to have anti-liver-fibrosis effects (*in vivo* or *in vitro*): three of them were the subject of target-related studies, while the other compounds only involved pathway-related studies. Among the eight lipophilic compounds in *Salvia miltiorrhiza*, only tanshinone IIA has been involved in more than 10 liver-fibrosis-related studies.

### Active Compounds Prediction Through the Construction of a Potential Active Compound Interaction Network

**FIGURE 2:**
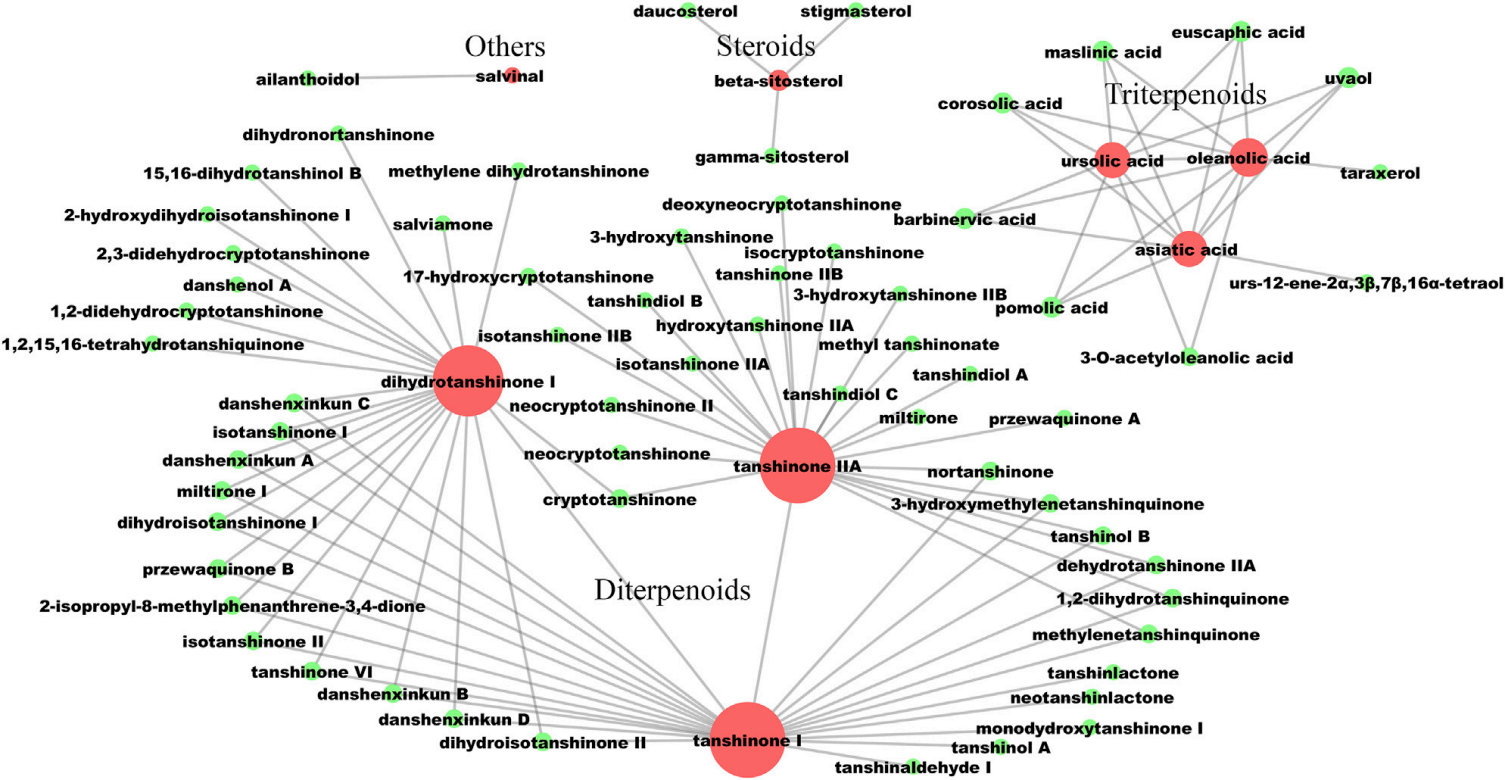
The lipophilic potential active compounds interaction network. The red circles represent compounds that have been clearly reported to have anti-liver fibrosis effects at the animal or cellular level. The green circles represent potential active compounds with a structural similarity greater than 0.5 to the corresponding red nodes. The larger the node, the more related similar compounds. The compound-compound interactions are linked by edges.

The screening process of active compounds in LS was hierarchically descripted in Supplementary Figure S1. A potential active compound–compound interaction network was constructed, which contained 71 nodes and 100 edges (Figure 2). In this network, the compounds were divided into four categories: including diterpenoids, triterpenoids, steroids and others. Based on eight known active ingredients in the center (red nodes, Figure 2), this network identified 63 potential active compounds (green nodes) from 130 lipophilic constituents of unknown activity. Among the 63 potential active compounds, 50 compounds were diterpenoids, nine compounds were triterpenoids, three compounds were steroids, and only one compound belongs to the others category.

### The Target Prediction and Validation of LS for the Treatment of Hepatic Fibrosis

A total of 342 potential target proteins were obtained by SwissTargetPrediction, of which 196 potential target proteins were enzymes, 51 potential target proteins were G-protein-coupled receptors, 19 potential target proteins were nuclear receptors, 18 potential target proteins were ion channels, and 58 were other kinds of proteins (Supplementary Table S2). Specifically, there were 53 diterpenoids targeting 298 potential proteins; 12 triterpenoids targeting 129 potential proteins; four steroids targeting 120 potential proteins; and two other compounds targeting 79 potential proteins.

We then we searched the ChEMBL and GEO datasets to determine the validated interaction between the potential active compounds and the potential target proteins (Supplementary Table S3). The results showed that 41 potential active compound-target interactions were reported in the ChEMBL database, and 94 differential genes corresponding to the predicted target protein were regulated by tanshinone IIA and oleanolic acid.

### Enriched Diseases and Pathways of the Potential Target Proteins

**FIGURE 3:**
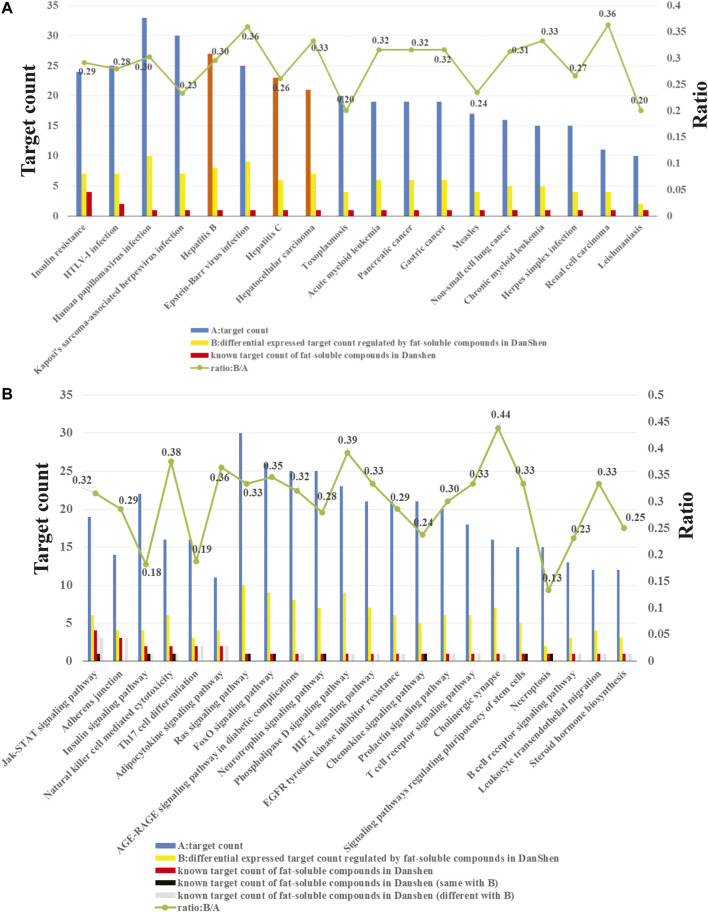
**(A)** Enriched diseases related to the potential target proteins. **(B)** Enriched pathways related to the potential target proteins.

The enrichment analysis results showed that the potential target proteins were mainly involved in 182 statistically significant pathways or diseases. At present, the enrichment-related data have the following three characteristics. First, there were numerous potential targets, related pathways and diseases regulated by the 71 potential active compounds (Figure 3). Second, we only retained the compound–target interaction pairs with Kd or IC_50_ values less than 10 μM collected from ChEMBL; thus, these compound–target interactions are credible. Third, since transcriptomics is a high-throughput tool, the reliability of differential genes regulated by the potential active lipophilic compounds in *Salvia miltiorrhiza* is limited. In order to identify biologically significant pathways or diseases and obtain more accurate results, pathways, or diseases with more than 10 potential target proteins, more than one known target protein and more than 10% known differential genes were selected (Figure 3).

As shown in Figure 3A, the potential target proteins are closely related to hepatitis B, hepatitis C, and hepatocellular carcinoma (orange bars). Hepatitis B and hepatitis C are the causes of liver fibrosis, and hepatocellular carcinoma is the result of liver fibrosis. These indicate that the potential target proteins are closely related to liver fibrosis, which proved the reliability of our target identification methods. Since there is no entry containing liver fibrosis in the KEGG database, it cannot be enriched by the enrichment method applied in this study.

In addition, the target pathway enrichment analysis results showed that the LS may exert its anti-liver-fibrosis effect by regulating 22 pathways (Figure 3B). Among them, the pathway containing the largest number of known targets is the JAK/STAT signaling pathway. Therefore, the LS may treat liver fibrosis primarily by regulating this pathway.

### JAK1/STAT3 Signaling Pathway was Representatively Selected for Further Experimental Validation

As seen in Figure 3B, the JAK/STAT signaling pathway contains a total of 19 potential target proteins (Supplementary Table S4), of which fours proteins are known targets for the LS and six proteins are known differential genes regulated by LS. The relationships between LS and the target proteins or differential genes in the JAK/STAT signaling pathway are shown in Table 1. In order to screen out the most potential target protein in the JAK/STAT signaling pathway for subsequent experimental verification, we searched the PubMed database and determined the known interactions between the target proteins and liver fibrosis. First, target proteins (PTPN2 and PIM1) that have not been clearly reported to be related to liver fibrosis were excluded. Then, target proteins (PTPN11, PTPN6, PIK3CB, PIK3CD, and EGFR) that had contradictory compound-target protein disease interactions were excluded. For example, the ChEMBL database results show that dihydrotanshinone I, tanshinone I and tanshinone IIA are inhibitors of PTPN11, and their IC_50_ is less than 10 μM. The literature also shows that PTPN11 inhibitors can alleviate CCl4-induced liver fibrosis (Kostallari et al., 2018), but transcriptome data show that tanshinone IIA can upregulate PTPN11. Due to this contradiction, the relationship between LS and PTPN11 requires further verification In addition, the ChEMBL database shows that dihydrotanshinone I, tanshinone I, and tanshinone IIA are inhibitors of PTPN6, and their IC_50_ is less than 10 μM. However, the literature reports that PTPN6 agonist could ameliorate liver fibrosis by upregulating PTPN6 (Su et al., 2017). Thus, PTPN6 was excluded due to this contradiction.

**TABLE 1:** The relationship between LS and the target proteins or differential genes.

Compound	Target	Gene	Interaction of compound and target/gene
Dihydrotanshinone I	PTPN11		IC_50_ = 3,940 nM
Tanshinone I	PTPN11		IC_50_ = 2,570 nM
Tanshinone IIA	PTPN11	PTPN11	IC_50_ = 2,590 nM; Fold change (drug/control) = 2.06
Ursolic acid	PTPN2		IC_50_ = 2,400 nM
Dihydrotanshinone I	PTPN6		IC_50_ = 3,670 nM
Tanshinone I	PTPN6		IC_50_ = 1970 nM
Tanshinone IIA	PTPN6		IC_50_ = 2,140 nM
Cryptotanshinone	STAT3		IC_50_ = 4,600 nM
Tanshinone IIA		PIK3CB	Fold change (drug/control) = 2.08
Oleanic acid		PIK3CD	Fold change (drug/control) = 2.58
Tanshinone IIA		PIM1	Fold change (drug/control) = 2.21
Tanshinone IIA		AKT2	Fold change (drug/control) = 0.20
Oleanic acid		AKT2	Fold change (drug/control) = 0.09
Tanshinone IIA		EGFR	Fold change (drug/control) = 2.0

Both STAT3 and AKT2 have been reported in the literature to be closely related to liver fibrosis, and inhibiting STAT3 or AKT2 can treat liver fibrosis (Su et al., 2015; Reyes-Gordillo et al., 2019). In addition, cryptotanshinone, tanshinone IIA and oleanic acid have been reported to be inhibitors of STAT3 and AKT2 (Table 1). Thus we speculate that STAT3 and AKT2 in the JAK/STAT signaling pathway are the main potential target proteins of LS. As STAT3 has higher target-prediction-related scores and number of target protein-related compounds than AKT2 (Supplementary Table S4), we selected STAT3 for subsequent experimental verification.

Apart from the four known target proteins and six known differential gene proteins regulated by LS, there are 10 other potential target proteins (PIK3CA, JAK3, JAK1, JAK2, MTOR, CCND1, MCL1, CREBBP, PDGFRA and TYK2) that have not been reported to be related to LS. Among these 10 proteins, PIK3CA, JAK3, JAK, and JAK2, which have more than 10 related compounds are regarded as high potential target proteins.

A previous study reported that STAT3 directly participated in the activation and transdifferentiation of HSC in response to TGFβ and subsequent hepatic fibrosis (Tang et al., 2017), it also showed that the JAK1/STAT3 signaling pathway played an important role in HSC activation and liver fibrosis (Tang et al., 2017). Therefore, we further validated whether the JAK1/STAT3 signaling pathway was involved in the LS-mediated anti-liver-fibrosis process.

### Experimental Validation

#### Quality Control of LS Extracts

**FIGURE 4:**
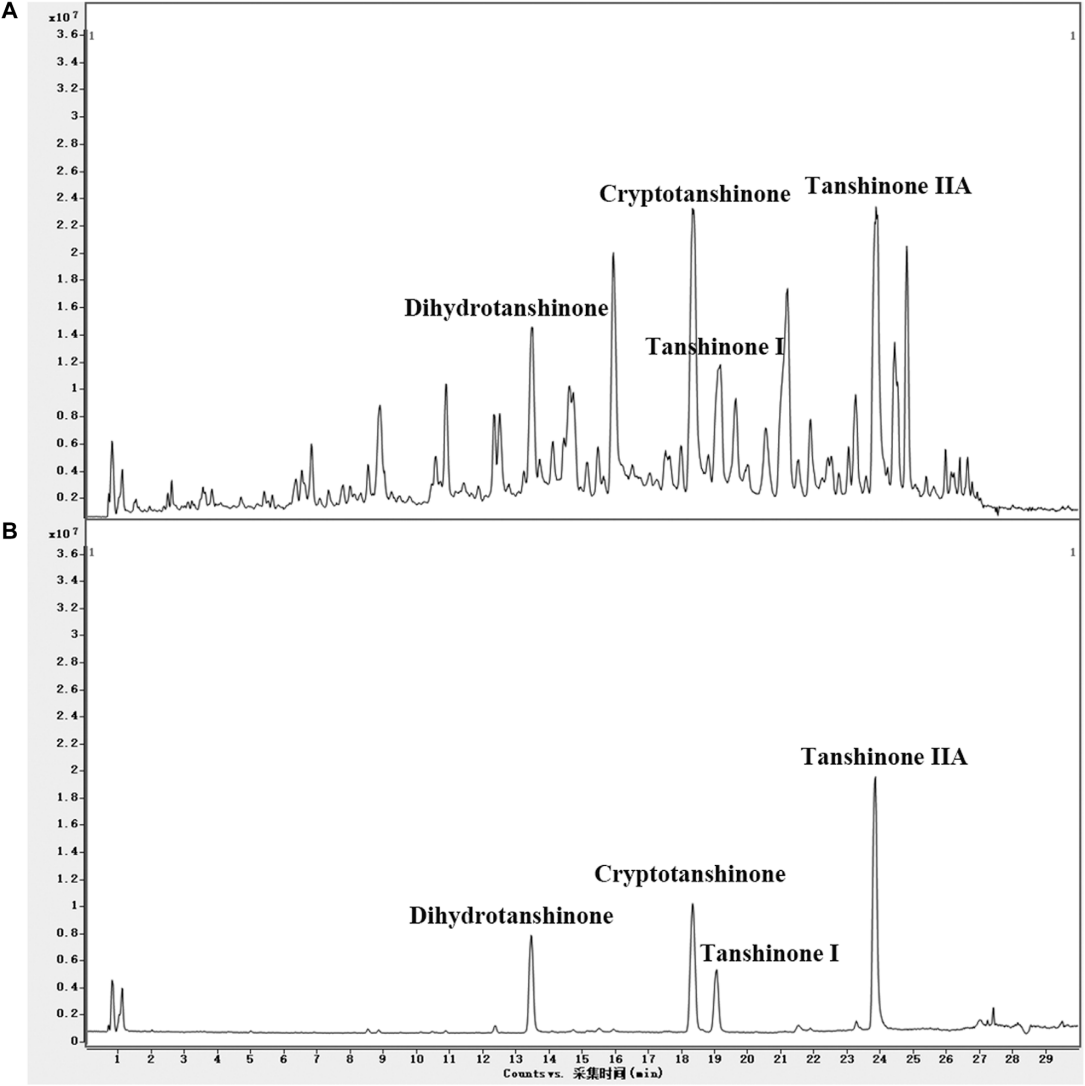
**(A)** UPLC/Q-TOF-MS TIC chromatogram in positive ion mode of the LS extracts and **(B)** four standards.

UPLC/Q-TOF-MS TIC chromatograms of the LS extracts and four standards were acquired. Compounds in LS were identified by the mass spectra and other procedures described in *Materials and Methods*. The molecular formulas were calculated by high-accuracy quasi-molecular ions using a widely accepted mass accuracy threshold of less than 5 ppm. Now, 102 diterpenoids, 16 triterpenoids, six steroids, and 14 other compounds were summarized in the chemical database of LS (Supplementary Table S1). As for non-target compound identification, 93 diterpenoids, 11 triterpenoids, one steroid and six other compounds were tentatively identified in LS (Supplementary Table S3). In addition, we identified the target compounds in LS by comparing the retention times and mass spectra with those of the standards. As shown in Figure 4, cryptotanshinone, dihydrotanshinone I, tanshinone I and tanshinone IIA as representative compounds in LS, were precisely identified. These results demonstrated that the LS extracts met the quality standard.

#### LS Ameliorated CCl_4_-Induced Liver Pathological Changes and Dysfunction

**FIGURE 5:**
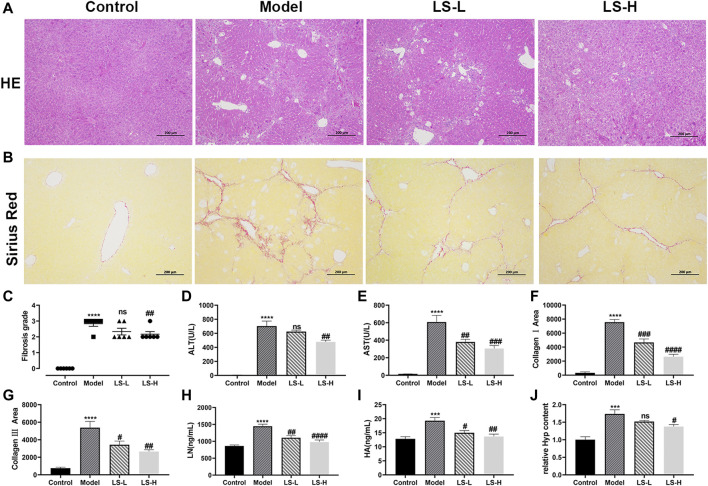
**(A)** Representative liver tissues after H&E-staining. **(B)** Representative liver tissues after Sirius Red-staining. **(C)** Metavir fibrosis scores. **(D,E,H,I)** The contents of alanine aminotransferase (ALT), aspartate aminotransferase (AST), laminin (LN) and hyaluronic acid (HA) in serum. **(F)** CollagenⅠarea determined by Sirius red staining. **(G)** Collagen Ⅲ area determined by Sirius red staining. **(J)** The relative contents of hydroxyproline (Hyp) in liver tissue. The data are expressed as the mean ± standard error of the mean (SEM) (*n* = 6), ***p < 0.001 vs. control group, ****p < 0.0001 vs. control group, ^#^ *p* < 0.05 vs. model group, ^##^ *p* < 0.01 vs. model group, ^###^ *p* < 0.001 vs. control group, ^####^ *p* < 0.0001 vs. model group.

To verify the effect of LS on the CCl_4_-induced liver fibrosis, H&E-stained liver tissue sections were subjected to microscopy analysis. As shown in Figure 5A, in the control group, the hepatic lobules were clearly demarcated and arranged regularly, without collagen fiber hyperplasia and inflammatory lesions. The liver tissue of the model group showed a blurred hepatic lobule structure, destruction of the hepatocyte cord, mild cell swelling, necrosis and fatty degeneration, and infiltration of inflammatory cells and fibroblasts, which were partially alleviated after LS-H treatment but not alleviated after LS-L treatment. The fibrosis grading scores, which came from the H&E-staining, showed the same trend. They were markedly elevated in CCl_4_-stimulated mice compared with the control group and conversely significantly reduced in the LS-H group, but not significantly reduced in the LS-L group (Figure 5C). Simultaneously, LS-H treatment led to a remarkable decrease in the ALT and AST contents, which demonstrated the protective effect of LS-H on liver function in liver fibrosis mice (Figures 5D,E). However, LS-L treatment could only lead to a remarkable decrease in the AST content, which demonstrated that LS-L treatment was less effective than LS-H treatment. In addition, the H&E-stained organ tissue sections, ALT and AST in the safety evaluation group were not significantly different from those in the control group, indicating that LS had no obvious toxicity to mice (Supplementary Figure S2).

#### LS Alleviates Liver Fibrosis Induced by CCl_4_

A reference reported that ECM included collagens (I, III and IV), LN, HA and so on, and fibrotic liver contained 3–10 times more ECM than the normal liver (Nallagangula et al., 2018). We investigated the regulatory role of LS on the ECM by several markers. Under the induction of CCl_4_, the area of collagen fibers, collagen I area, collagen III area, LN and HA in the model group was significantly higher than that in the normal group, and both LS-H and LS-L treatment significantly reduced them (Figures 5B, F–I). Hyp is an important constituent of collagen and play a key role in the synthesis and stability of the collagen. Hyp could be applied as an important biomarker for quantification of the collagen content. The Hyp in the liver tissue of the CCl_4_ group was significantly higher than that of the control group, and it was greatly down-regulated after LS-H treatment but not by LS-L treatment (Figure 5J). Above results demonstrated that LS-H was more effective than LS-L in treating liver fibrosis.

#### LS Inhibited the Viability and Increased the Apoptosis of HSCs

**FIGURE 6:**
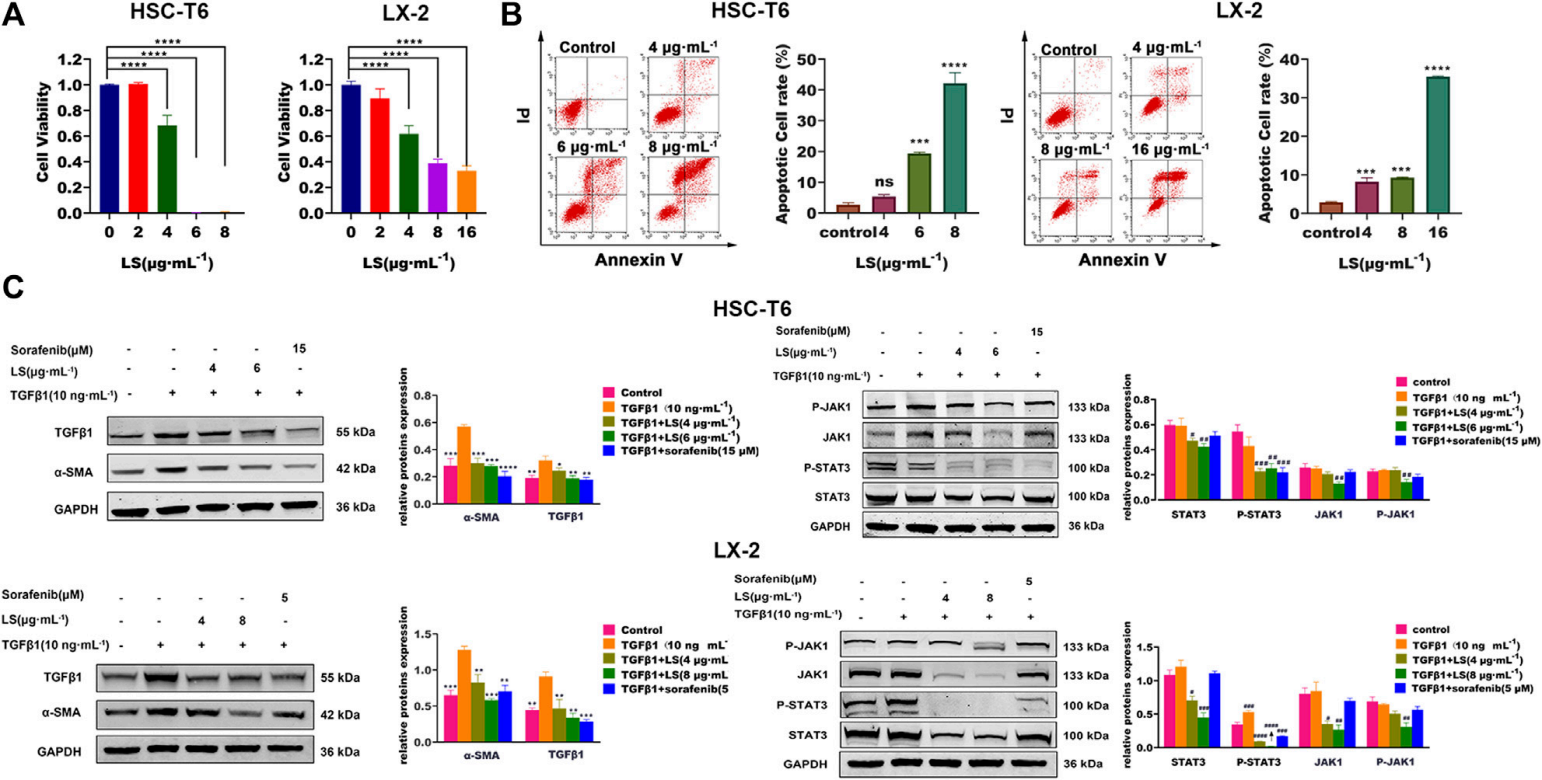
**(A)** Dose-escalation effects of LS for 24 h on cell viability in HSC-T6 and LX-2. The data are expressed as the mean ±SEM (*n* = 3), ****p < 0.0001 vs. control group. **(B)** Dose-escalation effects of LS for 24 h on apoptosis in HSC-T6 and LX-2. The data are expressed as the mean ± SEM (*n* = 3), ***p < 0.001 vs. control group, ****p < 0.0001 vs. control group. **(C)** Dose-escalation effects of LS for 24 h on alpha smooth muscle actin (α-SMA), transforming growth factor β1 (TGFβ1), signal transducer and activator of transcription 3(STAT3), phosphorylated signal transducer and activator of transcription 3(P-STAT3), Janus kinase 1(JAK1) and phosphorylated Janus kinase 1 (P-JAK1) in HSC-T6 and LX-2. The data are expressed as the mean ± SEM (*n* = 3), *p < 0.05 vs. TGFβ1 group, **p < 0.01 vs. TGFβ1 group, ***p < 0.001 vs. TGFβ1 group, ****p < 0.0001 vs. TGFβ1 group, ^#^ *p* < 0.05 vs. control group, ^##^ *p* < 0.01 vs. control, ^###^ *p* < 0.001 vs. control group.

HSC-T6, as an immortalized rat stellate cell line, proliferate and display a fibroblast-like morphology like activated primary stellate cells (Vogel et al., 2000). LX-2 cells, similar to primary HSCs, were generated by spontaneous immortalization in low serum conditions (Xu et al., 2005). HSC-T6 and LX-2-based biological experiments were firstly conducted to evaluate the *in vitro* anti-hepatic fibrosis activity of LS. As shown in Figure 6A, LS had dose-dependent effects against cell viability toward HSC-T6 and LX-2. In addition, apoptotic HSCs were also significantly increased after LS treatment in a dose-dependent manner by flow cytometry (Figure 6B). After exposure to LS at different concentrations of 0, 4, 6, and 8 µg/ml for 24 h. These results demonstrated that LS could suppress the activation and promote the apoptosis of HSC.

#### LS Inhibited JAK1/STAT3 Signaling Pathway in Hepatic Stellate Cells

HSC-T6 and LX-2 cells was further applied to investigate the effects of LS on HSC activation and the JAK1/STAT3 signaling pathway. TGFβ1 is an important pro-fibrogenic factor and a direct marker for the evaluation of liver fibrosis (Nallagangula et al., 2018), and α-SMA is a reliable marker for HSC activation (Nallagangula et al., 2018). Sorafenib, which has been reported to ameliorate liver fibrosis by reducing the expression of α-SMA, TGFβ1, and P-STAT3 (Su et al., 2015), was used as a positive control in this study.

As shown in Figure 6C, TGFβ1 treatment for 24 h significantly upregulated the expression of α-SMA and TGFβ1 compared to the control group, as detected by western blot tests, which indicated that HSCs were further activated by TGFβ1. After LS and sorafenib treatment, their expression was significantly reduced, which demonstrated that LS and sorafenib could inhibit the activation of HSCs and attenuate liver fibrosis. We then investigated the effects of LS on the JAK1/STAT3 signaling pathway, in which the expression of JAK1, P-JAK1, STAT3, and P- STAT3 was examined. As shown in Figure 6C, TGFβ1 treatment for 24 h did not affect the expression of JAK1, P-JAK1, STAT3 or P-STAT3. After LS and sorafenib treatment, the expression of P-STAT3 was significantly reduced. In addition, LS significantly downregulated the expression of STAT3, JAK1, and P-JAK1. However, the positive control, sorafenib didn’t affect their expression, which was in accordance with a known study (Su et al., 2015). To summarize, LS could inhibit the activation of HSCs by suppressing the JAK1/STAT3 signaling pathway.

## Discussion

In this study, we determined that LS could treat liver fibrosis through 71 active compounds, 342 potential target proteins and 22 signaling pathways. HSC-based *in vitro* experiments demonstrated that LS could inhibit the cell viability, promote the cell apoptosis, decrease the expression of liver fibrosis markers, as well as downregulate the JAK1/STAT3 signaling pathway. Our results suggested that LS could exert anti-liver-fibrosis effects by inhibiting the activation of HSCs, targeting JAK1 and STAT3, and regulating the JAK1/STAT3 signaling pathway.

LS has been reported to possess anti-oxidation, anti-inflammation, antitumor, phytoestrogens activity, vasodilation and other pharmacological activity (Wang et al., 2020). Among these indications, studies on the anti-hepatic fibrosis effects of LS are relatively few. Sung Hee Lee reported the anti-hepatic fibrosis effects of LS similar compounds, and identified that their anti-fibrotic mechanism involved reduced HSCs activation (Parajuli et al., 2015). Tanshinones, including tanshinone IIA, tanshinone I and dihydrotanshinone I, have been reported to treat liver fibrosis by inhibiting the activation of HSCs, regulating PI3K/Akt signaling pathways, disrupting the YAP /TEAD2 complex, and stimulating autophagy (Liu and Huang, 2014; Ge et al., 2017; Shi et al., 2020). However, the pharmacological mechanisms of LS on liver fibrosis have not been clarified and require further investigation.

Our network pharmacology strategy identified that the LS might exert anti-hepatic fibrosis primarily by regulating the JAK/STAT signaling pathway. The JAK/STAT pathway is one of the few multi-effect cascades that can be activated by a variety of cytokines, growth factors and hormones, thereby mediating a variety of cellular functions, including resistance to pathogens, differentiation, proliferation, apoptosis, metabolism, and cell transformation (Kagan et al., 2017). The cytokine binds to its corresponding receptor and activates the JAK family, which in turn activates STATs and induces their dimerization. Dimerized STATs are then transported to the nucleus to regulate the expression of target genes. Studies have shown that inhibiting the JAK/STAT signaling pathway can inhibit HSC activation and treat liver fibrosis (Handy et al., 2011; Kagan et al., 2017). Moreover, TGFβ can directly activate the JAK1/STAT3 axis in HSCs to induce liver fibrosis (Tang et al., 2017). Therefore, the JAK/STAT signaling pathway is closely related to the development of liver fibrosis.

STAT3 is a key protein in the JAK/STAT pathway. In chronic liver injury, damaged liver parenchymal cells, sinusoidal endothelial cells and Kupffer cells will release many cytokines, including TGFβ, IL-6 and others, which activate the phosphorylation of STAT3 (Kagan et al., 2017). Meng et al. (2012) reported that the deletion of STAT3 signaling in HSCs in mice attenuated liver fibrosis, and another study indicated that overexpression of STAT3 in HSCs promoted the proliferation, thereby inducing the formation of liver fibrosis (Su et al., 2015). In addition, STAT3 inhibitors could repress the migration and proliferation of activated HSCs, as well as attenuate CCl_4_-induced liver fibrosis (Wang Z. et al., 2018) JAK1 plays an important role in liver fibrosis through JAK/STAT signaling (Park et al., 2021). Eunsun Park reported that a JAK1-selective inhibitor reduced the proliferation, fibrogenic gene expression and JAK1/STAT3 pathway of TGFβ-induced HSCs (Gressner et al., 2002). Therefore, STAT3 and JAK1 are potential target proteins in HSCs for the treatment of liver fibrosis.

TGFβ1, which drives the transdifferentiation of phenotypical HSCs from quiescence to activation through paracrine and autocrine mechanisms, plays a critical role in the progression of HSC activation and liver fibrosis (Gressner et al., 2002). We tested the effects of LS on the TGFβ1-induced JAK1/STAT3 pathway and fibrosis markers (α-SMA and TGFβ1) in HSC-T6. LS significantly inhibited the TGFβ1-mediated expression of JAK1, P-JAK1, STAT3, P-STAT3, α-SMA and TGFβ1. However, TGFβ1 treatment did not affect the expression of P-STAT3, which is contradictory to known studies (Wang Z. et al., 2018; Lin et al., 2019). One explanation is that TGFβ1 treatment for 24 h cannot upregulate the expression of P-STAT3. Other modes and durations of TGFβ1 treatment must to be investigated. Even so, we can also conclude that LS can inhibit the expression of P-STAT3 in activated HSCs.

In conclusion, the network pharmacology results indicated that LS exerted an anti-hepatic fibrosis effect through 71 active compounds, 342 potential target proteins and 22 signaling pathways. Among these pathways, the JAK1/STAT3 signaling pathway was representatively selected for further experimental validation. *In vivo* animal experiments firstly confirmed the protective effect of LS in liver fibrosis. *In vitro* studies then demonstrated that LS could inhibit the HSC viability, promote the HSC apoptosis, decrease the expression of liver fibrosis markers, and downregulate the JAK1/STAT3 signaling pathway. This study is expected to benefit the clinical application of LS.

## Data Availability

The raw data supporting the conclusion of this article will be made available by the authors, without undue reservation.

## References

[B1] Aydin M. M. Akcali K. C. (2018). Liver Fibrosis. Turk J. Gastroenterol. 29 (1), 14–21. 10.5152/tjg.2018.17330 29391303 PMC6322608

[B2] Chen L. Teng H. Cao H. (2019). Chlorogenic Acid and Caffeic Acid from Sonchus Oleraceus Linn Synergistically Attenuate Insulin Resistance and Modulate Glucose Uptake in HepG2 Cells. Food Chem. Toxicol. 127, 182–187. 10.1016/j.fct.2019.03.038 30914352

[B3] Chen S. Jiang H. Cao Y. Wang Y. Hu Z. Zhu Z. (2016). Drug Target Identification Using Network Analysis: Taking Active Components in Sini Decoction as an Example. Sci. Rep. 6, 24245. 10.1038/srep24245 27095146 PMC4837341

[B4] Chen S. Wu S. Li W. Chen X. Dong X. Tan G. (2014). Investigation of the Therapeutic Effectiveness of Active Components in Sini Decoction by a Comprehensive GC/LC-MS Based Metabolomics and Network Pharmacology Approaches. Mol. Biosyst. 10, 3310–3321. 10.1039/c4mb00048j 25315049

[B5] Ge M. Liu H. Zhang Y. Li N. Zhao S. Zhao W. (2017). The Anti-Hepatic Fibrosis Effects of Dihydrotanshinone I are Mediated by Disrupting the Yes-Associated Protein and Transcriptional Enhancer Factor D2 Complex and Stimulating Autophagy. Br. J. Pharmacol. 174 (10), 1147–1160. 10.1111/bph.13766 28257144 PMC5406384

[B6] Gressner A. M. Weiskirchen R. Breitkopf K. Dooley S. (2002). Roles of TGF-Beta in Hepatic Fibrosis. Front. Biosci. 7, d793–807. 10.2741/A812 11897555

[B7] Handy J. A. Fu P. P. Kumar P. Mells J. E. Sharma S. Saxena N. K. (2011). Adiponectin Inhibits Leptin Signalling via Multiple Mechanisms to Exert Protective Effects Against Hepatic Fibrosis. Biochem. J. 440 (3), 385–395. 10.1042/BJ20102148 21846328 PMC3226855

[B8] Kagan P. Sultan M. Tachlytski I. Safran M. Ben-Ari Z. (2017). Both MAPK and STAT3 Signal Transduction Pathways are Necessary for IL-6-Dependent Hepatic Stellate Cells Activation. PLoS One 12 (5), e0176173. 10.1371/journal.pone.0176173 28472150 PMC5417441

[B9] Kostallari E. Hirsova P. Prasnicka A. Verma V. K. Yaqoob U. Wongjarupong N. (2018). Hepatic Stellate Cell-Derived Platelet-Derived Growth Factor Receptor-Alpha-Enriched Extracellular Vesicles Promote Liver Fibrosis in Mice through SHP2. Hepatology 68 (1), 333–348. 10.1002/hep.29803 29360139 PMC6033667

[B10] Liang Z. (2018). Effect of Tanshinone Capsules Combined with Entecavir on HBV-DNA Negative Rate and Liver Function in Patients with Chronic Hepatitis B and Liver Fibrosis. Strait Pharm. J. 30 (3), 119–120.

[B11] Lin I. Y. Chiou Y. S. Wu L. C. Tsai C. Y. Chen C. T. Chuang W. C. (2019). CCM111 Prevents Hepatic Fibrosis via Cooperative Inhibition of TGF-β, Wnt and STAT3 Signaling Pathways. J. Food Drug Anal. 27 (1), 184–194. 10.1016/j.jfda.2018.09.008 30648571 PMC9298635

[B12] Liu Y. W. Huang Y. T. (2014). Inhibitory Effect of Tanshinone IIA on Rat Hepatic Stellate Cells. PLoS One 9 (7), e103229. 10.1371/journal.pone.0103229 25076488 PMC4116159

[B13] Meng F. Wang K. Aoyama T. Grivennikov S. I. Paik Y. Scholten D. (2012). Interleukin-17 Signaling in Inflammatory, Kupffer Cells, and Hepatic Stellate Cells Exacerbates Liver Fibrosis in Mice. Gastroenterology 143 (3), 765–776.e3. 10.1053/j.gastro.2012.05.049 22687286 PMC3635475

[B14] Muegge I. Mukherjee P. (2016). An Overview of Molecular Fingerprint Similarity Search in Virtual Screening. Expert Opin. Drug Discov. 11 (2), 137–148. 10.1517/17460441.2016.1117070 26558489

[B15] Nallagangula K. S. Nagaraj S. K. Venkataswamy L. Chandrappa M. (2018). Liver Fibrosis: A Compilation on the Biomarkers Status and Their Significance during Disease Progression. Future Sci. OA 4 (1), FSO250. 10.4155/fsoa-2017-0083 29255622 PMC5729599

[B16] O’Boyle N. M. Banck M. James C. A. Morley C. Vandermeersch T. Hutchison G. R. (2011). Open Babel: An Open Chemical Toolbox. J. Cheminform 3, 33. 10.1186/1758-2946-3-33 21982300 PMC3198950

[B17] Parajuli D. R. Zhao Y. Z. Jin H. Chi J. H. Li S. Y. Kim Y. C. (2015). Anti-Fibrotic Effect of PF2401-SF, a Standardized Fraction of Salvia Miltiorrhiza, in Thioacetamide-Induced Experimental Rats Liver Fibrosis. Arch. Pharm. Res. 38 (4), 549–555. 10.1007/s12272-014-0425-2 25005065

[B18] Park E. Lee S. J. Moon H. Park J. Jeon H. Hwang J. S. (2021). Discovery and Biological Evaluation of N-Methyl-Pyrrolo[2,3-b]pyridine-5-Carboxamide Derivatives as JAK1-Selective Inhibitors. J. Med. Chem. 64 (2), 958–979. 10.1021/acs.jmedchem.0c01026 33428419

[B19] Reyes-Gordillo K. Shah R. Arellanes-Robledo J. Cheng Y. Ibrahim J. Tuma P. L. (2019). Akt1 and Akt2 Isoforms Play Distinct Roles in Regulating the Development of Inflammation and Fibrosis Associated with Alcoholic Liver Disease. Cells 8 (11), 1337. 10.3390/cells8111337 PMC6912497 31671832

[B20] Shi M. J. Yan X. L. Dong B. S. Yang W. N. Su S. B. Zhang H. (2020). A Network Pharmacology Approach to Investigating the Mechanism of Tanshinone IIA for the Treatment of Liver Fibrosis. J. Ethnopharmacol. 253, 112689. 10.1016/j.jep.2020.112689 32101775

[B21] Su T. H. Shiau C. W. Jao P. Liu C. H. Liu C. J. Tai W. T. (2015). Sorafenib and its Derivative SC-1 Exhibit Antifibrotic Effects through Signal Transducer and Activator of Transcription 3 Inhibition. Proc. Natl. Acad. Sci. U S A. 112 (23), 7243–7248. 10.1073/pnas.1507499112 26039995 PMC4466718

[B22] Su T. H. Shiau C. W. Jao P. Yang N. J. Tai W. T. Liu C. J. (2017). Src-Homology Protein Tyrosine Phosphatase-1 Agonist, SC-43, Reduces Liver Fibrosis. Sci. Rep. 7 (1), 1728. 10.1038/s41598-017-01572-z 28496142 PMC5431996

[B23] Tang L. Y. Heller M. Meng Z. Yu L. R. Tang Y. Zhou M. (2017). Transforming Growth Factor-β (TGF-β) Directly Activates the JAK1-STAT3 Axis to Induce Hepatic Fibrosis in Coordination with the SMAD Pathway. J. Biol. Chem. 292 (10), 4302–4312. 10.1074/jbc.M116.773085 28154170 PMC5354477

[B24] Trivella J. P. Martin P. Carrion A. F. (2020). Novel Targeted Therapies for the Management of Liver Fibrosis. Expert Opin. Emerg. Drugs 25, 59–70. 10.1080/14728214.2020.1735350 32098512

[B25] Vogel S. Piantedosi R. Frank J. Lalazar A. Rockey D. C. Friedman S. L. (2000). An Immortalized Rat Liver Stellate Cell Line (HSC-T6): a New Cell Model for the Study of Retinoid Metabolism *In Vitro* . J. Lipid Res. 41 (6), 882–893. 10.1016/S0022-2275(20)32030-7 10828080

[B26] Wang R. Wang J. Song F. Li S. Yuan Y. (2018). Tanshinol Ameliorates CCl4-Induced Liver Fibrosis in Rats through the Regulation of Nrf2/HO-1 and NF-κB/IκBα Signaling Pathway. Drug Des. Devel. Ther. 12, 1281–1292. 10.2147/DDDT.S159546 PMC5961642 29844659

[B27] Wang X. Yang Y. Liu X. Gao X. (2020). Pharmacological Properties of Tanshinones, the Natural Products from *Salvia miltiorrhiza* . Adv. Pharmacol. 87, 43–70. 10.1016/bs.apha.2019.10.001 32089238

[B28] Wang Z. Li J. Xiao W. Long J. Zhang H. (2018). The STAT3 Inhibitor S3I-201 Suppresses Fibrogenesis and Angiogenesis in Liver Fibrosis. Lab. Invest. 98 (12), 1600–1613. 10.1038/s41374-018-0127-3 30206312

[B29] Xing X. Chen S. Li L. Cao Y. Chen L. Wang X. (2018). The Active Components of Fuzheng Huayu Formula and Their Potential Mechanism of Action in Inhibiting the Hepatic Stellate Cells Viability - A Network Pharmacology and Transcriptomics Approach. Front. Pharmacol. 9, 525. 10.3389/fphar.2018.00525 29881350 PMC5976863

[B30] Xu L. Hui A. Y. Albanis E. Arthur M. J. O’Byrne S. M. Blaner W. S. (2005). Human Hepatic Stellate Cell Lines, LX-1 and LX-2: New Tools for Analysis of Hepatic Fibrosis. Gut 54 (1), 142–151. 10.1136/gut.2004.042127 15591520 PMC1774377

[B31] Xu M. Xu H. H. Lin Y. Sun X. Wang L. J. Fang Z. P. (2019). LECT2, a Ligand for Tie1, Plays a Crucial Role in Liver Fibrogenesis. Cell 178 (6), 1478–1492.e20. 10.1016/j.cell.2019.07.021 31474362

[B32] Yang G. Zhan J. Yang Y. Yuan L. Wang P. Ho C.-T. (2021). Inhibitory Effects of Oxyresveratrol on ERK and Smad1/2 Phosphorylation and HSC Activation in Preventing Carbon Tetrachloride-Induced Rat Liver Fibrosis. Food Sci. Hum. Wellness 10 (1), 6–12. 10.1016/j.fshw.2020.08.007

