# Supplementary material for: Lipophilic Constituents in Salvia miltiorrhiza Inhibit Activation of the Hepatic Stellate Cells by Suppressing the JAK1/STAT3 Signaling Pathway: A Network Pharmacology Study and Experimental Validation
Source: Front Pharmacol. 2022 Apr 20;13:770344. doi: 10.3389/fphar.2022.770344 (PMC9065469; doi:10.3389/fphar.2022.770344)

Supplementary Material

**Supplementary Table 1.** The chemical database of lipophilic constituents in *Salvia miltiorrhiza*

| No | Compound | Formula | Classification | No | Compound | Formula | Classification |
| --- | --- | --- | --- | --- | --- | --- | --- |
| 1 | tanshinone VI | C18H16O4 | Diterpenoids | 70 | miltirone I | C18H16O2 | Diterpenoids |
| 2 | 1,2,15,16-tetrahydrotanshiquinone (1,2,15,16-tetrahydrotanshinone I,Trijuganone B,Tetrahydro tanshinone I) | C18H16O3 | Diterpenoids | 71 | monodydroxytanshinone I | C18H12O4 | Diterpenoids |
| 3 | 1,2,3,4-tetrahydro-5-hydroxy-7-isopropyl-1,1-dimethyl-4-oxophenanthren-6-yl palmitate | C35H52O4 | Diterpenoids | 72 | neocryptotanshinone （tanshinone V） | C19H22O4 | Diterpenoids |
| 4 | 1,2-Didehydrocryptotanshinone | C19H18O3 | Diterpenoids | 73 | neocryptotanshinone II | C17H18O3 | Diterpenoids |
| 5 | 1,2-Dihydrotanshinquinone (1,2-Dihydrotanshinone I,1,2-Dihydrotanshinone) | C18H14O3 | Diterpenoids | 74 | neosalvianen | C21H21NO2 | Diterpenoids |
| 6 | 15,16-dihydrotanshinol B | C18H18O4 | Diterpenoids | 75 | neotanshinlactone | C17H12O3 | Diterpenoids |
| 7 | 17-hydroxycryptotanshinone | C19H20O4 | Diterpenoids | 76 | nortanshinone | C17H12O4 | Diterpenoids |
| 8 | 17-hydroxytanshindiol B | C18H18O5 | Diterpenoids | 77 | oleoyl danshenxinkun A | C36H48O5 | Diterpenoids |
| 9 | 1-hydroxytaxinine A | C26H36O9 | Diterpenoids | 78 | oleoyl neocryptotanshinone | C37H54O5 | Diterpenoids |
| 10 | 1-ketoaethiopinone | C20H22O3 | Diterpenoids | 79 | palmitoyl arucadiol | C35H52O4 | Diterpenoids |
| 11 | 1-ketoisocryptotanshinone | C19H18O4 | Diterpenoids | 80 | paramiltioic acid | C19H24O5 | Diterpenoids |
| 12 | 1-oxomiltirone | C19H20O3 | Diterpenoids | 81 | przewaquinone A | C19H18O4 | Diterpenoids |
| 13 | 1R-hydroxymiltirone | C19H22O3 | Diterpenoids | 82 | przewaquinone B | C18H12O4 | Diterpenoids |
| 14 | 2,3-Didehydrocryptotanshinone | C19H18O3 | Diterpenoids | 83 | salviadione | C19H19NO2 | Diterpenoids |
| 15 | 2alpha-acetoxysugiol | C22H30O4 | Diterpenoids | 84 | salviamone | C18H14O4 | Diterpenoids |
| 16 | 2-hydroxydihydroisotanshinone I | C18H14O4 | Diterpenoids | 85 | salvianan | C21H23NO2 | Diterpenoids |
| 17 | 2-Isopropyl-8-methylphenanthrene-3,4-dione (Ro 09-0680,miltirone I similar) | C18H16O2 | Diterpenoids | 86 | salvianen | C21H21NO2 | Diterpenoids |
| 18 | 3-hydroxycyptotanshinone | C19H20O4 | Diterpenoids | 87 | salvilenone | C20H20O2 | Diterpenoids |
| 19 | 3-hydroxymethylenetanshinquinone (hydroxymethylenetanshinone,3-beta-Hydroxymethylenetanshiquinone) | C18H14O4 | Diterpenoids | 88 | salvinone | C18H20O2 | Diterpenoids |
| 20 | 3-hydroxy-salvilenone | C20H20O3 | Diterpenoids | 89 | salviol （2-Hydroxyferruginol） | C20H30O2 | Diterpenoids |
| 21 | 3-hydroxytanshinone | C19H18O4 | Diterpenoids | 90 | salviolone | C18H20O2 | Diterpenoids |
| 22 | 3-hydroxytanshinone IIB | C19H18O5 | Diterpenoids | 91 | sugiol | C20H28O2 | Diterpenoids |
| 23 | 3-Oxosapriparaquinone | C20H24O4 | Diterpenoids | 92 | tanshinaldehyde （tanshinaldehyde II） | C19H18O4 | Diterpenoids |
| 24 | 4-methylenemiltirone (methylenemiltirone) | C18H18O2 | Diterpenoids | 93 | tanshinaldehyde I （formyltanshinone） | C18H10O4 | Diterpenoids |
| 25 | 5,6-dehydrosugiol | C20H26O2 | Diterpenoids | 94 | tanshindiol A | C18H16O5 | Diterpenoids |
| 26 | 6,12-Dihydroxyabieta-5,8,11,13-tetraen-7-one (montbretol) | C20H26O3 | Diterpenoids | 95 | tanshindiol B | C18H16O5 | Diterpenoids |
| 27 | 7β-hydroxy-8,13-abietadiene-11,12-dione | C20H28O3 | Diterpenoids | 96 | tanshindiol C | C18H16O5 | Diterpenoids |
| 28 | abietic acid | C20H30O2 | Diterpenoids | 97 | tanshinlactone | C17H12O3 | Diterpenoids |
| 29 | cryptoacetalide | C18H22O3 | Diterpenoids | 98 | tanshinol A (tanshinol I) | C18H12O4 | Diterpenoids |
| 30 | cryptotanshinone （15,17-Dihydrotanshinone IIA） | C19H20O3 | Diterpenoids | 99 | tanshinol B (Przewaquinone C) | C18H16O4 | Diterpenoids |
| 31 | danshenol a | C21H20O4 | Diterpenoids | 100 | tanshinone I | C18H12O3 | Diterpenoids |
| 32 | danshenol b | C22H26O4 | Diterpenoids | 101 | tanshinone II A | C19H18O3 | Diterpenoids |
| 33 | danshenxinkun A (neotanshinone A,Tanshiquinone A) | C18H16O4 | Diterpenoids | 102 | tanshinone II B | C19H18O4 | Diterpenoids |
| 34 | danshenxinkun B(tanshiquinone B,neotanshinone B) | C18H16O3 | Diterpenoids | 103 | gamma-sitosterol | C29H50O | Steroids |
| 35 | danshenxinkun C (Neotanshinone C,tanshiquinone C) | C16H12O3 | Diterpenoids | 104 | isocucurbitacin d | C30H44O7 | Steroids |
| 36 | danshenxinkun D | C21H20O4 | Diterpenoids | 105 | rutin | C27H30O16 | Steroids |
| 37 | Danshinspiroketallactone (Danshenspiroketallactone) | C17H16O3 | Diterpenoids | 106 | stigmasterol | C29H48O | Steroids |
| 38 | dehydromiltirone (1,2-didehydromiltirone,1-dehydromiltirone) | C19H20O2 | Diterpenoids | 107 | β-sitosterol | C29H50O | Steroids |
| 39 | Dehydrotanshinone II A (1,2-Didehydrotanshinone IIA,1,2-Dehydrotanshinone II A,delta1-Dehydrotanshinone II(A)) | C19H16O3 | Diterpenoids | 108 | daucosterol (Sitogluside,alexandrin) | C35H60O6 | Steroids |
| 40 | demethylcryptojaponol (11-Hydroxysugiol) | C20H28O3 | Diterpenoids | 109 | ailanthoidol | C19H18O5 | Others |
| 41 | deoxyneocryptotanshinone | C19H22O3 | Diterpenoids | 110 | 5-(methoxymethyl)-1H-pyrrole-2-carbaldehyde | C7H9NO2 | Others |
| 42 | dihydroisotanshinone I (isodihydrotanshinone) | C18H14O3 | Diterpenoids | 111 | 6-hydroxymethyllumazin | C7H6N4O3 | Others |
| 43 | dihydroisotanshinone II | C18H14O3 | Diterpenoids | 112 | cryptoxanthin | C40H56O | Others |
| 44 | dihydronortanshinone | C17H14O4 | Diterpenoids | 113 | daphneolone | C17H18O3 | Others |
| 45 | Dihydrotanshinone I (15,16-Dihydrotanshinone I) | C18H14O3 | Diterpenoids | 114 | dauricine | C38H44N2O6 | Others |
| 46 | Epi-Cryptoacetalide | C18H22O3 | Diterpenoids | 115 | isotenulin | C17H22O5 | Others |
| 47 | epidanshenspiroketallactone | C17H16O3 | Diterpenoids | 116 | Rehmanone C((E)-4-[5-(hydroxymethyl)furan-2-yl]but-3-en-2-one) | C9H10O3 | Others |
| 48 | epi-Danshenspiroketallactone | C20H20O5 | Diterpenoids | 117 | rosmarinine | C18H27NO6 | Others |
| 49 | ferruginol | C20H30O | Diterpenoids | 118 | sagecoumarin | C27H20O12 | Others |
| 50 | heteratisine | C22H33NO5 | Diterpenoids | 119 | taraxanthin | C40H56O3 | Others |
| 51 | hydroxytanshinone IIA (hydroxytanshinone) | C19H18O4 | Diterpenoids | 120 | salvinal (XH-14,5-(3-hydroxypropyl)-7-methoxy-2-(3′-methoxy-4′-hydroxyphenyl)-3-benzo[b]furancarbaldehyde) | C20H20O6 | Others |
| 52 | isocryptotanshinone(isodihydrotanshinone IIA) | C19H20O3 | Diterpenoids | 121 | O-methyltaxodine | C20H29NO3 | Others |
| 53 | isotanshinone I | C18H12O3 | Diterpenoids | 122 | Didrovaltrate (dihydrovalepotriate) | C22H32O8 | Others |
| 54 | isotanshinone II | C18H12O3 | Diterpenoids | 123 | Corosolic acid (colosolic acid,2alpha-hydroxyursolic acid) | C30H48O4 | Triterpenoids |
| 55 | isotanshinone IIA | C19H18O3 | Diterpenoids | 124 | euscaphic acid （tormentic acid，Jacarandic acid,2beta-hydroxypomolic acid） | C30H48O5 | Triterpenoids |
| 56 | isotanshinone IIB (18-hydroxyisotanshinone IIA) | C19H18O4 | Diterpenoids | 125 | maslinic acid | C30H48O4 | Triterpenoids |
| 57 | methyl dihydronortanshinonate（methylcryptotanshinoate） | C20H20O5 | Diterpenoids | 126 | neotigogenin | C27H44O3 | Triterpenoids |
| 58 | methyl tanshinonate (methyltanshinoate) | C20H18O5 | Diterpenoids | 127 | oleanolic acid | C30H48O3 | Triterpenoids |
| 59 | methylene dihydrotanshinone | C18H16O3 | Diterpenoids | 128 | pomolic acid | C30H48O4 | Triterpenoids |
| 60 | Methylenetanshinquinone (methylenetanshinone) | C18H14O3 | Diterpenoids | 129 | przewanoic acid A | C30H46O4 | Triterpenoids |
| 61 | miltiodiol (Arucadiol) | C19H22O3 | Diterpenoids | 130 | przewanoic acid B | C29H42O4 | Triterpenoids |
| 62 | miltionone I | C19H20O4 | Diterpenoids | 131 | taraxerol | C30H50O | Triterpenoids |
| 63 | miltionone II | C19H20O4 | Diterpenoids | 132 | Tigogenin | C27H44O3 | Triterpenoids |
| 64 | miltiorin A (2alpha-Acetoxyabieta-8(14),9(11),12-triene-12-ol) | C22H32O3 | Diterpenoids | 133 | ursolic acid | C30H48O3 | Triterpenoids |
| 65 | miltiorin B | C22H30O5 | Diterpenoids | 134 | uvaol | C30H50O2 | Triterpenoids |
| 66 | miltiorin C | C22H28O4 | Diterpenoids | 135 | 3-O-acetyloleanolic acid (Acetyl oleanolic acid, Acetyloleanolic acid) | C32H50O4 | Triterpenoids |
| 67 | miltiorin D | C19H22O4 | Diterpenoids | 136 | barbinervic acid | C30H48O5 | Triterpenoids |
| 68 | miltipolone | C19H24O3 | Diterpenoids | 137 | Asiatic acid (Dammarolic acid,Asiantic acid,2alpha,23-Dihydroxyursolic acid) | C30H48O5 | Triterpenoids |
| 69 | miltirone | C19H22O2 | Diterpenoids | 138 | urs-12-ene-2α,3β,7β,16α-tetraol | C30H50O4 | Triterpenoids |

**Supplementary Table 2.** The potential target proteins of lipophilic constituents in Salvia miltiorrhiza

| No | compound | compound category | Uniprot ID | average (probability) | Count |
| --- | --- | --- | --- | --- | --- |
| 1 | 1,2,15,16-tetrahydrotanshiquinone (1,2,15,16-tetrahydrotanshinone I,Trijuganone B) | Diterpenoids | B2RXH2 | 0.104871629 | 12 |
| 2 | 1,2,15,16-tetrahydrotanshiquinone (1,2,15,16-tetrahydrotanshinone I,Trijuganone B) | Diterpenoids | O00748 | 0.259386615 | 47 |
| 3 | 1,2,15,16-tetrahydrotanshiquinone (1,2,15,16-tetrahydrotanshinone I,Trijuganone B) | Diterpenoids | O75874 | 0.1046882 | 6 |
| 4 | 1,2,15,16-tetrahydrotanshiquinone (1,2,15,16-tetrahydrotanshinone I,Trijuganone B) | Diterpenoids | O95551 | 0.106334502 | 10 |
| 5 | 1,2,15,16-tetrahydrotanshiquinone (1,2,15,16-tetrahydrotanshinone I,Trijuganone B) | Diterpenoids | O96020 P24941 P24864 | 0.104035866 | 9 |
| 6 | 1,2,15,16-tetrahydrotanshiquinone (1,2,15,16-tetrahydrotanshinone I,Trijuganone B) | Diterpenoids | P00533 | 0.104151342 | 24 |
| 7 | 1,2,15,16-tetrahydrotanshiquinone (1,2,15,16-tetrahydrotanshinone I,Trijuganone B) | Diterpenoids | P04626 | 0.104502793 | 11 |
| 8 | 1,2,15,16-tetrahydrotanshiquinone (1,2,15,16-tetrahydrotanshinone I,Trijuganone B) | Diterpenoids | P08575 | 0.109148612 | 27 |
| 9 | 1,2,15,16-tetrahydrotanshiquinone (1,2,15,16-tetrahydrotanshinone I,Trijuganone B) | Diterpenoids | P11387 | 0.113455556 | 13 |
| 10 | 1,2,15,16-tetrahydrotanshiquinone (1,2,15,16-tetrahydrotanshinone I,Trijuganone B) | Diterpenoids | P11388 | 0.106757964 | 8 |
| 11 | 1,2,15,16-tetrahydrotanshiquinone (1,2,15,16-tetrahydrotanshinone I,Trijuganone B) | Diterpenoids | P14902 | 0.110454469 | 24 |
| 12 | 1,2,15,16-tetrahydrotanshiquinone (1,2,15,16-tetrahydrotanshinone I,Trijuganone B) | Diterpenoids | P15121 | 0.267155221 | 39 |
| 13 | 1,2,15,16-tetrahydrotanshiquinone (1,2,15,16-tetrahydrotanshinone I,Trijuganone B) | Diterpenoids | P21980 | 0.101957459 | 8 |
| 14 | 1,2,15,16-tetrahydrotanshiquinone (1,2,15,16-tetrahydrotanshinone I,Trijuganone B) | Diterpenoids | P22303 | 0.287353637 | 26 |
| 15 | 1,2,15,16-tetrahydrotanshiquinone (1,2,15,16-tetrahydrotanshinone I,Trijuganone B) | Diterpenoids | P23141 | 0.275271523 | 31 |
| 16 | 1,2,15,16-tetrahydrotanshiquinone (1,2,15,16-tetrahydrotanshinone I,Trijuganone B) | Diterpenoids | P24864 P24941 | 0.102540734 | 7 |
| 17 | 1,2,15,16-tetrahydrotanshiquinone (1,2,15,16-tetrahydrotanshinone I,Trijuganone B) | Diterpenoids | P28472 P18507 P14867 | 0.103855577 | 16 |
| 18 | 1,2,15,16-tetrahydrotanshiquinone (1,2,15,16-tetrahydrotanshinone I,Trijuganone B) | Diterpenoids | P28472 P18507 P31644 | 0.103791839 | 19 |
| 19 | 1,2,15,16-tetrahydrotanshiquinone (1,2,15,16-tetrahydrotanshinone I,Trijuganone B) | Diterpenoids | P28472 P34903 P18507 | 0.103855577 | 16 |
| 20 | 1,2,15,16-tetrahydrotanshiquinone (1,2,15,16-tetrahydrotanshinone I,Trijuganone B) | Diterpenoids | P28562 | 0.104003317 | 12 |
| 21 | 1,2,15,16-tetrahydrotanshiquinone (1,2,15,16-tetrahydrotanshinone I,Trijuganone B) | Diterpenoids | P29350 | 0.204438393 | 44 |
| 22 | 1,2,15,16-tetrahydrotanshiquinone (1,2,15,16-tetrahydrotanshinone I,Trijuganone B) | Diterpenoids | P35968 | 0.105052994 | 26 |
| 23 | 1,2,15,16-tetrahydrotanshiquinone (1,2,15,16-tetrahydrotanshinone I,Trijuganone B) | Diterpenoids | P40763 | 0.268996335 | 13 |
| 24 | 1,2,15,16-tetrahydrotanshiquinone (1,2,15,16-tetrahydrotanshinone I,Trijuganone B) | Diterpenoids | P42336 | 0.108042586 | 13 |
| 25 | 1,2,15,16-tetrahydrotanshiquinone (1,2,15,16-tetrahydrotanshinone I,Trijuganone B) | Diterpenoids | P45983 | 0.105088123 | 17 |
| 26 | 1,2,15,16-tetrahydrotanshiquinone (1,2,15,16-tetrahydrotanshinone I,Trijuganone B) | Diterpenoids | P47869 P28472 P18507 | 0.104254314 | 15 |
| 27 | 1,2,15,16-tetrahydrotanshiquinone (1,2,15,16-tetrahydrotanshinone I,Trijuganone B) | Diterpenoids | Q06124 | 0.211032909 | 40 |
| 28 | 1,2,15,16-tetrahydrotanshiquinone (1,2,15,16-tetrahydrotanshinone I,Trijuganone B) | Diterpenoids | Q13255 | 0.103848909 | 9 |
| 29 | 1,2,15,16-tetrahydrotanshiquinone (1,2,15,16-tetrahydrotanshinone I,Trijuganone B) | Diterpenoids | Q9UDY8 | 0.107158923 | 10 |
| 30 | 1,2-Didehydrocryptotanshinone | Diterpenoids | B2RXH2 | 0.104871629 | 12 |
| 31 | 1,2-Didehydrocryptotanshinone | Diterpenoids | O00748 | 0.259386615 | 47 |
| 32 | 1,2-Didehydrocryptotanshinone | Diterpenoids | O43570 | 0.137972322 | 10 |
| 33 | 1,2-Didehydrocryptotanshinone | Diterpenoids | O95551 | 0.106334502 | 10 |
| 34 | 1,2-Didehydrocryptotanshinone | Diterpenoids | P00915 | 0.106312298 | 8 |
| 35 | 1,2-Didehydrocryptotanshinone | Diterpenoids | P08575 | 0.109148612 | 27 |
| 36 | 1,2-Didehydrocryptotanshinone | Diterpenoids | P0DMS8 | 0.10464569 | 23 |
| 37 | 1,2-Didehydrocryptotanshinone | Diterpenoids | P14902 | 0.110454469 | 24 |
| 38 | 1,2-Didehydrocryptotanshinone | Diterpenoids | P15121 | 0.267155221 | 39 |
| 39 | 1,2-Didehydrocryptotanshinone | Diterpenoids | P22303 | 0.287353637 | 26 |
| 40 | 1,2-Didehydrocryptotanshinone | Diterpenoids | P23141 | 0.275271523 | 31 |
| 41 | 1,2-Didehydrocryptotanshinone | Diterpenoids | P28562 | 0.104003317 | 12 |
| 42 | 1,2-Didehydrocryptotanshinone | Diterpenoids | P29350 | 0.204438393 | 44 |
| 43 | 1,2-Didehydrocryptotanshinone | Diterpenoids | P30305 | 0.185734372 | 40 |
| 44 | 1,2-Didehydrocryptotanshinone | Diterpenoids | P35968 | 0.105052994 | 26 |
| 45 | 1,2-Didehydrocryptotanshinone | Diterpenoids | P40763 | 0.268996335 | 13 |
| 46 | 1,2-Didehydrocryptotanshinone | Diterpenoids | P42574 | 0.10400953 | 13 |
| 47 | 1,2-Didehydrocryptotanshinone | Diterpenoids | P43166 | 0.165991461 | 6 |
| 48 | 1,2-Didehydrocryptotanshinone | Diterpenoids | P51532 | 0.1046882 | 4 |
| 49 | 1,2-Didehydrocryptotanshinone | Diterpenoids | P55210 | 0.10400953 | 13 |
| 50 | 1,2-Didehydrocryptotanshinone | Diterpenoids | Q06124 | 0.211032909 | 40 |
| 51 | 1,2-Didehydrocryptotanshinone | Diterpenoids | Q16790 | 0.103433429 | 9 |
| 52 | 1,2-Didehydrocryptotanshinone | Diterpenoids | Q86U86 | 0.1046882 | 4 |
| 53 | 1,2-Didehydrocryptotanshinone | Diterpenoids | Q9UDY8 | 0.107158923 | 10 |
| 54 | 1,2-Dihydrotanshinquinone (1,2-Dihydrotanshinone I,1,2-Dihydrotanshinone) | Diterpenoids | O00748 | 0.259386615 | 47 |
| 55 | 1,2-Dihydrotanshinquinone (1,2-Dihydrotanshinone I,1,2-Dihydrotanshinone) | Diterpenoids | O14746 | 0.14728518 | 27 |
| 56 | 1,2-Dihydrotanshinquinone (1,2-Dihydrotanshinone I,1,2-Dihydrotanshinone) | Diterpenoids | O75530 Q15022 Q15910 | 0.341964133 | 19 |
| 57 | 1,2-Dihydrotanshinquinone (1,2-Dihydrotanshinone I,1,2-Dihydrotanshinone) | Diterpenoids | P08575 | 0.109148612 | 27 |
| 58 | 1,2-Dihydrotanshinquinone (1,2-Dihydrotanshinone I,1,2-Dihydrotanshinone) | Diterpenoids | P10275 | 0.132131799 | 34 |
| 59 | 1,2-Dihydrotanshinquinone (1,2-Dihydrotanshinone I,1,2-Dihydrotanshinone) | Diterpenoids | P11511 | 0.139056613 | 29 |
| 60 | 1,2-Dihydrotanshinquinone (1,2-Dihydrotanshinone I,1,2-Dihydrotanshinone) | Diterpenoids | P11940 | 0.1046882 | 6 |
| 61 | 1,2-Dihydrotanshinquinone (1,2-Dihydrotanshinone I,1,2-Dihydrotanshinone) | Diterpenoids | P15121 | 0.267155221 | 39 |
| 62 | 1,2-Dihydrotanshinquinone (1,2-Dihydrotanshinone I,1,2-Dihydrotanshinone) | Diterpenoids | P23141 | 0.275271523 | 31 |
| 63 | 1,2-Dihydrotanshinquinone (1,2-Dihydrotanshinone I,1,2-Dihydrotanshinone) | Diterpenoids | P25025 | 0.100320282 | 13 |
| 64 | 1,2-Dihydrotanshinquinone (1,2-Dihydrotanshinone I,1,2-Dihydrotanshinone) | Diterpenoids | P27338 | 0.105021471 | 16 |
| 65 | 1,2-Dihydrotanshinquinone (1,2-Dihydrotanshinone I,1,2-Dihydrotanshinone) | Diterpenoids | P29350 | 0.204438393 | 44 |
| 66 | 1,2-Dihydrotanshinquinone (1,2-Dihydrotanshinone I,1,2-Dihydrotanshinone) | Diterpenoids | P30305 | 0.185734372 | 40 |
| 67 | 1,2-Dihydrotanshinquinone (1,2-Dihydrotanshinone I,1,2-Dihydrotanshinone) | Diterpenoids | P30307 | 0.105546712 | 12 |
| 68 | 1,2-Dihydrotanshinquinone (1,2-Dihydrotanshinone I,1,2-Dihydrotanshinone) | Diterpenoids | P35348 | 0.101281367 | 4 |
| 69 | 1,2-Dihydrotanshinquinone (1,2-Dihydrotanshinone I,1,2-Dihydrotanshinone) | Diterpenoids | P48039 | 0.101354935 | 19 |
| 70 | 1,2-Dihydrotanshinquinone (1,2-Dihydrotanshinone I,1,2-Dihydrotanshinone) | Diterpenoids | P49286 | 0.101354935 | 19 |
| 71 | 1,2-Dihydrotanshinquinone (1,2-Dihydrotanshinone I,1,2-Dihydrotanshinone) | Diterpenoids | Q06124 | 0.211032909 | 40 |
| 72 | 1,2-Dihydrotanshinquinone (1,2-Dihydrotanshinone I,1,2-Dihydrotanshinone) | Diterpenoids | Q09028 Q16576 O75530 Q15022 Q15910 | 0.142197972 | 14 |
| 73 | 1,2-Dihydrotanshinquinone (1,2-Dihydrotanshinone I,1,2-Dihydrotanshinone) | Diterpenoids | Q99572 | 0.10437669 | 25 |
| 74 | 15,16-dihydrotanshinol B | Diterpenoids | B2RXH2 | 0.104871629 | 12 |
| 75 | 15,16-dihydrotanshinol B | Diterpenoids | O00748 | 0.259386615 | 47 |
| 76 | 15,16-dihydrotanshinol B | Diterpenoids | O14757 | 0.106739614 | 8 |
| 77 | 15,16-dihydrotanshinol B | Diterpenoids | O14965 | 0.107316885 | 8 |
| 78 | 15,16-dihydrotanshinol B | Diterpenoids | O43570 | 0.137972322 | 10 |
| 79 | 15,16-dihydrotanshinol B | Diterpenoids | O60706 | 0.103819513 | 5 |
| 80 | 15,16-dihydrotanshinol B | Diterpenoids | O60725 | 0.108205862 | 3 |
| 81 | 15,16-dihydrotanshinol B | Diterpenoids | O60885 | 0.104476685 | 6 |
| 82 | 15,16-dihydrotanshinol B | Diterpenoids | O75530 Q15022 Q15910 | 0.341964133 | 19 |
| 83 | 15,16-dihydrotanshinol B | Diterpenoids | O95551 | 0.106334502 | 10 |
| 84 | 15,16-dihydrotanshinol B | Diterpenoids | O96020 P24941 P24864 | 0.104035866 | 9 |
| 85 | 15,16-dihydrotanshinol B | Diterpenoids | P00533 | 0.104151342 | 24 |
| 86 | 15,16-dihydrotanshinol B | Diterpenoids | P00797 | 0.104471265 | 3 |
| 87 | 15,16-dihydrotanshinol B | Diterpenoids | P00915 | 0.106312298 | 8 |
| 88 | 15,16-dihydrotanshinol B | Diterpenoids | P00918 | 0.141292356 | 8 |
| 89 | 15,16-dihydrotanshinol B | Diterpenoids | P01375 | 0.108038789 | 7 |
| 90 | 15,16-dihydrotanshinol B | Diterpenoids | P03956 | 0.109842942 | 8 |
| 91 | 15,16-dihydrotanshinol B | Diterpenoids | P04626 | 0.104502793 | 11 |
| 92 | 15,16-dihydrotanshinol B | Diterpenoids | P04818 | 0.111100851 | 4 |
| 93 | 15,16-dihydrotanshinol B | Diterpenoids | P05186 | 0.104336352 | 9 |
| 94 | 15,16-dihydrotanshinol B | Diterpenoids | P06737 | 0.103331553 | 10 |
| 95 | 15,16-dihydrotanshinol B | Diterpenoids | P08172 | 0.117318329 | 18 |
| 96 | 15,16-dihydrotanshinol B | Diterpenoids | P08254 | 0.109296209 | 8 |
| 97 | 15,16-dihydrotanshinol B | Diterpenoids | P08575 | 0.109148612 | 27 |
| 98 | 15,16-dihydrotanshinol B | Diterpenoids | P08581 | 0.109668623 | 6 |
| 99 | 15,16-dihydrotanshinol B | Diterpenoids | P09874 | 0.106818993 | 18 |
| 100 | 15,16-dihydrotanshinol B | Diterpenoids | P0DMS8 | 0.10464569 | 23 |
| 101 | 15,16-dihydrotanshinol B | Diterpenoids | P10275 | 0.132131799 | 34 |
| 102 | 15,16-dihydrotanshinol B | Diterpenoids | P11229 | 0.103603883 | 11 |
| 103 | 15,16-dihydrotanshinol B | Diterpenoids | P11309 | 0.104805981 | 13 |
| 104 | 15,16-dihydrotanshinol B | Diterpenoids | P11473 | 0.109776491 | 5 |
| 105 | 15,16-dihydrotanshinol B | Diterpenoids | P12268 | 0.104524224 | 13 |
| 106 | 15,16-dihydrotanshinol B | Diterpenoids | P14780 | 0.106689076 | 6 |
| 107 | 15,16-dihydrotanshinol B | Diterpenoids | P14902 | 0.110454469 | 24 |
| 108 | 15,16-dihydrotanshinol B | Diterpenoids | P15056 | 0.101798823 | 4 |
| 109 | 15,16-dihydrotanshinol B | Diterpenoids | P15121 | 0.267155221 | 39 |
| 110 | 15,16-dihydrotanshinol B | Diterpenoids | P20309 | 0.105165619 | 10 |
| 111 | 15,16-dihydrotanshinol B | Diterpenoids | P22303 | 0.287353637 | 26 |
| 112 | 15,16-dihydrotanshinol B | Diterpenoids | P22460 | 0.106959422 | 3 |
| 113 | 15,16-dihydrotanshinol B | Diterpenoids | P23141 | 0.275271523 | 31 |
| 114 | 15,16-dihydrotanshinol B | Diterpenoids | P23219 | 0.109524644 | 16 |
| 115 | 15,16-dihydrotanshinol B | Diterpenoids | P23458 | 0.102916912 | 11 |
| 116 | 15,16-dihydrotanshinol B | Diterpenoids | P24864 P24941 | 0.102540734 | 7 |
| 117 | 15,16-dihydrotanshinol B | Diterpenoids | P24941 | 0.106080884 | 8 |
| 118 | 15,16-dihydrotanshinol B | Diterpenoids | P28482 | 0.111100851 | 4 |
| 119 | 15,16-dihydrotanshinol B | Diterpenoids | P28562 | 0.104003317 | 12 |
| 120 | 15,16-dihydrotanshinol B | Diterpenoids | P29274 | 0.103202685 | 27 |
| 121 | 15,16-dihydrotanshinol B | Diterpenoids | P29350 | 0.204438393 | 44 |
| 122 | 15,16-dihydrotanshinol B | Diterpenoids | P29371 | 0.103946422 | 5 |
| 123 | 15,16-dihydrotanshinol B | Diterpenoids | P29466 | 0.105464394 | 4 |
| 124 | 15,16-dihydrotanshinol B | Diterpenoids | P29597 | 0.106959422 | 3 |
| 125 | 15,16-dihydrotanshinol B | Diterpenoids | P30305 | 0.185734372 | 40 |
| 126 | 15,16-dihydrotanshinol B | Diterpenoids | P30542 | 0.101318267 | 24 |
| 127 | 15,16-dihydrotanshinol B | Diterpenoids | P32246 | 0.109647095 | 6 |
| 128 | 15,16-dihydrotanshinol B | Diterpenoids | P35968 | 0.105052994 | 26 |
| 129 | 15,16-dihydrotanshinol B | Diterpenoids | P40763 | 0.268996335 | 13 |
| 130 | 15,16-dihydrotanshinol B | Diterpenoids | P41145 | 0.107282863 | 6 |
| 131 | 15,16-dihydrotanshinol B | Diterpenoids | P43166 | 0.165991461 | 6 |
| 132 | 15,16-dihydrotanshinol B | Diterpenoids | P45983 | 0.105088123 | 17 |
| 133 | 15,16-dihydrotanshinol B | Diterpenoids | P49810 Q9NZ42 Q92542 Q96BI3 P49768 Q8WW43 | 0.101543467 | 9 |
| 134 | 15,16-dihydrotanshinol B | Diterpenoids | P50406 | 0.106847218 | 8 |
| 135 | 15,16-dihydrotanshinol B | Diterpenoids | P55263 | 0.107485158 | 3 |
| 136 | 15,16-dihydrotanshinol B | Diterpenoids | P56817 | 0.108325485 | 12 |
| 137 | 15,16-dihydrotanshinol B | Diterpenoids | P78536 | 0.104291862 | 9 |
| 138 | 15,16-dihydrotanshinol B | Diterpenoids | Q00987 | 0.107095673 | 11 |
| 139 | 15,16-dihydrotanshinol B | Diterpenoids | Q02750 | 0.106463868 | 11 |
| 140 | 15,16-dihydrotanshinol B | Diterpenoids | Q06124 | 0.211032909 | 40 |
| 141 | 15,16-dihydrotanshinol B | Diterpenoids | Q07343 | 0.107582642 | 6 |
| 142 | 15,16-dihydrotanshinol B | Diterpenoids | Q12809 | 0.105584236 | 3 |
| 143 | 15,16-dihydrotanshinol B | Diterpenoids | Q12866 | 0.103756913 | 4 |
| 144 | 15,16-dihydrotanshinol B | Diterpenoids | Q13547 | 0.110937969 | 6 |
| 145 | 15,16-dihydrotanshinol B | Diterpenoids | Q13627 | 0.104383674 | 7 |
| 146 | 15,16-dihydrotanshinol B | Diterpenoids | Q13946 | 0.104692547 | 7 |
| 147 | 15,16-dihydrotanshinol B | Diterpenoids | Q14790 | 0.104694286 | 5 |
| 148 | 15,16-dihydrotanshinol B | Diterpenoids | Q15078 Q00535 | 0.102910354 | 14 |
| 149 | 15,16-dihydrotanshinol B | Diterpenoids | Q15761 | 0.10562303 | 4 |
| 150 | 15,16-dihydrotanshinol B | Diterpenoids | Q16539 | 0.105438848 | 20 |
| 151 | 15,16-dihydrotanshinol B | Diterpenoids | Q16790 | 0.103433429 | 9 |
| 152 | 15,16-dihydrotanshinol B | Diterpenoids | Q16875 | 0.101683939 | 4 |
| 153 | 15,16-dihydrotanshinol B | Diterpenoids | Q5S007 | 0.102368749 | 9 |
| 154 | 15,16-dihydrotanshinol B | Diterpenoids | Q92793 | 0.102416978 | 3 |
| 155 | 15,16-dihydrotanshinol B | Diterpenoids | Q99572 | 0.10437669 | 25 |
| 156 | 15,16-dihydrotanshinol B | Diterpenoids | Q9NWZ3 | 0.105136637 | 5 |
| 157 | 15,16-dihydrotanshinol B | Diterpenoids | Q9P1W9 | 0.103344117 | 12 |
| 158 | 15,16-dihydrotanshinol B | Diterpenoids | Q9UBN7 | 0.106238718 | 3 |
| 159 | 15,16-dihydrotanshinol B | Diterpenoids | Q9UDY8 | 0.107158923 | 10 |
| 160 | 15,16-dihydrotanshinol B | Diterpenoids | Q9Y5Z0 | 0.106959422 | 3 |
| 161 | 2,3-Didehydrocryptotanshinone | Diterpenoids | B2RXH2 | 0.104871629 | 12 |
| 162 | 2,3-Didehydrocryptotanshinone | Diterpenoids | O00748 | 0.259386615 | 47 |
| 163 | 2,3-Didehydrocryptotanshinone | Diterpenoids | O43570 | 0.137972322 | 10 |
| 164 | 2,3-Didehydrocryptotanshinone | Diterpenoids | O95551 | 0.106334502 | 10 |
| 165 | 2,3-Didehydrocryptotanshinone | Diterpenoids | P00533 | 0.104151342 | 24 |
| 166 | 2,3-Didehydrocryptotanshinone | Diterpenoids | P00915 | 0.106312298 | 8 |
| 167 | 2,3-Didehydrocryptotanshinone | Diterpenoids | P04626 | 0.104502793 | 11 |
| 168 | 2,3-Didehydrocryptotanshinone | Diterpenoids | P08575 | 0.109148612 | 27 |
| 169 | 2,3-Didehydrocryptotanshinone | Diterpenoids | P0DMS8 | 0.10464569 | 23 |
| 170 | 2,3-Didehydrocryptotanshinone | Diterpenoids | P14902 | 0.110454469 | 24 |
| 171 | 2,3-Didehydrocryptotanshinone | Diterpenoids | P15121 | 0.267155221 | 39 |
| 172 | 2,3-Didehydrocryptotanshinone | Diterpenoids | P22303 | 0.287353637 | 26 |
| 173 | 2,3-Didehydrocryptotanshinone | Diterpenoids | P23141 | 0.275271523 | 31 |
| 174 | 2,3-Didehydrocryptotanshinone | Diterpenoids | P28562 | 0.104003317 | 12 |
| 175 | 2,3-Didehydrocryptotanshinone | Diterpenoids | P29350 | 0.204438393 | 44 |
| 176 | 2,3-Didehydrocryptotanshinone | Diterpenoids | P35968 | 0.105052994 | 26 |
| 177 | 2,3-Didehydrocryptotanshinone | Diterpenoids | P40763 | 0.268996335 | 13 |
| 178 | 2,3-Didehydrocryptotanshinone | Diterpenoids | P42336 | 0.108042586 | 13 |
| 179 | 2,3-Didehydrocryptotanshinone | Diterpenoids | P42574 | 0.10400953 | 13 |
| 180 | 2,3-Didehydrocryptotanshinone | Diterpenoids | P43166 | 0.165991461 | 6 |
| 181 | 2,3-Didehydrocryptotanshinone | Diterpenoids | P51532 | 0.1046882 | 4 |
| 182 | 2,3-Didehydrocryptotanshinone | Diterpenoids | P55210 | 0.10400953 | 13 |
| 183 | 2,3-Didehydrocryptotanshinone | Diterpenoids | Q06124 | 0.211032909 | 40 |
| 184 | 2,3-Didehydrocryptotanshinone | Diterpenoids | Q16790 | 0.103433429 | 9 |
| 185 | 2,3-Didehydrocryptotanshinone | Diterpenoids | Q86U86 | 0.1046882 | 4 |
| 186 | 2,3-Didehydrocryptotanshinone | Diterpenoids | Q9UDY8 | 0.107158923 | 10 |
| 187 | 2-hydroxydihydroisotanshinone I | Diterpenoids | O00329 | 0.105574288 | 6 |
| 188 | 2-hydroxydihydroisotanshinone I | Diterpenoids | O00408 | 0.103882897 | 9 |
| 189 | 2-hydroxydihydroisotanshinone I | Diterpenoids | O14965 | 0.107316885 | 8 |
| 190 | 2-hydroxydihydroisotanshinone I | Diterpenoids | O15530 | 0.110104207 | 4 |
| 191 | 2-hydroxydihydroisotanshinone I | Diterpenoids | O75460 | 0.105470076 | 5 |
| 192 | 2-hydroxydihydroisotanshinone I | Diterpenoids | O76074 | 0.103099774 | 12 |
| 193 | 2-hydroxydihydroisotanshinone I | Diterpenoids | O96020 P24941 P24864 | 0.104035866 | 9 |
| 194 | 2-hydroxydihydroisotanshinone I | Diterpenoids | P00519 | 0.104821195 | 5 |
| 195 | 2-hydroxydihydroisotanshinone I | Diterpenoids | P00533 | 0.104151342 | 24 |
| 196 | 2-hydroxydihydroisotanshinone I | Diterpenoids | P00915 | 0.106312298 | 8 |
| 197 | 2-hydroxydihydroisotanshinone I | Diterpenoids | P00918 | 0.141292356 | 8 |
| 198 | 2-hydroxydihydroisotanshinone I | Diterpenoids | P01375 | 0.108038789 | 7 |
| 199 | 2-hydroxydihydroisotanshinone I | Diterpenoids | P03956 | 0.109842942 | 8 |
| 200 | 2-hydroxydihydroisotanshinone I | Diterpenoids | P04278 | 0.16719946 | 19 |
| 201 | 2-hydroxydihydroisotanshinone I | Diterpenoids | P05186 | 0.104336352 | 9 |
| 202 | 2-hydroxydihydroisotanshinone I | Diterpenoids | P06239 | 0.102843593 | 5 |
| 203 | 2-hydroxydihydroisotanshinone I | Diterpenoids | P07949 | 0.103471701 | 8 |
| 204 | 2-hydroxydihydroisotanshinone I | Diterpenoids | P08246 | 0.10362417 | 7 |
| 205 | 2-hydroxydihydroisotanshinone I | Diterpenoids | P08253 | 0.106581489 | 9 |
| 206 | 2-hydroxydihydroisotanshinone I | Diterpenoids | P08254 | 0.109296209 | 8 |
| 207 | 2-hydroxydihydroisotanshinone I | Diterpenoids | P08581 | 0.109668623 | 6 |
| 208 | 2-hydroxydihydroisotanshinone I | Diterpenoids | P08908 | 0.10562303 | 4 |
| 209 | 2-hydroxydihydroisotanshinone I | Diterpenoids | P09874 | 0.106818993 | 18 |
| 210 | 2-hydroxydihydroisotanshinone I | Diterpenoids | P11362 | 0.105847024 | 4 |
| 211 | 2-hydroxydihydroisotanshinone I | Diterpenoids | P11511 | 0.139056613 | 29 |
| 212 | 2-hydroxydihydroisotanshinone I | Diterpenoids | P12268 | 0.104524224 | 13 |
| 213 | 2-hydroxydihydroisotanshinone I | Diterpenoids | P12931 | 0.104821195 | 5 |
| 214 | 2-hydroxydihydroisotanshinone I | Diterpenoids | P14780 | 0.106689076 | 6 |
| 215 | 2-hydroxydihydroisotanshinone I | Diterpenoids | P14902 | 0.110454469 | 24 |
| 216 | 2-hydroxydihydroisotanshinone I | Diterpenoids | P15121 | 0.267155221 | 39 |
| 217 | 2-hydroxydihydroisotanshinone I | Diterpenoids | P16083 | 0.112425971 | 14 |
| 218 | 2-hydroxydihydroisotanshinone I | Diterpenoids | P17948 | 0.105554897 | 13 |
| 219 | 2-hydroxydihydroisotanshinone I | Diterpenoids | P19320 | 0.105155078 | 5 |
| 220 | 2-hydroxydihydroisotanshinone I | Diterpenoids | P21554 | 0.104626059 | 10 |
| 221 | 2-hydroxydihydroisotanshinone I | Diterpenoids | P22894 | 0.111100851 | 4 |
| 222 | 2-hydroxydihydroisotanshinone I | Diterpenoids | P24385 P11802 | 0.105464394 | 4 |
| 223 | 2-hydroxydihydroisotanshinone I | Diterpenoids | P24941 P78396 P20248 | 0.108328679 | 5 |
| 224 | 2-hydroxydihydroisotanshinone I | Diterpenoids | P27815 | 0.106747907 | 3 |
| 225 | 2-hydroxydihydroisotanshinone I | Diterpenoids | P28472 P18507 P14867 | 0.103855577 | 16 |
| 226 | 2-hydroxydihydroisotanshinone I | Diterpenoids | P28472 P18507 P31644 | 0.103791839 | 19 |
| 227 | 2-hydroxydihydroisotanshinone I | Diterpenoids | P28472 P34903 P18507 | 0.103855577 | 16 |
| 228 | 2-hydroxydihydroisotanshinone I | Diterpenoids | P28845 | 0.253303956 | 34 |
| 229 | 2-hydroxydihydroisotanshinone I | Diterpenoids | P29275 | 0.103333221 | 16 |
| 230 | 2-hydroxydihydroisotanshinone I | Diterpenoids | P34972 | 0.103169308 | 13 |
| 231 | 2-hydroxydihydroisotanshinone I | Diterpenoids | P35354 | 0.113487837 | 23 |
| 232 | 2-hydroxydihydroisotanshinone I | Diterpenoids | P35968 | 0.105052994 | 26 |
| 233 | 2-hydroxydihydroisotanshinone I | Diterpenoids | P36888 | 0.102892289 | 8 |
| 234 | 2-hydroxydihydroisotanshinone I | Diterpenoids | P37059 | 0.106459925 | 6 |
| 235 | 2-hydroxydihydroisotanshinone I | Diterpenoids | P41594 | 0.103693674 | 22 |
| 236 | 2-hydroxydihydroisotanshinone I | Diterpenoids | P42338 | 0.107541648 | 9 |
| 237 | 2-hydroxydihydroisotanshinone I | Diterpenoids | P42345 | 0.10907142 | 6 |
| 238 | 2-hydroxydihydroisotanshinone I | Diterpenoids | P42574 | 0.10400953 | 13 |
| 239 | 2-hydroxydihydroisotanshinone I | Diterpenoids | P45452 | 0.106586322 | 6 |
| 240 | 2-hydroxydihydroisotanshinone I | Diterpenoids | P47869 P28472 P18507 | 0.104254314 | 15 |
| 241 | 2-hydroxydihydroisotanshinone I | Diterpenoids | P48039 | 0.101354935 | 19 |
| 242 | 2-hydroxydihydroisotanshinone I | Diterpenoids | P48147 | 0.107233003 | 22 |
| 243 | 2-hydroxydihydroisotanshinone I | Diterpenoids | P48736 | 0.108205862 | 3 |
| 244 | 2-hydroxydihydroisotanshinone I | Diterpenoids | P49286 | 0.101354935 | 19 |
| 245 | 2-hydroxydihydroisotanshinone I | Diterpenoids | P49841 | 0.105823985 | 11 |
| 246 | 2-hydroxydihydroisotanshinone I | Diterpenoids | P52333 | 0.104243408 | 12 |
| 247 | 2-hydroxydihydroisotanshinone I | Diterpenoids | P54760 | 0.111687884 | 3 |
| 248 | 2-hydroxydihydroisotanshinone I | Diterpenoids | P55210 | 0.10400953 | 13 |
| 249 | 2-hydroxydihydroisotanshinone I | Diterpenoids | P78527 | 0.104180881 | 6 |
| 250 | 2-hydroxydihydroisotanshinone I | Diterpenoids | P78536 | 0.104291862 | 9 |
| 251 | 2-hydroxydihydroisotanshinone I | Diterpenoids | Q02750 | 0.106463868 | 11 |
| 252 | 2-hydroxydihydroisotanshinone I | Diterpenoids | Q07343 | 0.107582642 | 6 |
| 253 | 2-hydroxydihydroisotanshinone I | Diterpenoids | Q08499 | 0.31881944 | 14 |
| 254 | 2-hydroxydihydroisotanshinone I | Diterpenoids | Q12884 | 0.102934441 | 6 |
| 255 | 2-hydroxydihydroisotanshinone I | Diterpenoids | Q13627 | 0.104383674 | 7 |
| 256 | 2-hydroxydihydroisotanshinone I | Diterpenoids | Q15078 Q00535 | 0.102910354 | 14 |
| 257 | 2-hydroxydihydroisotanshinone I | Diterpenoids | Q8WWL7 P06493 P14635 O95067 | 0.10231122 | 6 |
| 258 | 2-hydroxydihydroisotanshinone I | Diterpenoids | Q99572 | 0.10437669 | 25 |
| 259 | 2-hydroxydihydroisotanshinone I | Diterpenoids | Q99720 | 0.110060881 | 8 |
| 260 | 2-hydroxydihydroisotanshinone I | Diterpenoids | Q9H4B7 | 0.103451903 | 3 |
| 261 | 2-hydroxydihydroisotanshinone I | Diterpenoids | Q9UNQ0 | 0.106050933 | 5 |
| 262 | 2-Isopropyl-8-methylphenanthrene-3,4-dione (Ro 09-0680,miltirone I similar) | Diterpenoids | O00748 | 0.259386615 | 47 |
| 263 | 2-Isopropyl-8-methylphenanthrene-3,4-dione (Ro 09-0680,miltirone I similar) | Diterpenoids | O43194 | 0.10421989 | 3 |
| 264 | 2-Isopropyl-8-methylphenanthrene-3,4-dione (Ro 09-0680,miltirone I similar) | Diterpenoids | O43570 | 0.137972322 | 10 |
| 265 | 2-Isopropyl-8-methylphenanthrene-3,4-dione (Ro 09-0680,miltirone I similar) | Diterpenoids | O95271 | 0.102617882 | 7 |
| 266 | 2-Isopropyl-8-methylphenanthrene-3,4-dione (Ro 09-0680,miltirone I similar) | Diterpenoids | P00533 | 0.104151342 | 24 |
| 267 | 2-Isopropyl-8-methylphenanthrene-3,4-dione (Ro 09-0680,miltirone I similar) | Diterpenoids | P04626 | 0.104502793 | 11 |
| 268 | 2-Isopropyl-8-methylphenanthrene-3,4-dione (Ro 09-0680,miltirone I similar) | Diterpenoids | P05362 | 0.105155078 | 5 |
| 269 | 2-Isopropyl-8-methylphenanthrene-3,4-dione (Ro 09-0680,miltirone I similar) | Diterpenoids | P06276 | 0.130116287 | 14 |
| 270 | 2-Isopropyl-8-methylphenanthrene-3,4-dione (Ro 09-0680,miltirone I similar) | Diterpenoids | P07949 | 0.103471701 | 8 |
| 271 | 2-Isopropyl-8-methylphenanthrene-3,4-dione (Ro 09-0680,miltirone I similar) | Diterpenoids | P08172 | 0.117318329 | 18 |
| 272 | 2-Isopropyl-8-methylphenanthrene-3,4-dione (Ro 09-0680,miltirone I similar) | Diterpenoids | P08575 | 0.109148612 | 27 |
| 273 | 2-Isopropyl-8-methylphenanthrene-3,4-dione (Ro 09-0680,miltirone I similar) | Diterpenoids | P0DMS8 | 0.10464569 | 23 |
| 274 | 2-Isopropyl-8-methylphenanthrene-3,4-dione (Ro 09-0680,miltirone I similar) | Diterpenoids | P10586 | 0.312345915 | 15 |
| 275 | 2-Isopropyl-8-methylphenanthrene-3,4-dione (Ro 09-0680,miltirone I similar) | Diterpenoids | P11229 | 0.103603883 | 11 |
| 276 | 2-Isopropyl-8-methylphenanthrene-3,4-dione (Ro 09-0680,miltirone I similar) | Diterpenoids | P11387 | 0.113455556 | 13 |
| 277 | 2-Isopropyl-8-methylphenanthrene-3,4-dione (Ro 09-0680,miltirone I similar) | Diterpenoids | P14867 | 0.10300422 | 7 |
| 278 | 2-Isopropyl-8-methylphenanthrene-3,4-dione (Ro 09-0680,miltirone I similar) | Diterpenoids | P14902 | 0.110454469 | 24 |
| 279 | 2-Isopropyl-8-methylphenanthrene-3,4-dione (Ro 09-0680,miltirone I similar) | Diterpenoids | P15121 | 0.267155221 | 39 |
| 280 | 2-Isopropyl-8-methylphenanthrene-3,4-dione (Ro 09-0680,miltirone I similar) | Diterpenoids | P16581 | 0.105155078 | 5 |
| 281 | 2-Isopropyl-8-methylphenanthrene-3,4-dione (Ro 09-0680,miltirone I similar) | Diterpenoids | P17948 | 0.105554897 | 13 |
| 282 | 2-Isopropyl-8-methylphenanthrene-3,4-dione (Ro 09-0680,miltirone I similar) | Diterpenoids | P18031 | 0.474772974 | 23 |
| 283 | 2-Isopropyl-8-methylphenanthrene-3,4-dione (Ro 09-0680,miltirone I similar) | Diterpenoids | P19320 | 0.105155078 | 5 |
| 284 | 2-Isopropyl-8-methylphenanthrene-3,4-dione (Ro 09-0680,miltirone I similar) | Diterpenoids | P20309 | 0.105165619 | 10 |
| 285 | 2-Isopropyl-8-methylphenanthrene-3,4-dione (Ro 09-0680,miltirone I similar) | Diterpenoids | P21397 | 0.102816353 | 15 |
| 286 | 2-Isopropyl-8-methylphenanthrene-3,4-dione (Ro 09-0680,miltirone I similar) | Diterpenoids | P21980 | 0.101957459 | 8 |
| 287 | 2-Isopropyl-8-methylphenanthrene-3,4-dione (Ro 09-0680,miltirone I similar) | Diterpenoids | P22303 | 0.287353637 | 26 |
| 288 | 2-Isopropyl-8-methylphenanthrene-3,4-dione (Ro 09-0680,miltirone I similar) | Diterpenoids | P23141 | 0.275271523 | 31 |
| 289 | 2-Isopropyl-8-methylphenanthrene-3,4-dione (Ro 09-0680,miltirone I similar) | Diterpenoids | P24863 P49336 | 0.102633551 | 4 |
| 290 | 2-Isopropyl-8-methylphenanthrene-3,4-dione (Ro 09-0680,miltirone I similar) | Diterpenoids | P24864 P24941 | 0.102540734 | 7 |
| 291 | 2-Isopropyl-8-methylphenanthrene-3,4-dione (Ro 09-0680,miltirone I similar) | Diterpenoids | P27338 | 0.105021471 | 16 |
| 292 | 2-Isopropyl-8-methylphenanthrene-3,4-dione (Ro 09-0680,miltirone I similar) | Diterpenoids | P28472 P18507 P14867 | 0.103855577 | 16 |
| 293 | 2-Isopropyl-8-methylphenanthrene-3,4-dione (Ro 09-0680,miltirone I similar) | Diterpenoids | P28472 P18507 P31644 | 0.103791839 | 19 |
| 294 | 2-Isopropyl-8-methylphenanthrene-3,4-dione (Ro 09-0680,miltirone I similar) | Diterpenoids | P28472 P34903 P18507 | 0.103855577 | 16 |
| 295 | 2-Isopropyl-8-methylphenanthrene-3,4-dione (Ro 09-0680,miltirone I similar) | Diterpenoids | P28562 | 0.104003317 | 12 |
| 296 | 2-Isopropyl-8-methylphenanthrene-3,4-dione (Ro 09-0680,miltirone I similar) | Diterpenoids | P28845 | 0.253303956 | 34 |
| 297 | 2-Isopropyl-8-methylphenanthrene-3,4-dione (Ro 09-0680,miltirone I similar) | Diterpenoids | P29274 | 0.103202685 | 27 |
| 298 | 2-Isopropyl-8-methylphenanthrene-3,4-dione (Ro 09-0680,miltirone I similar) | Diterpenoids | P29350 | 0.204438393 | 44 |
| 299 | 2-Isopropyl-8-methylphenanthrene-3,4-dione (Ro 09-0680,miltirone I similar) | Diterpenoids | P30304 | 0.109145376 | 25 |
| 300 | 2-Isopropyl-8-methylphenanthrene-3,4-dione (Ro 09-0680,miltirone I similar) | Diterpenoids | P30305 | 0.185734372 | 40 |
| 301 | 2-Isopropyl-8-methylphenanthrene-3,4-dione (Ro 09-0680,miltirone I similar) | Diterpenoids | P30536 | 0.102095712 | 5 |
| 302 | 2-Isopropyl-8-methylphenanthrene-3,4-dione (Ro 09-0680,miltirone I similar) | Diterpenoids | P30542 | 0.101318267 | 24 |
| 303 | 2-Isopropyl-8-methylphenanthrene-3,4-dione (Ro 09-0680,miltirone I similar) | Diterpenoids | P31644 | 0.101037512 | 7 |
| 304 | 2-Isopropyl-8-methylphenanthrene-3,4-dione (Ro 09-0680,miltirone I similar) | Diterpenoids | P35557 | 0.102633551 | 4 |
| 305 | 2-Isopropyl-8-methylphenanthrene-3,4-dione (Ro 09-0680,miltirone I similar) | Diterpenoids | P35968 | 0.105052994 | 26 |
| 306 | 2-Isopropyl-8-methylphenanthrene-3,4-dione (Ro 09-0680,miltirone I similar) | Diterpenoids | P36888 | 0.102892289 | 8 |
| 307 | 2-Isopropyl-8-methylphenanthrene-3,4-dione (Ro 09-0680,miltirone I similar) | Diterpenoids | P41594 | 0.103693674 | 22 |
| 308 | 2-Isopropyl-8-methylphenanthrene-3,4-dione (Ro 09-0680,miltirone I similar) | Diterpenoids | P41595 | 0.102913571 | 7 |
| 309 | 2-Isopropyl-8-methylphenanthrene-3,4-dione (Ro 09-0680,miltirone I similar) | Diterpenoids | P42574 | 0.10400953 | 13 |
| 310 | 2-Isopropyl-8-methylphenanthrene-3,4-dione (Ro 09-0680,miltirone I similar) | Diterpenoids | P45983 | 0.105088123 | 17 |
| 311 | 2-Isopropyl-8-methylphenanthrene-3,4-dione (Ro 09-0680,miltirone I similar) | Diterpenoids | P46098 | 0.103859168 | 6 |
| 312 | 2-Isopropyl-8-methylphenanthrene-3,4-dione (Ro 09-0680,miltirone I similar) | Diterpenoids | P47869 P28472 P18507 | 0.104254314 | 15 |
| 313 | 2-Isopropyl-8-methylphenanthrene-3,4-dione (Ro 09-0680,miltirone I similar) | Diterpenoids | P55210 | 0.10400953 | 13 |
| 314 | 2-Isopropyl-8-methylphenanthrene-3,4-dione (Ro 09-0680,miltirone I similar) | Diterpenoids | P78536 | 0.104291862 | 9 |
| 315 | 2-Isopropyl-8-methylphenanthrene-3,4-dione (Ro 09-0680,miltirone I similar) | Diterpenoids | Q06124 | 0.211032909 | 40 |
| 316 | 2-Isopropyl-8-methylphenanthrene-3,4-dione (Ro 09-0680,miltirone I similar) | Diterpenoids | Q13255 | 0.103848909 | 9 |
| 317 | 2-Isopropyl-8-methylphenanthrene-3,4-dione (Ro 09-0680,miltirone I similar) | Diterpenoids | Q14833 | 0.102633551 | 4 |
| 318 | 2-Isopropyl-8-methylphenanthrene-3,4-dione (Ro 09-0680,miltirone I similar) | Diterpenoids | Q15078 Q00535 | 0.102910354 | 14 |
| 319 | 2-Isopropyl-8-methylphenanthrene-3,4-dione (Ro 09-0680,miltirone I similar) | Diterpenoids | Q16790 | 0.103433429 | 9 |
| 320 | 2-Isopropyl-8-methylphenanthrene-3,4-dione (Ro 09-0680,miltirone I similar) | Diterpenoids | Q7Z2W7 | 0.106776816 | 5 |
| 321 | 2-Isopropyl-8-methylphenanthrene-3,4-dione (Ro 09-0680,miltirone I similar) | Diterpenoids | Q9H2K2 | 0.104609573 | 11 |
| 322 | 2-Isopropyl-8-methylphenanthrene-3,4-dione (Ro 09-0680,miltirone I similar) | Diterpenoids | Q9Y2R2 | 0.111485907 | 5 |
| 323 | 2-Isopropyl-8-methylphenanthrene-3,4-dione (Ro 09-0680,miltirone I similar) | Diterpenoids | Q9Y5N1 | 0.101047212 | 6 |
| 324 | 3-hydroxytanshinone | Diterpenoids | O00311 | 0.105117399 | 6 |
| 325 | 3-hydroxytanshinone | Diterpenoids | O00329 | 0.105574288 | 6 |
| 326 | 3-hydroxytanshinone | Diterpenoids | O00408 | 0.103882897 | 9 |
| 327 | 3-hydroxytanshinone | Diterpenoids | O00748 | 0.259386615 | 47 |
| 328 | 3-hydroxytanshinone | Diterpenoids | O14649 | 0.100520388 | 4 |
| 329 | 3-hydroxytanshinone | Diterpenoids | O14746 | 0.14728518 | 27 |
| 330 | 3-hydroxytanshinone | Diterpenoids | O14757 | 0.106739614 | 8 |
| 331 | 3-hydroxytanshinone | Diterpenoids | O14965 | 0.107316885 | 8 |
| 332 | 3-hydroxytanshinone | Diterpenoids | O43570 | 0.137972322 | 10 |
| 333 | 3-hydroxytanshinone | Diterpenoids | O43613 | 0.10159278 | 9 |
| 334 | 3-hydroxytanshinone | Diterpenoids | O43614 | 0.100916735 | 11 |
| 335 | 3-hydroxytanshinone | Diterpenoids | O60674 | 0.103305945 | 11 |
| 336 | 3-hydroxytanshinone | Diterpenoids | O60725 | 0.108205862 | 3 |
| 337 | 3-hydroxytanshinone | Diterpenoids | O75530 Q15022 Q15910 | 0.341964133 | 19 |
| 338 | 3-hydroxytanshinone | Diterpenoids | O76074 | 0.103099774 | 12 |
| 339 | 3-hydroxytanshinone | Diterpenoids | P00533 | 0.104151342 | 24 |
| 340 | 3-hydroxytanshinone | Diterpenoids | P00746 | 0.101536397 | 5 |
| 341 | 3-hydroxytanshinone | Diterpenoids | P00918 | 0.141292356 | 8 |
| 342 | 3-hydroxytanshinone | Diterpenoids | P03956 | 0.109842942 | 8 |
| 343 | 3-hydroxytanshinone | Diterpenoids | P04035 | 0.166864971 | 20 |
| 344 | 3-hydroxytanshinone | Diterpenoids | P04150 | 0.109500234 | 17 |
| 345 | 3-hydroxytanshinone | Diterpenoids | P05186 | 0.104336352 | 9 |
| 346 | 3-hydroxytanshinone | Diterpenoids | P05362 | 0.105155078 | 5 |
| 347 | 3-hydroxytanshinone | Diterpenoids | P06493 | 0.102732601 | 7 |
| 348 | 3-hydroxytanshinone | Diterpenoids | P06737 | 0.103331553 | 10 |
| 349 | 3-hydroxytanshinone | Diterpenoids | P08183 | 0.104694286 | 5 |
| 350 | 3-hydroxytanshinone | Diterpenoids | P08575 | 0.109148612 | 27 |
| 351 | 3-hydroxytanshinone | Diterpenoids | P08684 | 0.104909858 | 3 |
| 352 | 3-hydroxytanshinone | Diterpenoids | P09211 | 0.100367415 | 3 |
| 353 | 3-hydroxytanshinone | Diterpenoids | P09601 | 0.103663418 | 3 |
| 354 | 3-hydroxytanshinone | Diterpenoids | P0DMS8 | 0.10464569 | 23 |
| 355 | 3-hydroxytanshinone | Diterpenoids | P10275 | 0.132131799 | 34 |
| 356 | 3-hydroxytanshinone | Diterpenoids | P11309 | 0.104805981 | 13 |
| 357 | 3-hydroxytanshinone | Diterpenoids | P12268 | 0.104524224 | 13 |
| 358 | 3-hydroxytanshinone | Diterpenoids | P15121 | 0.267155221 | 39 |
| 359 | 3-hydroxytanshinone | Diterpenoids | P16083 | 0.112425971 | 14 |
| 360 | 3-hydroxytanshinone | Diterpenoids | P16581 | 0.105155078 | 5 |
| 361 | 3-hydroxytanshinone | Diterpenoids | P17252 | 0.102754312 | 7 |
| 362 | 3-hydroxytanshinone | Diterpenoids | P19320 | 0.105155078 | 5 |
| 363 | 3-hydroxytanshinone | Diterpenoids | P21554 | 0.104626059 | 10 |
| 364 | 3-hydroxytanshinone | Diterpenoids | P23141 | 0.275271523 | 31 |
| 365 | 3-hydroxytanshinone | Diterpenoids | P23219 | 0.109524644 | 16 |
| 366 | 3-hydroxytanshinone | Diterpenoids | P23458 | 0.102916912 | 11 |
| 367 | 3-hydroxytanshinone | Diterpenoids | P25101 | 0.104008389 | 6 |
| 368 | 3-hydroxytanshinone | Diterpenoids | P28221 | 0.104909858 | 3 |
| 369 | 3-hydroxytanshinone | Diterpenoids | P28472 P18507 P14867 | 0.103855577 | 16 |
| 370 | 3-hydroxytanshinone | Diterpenoids | P28472 P18507 P31644 | 0.103791839 | 19 |
| 371 | 3-hydroxytanshinone | Diterpenoids | P28472 P34903 P18507 | 0.103855577 | 16 |
| 372 | 3-hydroxytanshinone | Diterpenoids | P28845 | 0.253303956 | 34 |
| 373 | 3-hydroxytanshinone | Diterpenoids | P29274 | 0.103202685 | 27 |
| 374 | 3-hydroxytanshinone | Diterpenoids | P29275 | 0.103333221 | 16 |
| 375 | 3-hydroxytanshinone | Diterpenoids | P29350 | 0.204438393 | 44 |
| 376 | 3-hydroxytanshinone | Diterpenoids | P30304 | 0.109145376 | 25 |
| 377 | 3-hydroxytanshinone | Diterpenoids | P30305 | 0.185734372 | 40 |
| 378 | 3-hydroxytanshinone | Diterpenoids | P30307 | 0.105546712 | 12 |
| 379 | 3-hydroxytanshinone | Diterpenoids | P30542 | 0.101318267 | 24 |
| 380 | 3-hydroxytanshinone | Diterpenoids | P32246 | 0.109647095 | 6 |
| 381 | 3-hydroxytanshinone | Diterpenoids | P33261 | 0.124903734 | 16 |
| 382 | 3-hydroxytanshinone | Diterpenoids | P34972 | 0.103169308 | 13 |
| 383 | 3-hydroxytanshinone | Diterpenoids | P35968 | 0.105052994 | 26 |
| 384 | 3-hydroxytanshinone | Diterpenoids | P41145 | 0.107282863 | 6 |
| 385 | 3-hydroxytanshinone | Diterpenoids | P41594 | 0.103693674 | 22 |
| 386 | 3-hydroxytanshinone | Diterpenoids | P42338 | 0.107541648 | 9 |
| 387 | 3-hydroxytanshinone | Diterpenoids | P42345 | 0.10907142 | 6 |
| 388 | 3-hydroxytanshinone | Diterpenoids | P47869 P28472 P18507 | 0.104254314 | 15 |
| 389 | 3-hydroxytanshinone | Diterpenoids | P48039 | 0.101354935 | 19 |
| 390 | 3-hydroxytanshinone | Diterpenoids | P48736 | 0.108205862 | 3 |
| 391 | 3-hydroxytanshinone | Diterpenoids | P49137 | 0.107101597 | 6 |
| 392 | 3-hydroxytanshinone | Diterpenoids | P49286 | 0.101354935 | 19 |
| 393 | 3-hydroxytanshinone | Diterpenoids | P49354 P49356 | 0.109904857 | 24 |
| 394 | 3-hydroxytanshinone | Diterpenoids | P49841 | 0.105823985 | 11 |
| 395 | 3-hydroxytanshinone | Diterpenoids | P50406 | 0.106847218 | 8 |
| 396 | 3-hydroxytanshinone | Diterpenoids | P52732 | 0.104747724 | 9 |
| 397 | 3-hydroxytanshinone | Diterpenoids | P53609 P49354 | 0.101613855 | 3 |
| 398 | 3-hydroxytanshinone | Diterpenoids | P56373 | 0.107098941 | 5 |
| 399 | 3-hydroxytanshinone | Diterpenoids | P56817 | 0.108325485 | 12 |
| 400 | 3-hydroxytanshinone | Diterpenoids | P78527 | 0.104180881 | 6 |
| 401 | 3-hydroxytanshinone | Diterpenoids | Q00987 | 0.107095673 | 11 |
| 402 | 3-hydroxytanshinone | Diterpenoids | Q02750 | 0.106463868 | 11 |
| 403 | 3-hydroxytanshinone | Diterpenoids | Q05655 | 0.102598878 | 5 |
| 404 | 3-hydroxytanshinone | Diterpenoids | Q06124 | 0.211032909 | 40 |
| 405 | 3-hydroxytanshinone | Diterpenoids | Q07343 | 0.107582642 | 6 |
| 406 | 3-hydroxytanshinone | Diterpenoids | Q08828 | 0.102451863 | 4 |
| 407 | 3-hydroxytanshinone | Diterpenoids | Q09028 Q16576 O75530 Q15022 Q15910 | 0.142197972 | 14 |
| 408 | 3-hydroxytanshinone | Diterpenoids | Q13946 | 0.104692547 | 7 |
| 409 | 3-hydroxytanshinone | Diterpenoids | Q15078 Q00535 | 0.102910354 | 14 |
| 410 | 3-hydroxytanshinone | Diterpenoids | Q16539 | 0.105438848 | 20 |
| 411 | 3-hydroxytanshinone | Diterpenoids | Q16790 | 0.103433429 | 9 |
| 412 | 3-hydroxytanshinone | Diterpenoids | Q86V86 | 0.100967138 | 7 |
| 413 | 3-hydroxytanshinone | Diterpenoids | Q99572 | 0.10437669 | 25 |
| 414 | 3-hydroxytanshinone | Diterpenoids | Q9NWZ3 | 0.105136637 | 5 |
| 415 | 3-hydroxytanshinone | Diterpenoids | Q9P1W9 | 0.103344117 | 12 |
| 416 | 3-hydroxytanshinone | Diterpenoids | Q9Y233 | 0.108085897 | 14 |
| 417 | cryptotanshinone | Diterpenoids | B2RXH2 | 0.104871629 | 12 |
| 418 | cryptotanshinone | Diterpenoids | O00748 | 0.259386615 | 47 |
| 419 | cryptotanshinone | Diterpenoids | O95551 | 0.106334502 | 10 |
| 420 | cryptotanshinone | Diterpenoids | P05093 | 0.148009819 | 20 |
| 421 | cryptotanshinone | Diterpenoids | P05362 | 0.105155078 | 5 |
| 422 | cryptotanshinone | Diterpenoids | P07333 | 0.104063456 | 9 |
| 423 | cryptotanshinone | Diterpenoids | P08172 | 0.117318329 | 18 |
| 424 | cryptotanshinone | Diterpenoids | P08575 | 0.109148612 | 27 |
| 425 | cryptotanshinone | Diterpenoids | P09874 | 0.106818993 | 18 |
| 426 | cryptotanshinone | Diterpenoids | P0DMS8 | 0.10464569 | 23 |
| 427 | cryptotanshinone | Diterpenoids | P11229 | 0.103603883 | 11 |
| 428 | cryptotanshinone | Diterpenoids | P11387 | 0.113455556 | 13 |
| 429 | cryptotanshinone | Diterpenoids | P11388 | 0.106757964 | 8 |
| 430 | cryptotanshinone | Diterpenoids | P11511 | 0.139056613 | 29 |
| 431 | cryptotanshinone | Diterpenoids | P14902 | 0.110454469 | 24 |
| 432 | cryptotanshinone | Diterpenoids | P15121 | 0.267155221 | 39 |
| 433 | cryptotanshinone | Diterpenoids | P16581 | 0.105155078 | 5 |
| 434 | cryptotanshinone | Diterpenoids | P18031 | 0.474772974 | 23 |
| 435 | cryptotanshinone | Diterpenoids | P20309 | 0.105165619 | 10 |
| 436 | cryptotanshinone | Diterpenoids | P21980 | 0.101957459 | 8 |
| 437 | cryptotanshinone | Diterpenoids | P22303 | 0.287353637 | 26 |
| 438 | cryptotanshinone | Diterpenoids | P23141 | 0.275271523 | 31 |
| 439 | cryptotanshinone | Diterpenoids | P23219 | 0.109524644 | 16 |
| 440 | cryptotanshinone | Diterpenoids | P28223 | 0.103919859 | 7 |
| 441 | cryptotanshinone | Diterpenoids | P28335 | 0.104521595 | 9 |
| 442 | cryptotanshinone | Diterpenoids | P28562 | 0.104003317 | 12 |
| 443 | cryptotanshinone | Diterpenoids | P28845 | 0.253303956 | 34 |
| 444 | cryptotanshinone | Diterpenoids | P29274 | 0.103202685 | 27 |
| 445 | cryptotanshinone | Diterpenoids | P29275 | 0.103333221 | 16 |
| 446 | cryptotanshinone | Diterpenoids | P29350 | 0.204438393 | 44 |
| 447 | cryptotanshinone | Diterpenoids | P29597 | 0.106959422 | 3 |
| 448 | cryptotanshinone | Diterpenoids | P30305 | 0.185734372 | 40 |
| 449 | cryptotanshinone | Diterpenoids | P30542 | 0.101318267 | 24 |
| 450 | cryptotanshinone | Diterpenoids | P34913 | 0.106959422 | 6 |
| 451 | cryptotanshinone | Diterpenoids | P34972 | 0.103169308 | 13 |
| 452 | cryptotanshinone | Diterpenoids | P35354 | 0.113487837 | 23 |
| 453 | cryptotanshinone | Diterpenoids | P35968 | 0.105052994 | 26 |
| 454 | cryptotanshinone | Diterpenoids | P40763 | 0.268996335 | 13 |
| 455 | cryptotanshinone | Diterpenoids | P41594 | 0.103693674 | 22 |
| 456 | cryptotanshinone | Diterpenoids | P42330 | 0.105243819 | 5 |
| 457 | cryptotanshinone | Diterpenoids | P42336 | 0.108042586 | 13 |
| 458 | cryptotanshinone | Diterpenoids | P52732 | 0.104747724 | 9 |
| 459 | cryptotanshinone | Diterpenoids | P53779 | 0.103177433 | 9 |
| 460 | cryptotanshinone | Diterpenoids | Q02083 | 0.102416978 | 6 |
| 461 | cryptotanshinone | Diterpenoids | Q05397 | 0.104909858 | 3 |
| 462 | cryptotanshinone | Diterpenoids | Q06124 | 0.211032909 | 40 |
| 463 | cryptotanshinone | Diterpenoids | Q13255 | 0.103848909 | 9 |
| 464 | cryptotanshinone | Diterpenoids | Q15761 | 0.10562303 | 4 |
| 465 | cryptotanshinone | Diterpenoids | Q15858 | 0.101761829 | 5 |
| 466 | cryptotanshinone | Diterpenoids | Q9H2K2 | 0.104609573 | 11 |
| 467 | cryptotanshinone | Diterpenoids | Q9H3R0 | 0.106959422 | 3 |
| 468 | cryptotanshinone | Diterpenoids | Q9UDY8 | 0.107158923 | 10 |
| 469 | danshenol a | Diterpenoids | B2RXH2 | 0.104871629 | 12 |
| 470 | danshenol a | Diterpenoids | O00408 | 0.103882897 | 9 |
| 471 | danshenol a | Diterpenoids | O00748 | 0.259386615 | 47 |
| 472 | danshenol a | Diterpenoids | O14684 | 0.116389783 | 19 |
| 473 | danshenol a | Diterpenoids | O14746 | 0.14728518 | 27 |
| 474 | danshenol a | Diterpenoids | O14965 | 0.107316885 | 8 |
| 475 | danshenol a | Diterpenoids | O43613 | 0.10159278 | 9 |
| 476 | danshenol a | Diterpenoids | O43614 | 0.100916735 | 11 |
| 477 | danshenol a | Diterpenoids | O60674 | 0.103305945 | 11 |
| 478 | danshenol a | Diterpenoids | O60706 | 0.103819513 | 5 |
| 479 | danshenol a | Diterpenoids | P00915 | 0.106312298 | 8 |
| 480 | danshenol a | Diterpenoids | P00918 | 0.141292356 | 8 |
| 481 | danshenol a | Diterpenoids | P04150 | 0.109500234 | 17 |
| 482 | danshenol a | Diterpenoids | P05093 | 0.148009819 | 20 |
| 483 | danshenol a | Diterpenoids | P06401 | 0.108868706 | 15 |
| 484 | danshenol a | Diterpenoids | P06493 | 0.102732601 | 7 |
| 485 | danshenol a | Diterpenoids | P06737 | 0.103331553 | 10 |
| 486 | danshenol a | Diterpenoids | P07384 | 0.10807591 | 4 |
| 487 | danshenol a | Diterpenoids | P08172 | 0.117318329 | 18 |
| 488 | danshenol a | Diterpenoids | P08183 | 0.104694286 | 5 |
| 489 | danshenol a | Diterpenoids | P08235 | 0.107998327 | 12 |
| 490 | danshenol a | Diterpenoids | P08254 | 0.109296209 | 8 |
| 491 | danshenol a | Diterpenoids | P09874 | 0.106818993 | 18 |
| 492 | danshenol a | Diterpenoids | P09917 | 0.106782208 | 14 |
| 493 | danshenol a | Diterpenoids | P0DMS8 | 0.10464569 | 23 |
| 494 | danshenol a | Diterpenoids | P10275 | 0.132131799 | 34 |
| 495 | danshenol a | Diterpenoids | P11229 | 0.103603883 | 11 |
| 496 | danshenol a | Diterpenoids | P11309 | 0.104805981 | 13 |
| 497 | danshenol a | Diterpenoids | P14902 | 0.110454469 | 24 |
| 498 | danshenol a | Diterpenoids | P15056 | 0.101798823 | 4 |
| 499 | danshenol a | Diterpenoids | P15121 | 0.267155221 | 39 |
| 500 | danshenol a | Diterpenoids | P18054 | 0.103847438 | 5 |
| 501 | danshenol a | Diterpenoids | P20309 | 0.105165619 | 10 |
| 502 | danshenol a | Diterpenoids | P22303 | 0.287353637 | 26 |
| 503 | danshenol a | Diterpenoids | P22748 | 0.219188074 | 3 |
| 504 | danshenol a | Diterpenoids | P23141 | 0.275271523 | 31 |
| 505 | danshenol a | Diterpenoids | P23458 | 0.102916912 | 11 |
| 506 | danshenol a | Diterpenoids | P23975 | 0.115012308 | 10 |
| 507 | danshenol a | Diterpenoids | P24941 | 0.106080884 | 8 |
| 508 | danshenol a | Diterpenoids | P25025 | 0.100320282 | 13 |
| 509 | danshenol a | Diterpenoids | P25101 | 0.104008389 | 6 |
| 510 | danshenol a | Diterpenoids | P28223 | 0.103919859 | 7 |
| 511 | danshenol a | Diterpenoids | P28845 | 0.253303956 | 34 |
| 512 | danshenol a | Diterpenoids | P29274 | 0.103202685 | 27 |
| 513 | danshenol a | Diterpenoids | P29275 | 0.103333221 | 16 |
| 514 | danshenol a | Diterpenoids | P29350 | 0.204438393 | 44 |
| 515 | danshenol a | Diterpenoids | P29466 | 0.105464394 | 4 |
| 516 | danshenol a | Diterpenoids | P30542 | 0.101318267 | 24 |
| 517 | danshenol a | Diterpenoids | P31645 | 0.1201852 | 17 |
| 518 | danshenol a | Diterpenoids | P32247 | 0.103071649 | 5 |
| 519 | danshenol a | Diterpenoids | P34969 | 0.102205463 | 3 |
| 520 | danshenol a | Diterpenoids | P35968 | 0.105052994 | 26 |
| 521 | danshenol a | Diterpenoids | P37059 | 0.106459925 | 6 |
| 522 | danshenol a | Diterpenoids | P40763 | 0.268996335 | 13 |
| 523 | danshenol a | Diterpenoids | P41145 | 0.107282863 | 6 |
| 524 | danshenol a | Diterpenoids | P41180 | 0.104664572 | 4 |
| 525 | danshenol a | Diterpenoids | P41594 | 0.103693674 | 22 |
| 526 | danshenol a | Diterpenoids | P42574 | 0.10400953 | 13 |
| 527 | danshenol a | Diterpenoids | P45452 | 0.106586322 | 6 |
| 528 | danshenol a | Diterpenoids | P45983 | 0.105088123 | 17 |
| 529 | danshenol a | Diterpenoids | P49354 P49356 | 0.109904857 | 24 |
| 530 | danshenol a | Diterpenoids | P49810 Q9NZ42 Q92542 Q96BI3 P49768 Q8WW43 | 0.101543467 | 9 |
| 531 | danshenol a | Diterpenoids | P52333 | 0.104243408 | 12 |
| 532 | danshenol a | Diterpenoids | P55210 | 0.10400953 | 13 |
| 533 | danshenol a | Diterpenoids | Q01432 | 0.101993948 | 3 |
| 534 | danshenol a | Diterpenoids | Q01959 | 0.107731401 | 12 |
| 535 | danshenol a | Diterpenoids | Q06124 | 0.211032909 | 40 |
| 536 | danshenol a | Diterpenoids | Q07820 | 0.101122731 | 4 |
| 537 | danshenol a | Diterpenoids | Q14790 | 0.104694286 | 5 |
| 538 | danshenol a | Diterpenoids | Q15858 | 0.101761829 | 5 |
| 539 | danshenol a | Diterpenoids | Q16539 | 0.105438848 | 20 |
| 540 | danshenol a | Diterpenoids | Q5S007 | 0.102368749 | 9 |
| 541 | danshenol a | Diterpenoids | Q8WWL7 P06493 P14635 O95067 | 0.10231122 | 6 |
| 542 | danshenol a | Diterpenoids | Q9NZJ5 | 0.102919059 | 6 |
| 543 | danshenol a | Diterpenoids | Q9Y233 | 0.108085897 | 14 |
| 544 | danshenxinkun B(tanshiquinone B,neotanshinone B) | Diterpenoids | B2RXH2 | 0.104871629 | 12 |
| 545 | danshenxinkun B(tanshiquinone B,neotanshinone B) | Diterpenoids | O43613 | 0.10159278 | 9 |
| 546 | danshenxinkun B(tanshiquinone B,neotanshinone B) | Diterpenoids | O43614 | 0.100916735 | 11 |
| 547 | danshenxinkun B(tanshiquinone B,neotanshinone B) | Diterpenoids | O75874 | 0.1046882 | 6 |
| 548 | danshenxinkun B(tanshiquinone B,neotanshinone B) | Diterpenoids | O76074 | 0.103099774 | 12 |
| 549 | danshenxinkun B(tanshiquinone B,neotanshinone B) | Diterpenoids | O95271 | 0.102617882 | 7 |
| 550 | danshenxinkun B(tanshiquinone B,neotanshinone B) | Diterpenoids | P00338 | 0.106699814 | 5 |
| 551 | danshenxinkun B(tanshiquinone B,neotanshinone B) | Diterpenoids | P04818 | 0.111100851 | 4 |
| 552 | danshenxinkun B(tanshiquinone B,neotanshinone B) | Diterpenoids | P06213 | 0.103151027 | 4 |
| 553 | danshenxinkun B(tanshiquinone B,neotanshinone B) | Diterpenoids | P08235 | 0.107998327 | 12 |
| 554 | danshenxinkun B(tanshiquinone B,neotanshinone B) | Diterpenoids | P08253 | 0.106581489 | 9 |
| 555 | danshenxinkun B(tanshiquinone B,neotanshinone B) | Diterpenoids | P09874 | 0.106818993 | 18 |
| 556 | danshenxinkun B(tanshiquinone B,neotanshinone B) | Diterpenoids | P0DMS8 | 0.10464569 | 23 |
| 557 | danshenxinkun B(tanshiquinone B,neotanshinone B) | Diterpenoids | P11229 | 0.103603883 | 11 |
| 558 | danshenxinkun B(tanshiquinone B,neotanshinone B) | Diterpenoids | P14780 | 0.106689076 | 6 |
| 559 | danshenxinkun B(tanshiquinone B,neotanshinone B) | Diterpenoids | P14867 | 0.10300422 | 7 |
| 560 | danshenxinkun B(tanshiquinone B,neotanshinone B) | Diterpenoids | P14902 | 0.110454469 | 24 |
| 561 | danshenxinkun B(tanshiquinone B,neotanshinone B) | Diterpenoids | P15121 | 0.267155221 | 39 |
| 562 | danshenxinkun B(tanshiquinone B,neotanshinone B) | Diterpenoids | P16050 | 0.105228934 | 6 |
| 563 | danshenxinkun B(tanshiquinone B,neotanshinone B) | Diterpenoids | P16083 | 0.112425971 | 14 |
| 564 | danshenxinkun B(tanshiquinone B,neotanshinone B) | Diterpenoids | P18054 | 0.103847438 | 5 |
| 565 | danshenxinkun B(tanshiquinone B,neotanshinone B) | Diterpenoids | P20309 | 0.105165619 | 10 |
| 566 | danshenxinkun B(tanshiquinone B,neotanshinone B) | Diterpenoids | P21397 | 0.102816353 | 15 |
| 567 | danshenxinkun B(tanshiquinone B,neotanshinone B) | Diterpenoids | P21554 | 0.104626059 | 10 |
| 568 | danshenxinkun B(tanshiquinone B,neotanshinone B) | Diterpenoids | P27338 | 0.105021471 | 16 |
| 569 | danshenxinkun B(tanshiquinone B,neotanshinone B) | Diterpenoids | P29274 | 0.103202685 | 27 |
| 570 | danshenxinkun B(tanshiquinone B,neotanshinone B) | Diterpenoids | P29371 | 0.103946422 | 5 |
| 571 | danshenxinkun B(tanshiquinone B,neotanshinone B) | Diterpenoids | P30305 | 0.185734372 | 40 |
| 572 | danshenxinkun B(tanshiquinone B,neotanshinone B) | Diterpenoids | P30542 | 0.101318267 | 24 |
| 573 | danshenxinkun B(tanshiquinone B,neotanshinone B) | Diterpenoids | P34972 | 0.103169308 | 13 |
| 574 | danshenxinkun B(tanshiquinone B,neotanshinone B) | Diterpenoids | P35968 | 0.105052994 | 26 |
| 575 | danshenxinkun B(tanshiquinone B,neotanshinone B) | Diterpenoids | P36888 | 0.102892289 | 8 |
| 576 | danshenxinkun B(tanshiquinone B,neotanshinone B) | Diterpenoids | P37059 | 0.106459925 | 6 |
| 577 | danshenxinkun B(tanshiquinone B,neotanshinone B) | Diterpenoids | P37231 | 0.111742909 | 15 |
| 578 | danshenxinkun B(tanshiquinone B,neotanshinone B) | Diterpenoids | P41180 | 0.104664572 | 4 |
| 579 | danshenxinkun B(tanshiquinone B,neotanshinone B) | Diterpenoids | Q00987 | 0.107095673 | 11 |
| 580 | danshenxinkun B(tanshiquinone B,neotanshinone B) | Diterpenoids | Q02127 | 0.106699814 | 5 |
| 581 | danshenxinkun B(tanshiquinone B,neotanshinone B) | Diterpenoids | Q12791 | 0.103498446 | 3 |
| 582 | danshenxinkun B(tanshiquinone B,neotanshinone B) | Diterpenoids | Q15078 Q00535 | 0.102910354 | 14 |
| 583 | danshenxinkun B(tanshiquinone B,neotanshinone B) | Diterpenoids | Q15746 | 0.102744609 | 5 |
| 584 | danshenxinkun B(tanshiquinone B,neotanshinone B) | Diterpenoids | Q8WWL7 P06493 P14635 O95067 | 0.10231122 | 6 |
| 585 | danshenxinkun B(tanshiquinone B,neotanshinone B) | Diterpenoids | Q92731 | 0.117586956 | 23 |
| 586 | danshenxinkun B(tanshiquinone B,neotanshinone B) | Diterpenoids | Q99683 | 0.104664572 | 4 |
| 587 | danshenxinkun B(tanshiquinone B,neotanshinone B) | Diterpenoids | Q9H2K2 | 0.104609573 | 11 |
| 588 | danshenxinkun B(tanshiquinone B,neotanshinone B) | Diterpenoids | Q9H3R0 | 0.106959422 | 3 |
| 589 | danshenxinkun B(tanshiquinone B,neotanshinone B) | Diterpenoids | Q9HC97 | 0.107139434 | 3 |
| 590 | danshenxinkun B(tanshiquinone B,neotanshinone B) | Diterpenoids | Q9UNQ0 | 0.106050933 | 5 |
| 591 | danshenxinkun B(tanshiquinone B,neotanshinone B) | Diterpenoids | Q9Y2R2 | 0.111485907 | 5 |
| 592 | danshenxinkun B(tanshiquinone B,neotanshinone B) | Diterpenoids | Q9Y5Z0 | 0.106959422 | 3 |
| 593 | danshenxinkun C (Neotanshinone C,tanshiquinone C) | Diterpenoids | O00408 | 0.103882897 | 9 |
| 594 | danshenxinkun C (Neotanshinone C,tanshiquinone C) | Diterpenoids | O14746 | 0.14728518 | 27 |
| 595 | danshenxinkun C (Neotanshinone C,tanshiquinone C) | Diterpenoids | O60427 | 0.10804089 | 3 |
| 596 | danshenxinkun C (Neotanshinone C,tanshiquinone C) | Diterpenoids | O60674 | 0.103305945 | 11 |
| 597 | danshenxinkun C (Neotanshinone C,tanshiquinone C) | Diterpenoids | O75460 | 0.105470076 | 5 |
| 598 | danshenxinkun C (Neotanshinone C,tanshiquinone C) | Diterpenoids | O76074 | 0.103099774 | 12 |
| 599 | danshenxinkun C (Neotanshinone C,tanshiquinone C) | Diterpenoids | O95271 | 0.102617882 | 7 |
| 600 | danshenxinkun C (Neotanshinone C,tanshiquinone C) | Diterpenoids | P00338 | 0.106699814 | 5 |
| 601 | danshenxinkun C (Neotanshinone C,tanshiquinone C) | Diterpenoids | P00533 | 0.104151342 | 24 |
| 602 | danshenxinkun C (Neotanshinone C,tanshiquinone C) | Diterpenoids | P04278 | 0.16719946 | 19 |
| 603 | danshenxinkun C (Neotanshinone C,tanshiquinone C) | Diterpenoids | P05093 | 0.148009819 | 20 |
| 604 | danshenxinkun C (Neotanshinone C,tanshiquinone C) | Diterpenoids | P08253 | 0.106581489 | 9 |
| 605 | danshenxinkun C (Neotanshinone C,tanshiquinone C) | Diterpenoids | P09874 | 0.106818993 | 18 |
| 606 | danshenxinkun C (Neotanshinone C,tanshiquinone C) | Diterpenoids | P0DMS8 | 0.10464569 | 23 |
| 607 | danshenxinkun C (Neotanshinone C,tanshiquinone C) | Diterpenoids | P14416 | 0.108887011 | 4 |
| 608 | danshenxinkun C (Neotanshinone C,tanshiquinone C) | Diterpenoids | P14780 | 0.106689076 | 6 |
| 609 | danshenxinkun C (Neotanshinone C,tanshiquinone C) | Diterpenoids | P14867 | 0.10300422 | 7 |
| 610 | danshenxinkun C (Neotanshinone C,tanshiquinone C) | Diterpenoids | P14902 | 0.110454469 | 24 |
| 611 | danshenxinkun C (Neotanshinone C,tanshiquinone C) | Diterpenoids | P15121 | 0.267155221 | 39 |
| 612 | danshenxinkun C (Neotanshinone C,tanshiquinone C) | Diterpenoids | P15538 | 0.10615894 | 5 |
| 613 | danshenxinkun C (Neotanshinone C,tanshiquinone C) | Diterpenoids | P16050 | 0.105228934 | 6 |
| 614 | danshenxinkun C (Neotanshinone C,tanshiquinone C) | Diterpenoids | P18054 | 0.103847438 | 5 |
| 615 | danshenxinkun C (Neotanshinone C,tanshiquinone C) | Diterpenoids | P19099 | 0.10615894 | 5 |
| 616 | danshenxinkun C (Neotanshinone C,tanshiquinone C) | Diterpenoids | P21397 | 0.102816353 | 15 |
| 617 | danshenxinkun C (Neotanshinone C,tanshiquinone C) | Diterpenoids | P21728 | 0.105764248 | 3 |
| 618 | danshenxinkun C (Neotanshinone C,tanshiquinone C) | Diterpenoids | P27338 | 0.105021471 | 16 |
| 619 | danshenxinkun C (Neotanshinone C,tanshiquinone C) | Diterpenoids | P28335 | 0.104521595 | 9 |
| 620 | danshenxinkun C (Neotanshinone C,tanshiquinone C) | Diterpenoids | P28472 P18507 P14867 | 0.103855577 | 16 |
| 621 | danshenxinkun C (Neotanshinone C,tanshiquinone C) | Diterpenoids | P28472 P18507 P31644 | 0.103791839 | 19 |
| 622 | danshenxinkun C (Neotanshinone C,tanshiquinone C) | Diterpenoids | P28472 P34903 P18507 | 0.103855577 | 16 |
| 623 | danshenxinkun C (Neotanshinone C,tanshiquinone C) | Diterpenoids | P28845 | 0.253303956 | 34 |
| 624 | danshenxinkun C (Neotanshinone C,tanshiquinone C) | Diterpenoids | P29274 | 0.103202685 | 27 |
| 625 | danshenxinkun C (Neotanshinone C,tanshiquinone C) | Diterpenoids | P30305 | 0.185734372 | 40 |
| 626 | danshenxinkun C (Neotanshinone C,tanshiquinone C) | Diterpenoids | P30542 | 0.101318267 | 24 |
| 627 | danshenxinkun C (Neotanshinone C,tanshiquinone C) | Diterpenoids | P35354 | 0.113487837 | 23 |
| 628 | danshenxinkun C (Neotanshinone C,tanshiquinone C) | Diterpenoids | P35462 | 0.105764248 | 3 |
| 629 | danshenxinkun C (Neotanshinone C,tanshiquinone C) | Diterpenoids | P37231 | 0.111742909 | 15 |
| 630 | danshenxinkun C (Neotanshinone C,tanshiquinone C) | Diterpenoids | P41180 | 0.104664572 | 4 |
| 631 | danshenxinkun C (Neotanshinone C,tanshiquinone C) | Diterpenoids | P41594 | 0.103693674 | 22 |
| 632 | danshenxinkun C (Neotanshinone C,tanshiquinone C) | Diterpenoids | P41595 | 0.102913571 | 7 |
| 633 | danshenxinkun C (Neotanshinone C,tanshiquinone C) | Diterpenoids | P42330 | 0.105243819 | 5 |
| 634 | danshenxinkun C (Neotanshinone C,tanshiquinone C) | Diterpenoids | P43166 | 0.165991461 | 6 |
| 635 | danshenxinkun C (Neotanshinone C,tanshiquinone C) | Diterpenoids | P45983 | 0.105088123 | 17 |
| 636 | danshenxinkun C (Neotanshinone C,tanshiquinone C) | Diterpenoids | P46098 | 0.103859168 | 6 |
| 637 | danshenxinkun C (Neotanshinone C,tanshiquinone C) | Diterpenoids | P47869 P28472 P18507 | 0.104254314 | 15 |
| 638 | danshenxinkun C (Neotanshinone C,tanshiquinone C) | Diterpenoids | P50406 | 0.106847218 | 8 |
| 639 | danshenxinkun C (Neotanshinone C,tanshiquinone C) | Diterpenoids | P52333 | 0.104243408 | 12 |
| 640 | danshenxinkun C (Neotanshinone C,tanshiquinone C) | Diterpenoids | P56817 | 0.108325485 | 12 |
| 641 | danshenxinkun C (Neotanshinone C,tanshiquinone C) | Diterpenoids | Q02127 | 0.106699814 | 5 |
| 642 | danshenxinkun C (Neotanshinone C,tanshiquinone C) | Diterpenoids | Q12791 | 0.103498446 | 3 |
| 643 | danshenxinkun C (Neotanshinone C,tanshiquinone C) | Diterpenoids | Q15746 | 0.102744609 | 5 |
| 644 | danshenxinkun C (Neotanshinone C,tanshiquinone C) | Diterpenoids | Q5S007 | 0.102368749 | 9 |
| 645 | danshenxinkun C (Neotanshinone C,tanshiquinone C) | Diterpenoids | Q99683 | 0.104664572 | 4 |
| 646 | danshenxinkun C (Neotanshinone C,tanshiquinone C) | Diterpenoids | Q9H2K2 | 0.104609573 | 11 |
| 647 | danshenxinkun C (Neotanshinone C,tanshiquinone C) | Diterpenoids | Q9HC97 | 0.107139434 | 3 |
| 648 | danshenxinkun C (Neotanshinone C,tanshiquinone C) | Diterpenoids | Q9Y233 | 0.108085897 | 14 |
| 649 | danshenxinkun C (Neotanshinone C,tanshiquinone C) | Diterpenoids | Q9Y2R2 | 0.111485907 | 5 |
| 650 | danshenxinkun D | Diterpenoids | O00408 | 0.103882897 | 9 |
| 651 | danshenxinkun D | Diterpenoids | O00519 | 0.110352709 | 16 |
| 652 | danshenxinkun D | Diterpenoids | O14649 | 0.100520388 | 4 |
| 653 | danshenxinkun D | Diterpenoids | O60674 | 0.103305945 | 11 |
| 654 | danshenxinkun D | Diterpenoids | O60706 | 0.103819513 | 5 |
| 655 | danshenxinkun D | Diterpenoids | O60885 | 0.104476685 | 6 |
| 656 | danshenxinkun D | Diterpenoids | O96020 P24941 P24864 | 0.104035866 | 9 |
| 657 | danshenxinkun D | Diterpenoids | P00533 | 0.104151342 | 24 |
| 658 | danshenxinkun D | Diterpenoids | P00742 | 0.111476369 | 3 |
| 659 | danshenxinkun D | Diterpenoids | P00746 | 0.101536397 | 5 |
| 660 | danshenxinkun D | Diterpenoids | P00797 | 0.104471265 | 3 |
| 661 | danshenxinkun D | Diterpenoids | P03951 | 0.106451353 | 3 |
| 662 | danshenxinkun D | Diterpenoids | P04035 | 0.166864971 | 20 |
| 663 | danshenxinkun D | Diterpenoids | P04626 | 0.104502793 | 11 |
| 664 | danshenxinkun D | Diterpenoids | P04629 | 0.107046621 | 8 |
| 665 | danshenxinkun D | Diterpenoids | P05186 | 0.104336352 | 9 |
| 666 | danshenxinkun D | Diterpenoids | P06493 | 0.102732601 | 7 |
| 667 | danshenxinkun D | Diterpenoids | P06737 | 0.103331553 | 10 |
| 668 | danshenxinkun D | Diterpenoids | P07477 | 0.103976264 | 3 |
| 669 | danshenxinkun D | Diterpenoids | P08246 | 0.10362417 | 7 |
| 670 | danshenxinkun D | Diterpenoids | P09874 | 0.106818993 | 18 |
| 671 | danshenxinkun D | Diterpenoids | P0DMS8 | 0.10464569 | 23 |
| 672 | danshenxinkun D | Diterpenoids | P10275 | 0.132131799 | 34 |
| 673 | danshenxinkun D | Diterpenoids | P11309 | 0.104805981 | 13 |
| 674 | danshenxinkun D | Diterpenoids | P14416 | 0.108887011 | 4 |
| 675 | danshenxinkun D | Diterpenoids | P14902 | 0.110454469 | 24 |
| 676 | danshenxinkun D | Diterpenoids | P17252 | 0.102754312 | 7 |
| 677 | danshenxinkun D | Diterpenoids | P21554 | 0.104626059 | 10 |
| 678 | danshenxinkun D | Diterpenoids | P23458 | 0.102916912 | 11 |
| 679 | danshenxinkun D | Diterpenoids | P24385 P11802 | 0.105464394 | 4 |
| 680 | danshenxinkun D | Diterpenoids | P24941 P78396 P20248 | 0.108328679 | 5 |
| 681 | danshenxinkun D | Diterpenoids | P25025 | 0.100320282 | 13 |
| 682 | danshenxinkun D | Diterpenoids | P25440 | 0.106747907 | 3 |
| 683 | danshenxinkun D | Diterpenoids | P25774 | 0.106840916 | 6 |
| 684 | danshenxinkun D | Diterpenoids | P27815 | 0.106747907 | 3 |
| 685 | danshenxinkun D | Diterpenoids | P28223 | 0.103919859 | 7 |
| 686 | danshenxinkun D | Diterpenoids | P28845 | 0.253303956 | 34 |
| 687 | danshenxinkun D | Diterpenoids | P29274 | 0.103202685 | 27 |
| 688 | danshenxinkun D | Diterpenoids | P29275 | 0.103333221 | 16 |
| 689 | danshenxinkun D | Diterpenoids | P30536 | 0.102095712 | 5 |
| 690 | danshenxinkun D | Diterpenoids | P30542 | 0.101318267 | 24 |
| 691 | danshenxinkun D | Diterpenoids | P31644 | 0.101037512 | 7 |
| 692 | danshenxinkun D | Diterpenoids | P32246 | 0.109647095 | 6 |
| 693 | danshenxinkun D | Diterpenoids | P34972 | 0.103169308 | 13 |
| 694 | danshenxinkun D | Diterpenoids | P34998 | 0.110755665 | 3 |
| 695 | danshenxinkun D | Diterpenoids | P35354 | 0.113487837 | 23 |
| 696 | danshenxinkun D | Diterpenoids | P37268 | 0.110958418 | 7 |
| 697 | danshenxinkun D | Diterpenoids | P41594 | 0.103693674 | 22 |
| 698 | danshenxinkun D | Diterpenoids | P42338 | 0.107541648 | 9 |
| 699 | danshenxinkun D | Diterpenoids | P42574 | 0.10400953 | 13 |
| 700 | danshenxinkun D | Diterpenoids | P43235 | 0.104158358 | 7 |
| 701 | danshenxinkun D | Diterpenoids | P43405 | 0.106351077 | 5 |
| 702 | danshenxinkun D | Diterpenoids | P48039 | 0.101354935 | 19 |
| 703 | danshenxinkun D | Diterpenoids | P48147 | 0.107233003 | 22 |
| 704 | danshenxinkun D | Diterpenoids | P49286 | 0.101354935 | 19 |
| 705 | danshenxinkun D | Diterpenoids | P49810 Q9NZ42 Q92542 Q96BI3 P49768 Q8WW43 | 0.101543467 | 9 |
| 706 | danshenxinkun D | Diterpenoids | P49840 | 0.108060801 | 5 |
| 707 | danshenxinkun D | Diterpenoids | P49841 | 0.105823985 | 11 |
| 708 | danshenxinkun D | Diterpenoids | P50406 | 0.106847218 | 8 |
| 709 | danshenxinkun D | Diterpenoids | P52333 | 0.104243408 | 12 |
| 710 | danshenxinkun D | Diterpenoids | P52732 | 0.104747724 | 9 |
| 711 | danshenxinkun D | Diterpenoids | P55210 | 0.10400953 | 13 |
| 712 | danshenxinkun D | Diterpenoids | P56373 | 0.107098941 | 5 |
| 713 | danshenxinkun D | Diterpenoids | P56817 | 0.108325485 | 12 |
| 714 | danshenxinkun D | Diterpenoids | P78527 | 0.104180881 | 6 |
| 715 | danshenxinkun D | Diterpenoids | Q00796 | 0.107710558 | 5 |
| 716 | danshenxinkun D | Diterpenoids | Q01432 | 0.101993948 | 3 |
| 717 | danshenxinkun D | Diterpenoids | Q02750 | 0.106463868 | 11 |
| 718 | danshenxinkun D | Diterpenoids | Q05655 | 0.102598878 | 5 |
| 719 | danshenxinkun D | Diterpenoids | Q08828 | 0.102451863 | 4 |
| 720 | danshenxinkun D | Diterpenoids | Q12866 | 0.103756913 | 4 |
| 721 | danshenxinkun D | Diterpenoids | Q12884 | 0.102934441 | 6 |
| 722 | danshenxinkun D | Diterpenoids | Q13627 | 0.104383674 | 7 |
| 723 | danshenxinkun D | Diterpenoids | Q13946 | 0.104692547 | 7 |
| 724 | danshenxinkun D | Diterpenoids | Q15059 | 0.106747907 | 3 |
| 725 | danshenxinkun D | Diterpenoids | Q15078 Q00535 | 0.102910354 | 14 |
| 726 | danshenxinkun D | Diterpenoids | Q16539 | 0.105438848 | 20 |
| 727 | danshenxinkun D | Diterpenoids | Q5S007 | 0.102368749 | 9 |
| 728 | danshenxinkun D | Diterpenoids | Q99572 | 0.10437669 | 25 |
| 729 | danshenxinkun D | Diterpenoids | Q99683 | 0.104664572 | 4 |
| 730 | danshenxinkun D | Diterpenoids | Q9H4B7 | 0.103451903 | 3 |
| 731 | danshenxinkun D | Diterpenoids | Q9P1W9 | 0.103344117 | 12 |
| 732 | danshenxinkun D | Diterpenoids | Q9Y233 | 0.108085897 | 14 |
| 733 | Dehydrotanshinone II A (1,2-Didehydrotanshinone IIA,1,2-Dehydrotanshinone II A,delta1-Dehydrotanshinone II(A)) | Diterpenoids | O00748 | 0.259386615 | 47 |
| 734 | Dehydrotanshinone II A (1,2-Didehydrotanshinone IIA,1,2-Dehydrotanshinone II A,delta1-Dehydrotanshinone II(A)) | Diterpenoids | O14684 | 0.116389783 | 19 |
| 735 | Dehydrotanshinone II A (1,2-Didehydrotanshinone IIA,1,2-Dehydrotanshinone II A,delta1-Dehydrotanshinone II(A)) | Diterpenoids | O14746 | 0.14728518 | 27 |
| 736 | Dehydrotanshinone II A (1,2-Didehydrotanshinone IIA,1,2-Dehydrotanshinone II A,delta1-Dehydrotanshinone II(A)) | Diterpenoids | O43613 | 0.10159278 | 9 |
| 737 | Dehydrotanshinone II A (1,2-Didehydrotanshinone IIA,1,2-Dehydrotanshinone II A,delta1-Dehydrotanshinone II(A)) | Diterpenoids | O43614 | 0.100916735 | 11 |
| 738 | Dehydrotanshinone II A (1,2-Didehydrotanshinone IIA,1,2-Dehydrotanshinone II A,delta1-Dehydrotanshinone II(A)) | Diterpenoids | O60885 | 0.104476685 | 6 |
| 739 | Dehydrotanshinone II A (1,2-Didehydrotanshinone IIA,1,2-Dehydrotanshinone II A,delta1-Dehydrotanshinone II(A)) | Diterpenoids | O75530 Q15022 Q15910 | 0.341964133 | 19 |
| 740 | Dehydrotanshinone II A (1,2-Didehydrotanshinone IIA,1,2-Dehydrotanshinone II A,delta1-Dehydrotanshinone II(A)) | Diterpenoids | O75874 | 0.1046882 | 6 |
| 741 | Dehydrotanshinone II A (1,2-Didehydrotanshinone IIA,1,2-Dehydrotanshinone II A,delta1-Dehydrotanshinone II(A)) | Diterpenoids | O76074 | 0.103099774 | 12 |
| 742 | Dehydrotanshinone II A (1,2-Didehydrotanshinone IIA,1,2-Dehydrotanshinone II A,delta1-Dehydrotanshinone II(A)) | Diterpenoids | P00533 | 0.104151342 | 24 |
| 743 | Dehydrotanshinone II A (1,2-Didehydrotanshinone IIA,1,2-Dehydrotanshinone II A,delta1-Dehydrotanshinone II(A)) | Diterpenoids | P06276 | 0.130116287 | 14 |
| 744 | Dehydrotanshinone II A (1,2-Didehydrotanshinone IIA,1,2-Dehydrotanshinone II A,delta1-Dehydrotanshinone II(A)) | Diterpenoids | P06401 | 0.108868706 | 15 |
| 745 | Dehydrotanshinone II A (1,2-Didehydrotanshinone IIA,1,2-Dehydrotanshinone II A,delta1-Dehydrotanshinone II(A)) | Diterpenoids | P07099 | 0.102416978 | 3 |
| 746 | Dehydrotanshinone II A (1,2-Didehydrotanshinone IIA,1,2-Dehydrotanshinone II A,delta1-Dehydrotanshinone II(A)) | Diterpenoids | P08069 | 0.112934324 | 3 |
| 747 | Dehydrotanshinone II A (1,2-Didehydrotanshinone IIA,1,2-Dehydrotanshinone II A,delta1-Dehydrotanshinone II(A)) | Diterpenoids | P08575 | 0.109148612 | 27 |
| 748 | Dehydrotanshinone II A (1,2-Didehydrotanshinone IIA,1,2-Dehydrotanshinone II A,delta1-Dehydrotanshinone II(A)) | Diterpenoids | P09874 | 0.106818993 | 18 |
| 749 | Dehydrotanshinone II A (1,2-Didehydrotanshinone IIA,1,2-Dehydrotanshinone II A,delta1-Dehydrotanshinone II(A)) | Diterpenoids | P0DMS8 | 0.10464569 | 23 |
| 750 | Dehydrotanshinone II A (1,2-Didehydrotanshinone IIA,1,2-Dehydrotanshinone II A,delta1-Dehydrotanshinone II(A)) | Diterpenoids | P10275 | 0.132131799 | 34 |
| 751 | Dehydrotanshinone II A (1,2-Didehydrotanshinone IIA,1,2-Dehydrotanshinone II A,delta1-Dehydrotanshinone II(A)) | Diterpenoids | P11511 | 0.139056613 | 29 |
| 752 | Dehydrotanshinone II A (1,2-Didehydrotanshinone IIA,1,2-Dehydrotanshinone II A,delta1-Dehydrotanshinone II(A)) | Diterpenoids | P11940 | 0.1046882 | 6 |
| 753 | Dehydrotanshinone II A (1,2-Didehydrotanshinone IIA,1,2-Dehydrotanshinone II A,delta1-Dehydrotanshinone II(A)) | Diterpenoids | P12268 | 0.104524224 | 13 |
| 754 | Dehydrotanshinone II A (1,2-Didehydrotanshinone IIA,1,2-Dehydrotanshinone II A,delta1-Dehydrotanshinone II(A)) | Diterpenoids | P15121 | 0.267155221 | 39 |
| 755 | Dehydrotanshinone II A (1,2-Didehydrotanshinone IIA,1,2-Dehydrotanshinone II A,delta1-Dehydrotanshinone II(A)) | Diterpenoids | P16083 | 0.112425971 | 14 |
| 756 | Dehydrotanshinone II A (1,2-Didehydrotanshinone IIA,1,2-Dehydrotanshinone II A,delta1-Dehydrotanshinone II(A)) | Diterpenoids | P22303 | 0.287353637 | 26 |
| 757 | Dehydrotanshinone II A (1,2-Didehydrotanshinone IIA,1,2-Dehydrotanshinone II A,delta1-Dehydrotanshinone II(A)) | Diterpenoids | P23141 | 0.275271523 | 31 |
| 758 | Dehydrotanshinone II A (1,2-Didehydrotanshinone IIA,1,2-Dehydrotanshinone II A,delta1-Dehydrotanshinone II(A)) | Diterpenoids | P25440 | 0.106747907 | 3 |
| 759 | Dehydrotanshinone II A (1,2-Didehydrotanshinone IIA,1,2-Dehydrotanshinone II A,delta1-Dehydrotanshinone II(A)) | Diterpenoids | P28223 | 0.103919859 | 7 |
| 760 | Dehydrotanshinone II A (1,2-Didehydrotanshinone IIA,1,2-Dehydrotanshinone II A,delta1-Dehydrotanshinone II(A)) | Diterpenoids | P28335 | 0.104521595 | 9 |
| 761 | Dehydrotanshinone II A (1,2-Didehydrotanshinone IIA,1,2-Dehydrotanshinone II A,delta1-Dehydrotanshinone II(A)) | Diterpenoids | P28845 | 0.253303956 | 34 |
| 762 | Dehydrotanshinone II A (1,2-Didehydrotanshinone IIA,1,2-Dehydrotanshinone II A,delta1-Dehydrotanshinone II(A)) | Diterpenoids | P29274 | 0.103202685 | 27 |
| 763 | Dehydrotanshinone II A (1,2-Didehydrotanshinone IIA,1,2-Dehydrotanshinone II A,delta1-Dehydrotanshinone II(A)) | Diterpenoids | P29275 | 0.103333221 | 16 |
| 764 | Dehydrotanshinone II A (1,2-Didehydrotanshinone IIA,1,2-Dehydrotanshinone II A,delta1-Dehydrotanshinone II(A)) | Diterpenoids | P29350 | 0.204438393 | 44 |
| 765 | Dehydrotanshinone II A (1,2-Didehydrotanshinone IIA,1,2-Dehydrotanshinone II A,delta1-Dehydrotanshinone II(A)) | Diterpenoids | P30305 | 0.185734372 | 40 |
| 766 | Dehydrotanshinone II A (1,2-Didehydrotanshinone IIA,1,2-Dehydrotanshinone II A,delta1-Dehydrotanshinone II(A)) | Diterpenoids | P30307 | 0.105546712 | 12 |
| 767 | Dehydrotanshinone II A (1,2-Didehydrotanshinone IIA,1,2-Dehydrotanshinone II A,delta1-Dehydrotanshinone II(A)) | Diterpenoids | P30542 | 0.101318267 | 24 |
| 768 | Dehydrotanshinone II A (1,2-Didehydrotanshinone IIA,1,2-Dehydrotanshinone II A,delta1-Dehydrotanshinone II(A)) | Diterpenoids | P32247 | 0.103071649 | 5 |
| 769 | Dehydrotanshinone II A (1,2-Didehydrotanshinone IIA,1,2-Dehydrotanshinone II A,delta1-Dehydrotanshinone II(A)) | Diterpenoids | P35348 | 0.101281367 | 4 |
| 770 | Dehydrotanshinone II A (1,2-Didehydrotanshinone IIA,1,2-Dehydrotanshinone II A,delta1-Dehydrotanshinone II(A)) | Diterpenoids | P35968 | 0.105052994 | 26 |
| 771 | Dehydrotanshinone II A (1,2-Didehydrotanshinone IIA,1,2-Dehydrotanshinone II A,delta1-Dehydrotanshinone II(A)) | Diterpenoids | P41595 | 0.102913571 | 7 |
| 772 | Dehydrotanshinone II A (1,2-Didehydrotanshinone IIA,1,2-Dehydrotanshinone II A,delta1-Dehydrotanshinone II(A)) | Diterpenoids | P43116 | 0.114821812 | 13 |
| 773 | Dehydrotanshinone II A (1,2-Didehydrotanshinone IIA,1,2-Dehydrotanshinone II A,delta1-Dehydrotanshinone II(A)) | Diterpenoids | P48039 | 0.101354935 | 19 |
| 774 | Dehydrotanshinone II A (1,2-Didehydrotanshinone IIA,1,2-Dehydrotanshinone II A,delta1-Dehydrotanshinone II(A)) | Diterpenoids | P49286 | 0.101354935 | 19 |
| 775 | Dehydrotanshinone II A (1,2-Didehydrotanshinone IIA,1,2-Dehydrotanshinone II A,delta1-Dehydrotanshinone II(A)) | Diterpenoids | Q01959 | 0.107731401 | 12 |
| 776 | Dehydrotanshinone II A (1,2-Didehydrotanshinone IIA,1,2-Dehydrotanshinone II A,delta1-Dehydrotanshinone II(A)) | Diterpenoids | Q06124 | 0.211032909 | 40 |
| 777 | Dehydrotanshinone II A (1,2-Didehydrotanshinone IIA,1,2-Dehydrotanshinone II A,delta1-Dehydrotanshinone II(A)) | Diterpenoids | Q07343 | 0.107582642 | 6 |
| 778 | Dehydrotanshinone II A (1,2-Didehydrotanshinone IIA,1,2-Dehydrotanshinone II A,delta1-Dehydrotanshinone II(A)) | Diterpenoids | Q09028 Q16576 O75530 Q15022 Q15910 | 0.142197972 | 14 |
| 779 | Dehydrotanshinone II A (1,2-Didehydrotanshinone IIA,1,2-Dehydrotanshinone II A,delta1-Dehydrotanshinone II(A)) | Diterpenoids | Q13936 | 0.102416978 | 3 |
| 780 | Dehydrotanshinone II A (1,2-Didehydrotanshinone IIA,1,2-Dehydrotanshinone II A,delta1-Dehydrotanshinone II(A)) | Diterpenoids | Q13946 | 0.104692547 | 7 |
| 781 | Dehydrotanshinone II A (1,2-Didehydrotanshinone IIA,1,2-Dehydrotanshinone II A,delta1-Dehydrotanshinone II(A)) | Diterpenoids | Q15059 | 0.106747907 | 3 |
| 782 | Dehydrotanshinone II A (1,2-Didehydrotanshinone IIA,1,2-Dehydrotanshinone II A,delta1-Dehydrotanshinone II(A)) | Diterpenoids | Q16539 | 0.105438848 | 20 |
| 783 | Dehydrotanshinone II A (1,2-Didehydrotanshinone IIA,1,2-Dehydrotanshinone II A,delta1-Dehydrotanshinone II(A)) | Diterpenoids | Q92731 | 0.117586956 | 23 |
| 784 | Dehydrotanshinone II A (1,2-Didehydrotanshinone IIA,1,2-Dehydrotanshinone II A,delta1-Dehydrotanshinone II(A)) | Diterpenoids | Q99572 | 0.10437669 | 25 |
| 785 | Dehydrotanshinone II A (1,2-Didehydrotanshinone IIA,1,2-Dehydrotanshinone II A,delta1-Dehydrotanshinone II(A)) | Diterpenoids | Q9H2K2 | 0.104609573 | 11 |
| 786 | Dehydrotanshinone II A (1,2-Didehydrotanshinone IIA,1,2-Dehydrotanshinone II A,delta1-Dehydrotanshinone II(A)) | Diterpenoids | Q9Y233 | 0.108085897 | 14 |
| 787 | deoxyneocryptotanshinone | Diterpenoids | B2RXH2 | 0.104871629 | 12 |
| 788 | deoxyneocryptotanshinone | Diterpenoids | O60427 | 0.10804089 | 3 |
| 789 | deoxyneocryptotanshinone | Diterpenoids | O75874 | 0.1046882 | 6 |
| 790 | deoxyneocryptotanshinone | Diterpenoids | O95271 | 0.102617882 | 7 |
| 791 | deoxyneocryptotanshinone | Diterpenoids | P00338 | 0.106699814 | 5 |
| 792 | deoxyneocryptotanshinone | Diterpenoids | P00533 | 0.104151342 | 24 |
| 793 | deoxyneocryptotanshinone | Diterpenoids | P04626 | 0.104502793 | 11 |
| 794 | deoxyneocryptotanshinone | Diterpenoids | P08183 | 0.104694286 | 5 |
| 795 | deoxyneocryptotanshinone | Diterpenoids | P08246 | 0.10362417 | 7 |
| 796 | deoxyneocryptotanshinone | Diterpenoids | P09874 | 0.106818993 | 18 |
| 797 | deoxyneocryptotanshinone | Diterpenoids | P11229 | 0.103603883 | 11 |
| 798 | deoxyneocryptotanshinone | Diterpenoids | P14867 | 0.10300422 | 7 |
| 799 | deoxyneocryptotanshinone | Diterpenoids | P14902 | 0.110454469 | 24 |
| 800 | deoxyneocryptotanshinone | Diterpenoids | P15121 | 0.267155221 | 39 |
| 801 | deoxyneocryptotanshinone | Diterpenoids | P16050 | 0.105228934 | 6 |
| 802 | deoxyneocryptotanshinone | Diterpenoids | P18054 | 0.103847438 | 5 |
| 803 | deoxyneocryptotanshinone | Diterpenoids | P20309 | 0.105165619 | 10 |
| 804 | deoxyneocryptotanshinone | Diterpenoids | P21397 | 0.102816353 | 15 |
| 805 | deoxyneocryptotanshinone | Diterpenoids | P23219 | 0.109524644 | 16 |
| 806 | deoxyneocryptotanshinone | Diterpenoids | P24557 | 0.107944463 | 3 |
| 807 | deoxyneocryptotanshinone | Diterpenoids | P27338 | 0.105021471 | 16 |
| 808 | deoxyneocryptotanshinone | Diterpenoids | P28472 P18507 P14867 | 0.103855577 | 16 |
| 809 | deoxyneocryptotanshinone | Diterpenoids | P28472 P18507 P31644 | 0.103791839 | 19 |
| 810 | deoxyneocryptotanshinone | Diterpenoids | P28472 P34903 P18507 | 0.103855577 | 16 |
| 811 | deoxyneocryptotanshinone | Diterpenoids | P29274 | 0.103202685 | 27 |
| 812 | deoxyneocryptotanshinone | Diterpenoids | P29371 | 0.103946422 | 5 |
| 813 | deoxyneocryptotanshinone | Diterpenoids | P30305 | 0.185734372 | 40 |
| 814 | deoxyneocryptotanshinone | Diterpenoids | P30542 | 0.101318267 | 24 |
| 815 | deoxyneocryptotanshinone | Diterpenoids | P35228 | 0.112440313 | 15 |
| 816 | deoxyneocryptotanshinone | Diterpenoids | P35354 | 0.113487837 | 23 |
| 817 | deoxyneocryptotanshinone | Diterpenoids | P37059 | 0.106459925 | 6 |
| 818 | deoxyneocryptotanshinone | Diterpenoids | P41180 | 0.104664572 | 4 |
| 819 | deoxyneocryptotanshinone | Diterpenoids | P47869 P28472 P18507 | 0.104254314 | 15 |
| 820 | deoxyneocryptotanshinone | Diterpenoids | P49354 P49356 | 0.109904857 | 24 |
| 821 | deoxyneocryptotanshinone | Diterpenoids | Q02127 | 0.106699814 | 5 |
| 822 | deoxyneocryptotanshinone | Diterpenoids | Q05513 | 0.104564874 | 3 |
| 823 | deoxyneocryptotanshinone | Diterpenoids | Q07343 | 0.107582642 | 6 |
| 824 | deoxyneocryptotanshinone | Diterpenoids | Q99572 | 0.10437669 | 25 |
| 825 | deoxyneocryptotanshinone | Diterpenoids | Q99683 | 0.104664572 | 4 |
| 826 | deoxyneocryptotanshinone | Diterpenoids | Q9H2K2 | 0.104609573 | 11 |
| 827 | deoxyneocryptotanshinone | Diterpenoids | Q9H3R0 | 0.106959422 | 3 |
| 828 | deoxyneocryptotanshinone | Diterpenoids | Q9HC97 | 0.107139434 | 3 |
| 829 | deoxyneocryptotanshinone | Diterpenoids | Q9UNQ0 | 0.106050933 | 5 |
| 830 | deoxyneocryptotanshinone | Diterpenoids | Q9Y5Z0 | 0.106959422 | 3 |
| 831 | dihydroisotanshinone I (isodihydrotanshinone) | Diterpenoids | O95271 | 0.102617882 | 7 |
| 832 | dihydroisotanshinone I (isodihydrotanshinone) | Diterpenoids | P07333 | 0.104063456 | 9 |
| 833 | dihydroisotanshinone I (isodihydrotanshinone) | Diterpenoids | P0DMS8 | 0.10464569 | 23 |
| 834 | dihydroisotanshinone I (isodihydrotanshinone) | Diterpenoids | P11511 | 0.139056613 | 29 |
| 835 | dihydroisotanshinone I (isodihydrotanshinone) | Diterpenoids | P14902 | 0.110454469 | 24 |
| 836 | dihydroisotanshinone I (isodihydrotanshinone) | Diterpenoids | P15121 | 0.267155221 | 39 |
| 837 | dihydroisotanshinone I (isodihydrotanshinone) | Diterpenoids | P21554 | 0.104626059 | 10 |
| 838 | dihydroisotanshinone I (isodihydrotanshinone) | Diterpenoids | P21980 | 0.101957459 | 8 |
| 839 | dihydroisotanshinone I (isodihydrotanshinone) | Diterpenoids | P24863 P49336 | 0.102633551 | 4 |
| 840 | dihydroisotanshinone I (isodihydrotanshinone) | Diterpenoids | P28472 P18507 P14867 | 0.103855577 | 16 |
| 841 | dihydroisotanshinone I (isodihydrotanshinone) | Diterpenoids | P28472 P18507 P31644 | 0.103791839 | 19 |
| 842 | dihydroisotanshinone I (isodihydrotanshinone) | Diterpenoids | P28472 P34903 P18507 | 0.103855577 | 16 |
| 843 | dihydroisotanshinone I (isodihydrotanshinone) | Diterpenoids | P28845 | 0.253303956 | 34 |
| 844 | dihydroisotanshinone I (isodihydrotanshinone) | Diterpenoids | P30304 | 0.109145376 | 25 |
| 845 | dihydroisotanshinone I (isodihydrotanshinone) | Diterpenoids | P34913 | 0.106959422 | 6 |
| 846 | dihydroisotanshinone I (isodihydrotanshinone) | Diterpenoids | P34972 | 0.103169308 | 13 |
| 847 | dihydroisotanshinone I (isodihydrotanshinone) | Diterpenoids | P35354 | 0.113487837 | 23 |
| 848 | dihydroisotanshinone I (isodihydrotanshinone) | Diterpenoids | P41594 | 0.103693674 | 22 |
| 849 | dihydroisotanshinone I (isodihydrotanshinone) | Diterpenoids | P47869 P28472 P18507 | 0.104254314 | 15 |
| 850 | dihydroisotanshinone I (isodihydrotanshinone) | Diterpenoids | P48039 | 0.101354935 | 19 |
| 851 | dihydroisotanshinone I (isodihydrotanshinone) | Diterpenoids | P48147 | 0.107233003 | 22 |
| 852 | dihydroisotanshinone I (isodihydrotanshinone) | Diterpenoids | P49286 | 0.101354935 | 19 |
| 853 | dihydroisotanshinone I (isodihydrotanshinone) | Diterpenoids | P52732 | 0.104747724 | 9 |
| 854 | dihydroisotanshinone I (isodihydrotanshinone) | Diterpenoids | Q01959 | 0.107731401 | 12 |
| 855 | dihydroisotanshinone I (isodihydrotanshinone) | Diterpenoids | Q02083 | 0.102416978 | 6 |
| 856 | dihydroisotanshinone I (isodihydrotanshinone) | Diterpenoids | Q99572 | 0.10437669 | 25 |
| 857 | dihydroisotanshinone II | Diterpenoids | B2RXH2 | 0.104871629 | 12 |
| 858 | dihydroisotanshinone II | Diterpenoids | O00748 | 0.259386615 | 47 |
| 859 | dihydroisotanshinone II | Diterpenoids | O43570 | 0.137972322 | 10 |
| 860 | dihydroisotanshinone II | Diterpenoids | O60674 | 0.103305945 | 11 |
| 861 | dihydroisotanshinone II | Diterpenoids | O95551 | 0.106334502 | 10 |
| 862 | dihydroisotanshinone II | Diterpenoids | P00519 | 0.104821195 | 5 |
| 863 | dihydroisotanshinone II | Diterpenoids | P00533 | 0.104151342 | 24 |
| 864 | dihydroisotanshinone II | Diterpenoids | P00915 | 0.106312298 | 8 |
| 865 | dihydroisotanshinone II | Diterpenoids | P00918 | 0.141292356 | 8 |
| 866 | dihydroisotanshinone II | Diterpenoids | P05186 | 0.104336352 | 9 |
| 867 | dihydroisotanshinone II | Diterpenoids | P07333 | 0.104063456 | 9 |
| 868 | dihydroisotanshinone II | Diterpenoids | P07949 | 0.103471701 | 8 |
| 869 | dihydroisotanshinone II | Diterpenoids | P08172 | 0.117318329 | 18 |
| 870 | dihydroisotanshinone II | Diterpenoids | P08246 | 0.10362417 | 7 |
| 871 | dihydroisotanshinone II | Diterpenoids | P08575 | 0.109148612 | 27 |
| 872 | dihydroisotanshinone II | Diterpenoids | P09601 | 0.103663418 | 3 |
| 873 | dihydroisotanshinone II | Diterpenoids | P09874 | 0.106818993 | 18 |
| 874 | dihydroisotanshinone II | Diterpenoids | P0DMS8 | 0.10464569 | 23 |
| 875 | dihydroisotanshinone II | Diterpenoids | P10275 | 0.132131799 | 34 |
| 876 | dihydroisotanshinone II | Diterpenoids | P11229 | 0.103603883 | 11 |
| 877 | dihydroisotanshinone II | Diterpenoids | P11362 | 0.105847024 | 4 |
| 878 | dihydroisotanshinone II | Diterpenoids | P11388 | 0.106757964 | 8 |
| 879 | dihydroisotanshinone II | Diterpenoids | P12931 | 0.104821195 | 5 |
| 880 | dihydroisotanshinone II | Diterpenoids | P14867 | 0.10300422 | 7 |
| 881 | dihydroisotanshinone II | Diterpenoids | P14902 | 0.110454469 | 24 |
| 882 | dihydroisotanshinone II | Diterpenoids | P15056 | 0.101798823 | 4 |
| 883 | dihydroisotanshinone II | Diterpenoids | P15121 | 0.267155221 | 39 |
| 884 | dihydroisotanshinone II | Diterpenoids | P15538 | 0.10615894 | 5 |
| 885 | dihydroisotanshinone II | Diterpenoids | P17948 | 0.105554897 | 13 |
| 886 | dihydroisotanshinone II | Diterpenoids | P18031 | 0.474772974 | 23 |
| 887 | dihydroisotanshinone II | Diterpenoids | P19099 | 0.10615894 | 5 |
| 888 | dihydroisotanshinone II | Diterpenoids | P20309 | 0.105165619 | 10 |
| 889 | dihydroisotanshinone II | Diterpenoids | P22303 | 0.287353637 | 26 |
| 890 | dihydroisotanshinone II | Diterpenoids | P23141 | 0.275271523 | 31 |
| 891 | dihydroisotanshinone II | Diterpenoids | P23458 | 0.102916912 | 11 |
| 892 | dihydroisotanshinone II | Diterpenoids | P23975 | 0.115012308 | 10 |
| 893 | dihydroisotanshinone II | Diterpenoids | P24864 P24941 | 0.102540734 | 7 |
| 894 | dihydroisotanshinone II | Diterpenoids | P25025 | 0.100320282 | 13 |
| 895 | dihydroisotanshinone II | Diterpenoids | P28223 | 0.103919859 | 7 |
| 896 | dihydroisotanshinone II | Diterpenoids | P28335 | 0.104521595 | 9 |
| 897 | dihydroisotanshinone II | Diterpenoids | P28472 P18507 P14867 | 0.103855577 | 16 |
| 898 | dihydroisotanshinone II | Diterpenoids | P28472 P18507 P31644 | 0.103791839 | 19 |
| 899 | dihydroisotanshinone II | Diterpenoids | P28472 P34903 P18507 | 0.103855577 | 16 |
| 900 | dihydroisotanshinone II | Diterpenoids | P28562 | 0.104003317 | 12 |
| 901 | dihydroisotanshinone II | Diterpenoids | P29274 | 0.103202685 | 27 |
| 902 | dihydroisotanshinone II | Diterpenoids | P29275 | 0.103333221 | 16 |
| 903 | dihydroisotanshinone II | Diterpenoids | P29350 | 0.204438393 | 44 |
| 904 | dihydroisotanshinone II | Diterpenoids | P29597 | 0.106959422 | 3 |
| 905 | dihydroisotanshinone II | Diterpenoids | P30305 | 0.185734372 | 40 |
| 906 | dihydroisotanshinone II | Diterpenoids | P30542 | 0.101318267 | 24 |
| 907 | dihydroisotanshinone II | Diterpenoids | P31644 | 0.101037512 | 7 |
| 908 | dihydroisotanshinone II | Diterpenoids | P31645 | 0.1201852 | 17 |
| 909 | dihydroisotanshinone II | Diterpenoids | P34972 | 0.103169308 | 13 |
| 910 | dihydroisotanshinone II | Diterpenoids | P35354 | 0.113487837 | 23 |
| 911 | dihydroisotanshinone II | Diterpenoids | P35968 | 0.105052994 | 26 |
| 912 | dihydroisotanshinone II | Diterpenoids | P40763 | 0.268996335 | 13 |
| 913 | dihydroisotanshinone II | Diterpenoids | P41594 | 0.103693674 | 22 |
| 914 | dihydroisotanshinone II | Diterpenoids | P43235 | 0.104158358 | 7 |
| 915 | dihydroisotanshinone II | Diterpenoids | P45983 | 0.105088123 | 17 |
| 916 | dihydroisotanshinone II | Diterpenoids | P45984 | 0.10939337 | 4 |
| 917 | dihydroisotanshinone II | Diterpenoids | P47869 P28472 P18507 | 0.104254314 | 15 |
| 918 | dihydroisotanshinone II | Diterpenoids | P48039 | 0.101354935 | 19 |
| 919 | dihydroisotanshinone II | Diterpenoids | P48147 | 0.107233003 | 22 |
| 920 | dihydroisotanshinone II | Diterpenoids | P49286 | 0.101354935 | 19 |
| 921 | dihydroisotanshinone II | Diterpenoids | P49354 P49356 | 0.109904857 | 24 |
| 922 | dihydroisotanshinone II | Diterpenoids | P49841 | 0.105823985 | 11 |
| 923 | dihydroisotanshinone II | Diterpenoids | P52333 | 0.104243408 | 12 |
| 924 | dihydroisotanshinone II | Diterpenoids | P53779 | 0.103177433 | 9 |
| 925 | dihydroisotanshinone II | Diterpenoids | P56817 | 0.108325485 | 12 |
| 926 | dihydroisotanshinone II | Diterpenoids | Q01959 | 0.107731401 | 12 |
| 927 | dihydroisotanshinone II | Diterpenoids | Q02083 | 0.102416978 | 6 |
| 928 | dihydroisotanshinone II | Diterpenoids | Q06124 | 0.211032909 | 40 |
| 929 | dihydroisotanshinone II | Diterpenoids | Q07343 | 0.107582642 | 6 |
| 930 | dihydroisotanshinone II | Diterpenoids | Q08499 | 0.31881944 | 14 |
| 931 | dihydroisotanshinone II | Diterpenoids | Q12884 | 0.102934441 | 6 |
| 932 | dihydroisotanshinone II | Diterpenoids | Q13936 | 0.102416978 | 3 |
| 933 | dihydroisotanshinone II | Diterpenoids | Q13946 | 0.104692547 | 7 |
| 934 | dihydroisotanshinone II | Diterpenoids | Q14833 | 0.102633551 | 4 |
| 935 | dihydroisotanshinone II | Diterpenoids | Q15761 | 0.10562303 | 4 |
| 936 | dihydroisotanshinone II | Diterpenoids | Q15858 | 0.101761829 | 5 |
| 937 | dihydroisotanshinone II | Diterpenoids | Q16539 | 0.105438848 | 20 |
| 938 | dihydroisotanshinone II | Diterpenoids | Q16790 | 0.103433429 | 9 |
| 939 | dihydroisotanshinone II | Diterpenoids | Q5S007 | 0.102368749 | 9 |
| 940 | dihydroisotanshinone II | Diterpenoids | Q92731 | 0.117586956 | 23 |
| 941 | dihydroisotanshinone II | Diterpenoids | Q9H2K2 | 0.104609573 | 11 |
| 942 | dihydroisotanshinone II | Diterpenoids | Q9HBH9 | 0.10939337 | 4 |
| 943 | dihydroisotanshinone II | Diterpenoids | Q9UDY8 | 0.107158923 | 10 |
| 944 | dihydroisotanshinone II | Diterpenoids | Q9UNQ0 | 0.106050933 | 5 |
| 945 | dihydronortanshinone | Diterpenoids | B2RXH2 | 0.104871629 | 12 |
| 946 | dihydronortanshinone | Diterpenoids | O00329 | 0.105574288 | 6 |
| 947 | dihydronortanshinone | Diterpenoids | O00748 | 0.259386615 | 47 |
| 948 | dihydronortanshinone | Diterpenoids | O14757 | 0.106739614 | 8 |
| 949 | dihydronortanshinone | Diterpenoids | O14965 | 0.107316885 | 8 |
| 950 | dihydronortanshinone | Diterpenoids | O43613 | 0.10159278 | 9 |
| 951 | dihydronortanshinone | Diterpenoids | O43614 | 0.100916735 | 11 |
| 952 | dihydronortanshinone | Diterpenoids | O60885 | 0.104476685 | 6 |
| 953 | dihydronortanshinone | Diterpenoids | O75874 | 0.1046882 | 6 |
| 954 | dihydronortanshinone | Diterpenoids | O95551 | 0.106334502 | 10 |
| 955 | dihydronortanshinone | Diterpenoids | P00533 | 0.104151342 | 24 |
| 956 | dihydronortanshinone | Diterpenoids | P00746 | 0.101536397 | 5 |
| 957 | dihydronortanshinone | Diterpenoids | P03956 | 0.109842942 | 8 |
| 958 | dihydronortanshinone | Diterpenoids | P04629 | 0.107046621 | 8 |
| 959 | dihydronortanshinone | Diterpenoids | P06239 | 0.102843593 | 5 |
| 960 | dihydronortanshinone | Diterpenoids | P06737 | 0.103331553 | 10 |
| 961 | dihydronortanshinone | Diterpenoids | P07384 | 0.10807591 | 4 |
| 962 | dihydronortanshinone | Diterpenoids | P08246 | 0.10362417 | 7 |
| 963 | dihydronortanshinone | Diterpenoids | P08253 | 0.106581489 | 9 |
| 964 | dihydronortanshinone | Diterpenoids | P08254 | 0.109296209 | 8 |
| 965 | dihydronortanshinone | Diterpenoids | P08473 | 0.108391881 | 3 |
| 966 | dihydronortanshinone | Diterpenoids | P08575 | 0.109148612 | 27 |
| 967 | dihydronortanshinone | Diterpenoids | P09211 | 0.100367415 | 3 |
| 968 | dihydronortanshinone | Diterpenoids | P09874 | 0.106818993 | 18 |
| 969 | dihydronortanshinone | Diterpenoids | P10275 | 0.132131799 | 34 |
| 970 | dihydronortanshinone | Diterpenoids | P11387 | 0.113455556 | 13 |
| 971 | dihydronortanshinone | Diterpenoids | P14780 | 0.106689076 | 6 |
| 972 | dihydronortanshinone | Diterpenoids | P14902 | 0.110454469 | 24 |
| 973 | dihydronortanshinone | Diterpenoids | P15121 | 0.267155221 | 39 |
| 974 | dihydronortanshinone | Diterpenoids | P16083 | 0.112425971 | 14 |
| 975 | dihydronortanshinone | Diterpenoids | P16234 | 0.100367415 | 3 |
| 976 | dihydronortanshinone | Diterpenoids | P17948 | 0.105554897 | 13 |
| 977 | dihydronortanshinone | Diterpenoids | P21980 | 0.101957459 | 8 |
| 978 | dihydronortanshinone | Diterpenoids | P22303 | 0.287353637 | 26 |
| 979 | dihydronortanshinone | Diterpenoids | P22460 | 0.106959422 | 3 |
| 980 | dihydronortanshinone | Diterpenoids | P23141 | 0.275271523 | 31 |
| 981 | dihydronortanshinone | Diterpenoids | P23219 | 0.109524644 | 16 |
| 982 | dihydronortanshinone | Diterpenoids | P23458 | 0.102916912 | 11 |
| 983 | dihydronortanshinone | Diterpenoids | P24941 | 0.106080884 | 8 |
| 984 | dihydronortanshinone | Diterpenoids | P24941 P78396 P20248 | 0.108328679 | 5 |
| 985 | dihydronortanshinone | Diterpenoids | P25774 | 0.106840916 | 6 |
| 986 | dihydronortanshinone | Diterpenoids | P27361 | 0.105473292 | 5 |
| 987 | dihydronortanshinone | Diterpenoids | P27487 | 0.104386227 | 3 |
| 988 | dihydronortanshinone | Diterpenoids | P28482 | 0.111100851 | 4 |
| 989 | dihydronortanshinone | Diterpenoids | P28562 | 0.104003317 | 12 |
| 990 | dihydronortanshinone | Diterpenoids | P29350 | 0.204438393 | 44 |
| 991 | dihydronortanshinone | Diterpenoids | P30291 | 0.103962076 | 3 |
| 992 | dihydronortanshinone | Diterpenoids | P34913 | 0.106959422 | 6 |
| 993 | dihydronortanshinone | Diterpenoids | P34972 | 0.103169308 | 13 |
| 994 | dihydronortanshinone | Diterpenoids | P35968 | 0.105052994 | 26 |
| 995 | dihydronortanshinone | Diterpenoids | P36888 | 0.102892289 | 8 |
| 996 | dihydronortanshinone | Diterpenoids | P40763 | 0.268996335 | 13 |
| 997 | dihydronortanshinone | Diterpenoids | P41594 | 0.103693674 | 22 |
| 998 | dihydronortanshinone | Diterpenoids | P42336 | 0.108042586 | 13 |
| 999 | dihydronortanshinone | Diterpenoids | P42338 | 0.107541648 | 9 |
| 1000 | dihydronortanshinone | Diterpenoids | P43235 | 0.104158358 | 7 |
| 1001 | dihydronortanshinone | Diterpenoids | P43405 | 0.106351077 | 5 |
| 1002 | dihydronortanshinone | Diterpenoids | P45452 | 0.106586322 | 6 |
| 1003 | dihydronortanshinone | Diterpenoids | P48039 | 0.101354935 | 19 |
| 1004 | dihydronortanshinone | Diterpenoids | P48147 | 0.107233003 | 22 |
| 1005 | dihydronortanshinone | Diterpenoids | P49286 | 0.101354935 | 19 |
| 1006 | dihydronortanshinone | Diterpenoids | P49354 P49356 | 0.109904857 | 24 |
| 1007 | dihydronortanshinone | Diterpenoids | P49810 Q9NZ42 Q92542 Q96BI3 P49768 Q8WW43 | 0.101543467 | 9 |
| 1008 | dihydronortanshinone | Diterpenoids | P49841 | 0.105823985 | 11 |
| 1009 | dihydronortanshinone | Diterpenoids | P50406 | 0.106847218 | 8 |
| 1010 | dihydronortanshinone | Diterpenoids | P52333 | 0.104243408 | 12 |
| 1011 | dihydronortanshinone | Diterpenoids | P78536 | 0.104291862 | 9 |
| 1012 | dihydronortanshinone | Diterpenoids | Q02750 | 0.106463868 | 11 |
| 1013 | dihydronortanshinone | Diterpenoids | Q06124 | 0.211032909 | 40 |
| 1014 | dihydronortanshinone | Diterpenoids | Q12884 | 0.102934441 | 6 |
| 1015 | dihydronortanshinone | Diterpenoids | Q13547 | 0.110937969 | 6 |
| 1016 | dihydronortanshinone | Diterpenoids | Q13627 | 0.104383674 | 7 |
| 1017 | dihydronortanshinone | Diterpenoids | Q15078 Q00535 | 0.102910354 | 14 |
| 1018 | dihydronortanshinone | Diterpenoids | Q16539 | 0.105438848 | 20 |
| 1019 | dihydronortanshinone | Diterpenoids | Q92731 | 0.117586956 | 23 |
| 1020 | dihydronortanshinone | Diterpenoids | Q92793 | 0.102416978 | 3 |
| 1021 | dihydronortanshinone | Diterpenoids | Q96GD4 | 0.103151027 | 4 |
| 1022 | dihydronortanshinone | Diterpenoids | Q99572 | 0.10437669 | 25 |
| 1023 | dihydronortanshinone | Diterpenoids | Q9UBN7 | 0.106238718 | 3 |
| 1024 | dihydronortanshinone | Diterpenoids | Q9UDY8 | 0.107158923 | 10 |
| 1025 | dihydronortanshinone | Diterpenoids | Q9Y5N1 | 0.101047212 | 6 |
| 1026 | Dihydrotanshinone I (15,16-Dihydrotanshinone I) | Diterpenoids | B2RXH2 | 0.104871629 | 12 |
| 1027 | Dihydrotanshinone I (15,16-Dihydrotanshinone I) | Diterpenoids | O00748 | 0.259386615 | 47 |
| 1028 | Dihydrotanshinone I (15,16-Dihydrotanshinone I) | Diterpenoids | O43570 | 0.137972322 | 10 |
| 1029 | Dihydrotanshinone I (15,16-Dihydrotanshinone I) | Diterpenoids | O95551 | 0.106334502 | 10 |
| 1030 | Dihydrotanshinone I (15,16-Dihydrotanshinone I) | Diterpenoids | O96020 P24941 P24864 | 0.104035866 | 9 |
| 1031 | Dihydrotanshinone I (15,16-Dihydrotanshinone I) | Diterpenoids | P00533 | 0.104151342 | 24 |
| 1032 | Dihydrotanshinone I (15,16-Dihydrotanshinone I) | Diterpenoids | P04626 | 0.104502793 | 11 |
| 1033 | Dihydrotanshinone I (15,16-Dihydrotanshinone I) | Diterpenoids | P05186 | 0.104336352 | 9 |
| 1034 | Dihydrotanshinone I (15,16-Dihydrotanshinone I) | Diterpenoids | P08172 | 0.117318329 | 18 |
| 1035 | Dihydrotanshinone I (15,16-Dihydrotanshinone I) | Diterpenoids | P08246 | 0.10362417 | 7 |
| 1036 | Dihydrotanshinone I (15,16-Dihydrotanshinone I) | Diterpenoids | P08575 | 0.109148612 | 27 |
| 1037 | Dihydrotanshinone I (15,16-Dihydrotanshinone I) | Diterpenoids | P08908 | 0.10562303 | 4 |
| 1038 | Dihydrotanshinone I (15,16-Dihydrotanshinone I) | Diterpenoids | P0DMS8 | 0.10464569 | 23 |
| 1039 | Dihydrotanshinone I (15,16-Dihydrotanshinone I) | Diterpenoids | P11229 | 0.103603883 | 11 |
| 1040 | Dihydrotanshinone I (15,16-Dihydrotanshinone I) | Diterpenoids | P11388 | 0.106757964 | 8 |
| 1041 | Dihydrotanshinone I (15,16-Dihydrotanshinone I) | Diterpenoids | P14902 | 0.110454469 | 24 |
| 1042 | Dihydrotanshinone I (15,16-Dihydrotanshinone I) | Diterpenoids | P15121 | 0.267155221 | 39 |
| 1043 | Dihydrotanshinone I (15,16-Dihydrotanshinone I) | Diterpenoids | P16050 | 0.105228934 | 6 |
| 1044 | Dihydrotanshinone I (15,16-Dihydrotanshinone I) | Diterpenoids | P18031 | 0.474772974 | 23 |
| 1045 | Dihydrotanshinone I (15,16-Dihydrotanshinone I) | Diterpenoids | P21980 | 0.101957459 | 8 |
| 1046 | Dihydrotanshinone I (15,16-Dihydrotanshinone I) | Diterpenoids | P22303 | 0.287353637 | 26 |
| 1047 | Dihydrotanshinone I (15,16-Dihydrotanshinone I) | Diterpenoids | P23141 | 0.275271523 | 31 |
| 1048 | Dihydrotanshinone I (15,16-Dihydrotanshinone I) | Diterpenoids | P24864 P24941 | 0.102540734 | 7 |
| 1049 | Dihydrotanshinone I (15,16-Dihydrotanshinone I) | Diterpenoids | P28335 | 0.104521595 | 9 |
| 1050 | Dihydrotanshinone I (15,16-Dihydrotanshinone I) | Diterpenoids | P28472 P18507 P14867 | 0.103855577 | 16 |
| 1051 | Dihydrotanshinone I (15,16-Dihydrotanshinone I) | Diterpenoids | P28472 P18507 P31644 | 0.103791839 | 19 |
| 1052 | Dihydrotanshinone I (15,16-Dihydrotanshinone I) | Diterpenoids | P28472 P34903 P18507 | 0.103855577 | 16 |
| 1053 | Dihydrotanshinone I (15,16-Dihydrotanshinone I) | Diterpenoids | P28562 | 0.104003317 | 12 |
| 1054 | Dihydrotanshinone I (15,16-Dihydrotanshinone I) | Diterpenoids | P29274 | 0.103202685 | 27 |
| 1055 | Dihydrotanshinone I (15,16-Dihydrotanshinone I) | Diterpenoids | P29275 | 0.103333221 | 16 |
| 1056 | Dihydrotanshinone I (15,16-Dihydrotanshinone I) | Diterpenoids | P29350 | 0.204438393 | 44 |
| 1057 | Dihydrotanshinone I (15,16-Dihydrotanshinone I) | Diterpenoids | P30305 | 0.185734372 | 40 |
| 1058 | Dihydrotanshinone I (15,16-Dihydrotanshinone I) | Diterpenoids | P30542 | 0.101318267 | 24 |
| 1059 | Dihydrotanshinone I (15,16-Dihydrotanshinone I) | Diterpenoids | P34969 | 0.102205463 | 3 |
| 1060 | Dihydrotanshinone I (15,16-Dihydrotanshinone I) | Diterpenoids | P35557 | 0.102633551 | 4 |
| 1061 | Dihydrotanshinone I (15,16-Dihydrotanshinone I) | Diterpenoids | P35968 | 0.105052994 | 26 |
| 1062 | Dihydrotanshinone I (15,16-Dihydrotanshinone I) | Diterpenoids | P40763 | 0.268996335 | 13 |
| 1063 | Dihydrotanshinone I (15,16-Dihydrotanshinone I) | Diterpenoids | P41594 | 0.103693674 | 22 |
| 1064 | Dihydrotanshinone I (15,16-Dihydrotanshinone I) | Diterpenoids | P41595 | 0.102913571 | 7 |
| 1065 | Dihydrotanshinone I (15,16-Dihydrotanshinone I) | Diterpenoids | P42574 | 0.10400953 | 13 |
| 1066 | Dihydrotanshinone I (15,16-Dihydrotanshinone I) | Diterpenoids | P45983 | 0.105088123 | 17 |
| 1067 | Dihydrotanshinone I (15,16-Dihydrotanshinone I) | Diterpenoids | P46098 | 0.103859168 | 6 |
| 1068 | Dihydrotanshinone I (15,16-Dihydrotanshinone I) | Diterpenoids | P53779 | 0.103177433 | 9 |
| 1069 | Dihydrotanshinone I (15,16-Dihydrotanshinone I) | Diterpenoids | P55210 | 0.10400953 | 13 |
| 1070 | Dihydrotanshinone I (15,16-Dihydrotanshinone I) | Diterpenoids | P78536 | 0.104291862 | 9 |
| 1071 | Dihydrotanshinone I (15,16-Dihydrotanshinone I) | Diterpenoids | Q02083 | 0.102416978 | 6 |
| 1072 | Dihydrotanshinone I (15,16-Dihydrotanshinone I) | Diterpenoids | Q06124 | 0.211032909 | 40 |
| 1073 | Dihydrotanshinone I (15,16-Dihydrotanshinone I) | Diterpenoids | Q13255 | 0.103848909 | 9 |
| 1074 | Dihydrotanshinone I (15,16-Dihydrotanshinone I) | Diterpenoids | Q15078 Q00535 | 0.102910354 | 14 |
| 1075 | Dihydrotanshinone I (15,16-Dihydrotanshinone I) | Diterpenoids | Q16790 | 0.103433429 | 9 |
| 1076 | Dihydrotanshinone I (15,16-Dihydrotanshinone I) | Diterpenoids | Q8IXJ6 | 0.108888011 | 5 |
| 1077 | Dihydrotanshinone I (15,16-Dihydrotanshinone I) | Diterpenoids | Q92731 | 0.117586956 | 23 |
| 1078 | Dihydrotanshinone I (15,16-Dihydrotanshinone I) | Diterpenoids | Q9UDY8 | 0.107158923 | 10 |
| 1079 | Dihydrotanshinone I (15,16-Dihydrotanshinone I) | Diterpenoids | Q9Y5N1 | 0.101047212 | 6 |
| 1080 | isocryptotanshinone(isodihydrotanshinone IIA) | Diterpenoids | O00519 | 0.110352709 | 16 |
| 1081 | isocryptotanshinone(isodihydrotanshinone IIA) | Diterpenoids | P09874 | 0.106818993 | 18 |
| 1082 | isocryptotanshinone(isodihydrotanshinone IIA) | Diterpenoids | P14902 | 0.110454469 | 24 |
| 1083 | isocryptotanshinone(isodihydrotanshinone IIA) | Diterpenoids | P15121 | 0.267155221 | 39 |
| 1084 | isocryptotanshinone(isodihydrotanshinone IIA) | Diterpenoids | P21980 | 0.101957459 | 8 |
| 1085 | isocryptotanshinone(isodihydrotanshinone IIA) | Diterpenoids | P23219 | 0.109524644 | 16 |
| 1086 | isocryptotanshinone(isodihydrotanshinone IIA) | Diterpenoids | P28845 | 0.253303956 | 34 |
| 1087 | isocryptotanshinone(isodihydrotanshinone IIA) | Diterpenoids | P34913 | 0.106959422 | 6 |
| 1088 | isocryptotanshinone(isodihydrotanshinone IIA) | Diterpenoids | P34972 | 0.103169308 | 13 |
| 1089 | isocryptotanshinone(isodihydrotanshinone IIA) | Diterpenoids | P35354 | 0.113487837 | 23 |
| 1090 | isocryptotanshinone(isodihydrotanshinone IIA) | Diterpenoids | P41594 | 0.103693674 | 22 |
| 1091 | isocryptotanshinone(isodihydrotanshinone IIA) | Diterpenoids | P43235 | 0.104158358 | 7 |
| 1092 | isocryptotanshinone(isodihydrotanshinone IIA) | Diterpenoids | P48147 | 0.107233003 | 22 |
| 1093 | isocryptotanshinone(isodihydrotanshinone IIA) | Diterpenoids | Q01959 | 0.107731401 | 12 |
| 1094 | isocryptotanshinone(isodihydrotanshinone IIA) | Diterpenoids | Q12884 | 0.102934441 | 6 |
| 1095 | isocryptotanshinone(isodihydrotanshinone IIA) | Diterpenoids | Q13255 | 0.103848909 | 9 |
| 1096 | isocryptotanshinone(isodihydrotanshinone IIA) | Diterpenoids | Q8IXJ6 | 0.108888011 | 5 |
| 1097 | isocryptotanshinone(isodihydrotanshinone IIA) | Diterpenoids | Q99572 | 0.10437669 | 25 |
| 1098 | isocryptotanshinone(isodihydrotanshinone IIA) | Diterpenoids | Q9UNQ0 | 0.106050933 | 5 |
| 1099 | isotanshinone I | Diterpenoids | O00311 | 0.105117399 | 6 |
| 1100 | isotanshinone I | Diterpenoids | O00748 | 0.259386615 | 47 |
| 1101 | isotanshinone I | Diterpenoids | O75530 Q15022 Q15910 | 0.341964133 | 19 |
| 1102 | isotanshinone I | Diterpenoids | P07099 | 0.102416978 | 3 |
| 1103 | isotanshinone I | Diterpenoids | P09917 | 0.106782208 | 14 |
| 1104 | isotanshinone I | Diterpenoids | P11309 | 0.104805981 | 13 |
| 1105 | isotanshinone I | Diterpenoids | P21397 | 0.102816353 | 15 |
| 1106 | isotanshinone I | Diterpenoids | P22736 | 0.100039999 | 6 |
| 1107 | isotanshinone I | Diterpenoids | P25025 | 0.100320282 | 13 |
| 1108 | isotanshinone I | Diterpenoids | P27338 | 0.105021471 | 16 |
| 1109 | isotanshinone I | Diterpenoids | P28845 | 0.253303956 | 34 |
| 1110 | isotanshinone I | Diterpenoids | P29274 | 0.103202685 | 27 |
| 1111 | isotanshinone I | Diterpenoids | P29350 | 0.204438393 | 44 |
| 1112 | isotanshinone I | Diterpenoids | P30542 | 0.101318267 | 24 |
| 1113 | isotanshinone I | Diterpenoids | P33261 | 0.124903734 | 16 |
| 1114 | isotanshinone I | Diterpenoids | P35354 | 0.113487837 | 23 |
| 1115 | isotanshinone I | Diterpenoids | Q06124 | 0.211032909 | 40 |
| 1116 | isotanshinone I | Diterpenoids | Q86V86 | 0.100967138 | 7 |
| 1117 | isotanshinone I | Diterpenoids | Q92731 | 0.117586956 | 23 |
| 1118 | isotanshinone I | Diterpenoids | Q99572 | 0.10437669 | 25 |
| 1119 | isotanshinone I | Diterpenoids | Q9P1W9 | 0.103344117 | 12 |
| 1120 | isotanshinone II | Diterpenoids | O00748 | 0.259386615 | 47 |
| 1121 | isotanshinone II | Diterpenoids | O14746 | 0.14728518 | 27 |
| 1122 | isotanshinone II | Diterpenoids | O43570 | 0.137972322 | 10 |
| 1123 | isotanshinone II | Diterpenoids | O43613 | 0.10159278 | 9 |
| 1124 | isotanshinone II | Diterpenoids | O43614 | 0.100916735 | 11 |
| 1125 | isotanshinone II | Diterpenoids | O75530 Q15022 Q15910 | 0.341964133 | 19 |
| 1126 | isotanshinone II | Diterpenoids | O75530 Q15022 Q15910 | 0.341964133 | 19 |
| 1127 | isotanshinone II | Diterpenoids | O96020 P24941 P24864 | 0.104035866 | 9 |
| 1128 | isotanshinone II | Diterpenoids | P00533 | 0.104151342 | 24 |
| 1129 | isotanshinone II | Diterpenoids | P00915 | 0.106312298 | 8 |
| 1130 | isotanshinone II | Diterpenoids | P00918 | 0.141292356 | 8 |
| 1131 | isotanshinone II | Diterpenoids | P07099 | 0.102416978 | 3 |
| 1132 | isotanshinone II | Diterpenoids | P08575 | 0.109148612 | 27 |
| 1133 | isotanshinone II | Diterpenoids | P0DMS8 | 0.10464569 | 23 |
| 1134 | isotanshinone II | Diterpenoids | P10275 | 0.132131799 | 34 |
| 1135 | isotanshinone II | Diterpenoids | P11511 | 0.139056613 | 29 |
| 1136 | isotanshinone II | Diterpenoids | P11940 | 0.1046882 | 6 |
| 1137 | isotanshinone II | Diterpenoids | P12268 | 0.104524224 | 13 |
| 1138 | isotanshinone II | Diterpenoids | P12268 | 0.104524224 | 13 |
| 1139 | isotanshinone II | Diterpenoids | P15121 | 0.267155221 | 39 |
| 1140 | isotanshinone II | Diterpenoids | P16083 | 0.112425971 | 14 |
| 1141 | isotanshinone II | Diterpenoids | P21397 | 0.102816353 | 15 |
| 1142 | isotanshinone II | Diterpenoids | P23141 | 0.275271523 | 31 |
| 1143 | isotanshinone II | Diterpenoids | P25025 | 0.100320282 | 13 |
| 1144 | isotanshinone II | Diterpenoids | P25025 | 0.100320282 | 13 |
| 1145 | isotanshinone II | Diterpenoids | P27338 | 0.105021471 | 16 |
| 1146 | isotanshinone II | Diterpenoids | P27338 | 0.105021471 | 16 |
| 1147 | isotanshinone II | Diterpenoids | P28845 | 0.253303956 | 34 |
| 1148 | isotanshinone II | Diterpenoids | P29274 | 0.103202685 | 27 |
| 1149 | isotanshinone II | Diterpenoids | P29275 | 0.103333221 | 16 |
| 1150 | isotanshinone II | Diterpenoids | P29350 | 0.204438393 | 44 |
| 1151 | isotanshinone II | Diterpenoids | P30304 | 0.109145376 | 25 |
| 1152 | isotanshinone II | Diterpenoids | P30305 | 0.185734372 | 40 |
| 1153 | isotanshinone II | Diterpenoids | P30307 | 0.105546712 | 12 |
| 1154 | isotanshinone II | Diterpenoids | P30542 | 0.101318267 | 24 |
| 1155 | isotanshinone II | Diterpenoids | P31644 | 0.101037512 | 7 |
| 1156 | isotanshinone II | Diterpenoids | P31645 | 0.1201852 | 17 |
| 1157 | isotanshinone II | Diterpenoids | P33261 | 0.124903734 | 16 |
| 1158 | isotanshinone II | Diterpenoids | P34913 | 0.106959422 | 6 |
| 1159 | isotanshinone II | Diterpenoids | P34949 | 0.105190036 | 9 |
| 1160 | isotanshinone II | Diterpenoids | P35968 | 0.105052994 | 26 |
| 1161 | isotanshinone II | Diterpenoids | P42336 | 0.108042586 | 13 |
| 1162 | isotanshinone II | Diterpenoids | P48039 | 0.101354935 | 19 |
| 1163 | isotanshinone II | Diterpenoids | P49286 | 0.101354935 | 19 |
| 1164 | isotanshinone II | Diterpenoids | P49354 P49356 | 0.109904857 | 24 |
| 1165 | isotanshinone II | Diterpenoids | P54760 | 0.111687884 | 3 |
| 1166 | isotanshinone II | Diterpenoids | Q01959 | 0.107731401 | 12 |
| 1167 | isotanshinone II | Diterpenoids | Q06124 | 0.211032909 | 40 |
| 1168 | isotanshinone II | Diterpenoids | Q09028 Q16576 O75530 Q15022 Q15910 | 0.142197972 | 14 |
| 1169 | isotanshinone II | Diterpenoids | Q13936 | 0.102416978 | 3 |
| 1170 | isotanshinone II | Diterpenoids | Q16790 | 0.103433429 | 9 |
| 1171 | isotanshinone II | Diterpenoids | Q8IXJ6 | 0.108888011 | 5 |
| 1172 | isotanshinone II | Diterpenoids | Q8WWL7 P06493 P14635 O95067 | 0.10231122 | 6 |
| 1173 | isotanshinone II | Diterpenoids | Q92793 | 0.102416978 | 3 |
| 1174 | isotanshinone II | Diterpenoids | Q99572 | 0.10437669 | 25 |
| 1175 | isotanshinone II | Diterpenoids | Q99572 | 0.10437669 | 25 |
| 1176 | isotanshinone IIA | Diterpenoids | O75530 Q15022 Q15910 | 0.341964133 | 19 |
| 1177 | isotanshinone IIA | Diterpenoids | P11940 | 0.1046882 | 6 |
| 1178 | isotanshinone IIA | Diterpenoids | P12268 | 0.104524224 | 13 |
| 1179 | isotanshinone IIA | Diterpenoids | P16083 | 0.112425971 | 14 |
| 1180 | isotanshinone IIA | Diterpenoids | P25025 | 0.100320282 | 13 |
| 1181 | isotanshinone IIA | Diterpenoids | P27338 | 0.105021471 | 16 |
| 1182 | isotanshinone IIA | Diterpenoids | P33261 | 0.124903734 | 16 |
| 1183 | isotanshinone IIA | Diterpenoids | P34913 | 0.106959422 | 6 |
| 1184 | isotanshinone IIA | Diterpenoids | P42336 | 0.108042586 | 13 |
| 1185 | isotanshinone IIA | Diterpenoids | P49354 P49356 | 0.109904857 | 24 |
| 1186 | isotanshinone IIA | Diterpenoids | Q99572 | 0.10437669 | 25 |
| 1187 | isotanshinone IIB (18-hydroxyisotanshinone IIA) | Diterpenoids | O00311 | 0.105117399 | 6 |
| 1188 | isotanshinone IIB (18-hydroxyisotanshinone IIA) | Diterpenoids | O00329 | 0.105574288 | 6 |
| 1189 | isotanshinone IIB (18-hydroxyisotanshinone IIA) | Diterpenoids | O00408 | 0.103882897 | 9 |
| 1190 | isotanshinone IIB (18-hydroxyisotanshinone IIA) | Diterpenoids | O00748 | 0.259386615 | 47 |
| 1191 | isotanshinone IIB (18-hydroxyisotanshinone IIA) | Diterpenoids | O14649 | 0.100520388 | 4 |
| 1192 | isotanshinone IIB (18-hydroxyisotanshinone IIA) | Diterpenoids | O14757 | 0.106739614 | 8 |
| 1193 | isotanshinone IIB (18-hydroxyisotanshinone IIA) | Diterpenoids | O14965 | 0.107316885 | 8 |
| 1194 | isotanshinone IIB (18-hydroxyisotanshinone IIA) | Diterpenoids | O43613 | 0.10159278 | 9 |
| 1195 | isotanshinone IIB (18-hydroxyisotanshinone IIA) | Diterpenoids | O43614 | 0.100916735 | 11 |
| 1196 | isotanshinone IIB (18-hydroxyisotanshinone IIA) | Diterpenoids | O60674 | 0.103305945 | 11 |
| 1197 | isotanshinone IIB (18-hydroxyisotanshinone IIA) | Diterpenoids | O60706 | 0.103819513 | 5 |
| 1198 | isotanshinone IIB (18-hydroxyisotanshinone IIA) | Diterpenoids | P00746 | 0.101536397 | 5 |
| 1199 | isotanshinone IIB (18-hydroxyisotanshinone IIA) | Diterpenoids | P04035 | 0.166864971 | 20 |
| 1200 | isotanshinone IIB (18-hydroxyisotanshinone IIA) | Diterpenoids | P04150 | 0.109500234 | 17 |
| 1201 | isotanshinone IIB (18-hydroxyisotanshinone IIA) | Diterpenoids | P06239 | 0.102843593 | 5 |
| 1202 | isotanshinone IIB (18-hydroxyisotanshinone IIA) | Diterpenoids | P06401 | 0.108868706 | 15 |
| 1203 | isotanshinone IIB (18-hydroxyisotanshinone IIA) | Diterpenoids | P06493 | 0.102732601 | 7 |
| 1204 | isotanshinone IIB (18-hydroxyisotanshinone IIA) | Diterpenoids | P06737 | 0.103331553 | 10 |
| 1205 | isotanshinone IIB (18-hydroxyisotanshinone IIA) | Diterpenoids | P07947 | 0.101613855 | 3 |
| 1206 | isotanshinone IIB (18-hydroxyisotanshinone IIA) | Diterpenoids | P08235 | 0.107998327 | 12 |
| 1207 | isotanshinone IIB (18-hydroxyisotanshinone IIA) | Diterpenoids | P08684 | 0.104909858 | 3 |
| 1208 | isotanshinone IIB (18-hydroxyisotanshinone IIA) | Diterpenoids | P08908 | 0.10562303 | 4 |
| 1209 | isotanshinone IIB (18-hydroxyisotanshinone IIA) | Diterpenoids | P09211 | 0.100367415 | 3 |
| 1210 | isotanshinone IIB (18-hydroxyisotanshinone IIA) | Diterpenoids | P09874 | 0.106818993 | 18 |
| 1211 | isotanshinone IIB (18-hydroxyisotanshinone IIA) | Diterpenoids | P10275 | 0.132131799 | 34 |
| 1212 | isotanshinone IIB (18-hydroxyisotanshinone IIA) | Diterpenoids | P11309 | 0.104805981 | 13 |
| 1213 | isotanshinone IIB (18-hydroxyisotanshinone IIA) | Diterpenoids | P12268 | 0.104524224 | 13 |
| 1214 | isotanshinone IIB (18-hydroxyisotanshinone IIA) | Diterpenoids | P17252 | 0.102754312 | 7 |
| 1215 | isotanshinone IIB (18-hydroxyisotanshinone IIA) | Diterpenoids | P21554 | 0.104626059 | 10 |
| 1216 | isotanshinone IIB (18-hydroxyisotanshinone IIA) | Diterpenoids | P23219 | 0.109524644 | 16 |
| 1217 | isotanshinone IIB (18-hydroxyisotanshinone IIA) | Diterpenoids | P23458 | 0.102916912 | 11 |
| 1218 | isotanshinone IIB (18-hydroxyisotanshinone IIA) | Diterpenoids | P24941 | 0.106080884 | 8 |
| 1219 | isotanshinone IIB (18-hydroxyisotanshinone IIA) | Diterpenoids | P25025 | 0.100320282 | 13 |
| 1220 | isotanshinone IIB (18-hydroxyisotanshinone IIA) | Diterpenoids | P25101 | 0.104008389 | 6 |
| 1221 | isotanshinone IIB (18-hydroxyisotanshinone IIA) | Diterpenoids | P28221 | 0.104909858 | 3 |
| 1222 | isotanshinone IIB (18-hydroxyisotanshinone IIA) | Diterpenoids | P28472 P18507 P14867 | 0.103855577 | 16 |
| 1223 | isotanshinone IIB (18-hydroxyisotanshinone IIA) | Diterpenoids | P28472 P18507 P31644 | 0.103791839 | 19 |
| 1224 | isotanshinone IIB (18-hydroxyisotanshinone IIA) | Diterpenoids | P28472 P34903 P18507 | 0.103855577 | 16 |
| 1225 | isotanshinone IIB (18-hydroxyisotanshinone IIA) | Diterpenoids | P28845 | 0.253303956 | 34 |
| 1226 | isotanshinone IIB (18-hydroxyisotanshinone IIA) | Diterpenoids | P29274 | 0.103202685 | 27 |
| 1227 | isotanshinone IIB (18-hydroxyisotanshinone IIA) | Diterpenoids | P29275 | 0.103333221 | 16 |
| 1228 | isotanshinone IIB (18-hydroxyisotanshinone IIA) | Diterpenoids | P29371 | 0.103946422 | 5 |
| 1229 | isotanshinone IIB (18-hydroxyisotanshinone IIA) | Diterpenoids | P29466 | 0.105464394 | 4 |
| 1230 | isotanshinone IIB (18-hydroxyisotanshinone IIA) | Diterpenoids | P30542 | 0.101318267 | 24 |
| 1231 | isotanshinone IIB (18-hydroxyisotanshinone IIA) | Diterpenoids | P31639 | 0.109951316 | 5 |
| 1232 | isotanshinone IIB (18-hydroxyisotanshinone IIA) | Diterpenoids | P31644 | 0.101037512 | 7 |
| 1233 | isotanshinone IIB (18-hydroxyisotanshinone IIA) | Diterpenoids | P32246 | 0.109647095 | 6 |
| 1234 | isotanshinone IIB (18-hydroxyisotanshinone IIA) | Diterpenoids | P33261 | 0.124903734 | 16 |
| 1235 | isotanshinone IIB (18-hydroxyisotanshinone IIA) | Diterpenoids | P34972 | 0.103169308 | 13 |
| 1236 | isotanshinone IIB (18-hydroxyisotanshinone IIA) | Diterpenoids | P35354 | 0.113487837 | 23 |
| 1237 | isotanshinone IIB (18-hydroxyisotanshinone IIA) | Diterpenoids | P41594 | 0.103693674 | 22 |
| 1238 | isotanshinone IIB (18-hydroxyisotanshinone IIA) | Diterpenoids | P42330 | 0.105243819 | 5 |
| 1239 | isotanshinone IIB (18-hydroxyisotanshinone IIA) | Diterpenoids | P42336 | 0.108042586 | 13 |
| 1240 | isotanshinone IIB (18-hydroxyisotanshinone IIA) | Diterpenoids | P42338 | 0.107541648 | 9 |
| 1241 | isotanshinone IIB (18-hydroxyisotanshinone IIA) | Diterpenoids | P42345 | 0.10907142 | 6 |
| 1242 | isotanshinone IIB (18-hydroxyisotanshinone IIA) | Diterpenoids | P42574 | 0.10400953 | 13 |
| 1243 | isotanshinone IIB (18-hydroxyisotanshinone IIA) | Diterpenoids | P43235 | 0.104158358 | 7 |
| 1244 | isotanshinone IIB (18-hydroxyisotanshinone IIA) | Diterpenoids | P47869 P28472 P18507 | 0.104254314 | 15 |
| 1245 | isotanshinone IIB (18-hydroxyisotanshinone IIA) | Diterpenoids | P48039 | 0.101354935 | 19 |
| 1246 | isotanshinone IIB (18-hydroxyisotanshinone IIA) | Diterpenoids | P48147 | 0.107233003 | 22 |
| 1247 | isotanshinone IIB (18-hydroxyisotanshinone IIA) | Diterpenoids | P49137 | 0.107101597 | 6 |
| 1248 | isotanshinone IIB (18-hydroxyisotanshinone IIA) | Diterpenoids | P49286 | 0.101354935 | 19 |
| 1249 | isotanshinone IIB (18-hydroxyisotanshinone IIA) | Diterpenoids | P49354 P49356 | 0.109904857 | 24 |
| 1250 | isotanshinone IIB (18-hydroxyisotanshinone IIA) | Diterpenoids | P49841 | 0.105823985 | 11 |
| 1251 | isotanshinone IIB (18-hydroxyisotanshinone IIA) | Diterpenoids | P51812 | 0.135853345 | 4 |
| 1252 | isotanshinone IIB (18-hydroxyisotanshinone IIA) | Diterpenoids | P52333 | 0.104243408 | 12 |
| 1253 | isotanshinone IIB (18-hydroxyisotanshinone IIA) | Diterpenoids | P52732 | 0.104747724 | 9 |
| 1254 | isotanshinone IIB (18-hydroxyisotanshinone IIA) | Diterpenoids | P53609 P49354 | 0.101613855 | 3 |
| 1255 | isotanshinone IIB (18-hydroxyisotanshinone IIA) | Diterpenoids | P55210 | 0.10400953 | 13 |
| 1256 | isotanshinone IIB (18-hydroxyisotanshinone IIA) | Diterpenoids | P56373 | 0.107098941 | 5 |
| 1257 | isotanshinone IIB (18-hydroxyisotanshinone IIA) | Diterpenoids | P56817 | 0.108325485 | 12 |
| 1258 | isotanshinone IIB (18-hydroxyisotanshinone IIA) | Diterpenoids | P78527 | 0.104180881 | 6 |
| 1259 | isotanshinone IIB (18-hydroxyisotanshinone IIA) | Diterpenoids | Q00987 | 0.107095673 | 11 |
| 1260 | isotanshinone IIB (18-hydroxyisotanshinone IIA) | Diterpenoids | Q02750 | 0.106463868 | 11 |
| 1261 | isotanshinone IIB (18-hydroxyisotanshinone IIA) | Diterpenoids | Q08828 | 0.102451863 | 4 |
| 1262 | isotanshinone IIB (18-hydroxyisotanshinone IIA) | Diterpenoids | Q12866 | 0.103756913 | 4 |
| 1263 | isotanshinone IIB (18-hydroxyisotanshinone IIA) | Diterpenoids | Q12884 | 0.102934441 | 6 |
| 1264 | isotanshinone IIB (18-hydroxyisotanshinone IIA) | Diterpenoids | Q13255 | 0.103848909 | 9 |
| 1265 | isotanshinone IIB (18-hydroxyisotanshinone IIA) | Diterpenoids | Q13946 | 0.104692547 | 7 |
| 1266 | isotanshinone IIB (18-hydroxyisotanshinone IIA) | Diterpenoids | Q14790 | 0.104694286 | 5 |
| 1267 | isotanshinone IIB (18-hydroxyisotanshinone IIA) | Diterpenoids | Q15078 Q00535 | 0.102910354 | 14 |
| 1268 | isotanshinone IIB (18-hydroxyisotanshinone IIA) | Diterpenoids | Q15761 | 0.10562303 | 4 |
| 1269 | isotanshinone IIB (18-hydroxyisotanshinone IIA) | Diterpenoids | Q5S007 | 0.102368749 | 9 |
| 1270 | isotanshinone IIB (18-hydroxyisotanshinone IIA) | Diterpenoids | Q86V86 | 0.100967138 | 7 |
| 1271 | isotanshinone IIB (18-hydroxyisotanshinone IIA) | Diterpenoids | Q99572 | 0.10437669 | 25 |
| 1272 | isotanshinone IIB (18-hydroxyisotanshinone IIA) | Diterpenoids | Q9H4B7 | 0.103451903 | 3 |
| 1273 | isotanshinone IIB (18-hydroxyisotanshinone IIA) | Diterpenoids | Q9NWZ3 | 0.105136637 | 5 |
| 1274 | isotanshinone IIB (18-hydroxyisotanshinone IIA) | Diterpenoids | Q9NZJ5 | 0.102919059 | 6 |
| 1275 | isotanshinone IIB (18-hydroxyisotanshinone IIA) | Diterpenoids | Q9P1W9 | 0.103344117 | 12 |
| 1276 | isotanshinone IIB (18-hydroxyisotanshinone IIA) | Diterpenoids | Q9Y233 | 0.108085897 | 14 |
| 1277 | methyl tanshinonate (methyltanshinoate) | Diterpenoids | O00748 | 0.259386615 | 47 |
| 1278 | methyl tanshinonate (methyltanshinoate) | Diterpenoids | O14684 | 0.116389783 | 19 |
| 1279 | methyl tanshinonate (methyltanshinoate) | Diterpenoids | O14746 | 0.14728518 | 27 |
| 1280 | methyl tanshinonate (methyltanshinoate) | Diterpenoids | O43613 | 0.10159278 | 9 |
| 1281 | methyl tanshinonate (methyltanshinoate) | Diterpenoids | O43614 | 0.100916735 | 11 |
| 1282 | methyl tanshinonate (methyltanshinoate) | Diterpenoids | O60885 | 0.104476685 | 6 |
| 1283 | methyl tanshinonate (methyltanshinoate) | Diterpenoids | O75530 Q15022 Q15910 | 0.341964133 | 19 |
| 1284 | methyl tanshinonate (methyltanshinoate) | Diterpenoids | P04150 | 0.109500234 | 17 |
| 1285 | methyl tanshinonate (methyltanshinoate) | Diterpenoids | P06276 | 0.130116287 | 14 |
| 1286 | methyl tanshinonate (methyltanshinoate) | Diterpenoids | P06401 | 0.108868706 | 15 |
| 1287 | methyl tanshinonate (methyltanshinoate) | Diterpenoids | P08581 | 0.109668623 | 6 |
| 1288 | methyl tanshinonate (methyltanshinoate) | Diterpenoids | P08842 | 0.103451903 | 3 |
| 1289 | methyl tanshinonate (methyltanshinoate) | Diterpenoids | P09874 | 0.106818993 | 18 |
| 1290 | methyl tanshinonate (methyltanshinoate) | Diterpenoids | P0DMS8 | 0.10464569 | 23 |
| 1291 | methyl tanshinonate (methyltanshinoate) | Diterpenoids | P11511 | 0.139056613 | 29 |
| 1292 | methyl tanshinonate (methyltanshinoate) | Diterpenoids | P12268 | 0.104524224 | 13 |
| 1293 | methyl tanshinonate (methyltanshinoate) | Diterpenoids | P15121 | 0.267155221 | 39 |
| 1294 | methyl tanshinonate (methyltanshinoate) | Diterpenoids | P16083 | 0.112425971 | 14 |
| 1295 | methyl tanshinonate (methyltanshinoate) | Diterpenoids | P21397 | 0.102816353 | 15 |
| 1296 | methyl tanshinonate (methyltanshinoate) | Diterpenoids | P22303 | 0.287353637 | 26 |
| 1297 | methyl tanshinonate (methyltanshinoate) | Diterpenoids | P22736 | 0.100039999 | 6 |
| 1298 | methyl tanshinonate (methyltanshinoate) | Diterpenoids | P23141 | 0.275271523 | 31 |
| 1299 | methyl tanshinonate (methyltanshinoate) | Diterpenoids | P25025 | 0.100320282 | 13 |
| 1300 | methyl tanshinonate (methyltanshinoate) | Diterpenoids | P25774 | 0.106840916 | 6 |
| 1301 | methyl tanshinonate (methyltanshinoate) | Diterpenoids | P28335 | 0.104521595 | 9 |
| 1302 | methyl tanshinonate (methyltanshinoate) | Diterpenoids | P28472 P18507 P31644 | 0.103791839 | 19 |
| 1303 | methyl tanshinonate (methyltanshinoate) | Diterpenoids | P29274 | 0.103202685 | 27 |
| 1304 | methyl tanshinonate (methyltanshinoate) | Diterpenoids | P29275 | 0.103333221 | 16 |
| 1305 | methyl tanshinonate (methyltanshinoate) | Diterpenoids | P29350 | 0.204438393 | 44 |
| 1306 | methyl tanshinonate (methyltanshinoate) | Diterpenoids | P29371 | 0.103946422 | 5 |
| 1307 | methyl tanshinonate (methyltanshinoate) | Diterpenoids | P30304 | 0.109145376 | 25 |
| 1308 | methyl tanshinonate (methyltanshinoate) | Diterpenoids | P30305 | 0.185734372 | 40 |
| 1309 | methyl tanshinonate (methyltanshinoate) | Diterpenoids | P30542 | 0.101318267 | 24 |
| 1310 | methyl tanshinonate (methyltanshinoate) | Diterpenoids | P32247 | 0.103071649 | 5 |
| 1311 | methyl tanshinonate (methyltanshinoate) | Diterpenoids | P34949 | 0.105190036 | 9 |
| 1312 | methyl tanshinonate (methyltanshinoate) | Diterpenoids | P41594 | 0.103693674 | 22 |
| 1313 | methyl tanshinonate (methyltanshinoate) | Diterpenoids | P41595 | 0.102913571 | 7 |
| 1314 | methyl tanshinonate (methyltanshinoate) | Diterpenoids | P43115 | 0.11287548 | 5 |
| 1315 | methyl tanshinonate (methyltanshinoate) | Diterpenoids | P45983 | 0.105088123 | 17 |
| 1316 | methyl tanshinonate (methyltanshinoate) | Diterpenoids | P48039 | 0.101354935 | 19 |
| 1317 | methyl tanshinonate (methyltanshinoate) | Diterpenoids | P48147 | 0.107233003 | 22 |
| 1318 | methyl tanshinonate (methyltanshinoate) | Diterpenoids | P49286 | 0.101354935 | 19 |
| 1319 | methyl tanshinonate (methyltanshinoate) | Diterpenoids | P53779 | 0.103177433 | 9 |
| 1320 | methyl tanshinonate (methyltanshinoate) | Diterpenoids | Q06124 | 0.211032909 | 40 |
| 1321 | methyl tanshinonate (methyltanshinoate) | Diterpenoids | Q09028 Q16576 O75530 Q15022 Q15910 | 0.142197972 | 14 |
| 1322 | methyl tanshinonate (methyltanshinoate) | Diterpenoids | Q16539 | 0.105438848 | 20 |
| 1323 | methyl tanshinonate (methyltanshinoate) | Diterpenoids | Q99572 | 0.10437669 | 25 |
| 1324 | methyl tanshinonate (methyltanshinoate) | Diterpenoids | Q9NZJ5 | 0.102919059 | 6 |
| 1325 | miltirone | Diterpenoids | O00748 | 0.259386615 | 47 |
| 1326 | miltirone | Diterpenoids | O95271 | 0.102617882 | 7 |
| 1327 | miltirone | Diterpenoids | P08575 | 0.109148612 | 27 |
| 1328 | miltirone | Diterpenoids | P10586 | 0.312345915 | 15 |
| 1329 | miltirone | Diterpenoids | P11387 | 0.113455556 | 13 |
| 1330 | miltirone | Diterpenoids | P15121 | 0.267155221 | 39 |
| 1331 | miltirone | Diterpenoids | P18031 | 0.474772974 | 23 |
| 1332 | miltirone | Diterpenoids | P22303 | 0.287353637 | 26 |
| 1333 | miltirone | Diterpenoids | P22894 | 0.111100851 | 4 |
| 1334 | miltirone | Diterpenoids | P23141 | 0.275271523 | 31 |
| 1335 | miltirone | Diterpenoids | P27338 | 0.105021471 | 16 |
| 1336 | miltirone | Diterpenoids | P30304 | 0.109145376 | 25 |
| 1337 | miltirone | Diterpenoids | P30305 | 0.185734372 | 40 |
| 1338 | miltirone | Diterpenoids | P40763 | 0.268996335 | 13 |
| 1339 | miltirone | Diterpenoids | P41594 | 0.103693674 | 22 |
| 1340 | miltirone | Diterpenoids | P42330 | 0.105243819 | 5 |
| 1341 | miltirone | Diterpenoids | P42336 | 0.108042586 | 13 |
| 1342 | miltirone | Diterpenoids | P45452 | 0.106586322 | 6 |
| 1343 | miltirone | Diterpenoids | P48039 | 0.101354935 | 19 |
| 1344 | miltirone | Diterpenoids | P49286 | 0.101354935 | 19 |
| 1345 | miltirone | Diterpenoids | P51532 | 0.1046882 | 4 |
| 1346 | miltirone | Diterpenoids | P78536 | 0.104291862 | 9 |
| 1347 | miltirone | Diterpenoids | Q86U86 | 0.1046882 | 4 |
| 1348 | miltirone | Diterpenoids | Q9H2K2 | 0.104609573 | 11 |
| 1349 | miltirone | Diterpenoids | Q9Y2R2 | 0.111485907 | 5 |
| 1350 | miltirone I | Diterpenoids | O00748 | 0.259386615 | 47 |
| 1351 | miltirone I | Diterpenoids | O43194 | 0.10421989 | 3 |
| 1352 | miltirone I | Diterpenoids | O43570 | 0.137972322 | 10 |
| 1353 | miltirone I | Diterpenoids | O95271 | 0.102617882 | 7 |
| 1354 | miltirone I | Diterpenoids | P00533 | 0.104151342 | 24 |
| 1355 | miltirone I | Diterpenoids | P04626 | 0.104502793 | 11 |
| 1356 | miltirone I | Diterpenoids | P05362 | 0.105155078 | 5 |
| 1357 | miltirone I | Diterpenoids | P06276 | 0.130116287 | 14 |
| 1358 | miltirone I | Diterpenoids | P07949 | 0.103471701 | 8 |
| 1359 | miltirone I | Diterpenoids | P08172 | 0.117318329 | 18 |
| 1360 | miltirone I | Diterpenoids | P08575 | 0.109148612 | 27 |
| 1361 | miltirone I | Diterpenoids | P0DMS8 | 0.10464569 | 23 |
| 1362 | miltirone I | Diterpenoids | P10586 | 0.312345915 | 15 |
| 1363 | miltirone I | Diterpenoids | P11229 | 0.103603883 | 11 |
| 1364 | miltirone I | Diterpenoids | P11387 | 0.113455556 | 13 |
| 1365 | miltirone I | Diterpenoids | P14867 | 0.10300422 | 7 |
| 1366 | miltirone I | Diterpenoids | P14902 | 0.110454469 | 24 |
| 1367 | miltirone I | Diterpenoids | P15121 | 0.267155221 | 39 |
| 1368 | miltirone I | Diterpenoids | P16581 | 0.105155078 | 5 |
| 1369 | miltirone I | Diterpenoids | P17948 | 0.105554897 | 13 |
| 1370 | miltirone I | Diterpenoids | P18031 | 0.474772974 | 23 |
| 1371 | miltirone I | Diterpenoids | P19320 | 0.105155078 | 5 |
| 1372 | miltirone I | Diterpenoids | P20309 | 0.105165619 | 10 |
| 1373 | miltirone I | Diterpenoids | P21397 | 0.102816353 | 15 |
| 1374 | miltirone I | Diterpenoids | P21980 | 0.101957459 | 8 |
| 1375 | miltirone I | Diterpenoids | P22303 | 0.287353637 | 26 |
| 1376 | miltirone I | Diterpenoids | P23141 | 0.275271523 | 31 |
| 1377 | miltirone I | Diterpenoids | P24863 P49336 | 0.102633551 | 4 |
| 1378 | miltirone I | Diterpenoids | P24864 P24941 | 0.102540734 | 7 |
| 1379 | miltirone I | Diterpenoids | P27338 | 0.105021471 | 16 |
| 1380 | miltirone I | Diterpenoids | P28472 P18507 P14867 | 0.103855577 | 16 |
| 1381 | miltirone I | Diterpenoids | P28472 P18507 P31644 | 0.103791839 | 19 |
| 1382 | miltirone I | Diterpenoids | P28472 P34903 P18507 | 0.103855577 | 16 |
| 1383 | miltirone I | Diterpenoids | P28562 | 0.104003317 | 12 |
| 1384 | miltirone I | Diterpenoids | P28845 | 0.253303956 | 34 |
| 1385 | miltirone I | Diterpenoids | P29274 | 0.103202685 | 27 |
| 1386 | miltirone I | Diterpenoids | P29350 | 0.204438393 | 44 |
| 1387 | miltirone I | Diterpenoids | P30304 | 0.109145376 | 25 |
| 1388 | miltirone I | Diterpenoids | P30305 | 0.185734372 | 40 |
| 1389 | miltirone I | Diterpenoids | P30536 | 0.102095712 | 5 |
| 1390 | miltirone I | Diterpenoids | P30542 | 0.101318267 | 24 |
| 1391 | miltirone I | Diterpenoids | P31644 | 0.101037512 | 7 |
| 1392 | miltirone I | Diterpenoids | P35557 | 0.102633551 | 4 |
| 1393 | miltirone I | Diterpenoids | P35968 | 0.105052994 | 26 |
| 1394 | miltirone I | Diterpenoids | P36888 | 0.102892289 | 8 |
| 1395 | miltirone I | Diterpenoids | P41594 | 0.103693674 | 22 |
| 1396 | miltirone I | Diterpenoids | P41595 | 0.102913571 | 7 |
| 1397 | miltirone I | Diterpenoids | P42574 | 0.10400953 | 13 |
| 1398 | miltirone I | Diterpenoids | P45983 | 0.105088123 | 17 |
| 1399 | miltirone I | Diterpenoids | P46098 | 0.103859168 | 6 |
| 1400 | miltirone I | Diterpenoids | P47869 P28472 P18507 | 0.104254314 | 15 |
| 1401 | miltirone I | Diterpenoids | P55210 | 0.10400953 | 13 |
| 1402 | miltirone I | Diterpenoids | P78536 | 0.104291862 | 9 |
| 1403 | miltirone I | Diterpenoids | Q06124 | 0.211032909 | 40 |
| 1404 | miltirone I | Diterpenoids | Q13255 | 0.103848909 | 9 |
| 1405 | miltirone I | Diterpenoids | Q14833 | 0.102633551 | 4 |
| 1406 | miltirone I | Diterpenoids | Q15078 Q00535 | 0.102910354 | 14 |
| 1407 | miltirone I | Diterpenoids | Q16790 | 0.103433429 | 9 |
| 1408 | miltirone I | Diterpenoids | Q7Z2W7 | 0.106776816 | 5 |
| 1409 | miltirone I | Diterpenoids | Q9H2K2 | 0.104609573 | 11 |
| 1410 | miltirone I | Diterpenoids | Q9Y2R2 | 0.111485907 | 5 |
| 1411 | miltirone I | Diterpenoids | Q9Y5N1 | 0.101047212 | 6 |
| 1412 | monodydroxytanshinone I | Diterpenoids | O00748 | 0.259386615 | 47 |
| 1413 | monodydroxytanshinone I | Diterpenoids | O14746 | 0.14728518 | 27 |
| 1414 | monodydroxytanshinone I | Diterpenoids | O75530 Q15022 Q15910 | 0.341964133 | 19 |
| 1415 | monodydroxytanshinone I | Diterpenoids | P08253 | 0.106581489 | 9 |
| 1416 | monodydroxytanshinone I | Diterpenoids | P08575 | 0.109148612 | 27 |
| 1417 | monodydroxytanshinone I | Diterpenoids | P09874 | 0.106818993 | 18 |
| 1418 | monodydroxytanshinone I | Diterpenoids | P10721 | 0.104909858 | 3 |
| 1419 | monodydroxytanshinone I | Diterpenoids | P15121 | 0.267155221 | 39 |
| 1420 | monodydroxytanshinone I | Diterpenoids | P16050 | 0.105228934 | 6 |
| 1421 | monodydroxytanshinone I | Diterpenoids | P17948 | 0.105554897 | 13 |
| 1422 | monodydroxytanshinone I | Diterpenoids | P23141 | 0.275271523 | 31 |
| 1423 | monodydroxytanshinone I | Diterpenoids | P29350 | 0.204438393 | 44 |
| 1424 | monodydroxytanshinone I | Diterpenoids | P30304 | 0.109145376 | 25 |
| 1425 | monodydroxytanshinone I | Diterpenoids | P30305 | 0.185734372 | 40 |
| 1426 | monodydroxytanshinone I | Diterpenoids | P30307 | 0.105546712 | 12 |
| 1427 | monodydroxytanshinone I | Diterpenoids | P34949 | 0.105190036 | 9 |
| 1428 | monodydroxytanshinone I | Diterpenoids | P49841 | 0.105823985 | 11 |
| 1429 | monodydroxytanshinone I | Diterpenoids | Q06124 | 0.211032909 | 40 |
| 1430 | monodydroxytanshinone I | Diterpenoids | Q09028 Q16576 O75530 Q15022 Q15910 | 0.142197972 | 14 |
| 1431 | monodydroxytanshinone I | Diterpenoids | Q16539 | 0.105438848 | 20 |
| 1432 | monodydroxytanshinone I | Diterpenoids | Q9NZJ5 | 0.102919059 | 6 |
| 1433 | neocryptotanshinone （tanshinone V） | Diterpenoids | O14649 | 0.100520388 | 4 |
| 1434 | neocryptotanshinone （tanshinone V） | Diterpenoids | O14920 | 0.104909858 | 3 |
| 1435 | neocryptotanshinone （tanshinone V） | Diterpenoids | O15530 | 0.110104207 | 4 |
| 1436 | neocryptotanshinone （tanshinone V） | Diterpenoids | O60674 | 0.103305945 | 11 |
| 1437 | neocryptotanshinone （tanshinone V） | Diterpenoids | O75460 | 0.105470076 | 5 |
| 1438 | neocryptotanshinone （tanshinone V） | Diterpenoids | O76074 | 0.103099774 | 12 |
| 1439 | neocryptotanshinone （tanshinone V） | Diterpenoids | O96017 | 0.109638321 | 3 |
| 1440 | neocryptotanshinone （tanshinone V） | Diterpenoids | O96020 P24941 P24864 | 0.104035866 | 9 |
| 1441 | neocryptotanshinone （tanshinone V） | Diterpenoids | P00519 | 0.104821195 | 5 |
| 1442 | neocryptotanshinone （tanshinone V） | Diterpenoids | P00533 | 0.104151342 | 24 |
| 1443 | neocryptotanshinone （tanshinone V） | Diterpenoids | P04629 | 0.107046621 | 8 |
| 1444 | neocryptotanshinone （tanshinone V） | Diterpenoids | P06213 | 0.103151027 | 4 |
| 1445 | neocryptotanshinone （tanshinone V） | Diterpenoids | P06239 | 0.102843593 | 5 |
| 1446 | neocryptotanshinone （tanshinone V） | Diterpenoids | P07333 | 0.104063456 | 9 |
| 1447 | neocryptotanshinone （tanshinone V） | Diterpenoids | P07947 | 0.101613855 | 3 |
| 1448 | neocryptotanshinone （tanshinone V） | Diterpenoids | P07949 | 0.103471701 | 8 |
| 1449 | neocryptotanshinone （tanshinone V） | Diterpenoids | P08069 | 0.112934324 | 3 |
| 1450 | neocryptotanshinone （tanshinone V） | Diterpenoids | P08183 | 0.104694286 | 5 |
| 1451 | neocryptotanshinone （tanshinone V） | Diterpenoids | P0DMS8 | 0.10464569 | 23 |
| 1452 | neocryptotanshinone （tanshinone V） | Diterpenoids | P10721 | 0.104909858 | 3 |
| 1453 | neocryptotanshinone （tanshinone V） | Diterpenoids | P12931 | 0.104821195 | 5 |
| 1454 | neocryptotanshinone （tanshinone V） | Diterpenoids | P14555 | 0.102138215 | 3 |
| 1455 | neocryptotanshinone （tanshinone V） | Diterpenoids | P15121 | 0.267155221 | 39 |
| 1456 | neocryptotanshinone （tanshinone V） | Diterpenoids | P16234 | 0.100367415 | 3 |
| 1457 | neocryptotanshinone （tanshinone V） | Diterpenoids | P17252 | 0.102754312 | 7 |
| 1458 | neocryptotanshinone （tanshinone V） | Diterpenoids | P17948 | 0.105554897 | 13 |
| 1459 | neocryptotanshinone （tanshinone V） | Diterpenoids | P24385 P11802 | 0.105464394 | 4 |
| 1460 | neocryptotanshinone （tanshinone V） | Diterpenoids | P24723 | 0.11185669 | 11 |
| 1461 | neocryptotanshinone （tanshinone V） | Diterpenoids | P24941 | 0.106080884 | 8 |
| 1462 | neocryptotanshinone （tanshinone V） | Diterpenoids | P28472 P18507 P31644 | 0.103791839 | 19 |
| 1463 | neocryptotanshinone （tanshinone V） | Diterpenoids | P29274 | 0.103202685 | 27 |
| 1464 | neocryptotanshinone （tanshinone V） | Diterpenoids | P30542 | 0.101318267 | 24 |
| 1465 | neocryptotanshinone （tanshinone V） | Diterpenoids | P31639 | 0.109951316 | 5 |
| 1466 | neocryptotanshinone （tanshinone V） | Diterpenoids | P31751 | 0.110657683 | 3 |
| 1467 | neocryptotanshinone （tanshinone V） | Diterpenoids | P34972 | 0.103169308 | 13 |
| 1468 | neocryptotanshinone （tanshinone V） | Diterpenoids | P35916 | 0.105748254 | 5 |
| 1469 | neocryptotanshinone （tanshinone V） | Diterpenoids | P35968 | 0.105052994 | 26 |
| 1470 | neocryptotanshinone （tanshinone V） | Diterpenoids | P36888 | 0.102892289 | 8 |
| 1471 | neocryptotanshinone （tanshinone V） | Diterpenoids | P43405 | 0.106351077 | 5 |
| 1472 | neocryptotanshinone （tanshinone V） | Diterpenoids | P45983 | 0.105088123 | 17 |
| 1473 | neocryptotanshinone （tanshinone V） | Diterpenoids | P49137 | 0.107101597 | 6 |
| 1474 | neocryptotanshinone （tanshinone V） | Diterpenoids | P49840 | 0.108060801 | 5 |
| 1475 | neocryptotanshinone （tanshinone V） | Diterpenoids | P49841 | 0.105823985 | 11 |
| 1476 | neocryptotanshinone （tanshinone V） | Diterpenoids | P51812 | 0.135853345 | 4 |
| 1477 | neocryptotanshinone （tanshinone V） | Diterpenoids | P52333 | 0.104243408 | 12 |
| 1478 | neocryptotanshinone （tanshinone V） | Diterpenoids | P53779 | 0.103177433 | 9 |
| 1479 | neocryptotanshinone （tanshinone V） | Diterpenoids | Q00987 | 0.107095673 | 11 |
| 1480 | neocryptotanshinone （tanshinone V） | Diterpenoids | Q05397 | 0.104909858 | 3 |
| 1481 | neocryptotanshinone （tanshinone V） | Diterpenoids | Q05513 | 0.104564874 | 3 |
| 1482 | neocryptotanshinone （tanshinone V） | Diterpenoids | Q05655 | 0.102598878 | 5 |
| 1483 | neocryptotanshinone （tanshinone V） | Diterpenoids | Q13627 | 0.104383674 | 7 |
| 1484 | neocryptotanshinone （tanshinone V） | Diterpenoids | Q15746 | 0.102744609 | 5 |
| 1485 | neocryptotanshinone （tanshinone V） | Diterpenoids | Q16539 | 0.105438848 | 20 |
| 1486 | neocryptotanshinone （tanshinone V） | Diterpenoids | Q16584 | 0.104189154 | 3 |
| 1487 | neocryptotanshinone （tanshinone V） | Diterpenoids | Q5S007 | 0.102368749 | 9 |
| 1488 | neocryptotanshinone （tanshinone V） | Diterpenoids | Q96GD4 | 0.103151027 | 4 |
| 1489 | neocryptotanshinone II | Diterpenoids | O00408 | 0.103882897 | 9 |
| 1490 | neocryptotanshinone II | Diterpenoids | O60427 | 0.10804089 | 3 |
| 1491 | neocryptotanshinone II | Diterpenoids | O75460 | 0.105470076 | 5 |
| 1492 | neocryptotanshinone II | Diterpenoids | P04278 | 0.16719946 | 19 |
| 1493 | neocryptotanshinone II | Diterpenoids | P05186 | 0.104336352 | 9 |
| 1494 | neocryptotanshinone II | Diterpenoids | P07333 | 0.104063456 | 9 |
| 1495 | neocryptotanshinone II | Diterpenoids | P08581 | 0.109668623 | 6 |
| 1496 | neocryptotanshinone II | Diterpenoids | P14416 | 0.108887011 | 4 |
| 1497 | neocryptotanshinone II | Diterpenoids | P14867 | 0.10300422 | 7 |
| 1498 | neocryptotanshinone II | Diterpenoids | P14902 | 0.110454469 | 24 |
| 1499 | neocryptotanshinone II | Diterpenoids | P15056 | 0.101798823 | 4 |
| 1500 | neocryptotanshinone II | Diterpenoids | P15121 | 0.267155221 | 39 |
| 1501 | neocryptotanshinone II | Diterpenoids | P16050 | 0.105228934 | 6 |
| 1502 | neocryptotanshinone II | Diterpenoids | P16083 | 0.112425971 | 14 |
| 1503 | neocryptotanshinone II | Diterpenoids | P18054 | 0.103847438 | 5 |
| 1504 | neocryptotanshinone II | Diterpenoids | P21397 | 0.102816353 | 15 |
| 1505 | neocryptotanshinone II | Diterpenoids | P21728 | 0.105764248 | 3 |
| 1506 | neocryptotanshinone II | Diterpenoids | P27338 | 0.105021471 | 16 |
| 1507 | neocryptotanshinone II | Diterpenoids | P28223 | 0.103919859 | 7 |
| 1508 | neocryptotanshinone II | Diterpenoids | P28472 P18507 P14867 | 0.103855577 | 16 |
| 1509 | neocryptotanshinone II | Diterpenoids | P28472 P18507 P31644 | 0.103791839 | 19 |
| 1510 | neocryptotanshinone II | Diterpenoids | P28472 P34903 P18507 | 0.103855577 | 16 |
| 1511 | neocryptotanshinone II | Diterpenoids | P29274 | 0.103202685 | 27 |
| 1512 | neocryptotanshinone II | Diterpenoids | P30305 | 0.185734372 | 40 |
| 1513 | neocryptotanshinone II | Diterpenoids | P30536 | 0.102095712 | 5 |
| 1514 | neocryptotanshinone II | Diterpenoids | P35228 | 0.112440313 | 15 |
| 1515 | neocryptotanshinone II | Diterpenoids | P35354 | 0.113487837 | 23 |
| 1516 | neocryptotanshinone II | Diterpenoids | P35462 | 0.105764248 | 3 |
| 1517 | neocryptotanshinone II | Diterpenoids | P37059 | 0.106459925 | 6 |
| 1518 | neocryptotanshinone II | Diterpenoids | P41595 | 0.102913571 | 7 |
| 1519 | neocryptotanshinone II | Diterpenoids | P43166 | 0.165991461 | 6 |
| 1520 | neocryptotanshinone II | Diterpenoids | P46098 | 0.103859168 | 6 |
| 1521 | neocryptotanshinone II | Diterpenoids | P47869 P28472 P18507 | 0.104254314 | 15 |
| 1522 | neocryptotanshinone II | Diterpenoids | P48039 | 0.101354935 | 19 |
| 1523 | neocryptotanshinone II | Diterpenoids | P49286 | 0.101354935 | 19 |
| 1524 | neocryptotanshinone II | Diterpenoids | P49354 P49356 | 0.109904857 | 24 |
| 1525 | neocryptotanshinone II | Diterpenoids | P49810 Q9NZ42 Q92542 Q96BI3 P49768 Q8WW43 | 0.101543467 | 9 |
| 1526 | neocryptotanshinone II | Diterpenoids | P52333 | 0.104243408 | 12 |
| 1527 | neocryptotanshinone II | Diterpenoids | P52732 | 0.104747724 | 9 |
| 1528 | neocryptotanshinone II | Diterpenoids | Q02127 | 0.106699814 | 5 |
| 1529 | neocryptotanshinone II | Diterpenoids | Q05513 | 0.104564874 | 3 |
| 1530 | neocryptotanshinone II | Diterpenoids | Q12791 | 0.103498446 | 3 |
| 1531 | neocryptotanshinone II | Diterpenoids | Q15746 | 0.102744609 | 5 |
| 1532 | neocryptotanshinone II | Diterpenoids | Q5S007 | 0.102368749 | 9 |
| 1533 | neocryptotanshinone II | Diterpenoids | Q9Y233 | 0.108085897 | 14 |
| 1534 | neotanshinlactone | Diterpenoids | O00748 | 0.259386615 | 47 |
| 1535 | neotanshinlactone | Diterpenoids | O14684 | 0.116389783 | 19 |
| 1536 | neotanshinlactone | Diterpenoids | O75530 Q15022 Q15910 | 0.341964133 | 19 |
| 1537 | neotanshinlactone | Diterpenoids | O76074 | 0.103099774 | 12 |
| 1538 | neotanshinlactone | Diterpenoids | P16083 | 0.112425971 | 14 |
| 1539 | neotanshinlactone | Diterpenoids | P21397 | 0.102816353 | 15 |
| 1540 | neotanshinlactone | Diterpenoids | P25025 | 0.100320282 | 13 |
| 1541 | neotanshinlactone | Diterpenoids | P28335 | 0.104521595 | 9 |
| 1542 | neotanshinlactone | Diterpenoids | P29274 | 0.103202685 | 27 |
| 1543 | neotanshinlactone | Diterpenoids | P29350 | 0.204438393 | 44 |
| 1544 | neotanshinlactone | Diterpenoids | P30305 | 0.185734372 | 40 |
| 1545 | neotanshinlactone | Diterpenoids | P30556 | 0.113117188 | 7 |
| 1546 | neotanshinlactone | Diterpenoids | P33261 | 0.124903734 | 16 |
| 1547 | neotanshinlactone | Diterpenoids | P48039 | 0.101354935 | 19 |
| 1548 | neotanshinlactone | Diterpenoids | P49286 | 0.101354935 | 19 |
| 1549 | neotanshinlactone | Diterpenoids | P52732 | 0.104747724 | 9 |
| 1550 | neotanshinlactone | Diterpenoids | Q06124 | 0.211032909 | 40 |
| 1551 | neotanshinlactone | Diterpenoids | Q16539 | 0.105438848 | 20 |
| 1552 | neotanshinlactone | Diterpenoids | Q86V86 | 0.100967138 | 7 |
| 1553 | neotanshinlactone | Diterpenoids | Q99572 | 0.10437669 | 25 |
| 1554 | neotanshinlactone | Diterpenoids | Q9P1W9 | 0.103344117 | 12 |
| 1555 | nortanshinone | Diterpenoids | O00748 | 0.259386615 | 47 |
| 1556 | nortanshinone | Diterpenoids | O14746 | 0.14728518 | 27 |
| 1557 | nortanshinone | Diterpenoids | O43614 | 0.100916735 | 11 |
| 1558 | nortanshinone | Diterpenoids | O75530 Q15022 Q15910 | 0.341964133 | 19 |
| 1559 | nortanshinone | Diterpenoids | P03372 | 0.132037828 | 16 |
| 1560 | nortanshinone | Diterpenoids | P08253 | 0.106581489 | 9 |
| 1561 | nortanshinone | Diterpenoids | P08575 | 0.109148612 | 27 |
| 1562 | nortanshinone | Diterpenoids | P0DMS8 | 0.10464569 | 23 |
| 1563 | nortanshinone | Diterpenoids | P10275 | 0.132131799 | 34 |
| 1564 | nortanshinone | Diterpenoids | P11511 | 0.139056613 | 29 |
| 1565 | nortanshinone | Diterpenoids | P11940 | 0.1046882 | 6 |
| 1566 | nortanshinone | Diterpenoids | P12268 | 0.104524224 | 13 |
| 1567 | nortanshinone | Diterpenoids | P15121 | 0.267155221 | 39 |
| 1568 | nortanshinone | Diterpenoids | P15538 | 0.10615894 | 5 |
| 1569 | nortanshinone | Diterpenoids | P16083 | 0.112425971 | 14 |
| 1570 | nortanshinone | Diterpenoids | P19099 | 0.10615894 | 5 |
| 1571 | nortanshinone | Diterpenoids | P22736 | 0.100039999 | 6 |
| 1572 | nortanshinone | Diterpenoids | P23141 | 0.275271523 | 31 |
| 1573 | nortanshinone | Diterpenoids | P29274 | 0.103202685 | 27 |
| 1574 | nortanshinone | Diterpenoids | P29275 | 0.103333221 | 16 |
| 1575 | nortanshinone | Diterpenoids | P29350 | 0.204438393 | 44 |
| 1576 | nortanshinone | Diterpenoids | P30304 | 0.109145376 | 25 |
| 1577 | nortanshinone | Diterpenoids | P30305 | 0.185734372 | 40 |
| 1578 | nortanshinone | Diterpenoids | P30307 | 0.105546712 | 12 |
| 1579 | nortanshinone | Diterpenoids | P30542 | 0.101318267 | 24 |
| 1580 | nortanshinone | Diterpenoids | P34949 | 0.105190036 | 9 |
| 1581 | nortanshinone | Diterpenoids | P45983 | 0.105088123 | 17 |
| 1582 | nortanshinone | Diterpenoids | P48039 | 0.101354935 | 19 |
| 1583 | nortanshinone | Diterpenoids | P48147 | 0.107233003 | 22 |
| 1584 | nortanshinone | Diterpenoids | P49286 | 0.101354935 | 19 |
| 1585 | nortanshinone | Diterpenoids | P49810 Q9NZ42 Q92542 Q96BI3 P49768 Q8WW43 | 0.101543467 | 9 |
| 1586 | nortanshinone | Diterpenoids | P53779 | 0.103177433 | 9 |
| 1587 | nortanshinone | Diterpenoids | Q06124 | 0.211032909 | 40 |
| 1588 | nortanshinone | Diterpenoids | Q07820 | 0.101122731 | 4 |
| 1589 | nortanshinone | Diterpenoids | Q09028 Q16576 O75530 Q15022 Q15910 | 0.142197972 | 14 |
| 1590 | nortanshinone | Diterpenoids | Q15078 Q00535 | 0.102910354 | 14 |
| 1591 | nortanshinone | Diterpenoids | Q8WWL7 P06493 P14635 O95067 | 0.10231122 | 6 |
| 1592 | nortanshinone | Diterpenoids | Q92731 | 0.117586956 | 23 |
| 1593 | nortanshinone | Diterpenoids | Q99572 | 0.10437669 | 25 |
| 1594 | nortanshinone | Diterpenoids | Q9H2K2 | 0.104609573 | 11 |
| 1595 | nortanshinone | Diterpenoids | Q9Y5N1 | 0.101047212 | 6 |
| 1596 | przewaquinone A | Diterpenoids | O00748 | 0.259386615 | 47 |
| 1597 | przewaquinone A | Diterpenoids | O14746 | 0.14728518 | 27 |
| 1598 | przewaquinone A | Diterpenoids | O14757 | 0.106739614 | 8 |
| 1599 | przewaquinone A | Diterpenoids | O75530 Q15022 Q15910 | 0.341964133 | 19 |
| 1600 | przewaquinone A | Diterpenoids | O76074 | 0.103099774 | 12 |
| 1601 | przewaquinone A | Diterpenoids | P04035 | 0.166864971 | 20 |
| 1602 | przewaquinone A | Diterpenoids | P04150 | 0.109500234 | 17 |
| 1603 | przewaquinone A | Diterpenoids | P06737 | 0.103331553 | 10 |
| 1604 | przewaquinone A | Diterpenoids | P08185 | 0.113039089 | 17 |
| 1605 | przewaquinone A | Diterpenoids | P08473 | 0.108391881 | 3 |
| 1606 | przewaquinone A | Diterpenoids | P08575 | 0.109148612 | 27 |
| 1607 | przewaquinone A | Diterpenoids | P08842 | 0.103451903 | 3 |
| 1608 | przewaquinone A | Diterpenoids | P11229 | 0.103603883 | 11 |
| 1609 | przewaquinone A | Diterpenoids | P14555 | 0.102138215 | 3 |
| 1610 | przewaquinone A | Diterpenoids | P15121 | 0.267155221 | 39 |
| 1611 | przewaquinone A | Diterpenoids | P21397 | 0.102816353 | 15 |
| 1612 | przewaquinone A | Diterpenoids | P22303 | 0.287353637 | 26 |
| 1613 | przewaquinone A | Diterpenoids | P23141 | 0.275271523 | 31 |
| 1614 | przewaquinone A | Diterpenoids | P28845 | 0.253303956 | 34 |
| 1615 | przewaquinone A | Diterpenoids | P29350 | 0.204438393 | 44 |
| 1616 | przewaquinone A | Diterpenoids | P30304 | 0.109145376 | 25 |
| 1617 | przewaquinone A | Diterpenoids | P30542 | 0.101318267 | 24 |
| 1618 | przewaquinone A | Diterpenoids | P34949 | 0.105190036 | 9 |
| 1619 | przewaquinone A | Diterpenoids | P41594 | 0.103693674 | 22 |
| 1620 | przewaquinone A | Diterpenoids | P42336 | 0.108042586 | 13 |
| 1621 | przewaquinone A | Diterpenoids | P42574 | 0.10400953 | 13 |
| 1622 | przewaquinone A | Diterpenoids | P55210 | 0.10400953 | 13 |
| 1623 | przewaquinone A | Diterpenoids | P55263 | 0.107485158 | 3 |
| 1624 | przewaquinone A | Diterpenoids | Q00796 | 0.107710558 | 5 |
| 1625 | przewaquinone A | Diterpenoids | Q02750 | 0.106463868 | 11 |
| 1626 | przewaquinone A | Diterpenoids | Q06124 | 0.211032909 | 40 |
| 1627 | przewaquinone A | Diterpenoids | Q09028 Q16576 O75530 Q15022 Q15910 | 0.142197972 | 14 |
| 1628 | przewaquinone A | Diterpenoids | Q13255 | 0.103848909 | 9 |
| 1629 | przewaquinone A | Diterpenoids | Q14790 | 0.104694286 | 5 |
| 1630 | przewaquinone A | Diterpenoids | Q15858 | 0.101761829 | 5 |
| 1631 | przewaquinone A | Diterpenoids | Q16539 | 0.105438848 | 20 |
| 1632 | przewaquinone A | Diterpenoids | Q99572 | 0.10437669 | 25 |
| 1633 | salviamone | Diterpenoids | O00519 | 0.110352709 | 16 |
| 1634 | salviamone | Diterpenoids | O00748 | 0.259386615 | 47 |
| 1635 | salviamone | Diterpenoids | O43194 | 0.10421989 | 3 |
| 1636 | salviamone | Diterpenoids | O75874 | 0.1046882 | 6 |
| 1637 | salviamone | Diterpenoids | O96020 P24941 P24864 | 0.104035866 | 9 |
| 1638 | salviamone | Diterpenoids | P00519 | 0.104821195 | 5 |
| 1639 | salviamone | Diterpenoids | P04626 | 0.104502793 | 11 |
| 1640 | salviamone | Diterpenoids | P04629 | 0.107046621 | 8 |
| 1641 | salviamone | Diterpenoids | P05362 | 0.105155078 | 5 |
| 1642 | salviamone | Diterpenoids | P06213 | 0.103151027 | 4 |
| 1643 | salviamone | Diterpenoids | P08172 | 0.117318329 | 18 |
| 1644 | salviamone | Diterpenoids | P08575 | 0.109148612 | 27 |
| 1645 | salviamone | Diterpenoids | P08581 | 0.109668623 | 6 |
| 1646 | salviamone | Diterpenoids | P08908 | 0.10562303 | 4 |
| 1647 | salviamone | Diterpenoids | P0DMS8 | 0.10464569 | 23 |
| 1648 | salviamone | Diterpenoids | P11229 | 0.103603883 | 11 |
| 1649 | salviamone | Diterpenoids | P11309 | 0.104805981 | 13 |
| 1650 | salviamone | Diterpenoids | P12931 | 0.104821195 | 5 |
| 1651 | salviamone | Diterpenoids | P15121 | 0.267155221 | 39 |
| 1652 | salviamone | Diterpenoids | P16581 | 0.105155078 | 5 |
| 1653 | salviamone | Diterpenoids | P17948 | 0.105554897 | 13 |
| 1654 | salviamone | Diterpenoids | P19320 | 0.105155078 | 5 |
| 1655 | salviamone | Diterpenoids | P20248 P24941 | 0.112800203 | 4 |
| 1656 | salviamone | Diterpenoids | P20309 | 0.105165619 | 10 |
| 1657 | salviamone | Diterpenoids | P21397 | 0.102816353 | 15 |
| 1658 | salviamone | Diterpenoids | P21554 | 0.104626059 | 10 |
| 1659 | salviamone | Diterpenoids | P22303 | 0.287353637 | 26 |
| 1660 | salviamone | Diterpenoids | P23141 | 0.275271523 | 31 |
| 1661 | salviamone | Diterpenoids | P24385 P11802 | 0.105464394 | 4 |
| 1662 | salviamone | Diterpenoids | P24863 P49336 | 0.102633551 | 4 |
| 1663 | salviamone | Diterpenoids | P24864 P24941 | 0.102540734 | 7 |
| 1664 | salviamone | Diterpenoids | P24941 | 0.106080884 | 8 |
| 1665 | salviamone | Diterpenoids | P25774 | 0.106840916 | 6 |
| 1666 | salviamone | Diterpenoids | P27815 | 0.106747907 | 3 |
| 1667 | salviamone | Diterpenoids | P28223 | 0.103919859 | 7 |
| 1668 | salviamone | Diterpenoids | P28335 | 0.104521595 | 9 |
| 1669 | salviamone | Diterpenoids | P28472 P18507 P31644 | 0.103791839 | 19 |
| 1670 | salviamone | Diterpenoids | P29274 | 0.103202685 | 27 |
| 1671 | salviamone | Diterpenoids | P29275 | 0.103333221 | 16 |
| 1672 | salviamone | Diterpenoids | P29350 | 0.204438393 | 44 |
| 1673 | salviamone | Diterpenoids | P30542 | 0.101318267 | 24 |
| 1674 | salviamone | Diterpenoids | P34969 | 0.102205463 | 3 |
| 1675 | salviamone | Diterpenoids | P34972 | 0.103169308 | 13 |
| 1676 | salviamone | Diterpenoids | P35557 | 0.102633551 | 4 |
| 1677 | salviamone | Diterpenoids | P35916 | 0.105748254 | 5 |
| 1678 | salviamone | Diterpenoids | P35968 | 0.105052994 | 26 |
| 1679 | salviamone | Diterpenoids | P36888 | 0.102892289 | 8 |
| 1680 | salviamone | Diterpenoids | P37023 | 0.111434828 | 4 |
| 1681 | salviamone | Diterpenoids | P41594 | 0.103693674 | 22 |
| 1682 | salviamone | Diterpenoids | P42336 | 0.108042586 | 13 |
| 1683 | salviamone | Diterpenoids | P42338 | 0.107541648 | 9 |
| 1684 | salviamone | Diterpenoids | P42574 | 0.10400953 | 13 |
| 1685 | salviamone | Diterpenoids | P43235 | 0.104158358 | 7 |
| 1686 | salviamone | Diterpenoids | P45983 | 0.105088123 | 17 |
| 1687 | salviamone | Diterpenoids | P46098 | 0.103859168 | 6 |
| 1688 | salviamone | Diterpenoids | P48039 | 0.101354935 | 19 |
| 1689 | salviamone | Diterpenoids | P48147 | 0.107233003 | 22 |
| 1690 | salviamone | Diterpenoids | P49286 | 0.101354935 | 19 |
| 1691 | salviamone | Diterpenoids | P49841 | 0.105823985 | 11 |
| 1692 | salviamone | Diterpenoids | P50406 | 0.106847218 | 8 |
| 1693 | salviamone | Diterpenoids | P53779 | 0.103177433 | 9 |
| 1694 | salviamone | Diterpenoids | P55210 | 0.10400953 | 13 |
| 1695 | salviamone | Diterpenoids | P78536 | 0.104291862 | 9 |
| 1696 | salviamone | Diterpenoids | Q02083 | 0.102416978 | 6 |
| 1697 | salviamone | Diterpenoids | Q06124 | 0.211032909 | 40 |
| 1698 | salviamone | Diterpenoids | Q13255 | 0.103848909 | 9 |
| 1699 | salviamone | Diterpenoids | Q14833 | 0.102633551 | 4 |
| 1700 | salviamone | Diterpenoids | Q15078 Q00535 | 0.102910354 | 14 |
| 1701 | salviamone | Diterpenoids | Q16539 | 0.105438848 | 20 |
| 1702 | salviamone | Diterpenoids | Q8IXJ6 | 0.108888011 | 5 |
| 1703 | salviamone | Diterpenoids | Q8WWL7 P06493 P14635 O95067 | 0.10231122 | 6 |
| 1704 | salviamone | Diterpenoids | Q99572 | 0.10437669 | 25 |
| 1705 | salviamone | Diterpenoids | Q9H2K2 | 0.104609573 | 11 |
| 1706 | salviamone | Diterpenoids | Q9P1W9 | 0.103344117 | 12 |
| 1707 | salviamone | Diterpenoids | Q9Y233 | 0.108085897 | 14 |
| 1708 | tanshinaldehyde I （formyltanshinone） | Diterpenoids | O00748 | 0.259386615 | 47 |
| 1709 | tanshinaldehyde I （formyltanshinone） | Diterpenoids | O14746 | 0.14728518 | 27 |
| 1710 | tanshinaldehyde I （formyltanshinone） | Diterpenoids | O75530 Q15022 Q15910 | 0.341964133 | 19 |
| 1711 | tanshinaldehyde I （formyltanshinone） | Diterpenoids | P00533 | 0.104151342 | 24 |
| 1712 | tanshinaldehyde I （formyltanshinone） | Diterpenoids | P04150 | 0.109500234 | 17 |
| 1713 | tanshinaldehyde I （formyltanshinone） | Diterpenoids | P04626 | 0.104502793 | 11 |
| 1714 | tanshinaldehyde I （formyltanshinone） | Diterpenoids | P06401 | 0.108868706 | 15 |
| 1715 | tanshinaldehyde I （formyltanshinone） | Diterpenoids | P08235 | 0.107998327 | 12 |
| 1716 | tanshinaldehyde I （formyltanshinone） | Diterpenoids | P08246 | 0.10362417 | 7 |
| 1717 | tanshinaldehyde I （formyltanshinone） | Diterpenoids | P08575 | 0.109148612 | 27 |
| 1718 | tanshinaldehyde I （formyltanshinone） | Diterpenoids | P09874 | 0.106818993 | 18 |
| 1719 | tanshinaldehyde I （formyltanshinone） | Diterpenoids | P10275 | 0.132131799 | 34 |
| 1720 | tanshinaldehyde I （formyltanshinone） | Diterpenoids | P15121 | 0.267155221 | 39 |
| 1721 | tanshinaldehyde I （formyltanshinone） | Diterpenoids | P15538 | 0.10615894 | 5 |
| 1722 | tanshinaldehyde I （formyltanshinone） | Diterpenoids | P19099 | 0.10615894 | 5 |
| 1723 | tanshinaldehyde I （formyltanshinone） | Diterpenoids | P23141 | 0.275271523 | 31 |
| 1724 | tanshinaldehyde I （formyltanshinone） | Diterpenoids | P25101 | 0.104008389 | 6 |
| 1725 | tanshinaldehyde I （formyltanshinone） | Diterpenoids | P27338 | 0.105021471 | 16 |
| 1726 | tanshinaldehyde I （formyltanshinone） | Diterpenoids | P29274 | 0.103202685 | 27 |
| 1727 | tanshinaldehyde I （formyltanshinone） | Diterpenoids | P29275 | 0.103333221 | 16 |
| 1728 | tanshinaldehyde I （formyltanshinone） | Diterpenoids | P29350 | 0.204438393 | 44 |
| 1729 | tanshinaldehyde I （formyltanshinone） | Diterpenoids | P30304 | 0.109145376 | 25 |
| 1730 | tanshinaldehyde I （formyltanshinone） | Diterpenoids | P30305 | 0.185734372 | 40 |
| 1731 | tanshinaldehyde I （formyltanshinone） | Diterpenoids | P30307 | 0.105546712 | 12 |
| 1732 | tanshinaldehyde I （formyltanshinone） | Diterpenoids | P30542 | 0.101318267 | 24 |
| 1733 | tanshinaldehyde I （formyltanshinone） | Diterpenoids | P34949 | 0.105190036 | 9 |
| 1734 | tanshinaldehyde I （formyltanshinone） | Diterpenoids | P41594 | 0.103693674 | 22 |
| 1735 | tanshinaldehyde I （formyltanshinone） | Diterpenoids | P45983 | 0.105088123 | 17 |
| 1736 | tanshinaldehyde I （formyltanshinone） | Diterpenoids | P53779 | 0.103177433 | 9 |
| 1737 | tanshinaldehyde I （formyltanshinone） | Diterpenoids | Q06124 | 0.211032909 | 40 |
| 1738 | tanshinaldehyde I （formyltanshinone） | Diterpenoids | Q09028 Q16576 O75530 Q15022 Q15910 | 0.142197972 | 14 |
| 1739 | tanshinaldehyde I （formyltanshinone） | Diterpenoids | Q99572 | 0.10437669 | 25 |
| 1740 | tanshinlactone | Diterpenoids | P00338 | 0.106699814 | 5 |
| 1741 | tanshinlactone | Diterpenoids | P06493 | 0.102732601 | 7 |
| 1742 | tanshinlactone | Diterpenoids | P10275 | 0.132131799 | 34 |
| 1743 | tanshinlactone | Diterpenoids | P21397 | 0.102816353 | 15 |
| 1744 | tanshinlactone | Diterpenoids | P25101 | 0.104008389 | 6 |
| 1745 | tanshinlactone | Diterpenoids | P28335 | 0.104521595 | 9 |
| 1746 | tanshinlactone | Diterpenoids | P28472 P18507 P14867 | 0.103855577 | 16 |
| 1747 | tanshinlactone | Diterpenoids | P28472 P18507 P31644 | 0.103791839 | 19 |
| 1748 | tanshinlactone | Diterpenoids | P28472 P34903 P18507 | 0.103855577 | 16 |
| 1749 | tanshinlactone | Diterpenoids | P29274 | 0.103202685 | 27 |
| 1750 | tanshinlactone | Diterpenoids | P31645 | 0.1201852 | 17 |
| 1751 | tanshinlactone | Diterpenoids | P47869 P28472 P18507 | 0.104254314 | 15 |
| 1752 | tanshinlactone | Diterpenoids | P49137 | 0.107101597 | 6 |
| 1753 | tanshinlactone | Diterpenoids | Q08499 | 0.31881944 | 14 |
| 1754 | tanshinlactone | Diterpenoids | Q12809 | 0.105584236 | 3 |
| 1755 | tanshinlactone | Diterpenoids | Q15858 | 0.101761829 | 5 |
| 1756 | tanshinlactone | Diterpenoids | Q16539 | 0.105438848 | 20 |
| 1757 | tanshinlactone | Diterpenoids | Q99720 | 0.110060881 | 8 |
| 1758 | tanshinlactone | Diterpenoids | Q9NZJ5 | 0.102919059 | 6 |
| 1759 | tanshinol B (Przewaquinone C) | Diterpenoids | O00311 | 0.105117399 | 6 |
| 1760 | tanshinol B (Przewaquinone C) | Diterpenoids | O00329 | 0.105574288 | 6 |
| 1761 | tanshinol B (Przewaquinone C) | Diterpenoids | O00408 | 0.103882897 | 9 |
| 1762 | tanshinol B (Przewaquinone C) | Diterpenoids | O00519 | 0.110352709 | 16 |
| 1763 | tanshinol B (Przewaquinone C) | Diterpenoids | O00748 | 0.259386615 | 47 |
| 1764 | tanshinol B (Przewaquinone C) | Diterpenoids | O14746 | 0.14728518 | 27 |
| 1765 | tanshinol B (Przewaquinone C) | Diterpenoids | O14965 | 0.107316885 | 8 |
| 1766 | tanshinol B (Przewaquinone C) | Diterpenoids | O60674 | 0.103305945 | 11 |
| 1767 | tanshinol B (Przewaquinone C) | Diterpenoids | O60706 | 0.103819513 | 5 |
| 1768 | tanshinol B (Przewaquinone C) | Diterpenoids | O60725 | 0.108205862 | 3 |
| 1769 | tanshinol B (Przewaquinone C) | Diterpenoids | O75530 Q15022 Q15910 | 0.341964133 | 19 |
| 1770 | tanshinol B (Przewaquinone C) | Diterpenoids | P00533 | 0.104151342 | 24 |
| 1771 | tanshinol B (Przewaquinone C) | Diterpenoids | P00742 | 0.111476369 | 3 |
| 1772 | tanshinol B (Przewaquinone C) | Diterpenoids | P00915 | 0.106312298 | 8 |
| 1773 | tanshinol B (Przewaquinone C) | Diterpenoids | P00918 | 0.141292356 | 8 |
| 1774 | tanshinol B (Przewaquinone C) | Diterpenoids | P03951 | 0.106451353 | 3 |
| 1775 | tanshinol B (Przewaquinone C) | Diterpenoids | P04035 | 0.166864971 | 20 |
| 1776 | tanshinol B (Przewaquinone C) | Diterpenoids | P04150 | 0.109500234 | 17 |
| 1777 | tanshinol B (Przewaquinone C) | Diterpenoids | P04629 | 0.107046621 | 8 |
| 1778 | tanshinol B (Przewaquinone C) | Diterpenoids | P05093 | 0.148009819 | 20 |
| 1779 | tanshinol B (Przewaquinone C) | Diterpenoids | P05186 | 0.104336352 | 9 |
| 1780 | tanshinol B (Przewaquinone C) | Diterpenoids | P06493 | 0.102732601 | 7 |
| 1781 | tanshinol B (Przewaquinone C) | Diterpenoids | P06737 | 0.103331553 | 10 |
| 1782 | tanshinol B (Przewaquinone C) | Diterpenoids | P07333 | 0.104063456 | 9 |
| 1783 | tanshinol B (Przewaquinone C) | Diterpenoids | P07384 | 0.10807591 | 4 |
| 1784 | tanshinol B (Przewaquinone C) | Diterpenoids | P07477 | 0.103976264 | 3 |
| 1785 | tanshinol B (Przewaquinone C) | Diterpenoids | P08183 | 0.104694286 | 5 |
| 1786 | tanshinol B (Przewaquinone C) | Diterpenoids | P08575 | 0.109148612 | 27 |
| 1787 | tanshinol B (Przewaquinone C) | Diterpenoids | P08684 | 0.104909858 | 3 |
| 1788 | tanshinol B (Przewaquinone C) | Diterpenoids | P09601 | 0.103663418 | 3 |
| 1789 | tanshinol B (Przewaquinone C) | Diterpenoids | P09874 | 0.106818993 | 18 |
| 1790 | tanshinol B (Przewaquinone C) | Diterpenoids | P09917 | 0.106782208 | 14 |
| 1791 | tanshinol B (Przewaquinone C) | Diterpenoids | P10275 | 0.132131799 | 34 |
| 1792 | tanshinol B (Przewaquinone C) | Diterpenoids | P11309 | 0.104805981 | 13 |
| 1793 | tanshinol B (Przewaquinone C) | Diterpenoids | P11511 | 0.139056613 | 29 |
| 1794 | tanshinol B (Przewaquinone C) | Diterpenoids | P15121 | 0.267155221 | 39 |
| 1795 | tanshinol B (Przewaquinone C) | Diterpenoids | P15538 | 0.10615894 | 5 |
| 1796 | tanshinol B (Przewaquinone C) | Diterpenoids | P19099 | 0.10615894 | 5 |
| 1797 | tanshinol B (Przewaquinone C) | Diterpenoids | P20309 | 0.105165619 | 10 |
| 1798 | tanshinol B (Przewaquinone C) | Diterpenoids | P22748 | 0.219188074 | 3 |
| 1799 | tanshinol B (Przewaquinone C) | Diterpenoids | P23141 | 0.275271523 | 31 |
| 1800 | tanshinol B (Przewaquinone C) | Diterpenoids | P23219 | 0.109524644 | 16 |
| 1801 | tanshinol B (Przewaquinone C) | Diterpenoids | P23458 | 0.102916912 | 11 |
| 1802 | tanshinol B (Przewaquinone C) | Diterpenoids | P28221 | 0.104909858 | 3 |
| 1803 | tanshinol B (Przewaquinone C) | Diterpenoids | P28472 P18507 P14867 | 0.103855577 | 16 |
| 1804 | tanshinol B (Przewaquinone C) | Diterpenoids | P28472 P18507 P31644 | 0.103791839 | 19 |
| 1805 | tanshinol B (Przewaquinone C) | Diterpenoids | P28472 P34903 P18507 | 0.103855577 | 16 |
| 1806 | tanshinol B (Przewaquinone C) | Diterpenoids | P28845 | 0.253303956 | 34 |
| 1807 | tanshinol B (Przewaquinone C) | Diterpenoids | P29274 | 0.103202685 | 27 |
| 1808 | tanshinol B (Przewaquinone C) | Diterpenoids | P29275 | 0.103333221 | 16 |
| 1809 | tanshinol B (Przewaquinone C) | Diterpenoids | P29350 | 0.204438393 | 44 |
| 1810 | tanshinol B (Przewaquinone C) | Diterpenoids | P29466 | 0.105464394 | 4 |
| 1811 | tanshinol B (Przewaquinone C) | Diterpenoids | P30304 | 0.109145376 | 25 |
| 1812 | tanshinol B (Przewaquinone C) | Diterpenoids | P30305 | 0.185734372 | 40 |
| 1813 | tanshinol B (Przewaquinone C) | Diterpenoids | P30307 | 0.105546712 | 12 |
| 1814 | tanshinol B (Przewaquinone C) | Diterpenoids | P31644 | 0.101037512 | 7 |
| 1815 | tanshinol B (Przewaquinone C) | Diterpenoids | P33261 | 0.124903734 | 16 |
| 1816 | tanshinol B (Przewaquinone C) | Diterpenoids | P34949 | 0.105190036 | 9 |
| 1817 | tanshinol B (Przewaquinone C) | Diterpenoids | P35968 | 0.105052994 | 26 |
| 1818 | tanshinol B (Przewaquinone C) | Diterpenoids | P41145 | 0.107282863 | 6 |
| 1819 | tanshinol B (Przewaquinone C) | Diterpenoids | P41594 | 0.103693674 | 22 |
| 1820 | tanshinol B (Przewaquinone C) | Diterpenoids | P42336 | 0.108042586 | 13 |
| 1821 | tanshinol B (Przewaquinone C) | Diterpenoids | P42338 | 0.107541648 | 9 |
| 1822 | tanshinol B (Przewaquinone C) | Diterpenoids | P42574 | 0.10400953 | 13 |
| 1823 | tanshinol B (Przewaquinone C) | Diterpenoids | P43235 | 0.104158358 | 7 |
| 1824 | tanshinol B (Przewaquinone C) | Diterpenoids | P47869 P28472 P18507 | 0.104254314 | 15 |
| 1825 | tanshinol B (Przewaquinone C) | Diterpenoids | P48736 | 0.108205862 | 3 |
| 1826 | tanshinol B (Przewaquinone C) | Diterpenoids | P49810 Q9NZ42 Q92542 Q96BI3 P49768 Q8WW43 | 0.101543467 | 9 |
| 1827 | tanshinol B (Przewaquinone C) | Diterpenoids | P49841 | 0.105823985 | 11 |
| 1828 | tanshinol B (Przewaquinone C) | Diterpenoids | P50406 | 0.106847218 | 8 |
| 1829 | tanshinol B (Przewaquinone C) | Diterpenoids | P52333 | 0.104243408 | 12 |
| 1830 | tanshinol B (Przewaquinone C) | Diterpenoids | P52732 | 0.104747724 | 9 |
| 1831 | tanshinol B (Przewaquinone C) | Diterpenoids | P55210 | 0.10400953 | 13 |
| 1832 | tanshinol B (Przewaquinone C) | Diterpenoids | P78527 | 0.104180881 | 6 |
| 1833 | tanshinol B (Przewaquinone C) | Diterpenoids | Q00987 | 0.107095673 | 11 |
| 1834 | tanshinol B (Przewaquinone C) | Diterpenoids | Q02750 | 0.106463868 | 11 |
| 1835 | tanshinol B (Przewaquinone C) | Diterpenoids | Q06124 | 0.211032909 | 40 |
| 1836 | tanshinol B (Przewaquinone C) | Diterpenoids | Q09028 Q16576 O75530 Q15022 Q15910 | 0.142197972 | 14 |
| 1837 | tanshinol B (Przewaquinone C) | Diterpenoids | Q13946 | 0.104692547 | 7 |
| 1838 | tanshinol B (Przewaquinone C) | Diterpenoids | Q14790 | 0.104694286 | 5 |
| 1839 | tanshinol B (Przewaquinone C) | Diterpenoids | Q15078 Q00535 | 0.102910354 | 14 |
| 1840 | tanshinol B (Przewaquinone C) | Diterpenoids | Q16539 | 0.105438848 | 20 |
| 1841 | tanshinol B (Przewaquinone C) | Diterpenoids | Q7Z2W7 | 0.106776816 | 5 |
| 1842 | tanshinol B (Przewaquinone C) | Diterpenoids | Q99572 | 0.10437669 | 25 |
| 1843 | tanshinol B (Przewaquinone C) | Diterpenoids | Q9P1W9 | 0.103344117 | 12 |
| 1844 | tanshinol B (Przewaquinone C) | Diterpenoids | Q9Y233 | 0.108085897 | 14 |
| 1845 | tanshinone I | Diterpenoids | O00311 | 0.105117399 | 6 |
| 1846 | tanshinone I | Diterpenoids | O00748 | 0.259386615 | 47 |
| 1847 | tanshinone I | Diterpenoids | O14746 | 0.14728518 | 27 |
| 1848 | tanshinone I | Diterpenoids | O43614 | 0.100916735 | 11 |
| 1849 | tanshinone I | Diterpenoids | O75530 Q15022 Q15910 | 0.341964133 | 19 |
| 1850 | tanshinone I | Diterpenoids | P00533 | 0.104151342 | 24 |
| 1851 | tanshinone I | Diterpenoids | P08575 | 0.109148612 | 27 |
| 1852 | tanshinone I | Diterpenoids | P0DMS8 | 0.10464569 | 23 |
| 1853 | tanshinone I | Diterpenoids | P10275 | 0.132131799 | 34 |
| 1854 | tanshinone I | Diterpenoids | P11309 | 0.104805981 | 13 |
| 1855 | tanshinone I | Diterpenoids | P11511 | 0.139056613 | 29 |
| 1856 | tanshinone I | Diterpenoids | P12268 | 0.104524224 | 13 |
| 1857 | tanshinone I | Diterpenoids | P15121 | 0.267155221 | 39 |
| 1858 | tanshinone I | Diterpenoids | P16083 | 0.112425971 | 14 |
| 1859 | tanshinone I | Diterpenoids | P21397 | 0.102816353 | 15 |
| 1860 | tanshinone I | Diterpenoids | P22736 | 0.100039999 | 6 |
| 1861 | tanshinone I | Diterpenoids | P23141 | 0.275271523 | 31 |
| 1862 | tanshinone I | Diterpenoids | P25025 | 0.100320282 | 13 |
| 1863 | tanshinone I | Diterpenoids | P27338 | 0.105021471 | 16 |
| 1864 | tanshinone I | Diterpenoids | P29274 | 0.103202685 | 27 |
| 1865 | tanshinone I | Diterpenoids | P29275 | 0.103333221 | 16 |
| 1866 | tanshinone I | Diterpenoids | P29350 | 0.204438393 | 44 |
| 1867 | tanshinone I | Diterpenoids | P30304 | 0.109145376 | 25 |
| 1868 | tanshinone I | Diterpenoids | P30305 | 0.185734372 | 40 |
| 1869 | tanshinone I | Diterpenoids | P30307 | 0.105546712 | 12 |
| 1870 | tanshinone I | Diterpenoids | P30542 | 0.101318267 | 24 |
| 1871 | tanshinone I | Diterpenoids | P32247 | 0.103071649 | 5 |
| 1872 | tanshinone I | Diterpenoids | P34949 | 0.105190036 | 9 |
| 1873 | tanshinone I | Diterpenoids | P35348 | 0.101281367 | 4 |
| 1874 | tanshinone I | Diterpenoids | P35968 | 0.105052994 | 26 |
| 1875 | tanshinone I | Diterpenoids | P48039 | 0.101354935 | 19 |
| 1876 | tanshinone I | Diterpenoids | P49286 | 0.101354935 | 19 |
| 1877 | tanshinone I | Diterpenoids | Q06124 | 0.211032909 | 40 |
| 1878 | tanshinone I | Diterpenoids | Q09028 Q16576 O75530 Q15022 Q15910 | 0.142197972 | 14 |
| 1879 | tanshinone I | Diterpenoids | Q86V86 | 0.100967138 | 7 |
| 1880 | tanshinone I | Diterpenoids | Q99572 | 0.10437669 | 25 |
| 1881 | tanshinone I | Diterpenoids | Q9P1W9 | 0.103344117 | 12 |
| 1882 | tanshinone II A | Diterpenoids | O00519 | 0.110352709 | 16 |
| 1883 | tanshinone II A | Diterpenoids | O00748 | 0.259386615 | 47 |
| 1884 | tanshinone II A | Diterpenoids | O14746 | 0.14728518 | 27 |
| 1885 | tanshinone II A | Diterpenoids | O43613 | 0.10159278 | 9 |
| 1886 | tanshinone II A | Diterpenoids | O43614 | 0.100916735 | 11 |
| 1887 | tanshinone II A | Diterpenoids | O60885 | 0.104476685 | 6 |
| 1888 | tanshinone II A | Diterpenoids | O75530 Q15022 Q15910 | 0.341964133 | 19 |
| 1889 | tanshinone II A | Diterpenoids | O76074 | 0.103099774 | 12 |
| 1890 | tanshinone II A | Diterpenoids | P06401 | 0.108868706 | 15 |
| 1891 | tanshinone II A | Diterpenoids | P08575 | 0.109148612 | 27 |
| 1892 | tanshinone II A | Diterpenoids | P09917 | 0.106782208 | 14 |
| 1893 | tanshinone II A | Diterpenoids | P10275 | 0.132131799 | 34 |
| 1894 | tanshinone II A | Diterpenoids | P11511 | 0.139056613 | 29 |
| 1895 | tanshinone II A | Diterpenoids | P15121 | 0.267155221 | 39 |
| 1896 | tanshinone II A | Diterpenoids | P22460 | 0.106959422 | 3 |
| 1897 | tanshinone II A | Diterpenoids | P22736 | 0.100039999 | 6 |
| 1898 | tanshinone II A | Diterpenoids | P23141 | 0.275271523 | 31 |
| 1899 | tanshinone II A | Diterpenoids | P25440 | 0.106747907 | 3 |
| 1900 | tanshinone II A | Diterpenoids | P25774 | 0.106840916 | 6 |
| 1901 | tanshinone II A | Diterpenoids | P28845 | 0.253303956 | 34 |
| 1902 | tanshinone II A | Diterpenoids | P29350 | 0.204438393 | 44 |
| 1903 | tanshinone II A | Diterpenoids | P30304 | 0.109145376 | 25 |
| 1904 | tanshinone II A | Diterpenoids | P30305 | 0.185734372 | 40 |
| 1905 | tanshinone II A | Diterpenoids | P30307 | 0.105546712 | 12 |
| 1906 | tanshinone II A | Diterpenoids | P30536 | 0.102095712 | 5 |
| 1907 | tanshinone II A | Diterpenoids | P32247 | 0.103071649 | 5 |
| 1908 | tanshinone II A | Diterpenoids | P34949 | 0.105190036 | 9 |
| 1909 | tanshinone II A | Diterpenoids | P41594 | 0.103693674 | 22 |
| 1910 | tanshinone II A | Diterpenoids | P48039 | 0.101354935 | 19 |
| 1911 | tanshinone II A | Diterpenoids | P49137 | 0.107101597 | 6 |
| 1912 | tanshinone II A | Diterpenoids | P49286 | 0.101354935 | 19 |
| 1913 | tanshinone II A | Diterpenoids | P50406 | 0.106847218 | 8 |
| 1914 | tanshinone II A | Diterpenoids | Q01959 | 0.107731401 | 12 |
| 1915 | tanshinone II A | Diterpenoids | Q06124 | 0.211032909 | 40 |
| 1916 | tanshinone II A | Diterpenoids | Q09028 Q16576 O75530 Q15022 Q15910 | 0.142197972 | 14 |
| 1917 | tanshinone II A | Diterpenoids | Q15059 | 0.106747907 | 3 |
| 1918 | tanshinone II A | Diterpenoids | Q96GD4 | 0.103151027 | 4 |
| 1919 | tanshinone II A | Diterpenoids | Q99572 | 0.10437669 | 25 |
| 1920 | tanshinone II A | Diterpenoids | Q9Y233 | 0.108085897 | 14 |
| 1921 | tanshinone II A | Diterpenoids | Q9Y5N1 | 0.101047212 | 6 |
| 1922 | tanshinone II B | Diterpenoids | O00408 | 0.103882897 | 9 |
| 1923 | tanshinone II B | Diterpenoids | O00748 | 0.259386615 | 47 |
| 1924 | tanshinone II B | Diterpenoids | O14746 | 0.14728518 | 27 |
| 1925 | tanshinone II B | Diterpenoids | O14920 | 0.104909858 | 3 |
| 1926 | tanshinone II B | Diterpenoids | O15530 | 0.110104207 | 4 |
| 1927 | tanshinone II B | Diterpenoids | O60674 | 0.103305945 | 11 |
| 1928 | tanshinone II B | Diterpenoids | O75460 | 0.105470076 | 5 |
| 1929 | tanshinone II B | Diterpenoids | O75530 Q15022 Q15910 | 0.341964133 | 19 |
| 1930 | tanshinone II B | Diterpenoids | O96017 | 0.109638321 | 3 |
| 1931 | tanshinone II B | Diterpenoids | P00519 | 0.104821195 | 5 |
| 1932 | tanshinone II B | Diterpenoids | P00533 | 0.104151342 | 24 |
| 1933 | tanshinone II B | Diterpenoids | P04629 | 0.107046621 | 8 |
| 1934 | tanshinone II B | Diterpenoids | P06213 | 0.103151027 | 4 |
| 1935 | tanshinone II B | Diterpenoids | P06239 | 0.102843593 | 5 |
| 1936 | tanshinone II B | Diterpenoids | P06737 | 0.103331553 | 10 |
| 1937 | tanshinone II B | Diterpenoids | P07333 | 0.104063456 | 9 |
| 1938 | tanshinone II B | Diterpenoids | P07947 | 0.101613855 | 3 |
| 1939 | tanshinone II B | Diterpenoids | P07949 | 0.103471701 | 8 |
| 1940 | tanshinone II B | Diterpenoids | P08575 | 0.109148612 | 27 |
| 1941 | tanshinone II B | Diterpenoids | P10721 | 0.104909858 | 3 |
| 1942 | tanshinone II B | Diterpenoids | P11309 | 0.104805981 | 13 |
| 1943 | tanshinone II B | Diterpenoids | P12931 | 0.104821195 | 5 |
| 1944 | tanshinone II B | Diterpenoids | P15121 | 0.267155221 | 39 |
| 1945 | tanshinone II B | Diterpenoids | P16234 | 0.100367415 | 3 |
| 1946 | tanshinone II B | Diterpenoids | P17948 | 0.105554897 | 13 |
| 1947 | tanshinone II B | Diterpenoids | P21554 | 0.104626059 | 10 |
| 1948 | tanshinone II B | Diterpenoids | P23141 | 0.275271523 | 31 |
| 1949 | tanshinone II B | Diterpenoids | P23458 | 0.102916912 | 11 |
| 1950 | tanshinone II B | Diterpenoids | P24941 | 0.106080884 | 8 |
| 1951 | tanshinone II B | Diterpenoids | P28845 | 0.253303956 | 34 |
| 1952 | tanshinone II B | Diterpenoids | P29350 | 0.204438393 | 44 |
| 1953 | tanshinone II B | Diterpenoids | P30304 | 0.109145376 | 25 |
| 1954 | tanshinone II B | Diterpenoids | P30305 | 0.185734372 | 40 |
| 1955 | tanshinone II B | Diterpenoids | P30307 | 0.105546712 | 12 |
| 1956 | tanshinone II B | Diterpenoids | P34972 | 0.103169308 | 13 |
| 1957 | tanshinone II B | Diterpenoids | P35916 | 0.105748254 | 5 |
| 1958 | tanshinone II B | Diterpenoids | P35968 | 0.105052994 | 26 |
| 1959 | tanshinone II B | Diterpenoids | P36888 | 0.102892289 | 8 |
| 1960 | tanshinone II B | Diterpenoids | P41145 | 0.107282863 | 6 |
| 1961 | tanshinone II B | Diterpenoids | P41594 | 0.103693674 | 22 |
| 1962 | tanshinone II B | Diterpenoids | P49354 P49356 | 0.109904857 | 24 |
| 1963 | tanshinone II B | Diterpenoids | P51812 | 0.135853345 | 4 |
| 1964 | tanshinone II B | Diterpenoids | P52333 | 0.104243408 | 12 |
| 1965 | tanshinone II B | Diterpenoids | P53609 P49354 | 0.101613855 | 3 |
| 1966 | tanshinone II B | Diterpenoids | P53779 | 0.103177433 | 9 |
| 1967 | tanshinone II B | Diterpenoids | P78527 | 0.104180881 | 6 |
| 1968 | tanshinone II B | Diterpenoids | Q02750 | 0.106463868 | 11 |
| 1969 | tanshinone II B | Diterpenoids | Q05397 | 0.104909858 | 3 |
| 1970 | tanshinone II B | Diterpenoids | Q06124 | 0.211032909 | 40 |
| 1971 | tanshinone II B | Diterpenoids | Q09028 Q16576 O75530 Q15022 Q15910 | 0.142197972 | 14 |
| 1972 | tanshinone II B | Diterpenoids | Q13627 | 0.104383674 | 7 |
| 1973 | tanshinone II B | Diterpenoids | Q15078 Q00535 | 0.102910354 | 14 |
| 1974 | tanshinone II B | Diterpenoids | Q15746 | 0.102744609 | 5 |
| 1975 | tanshinone II B | Diterpenoids | Q16584 | 0.104189154 | 3 |
| 1976 | tanshinone II B | Diterpenoids | Q5S007 | 0.102368749 | 9 |
| 1977 | tanshinone II B | Diterpenoids | Q96GD4 | 0.103151027 | 4 |
| 1978 | tanshinone II B | Diterpenoids | Q9NWZ3 | 0.105136637 | 5 |
| 1979 | tanshinone II B | Diterpenoids | Q9P1W9 | 0.103344117 | 12 |
| 1980 | tanshinone II B | Diterpenoids | Q9Y233 | 0.108085897 | 14 |
| 1981 | tanshinone VI | Diterpenoids | O00748 | 0.259386615 | 47 |
| 1982 | tanshinone VI | Diterpenoids | O14920 | 0.104909858 | 3 |
| 1983 | tanshinone VI | Diterpenoids | O95551 | 0.106334502 | 10 |
| 1984 | tanshinone VI | Diterpenoids | P00338 | 0.106699814 | 5 |
| 1985 | tanshinone VI | Diterpenoids | P00533 | 0.104151342 | 24 |
| 1986 | tanshinone VI | Diterpenoids | P06737 | 0.103331553 | 10 |
| 1987 | tanshinone VI | Diterpenoids | P08575 | 0.109148612 | 27 |
| 1988 | tanshinone VI | Diterpenoids | P08842 | 0.103451903 | 3 |
| 1989 | tanshinone VI | Diterpenoids | P11387 | 0.113455556 | 13 |
| 1990 | tanshinone VI | Diterpenoids | P14902 | 0.110454469 | 24 |
| 1991 | tanshinone VI | Diterpenoids | P15121 | 0.267155221 | 39 |
| 1992 | tanshinone VI | Diterpenoids | P22303 | 0.287353637 | 26 |
| 1993 | tanshinone VI | Diterpenoids | P23141 | 0.275271523 | 31 |
| 1994 | tanshinone VI | Diterpenoids | P25101 | 0.104008389 | 6 |
| 1995 | tanshinone VI | Diterpenoids | P27338 | 0.105021471 | 16 |
| 1996 | tanshinone VI | Diterpenoids | P28562 | 0.104003317 | 12 |
| 1997 | tanshinone VI | Diterpenoids | P29350 | 0.204438393 | 44 |
| 1998 | tanshinone VI | Diterpenoids | P30304 | 0.109145376 | 25 |
| 1999 | tanshinone VI | Diterpenoids | P30305 | 0.185734372 | 40 |
| 2000 | tanshinone VI | Diterpenoids | P31639 | 0.109951316 | 5 |
| 2001 | tanshinone VI | Diterpenoids | P37231 | 0.111742909 | 15 |
| 2002 | tanshinone VI | Diterpenoids | P45983 | 0.105088123 | 17 |
| 2003 | tanshinone VI | Diterpenoids | P49354 P49356 | 0.109904857 | 24 |
| 2004 | tanshinone VI | Diterpenoids | Q01432 | 0.101993948 | 3 |
| 2005 | tanshinone VI | Diterpenoids | Q01433 | 0.110908758 | 3 |
| 2006 | tanshinone VI | Diterpenoids | Q02127 | 0.106699814 | 5 |
| 2007 | tanshinone VI | Diterpenoids | Q06124 | 0.211032909 | 40 |
| 2008 | tanshinone VI | Diterpenoids | Q07820 | 0.101122731 | 4 |
| 2009 | tanshinone VI | Diterpenoids | Q07869 | 0.112467055 | 11 |
| 2010 | tanshinone VI | Diterpenoids | Q16875 | 0.101683939 | 4 |
| 2011 | tanshinone VI | Diterpenoids | Q9UDY8 | 0.107158923 | 10 |
| 2012 | ailanthoidol | Others | O14684 | 0.116389783 | 19 |
| 2013 | ailanthoidol | Others | O14757 | 0.106739614 | 8 |
| 2014 | ailanthoidol | Others | O60674 | 0.103305945 | 11 |
| 2015 | ailanthoidol | Others | P00797 | 0.104471265 | 3 |
| 2016 | ailanthoidol | Others | P03372 | 0.132037828 | 16 |
| 2017 | ailanthoidol | Others | P07333 | 0.104063456 | 9 |
| 2018 | ailanthoidol | Others | P07949 | 0.103471701 | 8 |
| 2019 | ailanthoidol | Others | P11362 | 0.105847024 | 4 |
| 2020 | ailanthoidol | Others | P15121 | 0.267155221 | 39 |
| 2021 | ailanthoidol | Others | P17252 | 0.102754312 | 7 |
| 2022 | ailanthoidol | Others | P17948 | 0.105554897 | 13 |
| 2023 | ailanthoidol | Others | P20248 P24941 | 0.112800203 | 4 |
| 2024 | ailanthoidol | Others | P20701 | 0.127026321 | 4 |
| 2025 | ailanthoidol | Others | P21728 | 0.105764248 | 3 |
| 2026 | ailanthoidol | Others | P23458 | 0.102916912 | 11 |
| 2027 | ailanthoidol | Others | P27487 | 0.104386227 | 3 |
| 2028 | ailanthoidol | Others | P30291 | 0.103962076 | 3 |
| 2029 | ailanthoidol | Others | P31751 | 0.110657683 | 3 |
| 2030 | ailanthoidol | Others | P35462 | 0.105764248 | 3 |
| 2031 | ailanthoidol | Others | P35916 | 0.105748254 | 5 |
| 2032 | ailanthoidol | Others | P37023 | 0.111434828 | 4 |
| 2033 | ailanthoidol | Others | P42336 | 0.108042586 | 13 |
| 2034 | ailanthoidol | Others | P42345 | 0.10907142 | 6 |
| 2035 | ailanthoidol | Others | P45983 | 0.105088123 | 17 |
| 2036 | ailanthoidol | Others | P45984 | 0.10939337 | 4 |
| 2037 | ailanthoidol | Others | Q00796 | 0.107710558 | 5 |
| 2038 | ailanthoidol | Others | Q12809 | 0.105584236 | 3 |
| 2039 | ailanthoidol | Others | Q12866 | 0.103756913 | 4 |
| 2040 | ailanthoidol | Others | Q16539 | 0.105438848 | 20 |
| 2041 | ailanthoidol | Others | Q92731 | 0.117586956 | 23 |
| 2042 | ailanthoidol | Others | Q9HBH9 | 0.10939337 | 4 |
| 2043 | daucosterol (Sitogluside,alexandrin) | Steroids | P17706 | 0.347170021 | 15 |
| 2044 | daucosterol (Sitogluside,alexandrin) | Steroids | P18031 | 0.474772974 | 23 |
| 2045 | daucosterol (Sitogluside,alexandrin) | Steroids | P24666 | 0.343119127 | 13 |
| 2046 | daucosterol (Sitogluside,alexandrin) | Steroids | P30305 | 0.185734372 | 40 |
| 2047 | daucosterol (Sitogluside,alexandrin) | Steroids | P40763 | 0.268996335 | 13 |
| 2048 | daucosterol (Sitogluside,alexandrin) | Steroids | P49810 Q9NZ42 Q92542 Q96BI3 P49768 Q8WW43 | 0.101543467 | 9 |
| 2049 | daucosterol (Sitogluside,alexandrin) | Steroids | P80365 | 0.109471969 | 12 |
| 2050 | daucosterol (Sitogluside,alexandrin) | Steroids | Q16875 | 0.101683939 | 4 |
| 2051 | gamma-sitosterol | Steroids | O00748 | 0.259386615 | 47 |
| 2052 | gamma-sitosterol | Steroids | O14684 | 0.116389783 | 19 |
| 2053 | gamma-sitosterol | Steroids | P03372 | 0.132037828 | 16 |
| 2054 | gamma-sitosterol | Steroids | P04035 | 0.166864971 | 20 |
| 2055 | gamma-sitosterol | Steroids | P04278 | 0.16719946 | 19 |
| 2056 | gamma-sitosterol | Steroids | P05093 | 0.148009819 | 20 |
| 2057 | gamma-sitosterol | Steroids | P06276 | 0.130116287 | 14 |
| 2058 | gamma-sitosterol | Steroids | P06746 | 0.307158581 | 16 |
| 2059 | gamma-sitosterol | Steroids | P08172 | 0.117318329 | 18 |
| 2060 | gamma-sitosterol | Steroids | P08185 | 0.113039089 | 17 |
| 2061 | gamma-sitosterol | Steroids | P10275 | 0.132131799 | 34 |
| 2062 | gamma-sitosterol | Steroids | P11413 | 0.113579529 | 12 |
| 2063 | gamma-sitosterol | Steroids | P11473 | 0.109776491 | 5 |
| 2064 | gamma-sitosterol | Steroids | P11511 | 0.139056613 | 29 |
| 2065 | gamma-sitosterol | Steroids | P16662 | 0.109345148 | 4 |
| 2066 | gamma-sitosterol | Steroids | P17706 | 0.347170021 | 15 |
| 2067 | gamma-sitosterol | Steroids | P18031 | 0.474772974 | 23 |
| 2068 | gamma-sitosterol | Steroids | P22303 | 0.287353637 | 26 |
| 2069 | gamma-sitosterol | Steroids | P23975 | 0.115012308 | 10 |
| 2070 | gamma-sitosterol | Steroids | P28845 | 0.253303956 | 34 |
| 2071 | gamma-sitosterol | Steroids | P29350 | 0.204438393 | 44 |
| 2072 | gamma-sitosterol | Steroids | P31645 | 0.1201852 | 17 |
| 2073 | gamma-sitosterol | Steroids | P33261 | 0.124903734 | 16 |
| 2074 | gamma-sitosterol | Steroids | P34995 | 0.114009578 | 9 |
| 2075 | gamma-sitosterol | Steroids | P35228 | 0.112440313 | 15 |
| 2076 | gamma-sitosterol | Steroids | P35398 | 0.130342003 | 12 |
| 2077 | gamma-sitosterol | Steroids | P37231 | 0.111742909 | 15 |
| 2078 | gamma-sitosterol | Steroids | P37268 | 0.110958418 | 7 |
| 2079 | gamma-sitosterol | Steroids | P43116 | 0.114821812 | 13 |
| 2080 | gamma-sitosterol | Steroids | P51449 | 0.406390508 | 15 |
| 2081 | gamma-sitosterol | Steroids | P55055 | 0.106165761 | 3 |
| 2082 | gamma-sitosterol | Steroids | Q03181 | 0.111670964 | 14 |
| 2083 | gamma-sitosterol | Steroids | Q12772 | 0.216727786 | 7 |
| 2084 | gamma-sitosterol | Steroids | Q13133 | 0.216325973 | 16 |
| 2085 | gamma-sitosterol | Steroids | Q14534 | 0.110691545 | 6 |
| 2086 | gamma-sitosterol | Steroids | Q14994 | 0.12030258 | 8 |
| 2087 | gamma-sitosterol | Steroids | Q16850 | 0.152847021 | 14 |
| 2088 | gamma-sitosterol | Steroids | Q92731 | 0.117586956 | 23 |
| 2089 | gamma-sitosterol | Steroids | Q9UBM7 | 0.106165761 | 3 |
| 2090 | gamma-sitosterol | Steroids | Q9UHC9 | 0.28130208 | 13 |
| 2091 | isocucurbitacin d | Steroids | O00748 | 0.259386615 | 47 |
| 2092 | isocucurbitacin d | Steroids | O14757 | 0.106739614 | 8 |
| 2093 | isocucurbitacin d | Steroids | O14965 | 0.107316885 | 8 |
| 2094 | isocucurbitacin d | Steroids | O15530 | 0.110104207 | 4 |
| 2095 | isocucurbitacin d | Steroids | O96017 | 0.109638321 | 3 |
| 2096 | isocucurbitacin d | Steroids | P00742 | 0.111476369 | 3 |
| 2097 | isocucurbitacin d | Steroids | P01375 | 0.108038789 | 7 |
| 2098 | isocucurbitacin d | Steroids | P03956 | 0.109842942 | 8 |
| 2099 | isocucurbitacin d | Steroids | P04150 | 0.109500234 | 17 |
| 2100 | isocucurbitacin d | Steroids | P04278 | 0.16719946 | 19 |
| 2101 | isocucurbitacin d | Steroids | P04629 | 0.107046621 | 8 |
| 2102 | isocucurbitacin d | Steroids | P04818 | 0.111100851 | 4 |
| 2103 | isocucurbitacin d | Steroids | P05093 | 0.148009819 | 20 |
| 2104 | isocucurbitacin d | Steroids | P06401 | 0.108868706 | 15 |
| 2105 | isocucurbitacin d | Steroids | P06746 | 0.307158581 | 16 |
| 2106 | isocucurbitacin d | Steroids | P07384 | 0.10807591 | 4 |
| 2107 | isocucurbitacin d | Steroids | P08069 | 0.112934324 | 3 |
| 2108 | isocucurbitacin d | Steroids | P08185 | 0.113039089 | 17 |
| 2109 | isocucurbitacin d | Steroids | P08235 | 0.107998327 | 12 |
| 2110 | isocucurbitacin d | Steroids | P08254 | 0.109296209 | 8 |
| 2111 | isocucurbitacin d | Steroids | P08473 | 0.108391881 | 3 |
| 2112 | isocucurbitacin d | Steroids | P08581 | 0.109668623 | 6 |
| 2113 | isocucurbitacin d | Steroids | P09917 | 0.106782208 | 14 |
| 2114 | isocucurbitacin d | Steroids | P0DMS8 | 0.10464569 | 23 |
| 2115 | isocucurbitacin d | Steroids | P10275 | 0.132131799 | 34 |
| 2116 | isocucurbitacin d | Steroids | P11309 | 0.104805981 | 13 |
| 2117 | isocucurbitacin d | Steroids | P11413 | 0.113579529 | 12 |
| 2118 | isocucurbitacin d | Steroids | P11511 | 0.139056613 | 29 |
| 2119 | isocucurbitacin d | Steroids | P14416 | 0.108887011 | 4 |
| 2120 | isocucurbitacin d | Steroids | P17948 | 0.105554897 | 13 |
| 2121 | isocucurbitacin d | Steroids | P18031 | 0.474772974 | 23 |
| 2122 | isocucurbitacin d | Steroids | P20248 P24941 | 0.112800203 | 4 |
| 2123 | isocucurbitacin d | Steroids | P20701 | 0.127026321 | 4 |
| 2124 | isocucurbitacin d | Steroids | P22894 | 0.111100851 | 4 |
| 2125 | isocucurbitacin d | Steroids | P24723 | 0.11185669 | 11 |
| 2126 | isocucurbitacin d | Steroids | P24941 | 0.106080884 | 8 |
| 2127 | isocucurbitacin d | Steroids | P24941 P78396 P20248 | 0.108328679 | 5 |
| 2128 | isocucurbitacin d | Steroids | P25774 | 0.106840916 | 6 |
| 2129 | isocucurbitacin d | Steroids | P28482 | 0.111100851 | 4 |
| 2130 | isocucurbitacin d | Steroids | P28845 | 0.253303956 | 34 |
| 2131 | isocucurbitacin d | Steroids | P31639 | 0.109951316 | 5 |
| 2132 | isocucurbitacin d | Steroids | P31645 | 0.1201852 | 17 |
| 2133 | isocucurbitacin d | Steroids | P31751 | 0.110657683 | 3 |
| 2134 | isocucurbitacin d | Steroids | P32246 | 0.109647095 | 6 |
| 2135 | isocucurbitacin d | Steroids | P34995 | 0.114009578 | 9 |
| 2136 | isocucurbitacin d | Steroids | P34998 | 0.110755665 | 3 |
| 2137 | isocucurbitacin d | Steroids | P35228 | 0.112440313 | 15 |
| 2138 | isocucurbitacin d | Steroids | P35354 | 0.113487837 | 23 |
| 2139 | isocucurbitacin d | Steroids | P35398 | 0.130342003 | 12 |
| 2140 | isocucurbitacin d | Steroids | P35408 | 0.113274443 | 9 |
| 2141 | isocucurbitacin d | Steroids | P35968 | 0.105052994 | 26 |
| 2142 | isocucurbitacin d | Steroids | P37231 | 0.111742909 | 15 |
| 2143 | isocucurbitacin d | Steroids | P37268 | 0.110958418 | 7 |
| 2144 | isocucurbitacin d | Steroids | P40763 | 0.268996335 | 13 |
| 2145 | isocucurbitacin d | Steroids | P42336 | 0.108042586 | 13 |
| 2146 | isocucurbitacin d | Steroids | P42338 | 0.107541648 | 9 |
| 2147 | isocucurbitacin d | Steroids | P42345 | 0.10907142 | 6 |
| 2148 | isocucurbitacin d | Steroids | P43088 | 0.115549923 | 5 |
| 2149 | isocucurbitacin d | Steroids | P43115 | 0.11287548 | 5 |
| 2150 | isocucurbitacin d | Steroids | P43116 | 0.114821812 | 13 |
| 2151 | isocucurbitacin d | Steroids | P43119 | 0.112265692 | 10 |
| 2152 | isocucurbitacin d | Steroids | P43405 | 0.106351077 | 5 |
| 2153 | isocucurbitacin d | Steroids | P45452 | 0.106586322 | 6 |
| 2154 | isocucurbitacin d | Steroids | P45983 | 0.105088123 | 17 |
| 2155 | isocucurbitacin d | Steroids | P45984 | 0.10939337 | 4 |
| 2156 | isocucurbitacin d | Steroids | P49137 | 0.107101597 | 6 |
| 2157 | isocucurbitacin d | Steroids | P49354 P49356 | 0.109904857 | 24 |
| 2158 | isocucurbitacin d | Steroids | P49810 Q9NZ42 Q92542 Q96BI3 P49768 Q8WW43 | 0.101543467 | 9 |
| 2159 | isocucurbitacin d | Steroids | P54760 | 0.111687884 | 3 |
| 2160 | isocucurbitacin d | Steroids | P56373 | 0.107098941 | 5 |
| 2161 | isocucurbitacin d | Steroids | P56817 | 0.108325485 | 12 |
| 2162 | isocucurbitacin d | Steroids | Q00796 | 0.107710558 | 5 |
| 2163 | isocucurbitacin d | Steroids | Q01959 | 0.107731401 | 12 |
| 2164 | isocucurbitacin d | Steroids | Q02750 | 0.106463868 | 11 |
| 2165 | isocucurbitacin d | Steroids | Q03181 | 0.111670964 | 14 |
| 2166 | isocucurbitacin d | Steroids | Q06124 | 0.211032909 | 40 |
| 2167 | isocucurbitacin d | Steroids | Q07869 | 0.112467055 | 11 |
| 2168 | isocucurbitacin d | Steroids | Q13133 | 0.216325973 | 16 |
| 2169 | isocucurbitacin d | Steroids | Q13547 | 0.110937969 | 6 |
| 2170 | isocucurbitacin d | Steroids | Q16539 | 0.105438848 | 20 |
| 2171 | isocucurbitacin d | Steroids | Q8IXJ6 | 0.108888011 | 5 |
| 2172 | isocucurbitacin d | Steroids | Q92731 | 0.117586956 | 23 |
| 2173 | isocucurbitacin d | Steroids | Q99720 | 0.110060881 | 8 |
| 2174 | isocucurbitacin d | Steroids | Q9HBH9 | 0.10939337 | 4 |
| 2175 | isocucurbitacin d | Steroids | Q9UHC9 | 0.28130208 | 13 |
| 2176 | isocucurbitacin d | Steroids | Q9Y233 | 0.108085897 | 14 |
| 2177 | rutin | Steroids | O14746 | 0.14728518 | 27 |
| 2178 | rutin | Steroids | O43570 | 0.137972322 | 10 |
| 2179 | rutin | Steroids | O76074 | 0.103099774 | 12 |
| 2180 | rutin | Steroids | P00918 | 0.141292356 | 8 |
| 2181 | rutin | Steroids | P01375 | 0.108038789 | 7 |
| 2182 | rutin | Steroids | P09917 | 0.106782208 | 14 |
| 2183 | rutin | Steroids | P15121 | 0.267155221 | 39 |
| 2184 | rutin | Steroids | P16083 | 0.112425971 | 14 |
| 2185 | rutin | Steroids | P22303 | 0.287353637 | 26 |
| 2186 | rutin | Steroids | P22748 | 0.219188074 | 3 |
| 2187 | rutin | Steroids | P30542 | 0.101318267 | 24 |
| 2188 | rutin | Steroids | P35354 | 0.113487837 | 23 |
| 2189 | rutin | Steroids | P43166 | 0.165991461 | 6 |
| 2190 | rutin | Steroids | P51812 | 0.135853345 | 4 |
| 2191 | stigmasterol | Steroids | O14684 | 0.116389783 | 19 |
| 2192 | stigmasterol | Steroids | P03372 | 0.132037828 | 16 |
| 2193 | stigmasterol | Steroids | P04035 | 0.166864971 | 20 |
| 2194 | stigmasterol | Steroids | P04278 | 0.16719946 | 19 |
| 2195 | stigmasterol | Steroids | P05093 | 0.148009819 | 20 |
| 2196 | stigmasterol | Steroids | P06276 | 0.130116287 | 14 |
| 2197 | stigmasterol | Steroids | P06746 | 0.307158581 | 16 |
| 2198 | stigmasterol | Steroids | P08172 | 0.117318329 | 18 |
| 2199 | stigmasterol | Steroids | P08185 | 0.113039089 | 17 |
| 2200 | stigmasterol | Steroids | P10275 | 0.132131799 | 34 |
| 2201 | stigmasterol | Steroids | P11413 | 0.113579529 | 12 |
| 2202 | stigmasterol | Steroids | P11473 | 0.109776491 | 5 |
| 2203 | stigmasterol | Steroids | P11511 | 0.139056613 | 29 |
| 2204 | stigmasterol | Steroids | P16662 | 0.109345148 | 4 |
| 2205 | stigmasterol | Steroids | P17706 | 0.347170021 | 15 |
| 2206 | stigmasterol | Steroids | P18031 | 0.474772974 | 23 |
| 2207 | stigmasterol | Steroids | P22303 | 0.287353637 | 26 |
| 2208 | stigmasterol | Steroids | P23975 | 0.115012308 | 10 |
| 2209 | stigmasterol | Steroids | P24557 | 0.107944463 | 3 |
| 2210 | stigmasterol | Steroids | P28845 | 0.253303956 | 34 |
| 2211 | stigmasterol | Steroids | P29350 | 0.204438393 | 44 |
| 2212 | stigmasterol | Steroids | P31645 | 0.1201852 | 17 |
| 2213 | stigmasterol | Steroids | P33261 | 0.124903734 | 16 |
| 2214 | stigmasterol | Steroids | P34995 | 0.114009578 | 9 |
| 2215 | stigmasterol | Steroids | P35228 | 0.112440313 | 15 |
| 2216 | stigmasterol | Steroids | P35398 | 0.130342003 | 12 |
| 2217 | stigmasterol | Steroids | P37231 | 0.111742909 | 15 |
| 2218 | stigmasterol | Steroids | P37268 | 0.110958418 | 7 |
| 2219 | stigmasterol | Steroids | P43116 | 0.114821812 | 13 |
| 2220 | stigmasterol | Steroids | P51449 | 0.406390508 | 15 |
| 2221 | stigmasterol | Steroids | P55055 | 0.106165761 | 3 |
| 2222 | stigmasterol | Steroids | Q03181 | 0.111670964 | 14 |
| 2223 | stigmasterol | Steroids | Q07869 | 0.112467055 | 11 |
| 2224 | stigmasterol | Steroids | Q12772 | 0.216727786 | 7 |
| 2225 | stigmasterol | Steroids | Q13133 | 0.216325973 | 16 |
| 2226 | stigmasterol | Steroids | Q14534 | 0.110691545 | 6 |
| 2227 | stigmasterol | Steroids | Q14994 | 0.12030258 | 8 |
| 2228 | stigmasterol | Steroids | Q16850 | 0.152847021 | 14 |
| 2229 | stigmasterol | Steroids | Q92731 | 0.117586956 | 23 |
| 2230 | stigmasterol | Steroids | Q9UBM7 | 0.106165761 | 3 |
| 2231 | stigmasterol | Steroids | Q9UHC9 | 0.28130208 | 13 |
| 2232 | β-sitosterol | Steroids | O00748 | 0.259386615 | 47 |
| 2233 | β-sitosterol | Steroids | O14684 | 0.116389783 | 19 |
| 2234 | β-sitosterol | Steroids | P03372 | 0.132037828 | 16 |
| 2235 | β-sitosterol | Steroids | P04035 | 0.166864971 | 20 |
| 2236 | β-sitosterol | Steroids | P04278 | 0.16719946 | 19 |
| 2237 | β-sitosterol | Steroids | P05093 | 0.148009819 | 20 |
| 2238 | β-sitosterol | Steroids | P06276 | 0.130116287 | 14 |
| 2239 | β-sitosterol | Steroids | P06746 | 0.307158581 | 16 |
| 2240 | β-sitosterol | Steroids | P08172 | 0.117318329 | 18 |
| 2241 | β-sitosterol | Steroids | P08185 | 0.113039089 | 17 |
| 2242 | β-sitosterol | Steroids | P10275 | 0.132131799 | 34 |
| 2243 | β-sitosterol | Steroids | P11413 | 0.113579529 | 12 |
| 2244 | β-sitosterol | Steroids | P11473 | 0.109776491 | 5 |
| 2245 | β-sitosterol | Steroids | P11511 | 0.139056613 | 29 |
| 2246 | β-sitosterol | Steroids | P16662 | 0.109345148 | 4 |
| 2247 | β-sitosterol | Steroids | P17706 | 0.347170021 | 15 |
| 2248 | β-sitosterol | Steroids | P18031 | 0.474772974 | 23 |
| 2249 | β-sitosterol | Steroids | P22303 | 0.287353637 | 26 |
| 2250 | β-sitosterol | Steroids | P23975 | 0.115012308 | 10 |
| 2251 | β-sitosterol | Steroids | P24557 | 0.107944463 | 3 |
| 2252 | β-sitosterol | Steroids | P28845 | 0.253303956 | 34 |
| 2253 | β-sitosterol | Steroids | P29350 | 0.204438393 | 44 |
| 2254 | β-sitosterol | Steroids | P31645 | 0.1201852 | 17 |
| 2255 | β-sitosterol | Steroids | P33261 | 0.124903734 | 16 |
| 2256 | β-sitosterol | Steroids | P34995 | 0.114009578 | 9 |
| 2257 | β-sitosterol | Steroids | P35228 | 0.112440313 | 15 |
| 2258 | β-sitosterol | Steroids | P35398 | 0.130342003 | 12 |
| 2259 | β-sitosterol | Steroids | P37231 | 0.111742909 | 15 |
| 2260 | β-sitosterol | Steroids | P43116 | 0.114821812 | 13 |
| 2261 | β-sitosterol | Steroids | P51449 | 0.406390508 | 15 |
| 2262 | β-sitosterol | Steroids | P55055 | 0.106165761 | 3 |
| 2263 | β-sitosterol | Steroids | Q03181 | 0.111670964 | 14 |
| 2264 | β-sitosterol | Steroids | Q12772 | 0.216727786 | 7 |
| 2265 | β-sitosterol | Steroids | Q13133 | 0.216325973 | 16 |
| 2266 | β-sitosterol | Steroids | Q14534 | 0.110691545 | 6 |
| 2267 | β-sitosterol | Steroids | Q14994 | 0.12030258 | 8 |
| 2268 | β-sitosterol | Steroids | Q16850 | 0.152847021 | 14 |
| 2269 | β-sitosterol | Steroids | Q92731 | 0.117586956 | 23 |
| 2270 | β-sitosterol | Steroids | Q9UBM7 | 0.106165761 | 3 |
| 2271 | β-sitosterol | Steroids | Q9UHC9 | 0.28130208 | 13 |
| 2272 | 3-O-acetyloleanolic acid (Acetyl oleanolic acid, Acetyloleanolic acid) | Triterpenoids | O00519 | 0.110352709 | 16 |
| 2273 | 3-O-acetyloleanolic acid (Acetyl oleanolic acid, Acetyloleanolic acid) | Triterpenoids | O00748 | 0.259386615 | 47 |
| 2274 | 3-O-acetyloleanolic acid (Acetyl oleanolic acid, Acetyloleanolic acid) | Triterpenoids | O14684 | 0.116389783 | 19 |
| 2275 | 3-O-acetyloleanolic acid (Acetyl oleanolic acid, Acetyloleanolic acid) | Triterpenoids | O60218 | 0.499225177 | 13 |
| 2276 | 3-O-acetyloleanolic acid (Acetyl oleanolic acid, Acetyloleanolic acid) | Triterpenoids | P04035 | 0.166864971 | 20 |
| 2277 | 3-O-acetyloleanolic acid (Acetyl oleanolic acid, Acetyloleanolic acid) | Triterpenoids | P04054 | 0.395466092 | 11 |
| 2278 | 3-O-acetyloleanolic acid (Acetyl oleanolic acid, Acetyloleanolic acid) | Triterpenoids | P04150 | 0.109500234 | 17 |
| 2279 | 3-O-acetyloleanolic acid (Acetyl oleanolic acid, Acetyloleanolic acid) | Triterpenoids | P04278 | 0.16719946 | 19 |
| 2280 | 3-O-acetyloleanolic acid (Acetyl oleanolic acid, Acetyloleanolic acid) | Triterpenoids | P05093 | 0.148009819 | 20 |
| 2281 | 3-O-acetyloleanolic acid (Acetyl oleanolic acid, Acetyloleanolic acid) | Triterpenoids | P06401 | 0.108868706 | 15 |
| 2282 | 3-O-acetyloleanolic acid (Acetyl oleanolic acid, Acetyloleanolic acid) | Triterpenoids | P06746 | 0.307158581 | 16 |
| 2283 | 3-O-acetyloleanolic acid (Acetyl oleanolic acid, Acetyloleanolic acid) | Triterpenoids | P07477 | 0.103976264 | 3 |
| 2284 | 3-O-acetyloleanolic acid (Acetyl oleanolic acid, Acetyloleanolic acid) | Triterpenoids | P08185 | 0.113039089 | 17 |
| 2285 | 3-O-acetyloleanolic acid (Acetyl oleanolic acid, Acetyloleanolic acid) | Triterpenoids | P08235 | 0.107998327 | 12 |
| 2286 | 3-O-acetyloleanolic acid (Acetyl oleanolic acid, Acetyloleanolic acid) | Triterpenoids | P09917 | 0.106782208 | 14 |
| 2287 | 3-O-acetyloleanolic acid (Acetyl oleanolic acid, Acetyloleanolic acid) | Triterpenoids | P10275 | 0.132131799 | 34 |
| 2288 | 3-O-acetyloleanolic acid (Acetyl oleanolic acid, Acetyloleanolic acid) | Triterpenoids | P10586 | 0.312345915 | 15 |
| 2289 | 3-O-acetyloleanolic acid (Acetyl oleanolic acid, Acetyloleanolic acid) | Triterpenoids | P11511 | 0.139056613 | 29 |
| 2290 | 3-O-acetyloleanolic acid (Acetyl oleanolic acid, Acetyloleanolic acid) | Triterpenoids | P14555 | 0.102138215 | 3 |
| 2291 | 3-O-acetyloleanolic acid (Acetyl oleanolic acid, Acetyloleanolic acid) | Triterpenoids | P17252 | 0.102754312 | 7 |
| 2292 | 3-O-acetyloleanolic acid (Acetyl oleanolic acid, Acetyloleanolic acid) | Triterpenoids | P17706 | 0.347170021 | 15 |
| 2293 | 3-O-acetyloleanolic acid (Acetyl oleanolic acid, Acetyloleanolic acid) | Triterpenoids | P18031 | 0.474772974 | 23 |
| 2294 | 3-O-acetyloleanolic acid (Acetyl oleanolic acid, Acetyloleanolic acid) | Triterpenoids | P24666 | 0.343119127 | 13 |
| 2295 | 3-O-acetyloleanolic acid (Acetyl oleanolic acid, Acetyloleanolic acid) | Triterpenoids | P28845 | 0.253303956 | 34 |
| 2296 | 3-O-acetyloleanolic acid (Acetyl oleanolic acid, Acetyloleanolic acid) | Triterpenoids | P30305 | 0.185734372 | 40 |
| 2297 | 3-O-acetyloleanolic acid (Acetyl oleanolic acid, Acetyloleanolic acid) | Triterpenoids | P33261 | 0.124903734 | 16 |
| 2298 | 3-O-acetyloleanolic acid (Acetyl oleanolic acid, Acetyloleanolic acid) | Triterpenoids | P35228 | 0.112440313 | 15 |
| 2299 | 3-O-acetyloleanolic acid (Acetyl oleanolic acid, Acetyloleanolic acid) | Triterpenoids | P35354 | 0.113487837 | 23 |
| 2300 | 3-O-acetyloleanolic acid (Acetyl oleanolic acid, Acetyloleanolic acid) | Triterpenoids | P37059 | 0.106459925 | 6 |
| 2301 | 3-O-acetyloleanolic acid (Acetyl oleanolic acid, Acetyloleanolic acid) | Triterpenoids | P42330 | 0.105243819 | 5 |
| 2302 | 3-O-acetyloleanolic acid (Acetyl oleanolic acid, Acetyloleanolic acid) | Triterpenoids | P43119 | 0.112265692 | 10 |
| 2303 | 3-O-acetyloleanolic acid (Acetyl oleanolic acid, Acetyloleanolic acid) | Triterpenoids | P48147 | 0.107233003 | 22 |
| 2304 | 3-O-acetyloleanolic acid (Acetyl oleanolic acid, Acetyloleanolic acid) | Triterpenoids | P49354 P49356 | 0.109904857 | 24 |
| 2305 | 3-O-acetyloleanolic acid (Acetyl oleanolic acid, Acetyloleanolic acid) | Triterpenoids | P51449 | 0.406390508 | 15 |
| 2306 | 3-O-acetyloleanolic acid (Acetyl oleanolic acid, Acetyloleanolic acid) | Triterpenoids | P80365 | 0.109471969 | 12 |
| 2307 | 3-O-acetyloleanolic acid (Acetyl oleanolic acid, Acetyloleanolic acid) | Triterpenoids | Q05655 | 0.102598878 | 5 |
| 2308 | 3-O-acetyloleanolic acid (Acetyl oleanolic acid, Acetyloleanolic acid) | Triterpenoids | Q08499 | 0.31881944 | 14 |
| 2309 | 3-O-acetyloleanolic acid (Acetyl oleanolic acid, Acetyloleanolic acid) | Triterpenoids | Q13133 | 0.216325973 | 16 |
| 2310 | 3-O-acetyloleanolic acid (Acetyl oleanolic acid, Acetyloleanolic acid) | Triterpenoids | Q9Y5Y4 | 0.113671543 | 7 |
| 2311 | Asiatic acid (Dammarolic acid,Asiantic acid,2alpha,23-Dihydroxyursolic acid) | Triterpenoids | O00519 | 0.110352709 | 16 |
| 2312 | Asiatic acid (Dammarolic acid,Asiantic acid,2alpha,23-Dihydroxyursolic acid) | Triterpenoids | O00748 | 0.259386615 | 47 |
| 2313 | Asiatic acid (Dammarolic acid,Asiantic acid,2alpha,23-Dihydroxyursolic acid) | Triterpenoids | O00767 | 0.113596703 | 10 |
| 2314 | Asiatic acid (Dammarolic acid,Asiantic acid,2alpha,23-Dihydroxyursolic acid) | Triterpenoids | O14684 | 0.116389783 | 19 |
| 2315 | Asiatic acid (Dammarolic acid,Asiantic acid,2alpha,23-Dihydroxyursolic acid) | Triterpenoids | O14746 | 0.14728518 | 27 |
| 2316 | Asiatic acid (Dammarolic acid,Asiantic acid,2alpha,23-Dihydroxyursolic acid) | Triterpenoids | O60218 | 0.499225177 | 13 |
| 2317 | Asiatic acid (Dammarolic acid,Asiantic acid,2alpha,23-Dihydroxyursolic acid) | Triterpenoids | P03372 | 0.132037828 | 16 |
| 2318 | Asiatic acid (Dammarolic acid,Asiantic acid,2alpha,23-Dihydroxyursolic acid) | Triterpenoids | P04035 | 0.166864971 | 20 |
| 2319 | Asiatic acid (Dammarolic acid,Asiantic acid,2alpha,23-Dihydroxyursolic acid) | Triterpenoids | P04054 | 0.395466092 | 11 |
| 2320 | Asiatic acid (Dammarolic acid,Asiantic acid,2alpha,23-Dihydroxyursolic acid) | Triterpenoids | P04150 | 0.109500234 | 17 |
| 2321 | Asiatic acid (Dammarolic acid,Asiantic acid,2alpha,23-Dihydroxyursolic acid) | Triterpenoids | P04278 | 0.16719946 | 19 |
| 2322 | Asiatic acid (Dammarolic acid,Asiantic acid,2alpha,23-Dihydroxyursolic acid) | Triterpenoids | P05093 | 0.148009819 | 20 |
| 2323 | Asiatic acid (Dammarolic acid,Asiantic acid,2alpha,23-Dihydroxyursolic acid) | Triterpenoids | P05413 | 0.111920896 | 10 |
| 2324 | Asiatic acid (Dammarolic acid,Asiantic acid,2alpha,23-Dihydroxyursolic acid) | Triterpenoids | P06401 | 0.108868706 | 15 |
| 2325 | Asiatic acid (Dammarolic acid,Asiantic acid,2alpha,23-Dihydroxyursolic acid) | Triterpenoids | P06746 | 0.307158581 | 16 |
| 2326 | Asiatic acid (Dammarolic acid,Asiantic acid,2alpha,23-Dihydroxyursolic acid) | Triterpenoids | P07148 | 0.115257869 | 10 |
| 2327 | Asiatic acid (Dammarolic acid,Asiantic acid,2alpha,23-Dihydroxyursolic acid) | Triterpenoids | P08185 | 0.113039089 | 17 |
| 2328 | Asiatic acid (Dammarolic acid,Asiantic acid,2alpha,23-Dihydroxyursolic acid) | Triterpenoids | P08235 | 0.107998327 | 12 |
| 2329 | Asiatic acid (Dammarolic acid,Asiantic acid,2alpha,23-Dihydroxyursolic acid) | Triterpenoids | P09917 | 0.106782208 | 14 |
| 2330 | Asiatic acid (Dammarolic acid,Asiantic acid,2alpha,23-Dihydroxyursolic acid) | Triterpenoids | P10275 | 0.132131799 | 34 |
| 2331 | Asiatic acid (Dammarolic acid,Asiantic acid,2alpha,23-Dihydroxyursolic acid) | Triterpenoids | P10586 | 0.312345915 | 15 |
| 2332 | Asiatic acid (Dammarolic acid,Asiantic acid,2alpha,23-Dihydroxyursolic acid) | Triterpenoids | P10827 | 0.110612204 | 3 |
| 2333 | Asiatic acid (Dammarolic acid,Asiantic acid,2alpha,23-Dihydroxyursolic acid) | Triterpenoids | P10828 | 0.110612204 | 3 |
| 2334 | Asiatic acid (Dammarolic acid,Asiantic acid,2alpha,23-Dihydroxyursolic acid) | Triterpenoids | P11387 | 0.113455556 | 13 |
| 2335 | Asiatic acid (Dammarolic acid,Asiantic acid,2alpha,23-Dihydroxyursolic acid) | Triterpenoids | P11388 | 0.106757964 | 8 |
| 2336 | Asiatic acid (Dammarolic acid,Asiantic acid,2alpha,23-Dihydroxyursolic acid) | Triterpenoids | P11413 | 0.113579529 | 12 |
| 2337 | Asiatic acid (Dammarolic acid,Asiantic acid,2alpha,23-Dihydroxyursolic acid) | Triterpenoids | P11511 | 0.139056613 | 29 |
| 2338 | Asiatic acid (Dammarolic acid,Asiantic acid,2alpha,23-Dihydroxyursolic acid) | Triterpenoids | P15090 | 0.111920896 | 10 |
| 2339 | Asiatic acid (Dammarolic acid,Asiantic acid,2alpha,23-Dihydroxyursolic acid) | Triterpenoids | P17706 | 0.347170021 | 15 |
| 2340 | Asiatic acid (Dammarolic acid,Asiantic acid,2alpha,23-Dihydroxyursolic acid) | Triterpenoids | P18031 | 0.474772974 | 23 |
| 2341 | Asiatic acid (Dammarolic acid,Asiantic acid,2alpha,23-Dihydroxyursolic acid) | Triterpenoids | P20292 | 0.111722841 | 8 |
| 2342 | Asiatic acid (Dammarolic acid,Asiantic acid,2alpha,23-Dihydroxyursolic acid) | Triterpenoids | P23219 | 0.109524644 | 16 |
| 2343 | Asiatic acid (Dammarolic acid,Asiantic acid,2alpha,23-Dihydroxyursolic acid) | Triterpenoids | P24666 | 0.343119127 | 13 |
| 2344 | Asiatic acid (Dammarolic acid,Asiantic acid,2alpha,23-Dihydroxyursolic acid) | Triterpenoids | P24723 | 0.11185669 | 11 |
| 2345 | Asiatic acid (Dammarolic acid,Asiantic acid,2alpha,23-Dihydroxyursolic acid) | Triterpenoids | P28845 | 0.253303956 | 34 |
| 2346 | Asiatic acid (Dammarolic acid,Asiantic acid,2alpha,23-Dihydroxyursolic acid) | Triterpenoids | P29350 | 0.204438393 | 44 |
| 2347 | Asiatic acid (Dammarolic acid,Asiantic acid,2alpha,23-Dihydroxyursolic acid) | Triterpenoids | P30304 | 0.109145376 | 25 |
| 2348 | Asiatic acid (Dammarolic acid,Asiantic acid,2alpha,23-Dihydroxyursolic acid) | Triterpenoids | P30305 | 0.185734372 | 40 |
| 2349 | Asiatic acid (Dammarolic acid,Asiantic acid,2alpha,23-Dihydroxyursolic acid) | Triterpenoids | P35228 | 0.112440313 | 15 |
| 2350 | Asiatic acid (Dammarolic acid,Asiantic acid,2alpha,23-Dihydroxyursolic acid) | Triterpenoids | P35354 | 0.113487837 | 23 |
| 2351 | Asiatic acid (Dammarolic acid,Asiantic acid,2alpha,23-Dihydroxyursolic acid) | Triterpenoids | P35408 | 0.113274443 | 9 |
| 2352 | Asiatic acid (Dammarolic acid,Asiantic acid,2alpha,23-Dihydroxyursolic acid) | Triterpenoids | P37231 | 0.111742909 | 15 |
| 2353 | Asiatic acid (Dammarolic acid,Asiantic acid,2alpha,23-Dihydroxyursolic acid) | Triterpenoids | P43088 | 0.115549923 | 5 |
| 2354 | Asiatic acid (Dammarolic acid,Asiantic acid,2alpha,23-Dihydroxyursolic acid) | Triterpenoids | P43116 | 0.114821812 | 13 |
| 2355 | Asiatic acid (Dammarolic acid,Asiantic acid,2alpha,23-Dihydroxyursolic acid) | Triterpenoids | P43119 | 0.112265692 | 10 |
| 2356 | Asiatic acid (Dammarolic acid,Asiantic acid,2alpha,23-Dihydroxyursolic acid) | Triterpenoids | P48147 | 0.107233003 | 22 |
| 2357 | Asiatic acid (Dammarolic acid,Asiantic acid,2alpha,23-Dihydroxyursolic acid) | Triterpenoids | P49354 P49356 | 0.109904857 | 24 |
| 2358 | Asiatic acid (Dammarolic acid,Asiantic acid,2alpha,23-Dihydroxyursolic acid) | Triterpenoids | P51449 | 0.406390508 | 15 |
| 2359 | Asiatic acid (Dammarolic acid,Asiantic acid,2alpha,23-Dihydroxyursolic acid) | Triterpenoids | P60033 | 0.195084726 | 11 |
| 2360 | Asiatic acid (Dammarolic acid,Asiantic acid,2alpha,23-Dihydroxyursolic acid) | Triterpenoids | P80365 | 0.109471969 | 12 |
| 2361 | Asiatic acid (Dammarolic acid,Asiantic acid,2alpha,23-Dihydroxyursolic acid) | Triterpenoids | Q01469 | 0.111920896 | 10 |
| 2362 | Asiatic acid (Dammarolic acid,Asiantic acid,2alpha,23-Dihydroxyursolic acid) | Triterpenoids | Q03181 | 0.111670964 | 14 |
| 2363 | Asiatic acid (Dammarolic acid,Asiantic acid,2alpha,23-Dihydroxyursolic acid) | Triterpenoids | Q06124 | 0.211032909 | 40 |
| 2364 | Asiatic acid (Dammarolic acid,Asiantic acid,2alpha,23-Dihydroxyursolic acid) | Triterpenoids | Q07869 | 0.112467055 | 11 |
| 2365 | Asiatic acid (Dammarolic acid,Asiantic acid,2alpha,23-Dihydroxyursolic acid) | Triterpenoids | Q08499 | 0.31881944 | 14 |
| 2366 | Asiatic acid (Dammarolic acid,Asiantic acid,2alpha,23-Dihydroxyursolic acid) | Triterpenoids | Q12908 | 0.115418978 | 4 |
| 2367 | Asiatic acid (Dammarolic acid,Asiantic acid,2alpha,23-Dihydroxyursolic acid) | Triterpenoids | Q13133 | 0.216325973 | 16 |
| 2368 | Asiatic acid (Dammarolic acid,Asiantic acid,2alpha,23-Dihydroxyursolic acid) | Triterpenoids | Q13258 | 0.113015591 | 4 |
| 2369 | Asiatic acid (Dammarolic acid,Asiantic acid,2alpha,23-Dihydroxyursolic acid) | Triterpenoids | Q14973 | 0.116380332 | 5 |
| 2370 | Asiatic acid (Dammarolic acid,Asiantic acid,2alpha,23-Dihydroxyursolic acid) | Triterpenoids | Q15722 | 0.111722841 | 8 |
| 2371 | Asiatic acid (Dammarolic acid,Asiantic acid,2alpha,23-Dihydroxyursolic acid) | Triterpenoids | Q16850 | 0.152847021 | 14 |
| 2372 | Asiatic acid (Dammarolic acid,Asiantic acid,2alpha,23-Dihydroxyursolic acid) | Triterpenoids | Q8TDU6 | 0.111722841 | 8 |
| 2373 | Asiatic acid (Dammarolic acid,Asiantic acid,2alpha,23-Dihydroxyursolic acid) | Triterpenoids | Q92731 | 0.117586956 | 23 |
| 2374 | Asiatic acid (Dammarolic acid,Asiantic acid,2alpha,23-Dihydroxyursolic acid) | Triterpenoids | Q96RI1 | 0.11050814 | 7 |
| 2375 | Asiatic acid (Dammarolic acid,Asiantic acid,2alpha,23-Dihydroxyursolic acid) | Triterpenoids | Q9NR96 | 0.108367851 | 6 |
| 2376 | Asiatic acid (Dammarolic acid,Asiantic acid,2alpha,23-Dihydroxyursolic acid) | Triterpenoids | Q9UHC9 | 0.28130208 | 13 |
| 2377 | Asiatic acid (Dammarolic acid,Asiantic acid,2alpha,23-Dihydroxyursolic acid) | Triterpenoids | Q9Y5Y4 | 0.113671543 | 7 |
| 2378 | barbinervic acid | Triterpenoids | O00519 | 0.110352709 | 16 |
| 2379 | barbinervic acid | Triterpenoids | O00748 | 0.259386615 | 47 |
| 2380 | barbinervic acid | Triterpenoids | O00767 | 0.113596703 | 10 |
| 2381 | barbinervic acid | Triterpenoids | O14684 | 0.116389783 | 19 |
| 2382 | barbinervic acid | Triterpenoids | O14746 | 0.14728518 | 27 |
| 2383 | barbinervic acid | Triterpenoids | O60218 | 0.499225177 | 13 |
| 2384 | barbinervic acid | Triterpenoids | O76074 | 0.103099774 | 12 |
| 2385 | barbinervic acid | Triterpenoids | P01375 | 0.108038789 | 7 |
| 2386 | barbinervic acid | Triterpenoids | P03372 | 0.132037828 | 16 |
| 2387 | barbinervic acid | Triterpenoids | P03956 | 0.109842942 | 8 |
| 2388 | barbinervic acid | Triterpenoids | P04035 | 0.166864971 | 20 |
| 2389 | barbinervic acid | Triterpenoids | P04054 | 0.395466092 | 11 |
| 2390 | barbinervic acid | Triterpenoids | P04150 | 0.109500234 | 17 |
| 2391 | barbinervic acid | Triterpenoids | P04278 | 0.16719946 | 19 |
| 2392 | barbinervic acid | Triterpenoids | P05093 | 0.148009819 | 20 |
| 2393 | barbinervic acid | Triterpenoids | P05413 | 0.111920896 | 10 |
| 2394 | barbinervic acid | Triterpenoids | P06401 | 0.108868706 | 15 |
| 2395 | barbinervic acid | Triterpenoids | P06746 | 0.307158581 | 16 |
| 2396 | barbinervic acid | Triterpenoids | P07148 | 0.115257869 | 10 |
| 2397 | barbinervic acid | Triterpenoids | P08185 | 0.113039089 | 17 |
| 2398 | barbinervic acid | Triterpenoids | P08253 | 0.106581489 | 9 |
| 2399 | barbinervic acid | Triterpenoids | P08254 | 0.109296209 | 8 |
| 2400 | barbinervic acid | Triterpenoids | P09917 | 0.106782208 | 14 |
| 2401 | barbinervic acid | Triterpenoids | P10275 | 0.132131799 | 34 |
| 2402 | barbinervic acid | Triterpenoids | P10586 | 0.312345915 | 15 |
| 2403 | barbinervic acid | Triterpenoids | P10827 | 0.110612204 | 3 |
| 2404 | barbinervic acid | Triterpenoids | P10828 | 0.110612204 | 3 |
| 2405 | barbinervic acid | Triterpenoids | P11413 | 0.113579529 | 12 |
| 2406 | barbinervic acid | Triterpenoids | P11511 | 0.139056613 | 29 |
| 2407 | barbinervic acid | Triterpenoids | P14902 | 0.110454469 | 24 |
| 2408 | barbinervic acid | Triterpenoids | P15090 | 0.111920896 | 10 |
| 2409 | barbinervic acid | Triterpenoids | P17706 | 0.347170021 | 15 |
| 2410 | barbinervic acid | Triterpenoids | P18031 | 0.474772974 | 23 |
| 2411 | barbinervic acid | Triterpenoids | P20292 | 0.111722841 | 8 |
| 2412 | barbinervic acid | Triterpenoids | P20701 | 0.127026321 | 4 |
| 2413 | barbinervic acid | Triterpenoids | P23219 | 0.109524644 | 16 |
| 2414 | barbinervic acid | Triterpenoids | P24666 | 0.343119127 | 13 |
| 2415 | barbinervic acid | Triterpenoids | P24723 | 0.11185669 | 11 |
| 2416 | barbinervic acid | Triterpenoids | P28845 | 0.253303956 | 34 |
| 2417 | barbinervic acid | Triterpenoids | P29350 | 0.204438393 | 44 |
| 2418 | barbinervic acid | Triterpenoids | P30304 | 0.109145376 | 25 |
| 2419 | barbinervic acid | Triterpenoids | P30305 | 0.185734372 | 40 |
| 2420 | barbinervic acid | Triterpenoids | P30556 | 0.113117188 | 7 |
| 2421 | barbinervic acid | Triterpenoids | P31645 | 0.1201852 | 17 |
| 2422 | barbinervic acid | Triterpenoids | P34995 | 0.114009578 | 9 |
| 2423 | barbinervic acid | Triterpenoids | P35228 | 0.112440313 | 15 |
| 2424 | barbinervic acid | Triterpenoids | P35354 | 0.113487837 | 23 |
| 2425 | barbinervic acid | Triterpenoids | P35398 | 0.130342003 | 12 |
| 2426 | barbinervic acid | Triterpenoids | P35408 | 0.113274443 | 9 |
| 2427 | barbinervic acid | Triterpenoids | P37231 | 0.111742909 | 15 |
| 2428 | barbinervic acid | Triterpenoids | P37268 | 0.110958418 | 7 |
| 2429 | barbinervic acid | Triterpenoids | P43088 | 0.115549923 | 5 |
| 2430 | barbinervic acid | Triterpenoids | P43115 | 0.11287548 | 5 |
| 2431 | barbinervic acid | Triterpenoids | P43116 | 0.114821812 | 13 |
| 2432 | barbinervic acid | Triterpenoids | P43119 | 0.112265692 | 10 |
| 2433 | barbinervic acid | Triterpenoids | P48147 | 0.107233003 | 22 |
| 2434 | barbinervic acid | Triterpenoids | P49354 P49356 | 0.109904857 | 24 |
| 2435 | barbinervic acid | Triterpenoids | P49840 | 0.108060801 | 5 |
| 2436 | barbinervic acid | Triterpenoids | P51449 | 0.406390508 | 15 |
| 2437 | barbinervic acid | Triterpenoids | P60033 | 0.195084726 | 11 |
| 2438 | barbinervic acid | Triterpenoids | P80365 | 0.109471969 | 12 |
| 2439 | barbinervic acid | Triterpenoids | Q00987 | 0.107095673 | 11 |
| 2440 | barbinervic acid | Triterpenoids | Q01433 | 0.110908758 | 3 |
| 2441 | barbinervic acid | Triterpenoids | Q01469 | 0.111920896 | 10 |
| 2442 | barbinervic acid | Triterpenoids | Q03181 | 0.111670964 | 14 |
| 2443 | barbinervic acid | Triterpenoids | Q06124 | 0.211032909 | 40 |
| 2444 | barbinervic acid | Triterpenoids | Q07869 | 0.112467055 | 11 |
| 2445 | barbinervic acid | Triterpenoids | Q08499 | 0.31881944 | 14 |
| 2446 | barbinervic acid | Triterpenoids | Q12908 | 0.115418978 | 4 |
| 2447 | barbinervic acid | Triterpenoids | Q13133 | 0.216325973 | 16 |
| 2448 | barbinervic acid | Triterpenoids | Q13258 | 0.113015591 | 4 |
| 2449 | barbinervic acid | Triterpenoids | Q13547 | 0.110937969 | 6 |
| 2450 | barbinervic acid | Triterpenoids | Q14973 | 0.116380332 | 5 |
| 2451 | barbinervic acid | Triterpenoids | Q15722 | 0.111722841 | 8 |
| 2452 | barbinervic acid | Triterpenoids | Q16850 | 0.152847021 | 14 |
| 2453 | barbinervic acid | Triterpenoids | Q7Z2W7 | 0.106776816 | 5 |
| 2454 | barbinervic acid | Triterpenoids | Q8TDU6 | 0.111722841 | 8 |
| 2455 | barbinervic acid | Triterpenoids | Q92731 | 0.117586956 | 23 |
| 2456 | barbinervic acid | Triterpenoids | Q96RI1 | 0.11050814 | 7 |
| 2457 | barbinervic acid | Triterpenoids | Q99720 | 0.110060881 | 8 |
| 2458 | barbinervic acid | Triterpenoids | Q9UHC9 | 0.28130208 | 13 |
| 2459 | barbinervic acid | Triterpenoids | Q9Y5Y4 | 0.113671543 | 7 |
| 2460 | Corosolic acid (colosolic acid,2alpha-hydroxyursolic acid) | Triterpenoids | O00519 | 0.110352709 | 16 |
| 2461 | Corosolic acid (colosolic acid,2alpha-hydroxyursolic acid) | Triterpenoids | O00748 | 0.259386615 | 47 |
| 2462 | Corosolic acid (colosolic acid,2alpha-hydroxyursolic acid) | Triterpenoids | O00767 | 0.113596703 | 10 |
| 2463 | Corosolic acid (colosolic acid,2alpha-hydroxyursolic acid) | Triterpenoids | O14684 | 0.116389783 | 19 |
| 2464 | Corosolic acid (colosolic acid,2alpha-hydroxyursolic acid) | Triterpenoids | O14746 | 0.14728518 | 27 |
| 2465 | Corosolic acid (colosolic acid,2alpha-hydroxyursolic acid) | Triterpenoids | O14842 | 0.110430092 | 4 |
| 2466 | Corosolic acid (colosolic acid,2alpha-hydroxyursolic acid) | Triterpenoids | O60218 | 0.499225177 | 13 |
| 2467 | Corosolic acid (colosolic acid,2alpha-hydroxyursolic acid) | Triterpenoids | P03372 | 0.132037828 | 16 |
| 2468 | Corosolic acid (colosolic acid,2alpha-hydroxyursolic acid) | Triterpenoids | P04035 | 0.166864971 | 20 |
| 2469 | Corosolic acid (colosolic acid,2alpha-hydroxyursolic acid) | Triterpenoids | P04054 | 0.395466092 | 11 |
| 2470 | Corosolic acid (colosolic acid,2alpha-hydroxyursolic acid) | Triterpenoids | P04150 | 0.109500234 | 17 |
| 2471 | Corosolic acid (colosolic acid,2alpha-hydroxyursolic acid) | Triterpenoids | P04278 | 0.16719946 | 19 |
| 2472 | Corosolic acid (colosolic acid,2alpha-hydroxyursolic acid) | Triterpenoids | P05093 | 0.148009819 | 20 |
| 2473 | Corosolic acid (colosolic acid,2alpha-hydroxyursolic acid) | Triterpenoids | P05413 | 0.111920896 | 10 |
| 2474 | Corosolic acid (colosolic acid,2alpha-hydroxyursolic acid) | Triterpenoids | P06276 | 0.130116287 | 14 |
| 2475 | Corosolic acid (colosolic acid,2alpha-hydroxyursolic acid) | Triterpenoids | P06401 | 0.108868706 | 15 |
| 2476 | Corosolic acid (colosolic acid,2alpha-hydroxyursolic acid) | Triterpenoids | P06746 | 0.307158581 | 16 |
| 2477 | Corosolic acid (colosolic acid,2alpha-hydroxyursolic acid) | Triterpenoids | P07148 | 0.115257869 | 10 |
| 2478 | Corosolic acid (colosolic acid,2alpha-hydroxyursolic acid) | Triterpenoids | P08172 | 0.117318329 | 18 |
| 2479 | Corosolic acid (colosolic acid,2alpha-hydroxyursolic acid) | Triterpenoids | P08185 | 0.113039089 | 17 |
| 2480 | Corosolic acid (colosolic acid,2alpha-hydroxyursolic acid) | Triterpenoids | P08235 | 0.107998327 | 12 |
| 2481 | Corosolic acid (colosolic acid,2alpha-hydroxyursolic acid) | Triterpenoids | P09917 | 0.106782208 | 14 |
| 2482 | Corosolic acid (colosolic acid,2alpha-hydroxyursolic acid) | Triterpenoids | P10275 | 0.132131799 | 34 |
| 2483 | Corosolic acid (colosolic acid,2alpha-hydroxyursolic acid) | Triterpenoids | P10586 | 0.312345915 | 15 |
| 2484 | Corosolic acid (colosolic acid,2alpha-hydroxyursolic acid) | Triterpenoids | P11387 | 0.113455556 | 13 |
| 2485 | Corosolic acid (colosolic acid,2alpha-hydroxyursolic acid) | Triterpenoids | P11388 | 0.106757964 | 8 |
| 2486 | Corosolic acid (colosolic acid,2alpha-hydroxyursolic acid) | Triterpenoids | P11413 | 0.113579529 | 12 |
| 2487 | Corosolic acid (colosolic acid,2alpha-hydroxyursolic acid) | Triterpenoids | P11511 | 0.139056613 | 29 |
| 2488 | Corosolic acid (colosolic acid,2alpha-hydroxyursolic acid) | Triterpenoids | P15090 | 0.111920896 | 10 |
| 2489 | Corosolic acid (colosolic acid,2alpha-hydroxyursolic acid) | Triterpenoids | P17706 | 0.347170021 | 15 |
| 2490 | Corosolic acid (colosolic acid,2alpha-hydroxyursolic acid) | Triterpenoids | P18031 | 0.474772974 | 23 |
| 2491 | Corosolic acid (colosolic acid,2alpha-hydroxyursolic acid) | Triterpenoids | P20292 | 0.111722841 | 8 |
| 2492 | Corosolic acid (colosolic acid,2alpha-hydroxyursolic acid) | Triterpenoids | P23219 | 0.109524644 | 16 |
| 2493 | Corosolic acid (colosolic acid,2alpha-hydroxyursolic acid) | Triterpenoids | P24666 | 0.343119127 | 13 |
| 2494 | Corosolic acid (colosolic acid,2alpha-hydroxyursolic acid) | Triterpenoids | P24723 | 0.11185669 | 11 |
| 2495 | Corosolic acid (colosolic acid,2alpha-hydroxyursolic acid) | Triterpenoids | P28845 | 0.253303956 | 34 |
| 2496 | Corosolic acid (colosolic acid,2alpha-hydroxyursolic acid) | Triterpenoids | P29350 | 0.204438393 | 44 |
| 2497 | Corosolic acid (colosolic acid,2alpha-hydroxyursolic acid) | Triterpenoids | P30304 | 0.109145376 | 25 |
| 2498 | Corosolic acid (colosolic acid,2alpha-hydroxyursolic acid) | Triterpenoids | P30305 | 0.185734372 | 40 |
| 2499 | Corosolic acid (colosolic acid,2alpha-hydroxyursolic acid) | Triterpenoids | P30556 | 0.113117188 | 7 |
| 2500 | Corosolic acid (colosolic acid,2alpha-hydroxyursolic acid) | Triterpenoids | P31213 | 0.110430092 | 4 |
| 2501 | Corosolic acid (colosolic acid,2alpha-hydroxyursolic acid) | Triterpenoids | P31645 | 0.1201852 | 17 |
| 2502 | Corosolic acid (colosolic acid,2alpha-hydroxyursolic acid) | Triterpenoids | P34995 | 0.114009578 | 9 |
| 2503 | Corosolic acid (colosolic acid,2alpha-hydroxyursolic acid) | Triterpenoids | P35228 | 0.112440313 | 15 |
| 2504 | Corosolic acid (colosolic acid,2alpha-hydroxyursolic acid) | Triterpenoids | P35354 | 0.113487837 | 23 |
| 2505 | Corosolic acid (colosolic acid,2alpha-hydroxyursolic acid) | Triterpenoids | P35398 | 0.130342003 | 12 |
| 2506 | Corosolic acid (colosolic acid,2alpha-hydroxyursolic acid) | Triterpenoids | P35408 | 0.113274443 | 9 |
| 2507 | Corosolic acid (colosolic acid,2alpha-hydroxyursolic acid) | Triterpenoids | P37231 | 0.111742909 | 15 |
| 2508 | Corosolic acid (colosolic acid,2alpha-hydroxyursolic acid) | Triterpenoids | P39086 | 0.110430092 | 4 |
| 2509 | Corosolic acid (colosolic acid,2alpha-hydroxyursolic acid) | Triterpenoids | P43116 | 0.114821812 | 13 |
| 2510 | Corosolic acid (colosolic acid,2alpha-hydroxyursolic acid) | Triterpenoids | P43119 | 0.112265692 | 10 |
| 2511 | Corosolic acid (colosolic acid,2alpha-hydroxyursolic acid) | Triterpenoids | P48147 | 0.107233003 | 22 |
| 2512 | Corosolic acid (colosolic acid,2alpha-hydroxyursolic acid) | Triterpenoids | P49354 P49356 | 0.109904857 | 24 |
| 2513 | Corosolic acid (colosolic acid,2alpha-hydroxyursolic acid) | Triterpenoids | P51449 | 0.406390508 | 15 |
| 2514 | Corosolic acid (colosolic acid,2alpha-hydroxyursolic acid) | Triterpenoids | P56817 | 0.108325485 | 12 |
| 2515 | Corosolic acid (colosolic acid,2alpha-hydroxyursolic acid) | Triterpenoids | P60033 | 0.195084726 | 11 |
| 2516 | Corosolic acid (colosolic acid,2alpha-hydroxyursolic acid) | Triterpenoids | P80365 | 0.109471969 | 12 |
| 2517 | Corosolic acid (colosolic acid,2alpha-hydroxyursolic acid) | Triterpenoids | Q00987 | 0.107095673 | 11 |
| 2518 | Corosolic acid (colosolic acid,2alpha-hydroxyursolic acid) | Triterpenoids | Q01469 | 0.111920896 | 10 |
| 2519 | Corosolic acid (colosolic acid,2alpha-hydroxyursolic acid) | Triterpenoids | Q01959 | 0.107731401 | 12 |
| 2520 | Corosolic acid (colosolic acid,2alpha-hydroxyursolic acid) | Triterpenoids | Q03181 | 0.111670964 | 14 |
| 2521 | Corosolic acid (colosolic acid,2alpha-hydroxyursolic acid) | Triterpenoids | Q06124 | 0.211032909 | 40 |
| 2522 | Corosolic acid (colosolic acid,2alpha-hydroxyursolic acid) | Triterpenoids | Q07869 | 0.112467055 | 11 |
| 2523 | Corosolic acid (colosolic acid,2alpha-hydroxyursolic acid) | Triterpenoids | Q08499 | 0.31881944 | 14 |
| 2524 | Corosolic acid (colosolic acid,2alpha-hydroxyursolic acid) | Triterpenoids | Q12908 | 0.115418978 | 4 |
| 2525 | Corosolic acid (colosolic acid,2alpha-hydroxyursolic acid) | Triterpenoids | Q13002 | 0.110430092 | 4 |
| 2526 | Corosolic acid (colosolic acid,2alpha-hydroxyursolic acid) | Triterpenoids | Q13133 | 0.216325973 | 16 |
| 2527 | Corosolic acid (colosolic acid,2alpha-hydroxyursolic acid) | Triterpenoids | Q14973 | 0.116380332 | 5 |
| 2528 | Corosolic acid (colosolic acid,2alpha-hydroxyursolic acid) | Triterpenoids | Q15722 | 0.111722841 | 8 |
| 2529 | Corosolic acid (colosolic acid,2alpha-hydroxyursolic acid) | Triterpenoids | Q16850 | 0.152847021 | 14 |
| 2530 | Corosolic acid (colosolic acid,2alpha-hydroxyursolic acid) | Triterpenoids | Q8TDU6 | 0.111722841 | 8 |
| 2531 | Corosolic acid (colosolic acid,2alpha-hydroxyursolic acid) | Triterpenoids | Q92731 | 0.117586956 | 23 |
| 2532 | Corosolic acid (colosolic acid,2alpha-hydroxyursolic acid) | Triterpenoids | Q96RI1 | 0.11050814 | 7 |
| 2533 | Corosolic acid (colosolic acid,2alpha-hydroxyursolic acid) | Triterpenoids | Q9NR96 | 0.108367851 | 6 |
| 2534 | Corosolic acid (colosolic acid,2alpha-hydroxyursolic acid) | Triterpenoids | Q9Y5Y4 | 0.113671543 | 7 |
| 2535 | maslinic acid | Triterpenoids | O00519 | 0.110352709 | 16 |
| 2536 | maslinic acid | Triterpenoids | O00748 | 0.259386615 | 47 |
| 2537 | maslinic acid | Triterpenoids | O00767 | 0.113596703 | 10 |
| 2538 | maslinic acid | Triterpenoids | O14684 | 0.116389783 | 19 |
| 2539 | maslinic acid | Triterpenoids | O14746 | 0.14728518 | 27 |
| 2540 | maslinic acid | Triterpenoids | O14842 | 0.110430092 | 4 |
| 2541 | maslinic acid | Triterpenoids | O60218 | 0.499225177 | 13 |
| 2542 | maslinic acid | Triterpenoids | P03372 | 0.132037828 | 16 |
| 2543 | maslinic acid | Triterpenoids | P04035 | 0.166864971 | 20 |
| 2544 | maslinic acid | Triterpenoids | P04054 | 0.395466092 | 11 |
| 2545 | maslinic acid | Triterpenoids | P04150 | 0.109500234 | 17 |
| 2546 | maslinic acid | Triterpenoids | P04278 | 0.16719946 | 19 |
| 2547 | maslinic acid | Triterpenoids | P05093 | 0.148009819 | 20 |
| 2548 | maslinic acid | Triterpenoids | P05413 | 0.111920896 | 10 |
| 2549 | maslinic acid | Triterpenoids | P06276 | 0.130116287 | 14 |
| 2550 | maslinic acid | Triterpenoids | P06401 | 0.108868706 | 15 |
| 2551 | maslinic acid | Triterpenoids | P06746 | 0.307158581 | 16 |
| 2552 | maslinic acid | Triterpenoids | P07148 | 0.115257869 | 10 |
| 2553 | maslinic acid | Triterpenoids | P08172 | 0.117318329 | 18 |
| 2554 | maslinic acid | Triterpenoids | P08185 | 0.113039089 | 17 |
| 2555 | maslinic acid | Triterpenoids | P08235 | 0.107998327 | 12 |
| 2556 | maslinic acid | Triterpenoids | P09917 | 0.106782208 | 14 |
| 2557 | maslinic acid | Triterpenoids | P10275 | 0.132131799 | 34 |
| 2558 | maslinic acid | Triterpenoids | P10586 | 0.312345915 | 15 |
| 2559 | maslinic acid | Triterpenoids | P11387 | 0.113455556 | 13 |
| 2560 | maslinic acid | Triterpenoids | P11388 | 0.106757964 | 8 |
| 2561 | maslinic acid | Triterpenoids | P11413 | 0.113579529 | 12 |
| 2562 | maslinic acid | Triterpenoids | P11511 | 0.139056613 | 29 |
| 2563 | maslinic acid | Triterpenoids | P15090 | 0.111920896 | 10 |
| 2564 | maslinic acid | Triterpenoids | P17706 | 0.347170021 | 15 |
| 2565 | maslinic acid | Triterpenoids | P18031 | 0.474772974 | 23 |
| 2566 | maslinic acid | Triterpenoids | P20292 | 0.111722841 | 8 |
| 2567 | maslinic acid | Triterpenoids | P23219 | 0.109524644 | 16 |
| 2568 | maslinic acid | Triterpenoids | P24666 | 0.343119127 | 13 |
| 2569 | maslinic acid | Triterpenoids | P24723 | 0.11185669 | 11 |
| 2570 | maslinic acid | Triterpenoids | P28845 | 0.253303956 | 34 |
| 2571 | maslinic acid | Triterpenoids | P29350 | 0.204438393 | 44 |
| 2572 | maslinic acid | Triterpenoids | P30304 | 0.109145376 | 25 |
| 2573 | maslinic acid | Triterpenoids | P30305 | 0.185734372 | 40 |
| 2574 | maslinic acid | Triterpenoids | P30556 | 0.113117188 | 7 |
| 2575 | maslinic acid | Triterpenoids | P31213 | 0.110430092 | 4 |
| 2576 | maslinic acid | Triterpenoids | P31645 | 0.1201852 | 17 |
| 2577 | maslinic acid | Triterpenoids | P34995 | 0.114009578 | 9 |
| 2578 | maslinic acid | Triterpenoids | P35228 | 0.112440313 | 15 |
| 2579 | maslinic acid | Triterpenoids | P35354 | 0.113487837 | 23 |
| 2580 | maslinic acid | Triterpenoids | P35398 | 0.130342003 | 12 |
| 2581 | maslinic acid | Triterpenoids | P35408 | 0.113274443 | 9 |
| 2582 | maslinic acid | Triterpenoids | P37231 | 0.111742909 | 15 |
| 2583 | maslinic acid | Triterpenoids | P39086 | 0.110430092 | 4 |
| 2584 | maslinic acid | Triterpenoids | P43116 | 0.114821812 | 13 |
| 2585 | maslinic acid | Triterpenoids | P43119 | 0.112265692 | 10 |
| 2586 | maslinic acid | Triterpenoids | P48147 | 0.107233003 | 22 |
| 2587 | maslinic acid | Triterpenoids | P49354 P49356 | 0.109904857 | 24 |
| 2588 | maslinic acid | Triterpenoids | P51449 | 0.406390508 | 15 |
| 2589 | maslinic acid | Triterpenoids | P56817 | 0.108325485 | 12 |
| 2590 | maslinic acid | Triterpenoids | P60033 | 0.195084726 | 11 |
| 2591 | maslinic acid | Triterpenoids | P80365 | 0.109471969 | 12 |
| 2592 | maslinic acid | Triterpenoids | Q00987 | 0.107095673 | 11 |
| 2593 | maslinic acid | Triterpenoids | Q01469 | 0.111920896 | 10 |
| 2594 | maslinic acid | Triterpenoids | Q01959 | 0.107731401 | 12 |
| 2595 | maslinic acid | Triterpenoids | Q03181 | 0.111670964 | 14 |
| 2596 | maslinic acid | Triterpenoids | Q06124 | 0.211032909 | 40 |
| 2597 | maslinic acid | Triterpenoids | Q07869 | 0.112467055 | 11 |
| 2598 | maslinic acid | Triterpenoids | Q08499 | 0.31881944 | 14 |
| 2599 | maslinic acid | Triterpenoids | Q12908 | 0.115418978 | 4 |
| 2600 | maslinic acid | Triterpenoids | Q13002 | 0.110430092 | 4 |
| 2601 | maslinic acid | Triterpenoids | Q13133 | 0.216325973 | 16 |
| 2602 | maslinic acid | Triterpenoids | Q14973 | 0.116380332 | 5 |
| 2603 | maslinic acid | Triterpenoids | Q15722 | 0.111722841 | 8 |
| 2604 | maslinic acid | Triterpenoids | Q16850 | 0.152847021 | 14 |
| 2605 | maslinic acid | Triterpenoids | Q8TDU6 | 0.111722841 | 8 |
| 2606 | maslinic acid | Triterpenoids | Q92731 | 0.117586956 | 23 |
| 2607 | maslinic acid | Triterpenoids | Q96RI1 | 0.11050814 | 7 |
| 2608 | maslinic acid | Triterpenoids | Q9NR96 | 0.108367851 | 6 |
| 2609 | maslinic acid | Triterpenoids | Q9Y5Y4 | 0.113671543 | 7 |
| 2610 | oleanolic acid | Triterpenoids | O00519 | 0.110352709 | 16 |
| 2611 | oleanolic acid | Triterpenoids | O00748 | 0.259386615 | 47 |
| 2612 | oleanolic acid | Triterpenoids | O00767 | 0.113596703 | 10 |
| 2613 | oleanolic acid | Triterpenoids | O14684 | 0.116389783 | 19 |
| 2614 | oleanolic acid | Triterpenoids | O14746 | 0.14728518 | 27 |
| 2615 | oleanolic acid | Triterpenoids | O14842 | 0.110430092 | 4 |
| 2616 | oleanolic acid | Triterpenoids | O60218 | 0.499225177 | 13 |
| 2617 | oleanolic acid | Triterpenoids | P03372 | 0.132037828 | 16 |
| 2618 | oleanolic acid | Triterpenoids | P04035 | 0.166864971 | 20 |
| 2619 | oleanolic acid | Triterpenoids | P04054 | 0.395466092 | 11 |
| 2620 | oleanolic acid | Triterpenoids | P04150 | 0.109500234 | 17 |
| 2621 | oleanolic acid | Triterpenoids | P04278 | 0.16719946 | 19 |
| 2622 | oleanolic acid | Triterpenoids | P05093 | 0.148009819 | 20 |
| 2623 | oleanolic acid | Triterpenoids | P05413 | 0.111920896 | 10 |
| 2624 | oleanolic acid | Triterpenoids | P06276 | 0.130116287 | 14 |
| 2625 | oleanolic acid | Triterpenoids | P06401 | 0.108868706 | 15 |
| 2626 | oleanolic acid | Triterpenoids | P06746 | 0.307158581 | 16 |
| 2627 | oleanolic acid | Triterpenoids | P07148 | 0.115257869 | 10 |
| 2628 | oleanolic acid | Triterpenoids | P08172 | 0.117318329 | 18 |
| 2629 | oleanolic acid | Triterpenoids | P08185 | 0.113039089 | 17 |
| 2630 | oleanolic acid | Triterpenoids | P08235 | 0.107998327 | 12 |
| 2631 | oleanolic acid | Triterpenoids | P09917 | 0.106782208 | 14 |
| 2632 | oleanolic acid | Triterpenoids | P10275 | 0.132131799 | 34 |
| 2633 | oleanolic acid | Triterpenoids | P10586 | 0.312345915 | 15 |
| 2634 | oleanolic acid | Triterpenoids | P11387 | 0.113455556 | 13 |
| 2635 | oleanolic acid | Triterpenoids | P11413 | 0.113579529 | 12 |
| 2636 | oleanolic acid | Triterpenoids | P11511 | 0.139056613 | 29 |
| 2637 | oleanolic acid | Triterpenoids | P15090 | 0.111920896 | 10 |
| 2638 | oleanolic acid | Triterpenoids | P17706 | 0.347170021 | 15 |
| 2639 | oleanolic acid | Triterpenoids | P18031 | 0.474772974 | 23 |
| 2640 | oleanolic acid | Triterpenoids | P20292 | 0.111722841 | 8 |
| 2641 | oleanolic acid | Triterpenoids | P23141 | 0.275271523 | 31 |
| 2642 | oleanolic acid | Triterpenoids | P23219 | 0.109524644 | 16 |
| 2643 | oleanolic acid | Triterpenoids | P23975 | 0.115012308 | 10 |
| 2644 | oleanolic acid | Triterpenoids | P24666 | 0.343119127 | 13 |
| 2645 | oleanolic acid | Triterpenoids | P24723 | 0.11185669 | 11 |
| 2646 | oleanolic acid | Triterpenoids | P27361 | 0.105473292 | 5 |
| 2647 | oleanolic acid | Triterpenoids | P28845 | 0.253303956 | 34 |
| 2648 | oleanolic acid | Triterpenoids | P29350 | 0.204438393 | 44 |
| 2649 | oleanolic acid | Triterpenoids | P30304 | 0.109145376 | 25 |
| 2650 | oleanolic acid | Triterpenoids | P30305 | 0.185734372 | 40 |
| 2651 | oleanolic acid | Triterpenoids | P31213 | 0.110430092 | 4 |
| 2652 | oleanolic acid | Triterpenoids | P33261 | 0.124903734 | 16 |
| 2653 | oleanolic acid | Triterpenoids | P35228 | 0.112440313 | 15 |
| 2654 | oleanolic acid | Triterpenoids | P35354 | 0.113487837 | 23 |
| 2655 | oleanolic acid | Triterpenoids | P35398 | 0.130342003 | 12 |
| 2656 | oleanolic acid | Triterpenoids | P35408 | 0.113274443 | 9 |
| 2657 | oleanolic acid | Triterpenoids | P37231 | 0.111742909 | 15 |
| 2658 | oleanolic acid | Triterpenoids | P39086 | 0.110430092 | 4 |
| 2659 | oleanolic acid | Triterpenoids | P43116 | 0.114821812 | 13 |
| 2660 | oleanolic acid | Triterpenoids | P43119 | 0.112265692 | 10 |
| 2661 | oleanolic acid | Triterpenoids | P48147 | 0.107233003 | 22 |
| 2662 | oleanolic acid | Triterpenoids | P49354 P49356 | 0.109904857 | 24 |
| 2663 | oleanolic acid | Triterpenoids | P51449 | 0.406390508 | 15 |
| 2664 | oleanolic acid | Triterpenoids | P56817 | 0.108325485 | 12 |
| 2665 | oleanolic acid | Triterpenoids | P60033 | 0.195084726 | 11 |
| 2666 | oleanolic acid | Triterpenoids | P80365 | 0.109471969 | 12 |
| 2667 | oleanolic acid | Triterpenoids | Q00987 | 0.107095673 | 11 |
| 2668 | oleanolic acid | Triterpenoids | Q01469 | 0.111920896 | 10 |
| 2669 | oleanolic acid | Triterpenoids | Q01959 | 0.107731401 | 12 |
| 2670 | oleanolic acid | Triterpenoids | Q03181 | 0.111670964 | 14 |
| 2671 | oleanolic acid | Triterpenoids | Q06124 | 0.211032909 | 40 |
| 2672 | oleanolic acid | Triterpenoids | Q07869 | 0.112467055 | 11 |
| 2673 | oleanolic acid | Triterpenoids | Q08499 | 0.31881944 | 14 |
| 2674 | oleanolic acid | Triterpenoids | Q13002 | 0.110430092 | 4 |
| 2675 | oleanolic acid | Triterpenoids | Q13133 | 0.216325973 | 16 |
| 2676 | oleanolic acid | Triterpenoids | Q14994 | 0.12030258 | 8 |
| 2677 | oleanolic acid | Triterpenoids | Q15722 | 0.111722841 | 8 |
| 2678 | oleanolic acid | Triterpenoids | Q16850 | 0.152847021 | 14 |
| 2679 | oleanolic acid | Triterpenoids | Q8TDU6 | 0.111722841 | 8 |
| 2680 | oleanolic acid | Triterpenoids | Q92731 | 0.117586956 | 23 |
| 2681 | oleanolic acid | Triterpenoids | Q96RI1 | 0.11050814 | 7 |
| 2682 | oleanolic acid | Triterpenoids | Q99720 | 0.110060881 | 8 |
| 2683 | oleanolic acid | Triterpenoids | Q9NR96 | 0.108367851 | 6 |
| 2684 | oleanolic acid | Triterpenoids | Q9UHC9 | 0.28130208 | 13 |
| 2685 | pomolic acid | Triterpenoids | O00519 | 0.110352709 | 16 |
| 2686 | pomolic acid | Triterpenoids | O00748 | 0.259386615 | 47 |
| 2687 | pomolic acid | Triterpenoids | O00767 | 0.113596703 | 10 |
| 2688 | pomolic acid | Triterpenoids | O14684 | 0.116389783 | 19 |
| 2689 | pomolic acid | Triterpenoids | O14746 | 0.14728518 | 27 |
| 2690 | pomolic acid | Triterpenoids | O60218 | 0.499225177 | 13 |
| 2691 | pomolic acid | Triterpenoids | P01375 | 0.108038789 | 7 |
| 2692 | pomolic acid | Triterpenoids | P03372 | 0.132037828 | 16 |
| 2693 | pomolic acid | Triterpenoids | P04054 | 0.395466092 | 11 |
| 2694 | pomolic acid | Triterpenoids | P04150 | 0.109500234 | 17 |
| 2695 | pomolic acid | Triterpenoids | P04278 | 0.16719946 | 19 |
| 2696 | pomolic acid | Triterpenoids | P05093 | 0.148009819 | 20 |
| 2697 | pomolic acid | Triterpenoids | P05413 | 0.111920896 | 10 |
| 2698 | pomolic acid | Triterpenoids | P06746 | 0.307158581 | 16 |
| 2699 | pomolic acid | Triterpenoids | P07148 | 0.115257869 | 10 |
| 2700 | pomolic acid | Triterpenoids | P08185 | 0.113039089 | 17 |
| 2701 | pomolic acid | Triterpenoids | P10275 | 0.132131799 | 34 |
| 2702 | pomolic acid | Triterpenoids | P10586 | 0.312345915 | 15 |
| 2703 | pomolic acid | Triterpenoids | P11413 | 0.113579529 | 12 |
| 2704 | pomolic acid | Triterpenoids | P11511 | 0.139056613 | 29 |
| 2705 | pomolic acid | Triterpenoids | P14902 | 0.110454469 | 24 |
| 2706 | pomolic acid | Triterpenoids | P15090 | 0.111920896 | 10 |
| 2707 | pomolic acid | Triterpenoids | P17706 | 0.347170021 | 15 |
| 2708 | pomolic acid | Triterpenoids | P18031 | 0.474772974 | 23 |
| 2709 | pomolic acid | Triterpenoids | P20292 | 0.111722841 | 8 |
| 2710 | pomolic acid | Triterpenoids | P23219 | 0.109524644 | 16 |
| 2711 | pomolic acid | Triterpenoids | P24666 | 0.343119127 | 13 |
| 2712 | pomolic acid | Triterpenoids | P24723 | 0.11185669 | 11 |
| 2713 | pomolic acid | Triterpenoids | P28845 | 0.253303956 | 34 |
| 2714 | pomolic acid | Triterpenoids | P29350 | 0.204438393 | 44 |
| 2715 | pomolic acid | Triterpenoids | P30304 | 0.109145376 | 25 |
| 2716 | pomolic acid | Triterpenoids | P30305 | 0.185734372 | 40 |
| 2717 | pomolic acid | Triterpenoids | P30556 | 0.113117188 | 7 |
| 2718 | pomolic acid | Triterpenoids | P31645 | 0.1201852 | 17 |
| 2719 | pomolic acid | Triterpenoids | P34995 | 0.114009578 | 9 |
| 2720 | pomolic acid | Triterpenoids | P35228 | 0.112440313 | 15 |
| 2721 | pomolic acid | Triterpenoids | P35354 | 0.113487837 | 23 |
| 2722 | pomolic acid | Triterpenoids | P35398 | 0.130342003 | 12 |
| 2723 | pomolic acid | Triterpenoids | P35408 | 0.113274443 | 9 |
| 2724 | pomolic acid | Triterpenoids | P37231 | 0.111742909 | 15 |
| 2725 | pomolic acid | Triterpenoids | P37268 | 0.110958418 | 7 |
| 2726 | pomolic acid | Triterpenoids | P43088 | 0.115549923 | 5 |
| 2727 | pomolic acid | Triterpenoids | P43115 | 0.11287548 | 5 |
| 2728 | pomolic acid | Triterpenoids | P43116 | 0.114821812 | 13 |
| 2729 | pomolic acid | Triterpenoids | P43119 | 0.112265692 | 10 |
| 2730 | pomolic acid | Triterpenoids | P48147 | 0.107233003 | 22 |
| 2731 | pomolic acid | Triterpenoids | P49354 P49356 | 0.109904857 | 24 |
| 2732 | pomolic acid | Triterpenoids | P51449 | 0.406390508 | 15 |
| 2733 | pomolic acid | Triterpenoids | P60033 | 0.195084726 | 11 |
| 2734 | pomolic acid | Triterpenoids | P80365 | 0.109471969 | 12 |
| 2735 | pomolic acid | Triterpenoids | Q01469 | 0.111920896 | 10 |
| 2736 | pomolic acid | Triterpenoids | Q03181 | 0.111670964 | 14 |
| 2737 | pomolic acid | Triterpenoids | Q06124 | 0.211032909 | 40 |
| 2738 | pomolic acid | Triterpenoids | Q07869 | 0.112467055 | 11 |
| 2739 | pomolic acid | Triterpenoids | Q08499 | 0.31881944 | 14 |
| 2740 | pomolic acid | Triterpenoids | Q12772 | 0.216727786 | 7 |
| 2741 | pomolic acid | Triterpenoids | Q13133 | 0.216325973 | 16 |
| 2742 | pomolic acid | Triterpenoids | Q13258 | 0.113015591 | 4 |
| 2743 | pomolic acid | Triterpenoids | Q14973 | 0.116380332 | 5 |
| 2744 | pomolic acid | Triterpenoids | Q15722 | 0.111722841 | 8 |
| 2745 | pomolic acid | Triterpenoids | Q16850 | 0.152847021 | 14 |
| 2746 | pomolic acid | Triterpenoids | Q8TDU6 | 0.111722841 | 8 |
| 2747 | pomolic acid | Triterpenoids | Q92731 | 0.117586956 | 23 |
| 2748 | pomolic acid | Triterpenoids | Q99720 | 0.110060881 | 8 |
| 2749 | pomolic acid | Triterpenoids | Q9UHC9 | 0.28130208 | 13 |
| 2750 | pomolic acid | Triterpenoids | Q9Y5Y4 | 0.113671543 | 7 |
| 2751 | taraxerol | Triterpenoids | O00519 | 0.110352709 | 16 |
| 2752 | taraxerol | Triterpenoids | O00748 | 0.259386615 | 47 |
| 2753 | taraxerol | Triterpenoids | O00767 | 0.113596703 | 10 |
| 2754 | taraxerol | Triterpenoids | O14684 | 0.116389783 | 19 |
| 2755 | taraxerol | Triterpenoids | O14746 | 0.14728518 | 27 |
| 2756 | taraxerol | Triterpenoids | O60218 | 0.499225177 | 13 |
| 2757 | taraxerol | Triterpenoids | P03372 | 0.132037828 | 16 |
| 2758 | taraxerol | Triterpenoids | P04035 | 0.166864971 | 20 |
| 2759 | taraxerol | Triterpenoids | P04054 | 0.395466092 | 11 |
| 2760 | taraxerol | Triterpenoids | P04278 | 0.16719946 | 19 |
| 2761 | taraxerol | Triterpenoids | P05093 | 0.148009819 | 20 |
| 2762 | taraxerol | Triterpenoids | P05413 | 0.111920896 | 10 |
| 2763 | taraxerol | Triterpenoids | P06276 | 0.130116287 | 14 |
| 2764 | taraxerol | Triterpenoids | P06746 | 0.307158581 | 16 |
| 2765 | taraxerol | Triterpenoids | P07148 | 0.115257869 | 10 |
| 2766 | taraxerol | Triterpenoids | P08172 | 0.117318329 | 18 |
| 2767 | taraxerol | Triterpenoids | P08185 | 0.113039089 | 17 |
| 2768 | taraxerol | Triterpenoids | P09917 | 0.106782208 | 14 |
| 2769 | taraxerol | Triterpenoids | P0DMS8 | 0.10464569 | 23 |
| 2770 | taraxerol | Triterpenoids | P10275 | 0.132131799 | 34 |
| 2771 | taraxerol | Triterpenoids | P10586 | 0.312345915 | 15 |
| 2772 | taraxerol | Triterpenoids | P11473 | 0.109776491 | 5 |
| 2773 | taraxerol | Triterpenoids | P11511 | 0.139056613 | 29 |
| 2774 | taraxerol | Triterpenoids | P15090 | 0.111920896 | 10 |
| 2775 | taraxerol | Triterpenoids | P16662 | 0.109345148 | 4 |
| 2776 | taraxerol | Triterpenoids | P17706 | 0.347170021 | 15 |
| 2777 | taraxerol | Triterpenoids | P18031 | 0.474772974 | 23 |
| 2778 | taraxerol | Triterpenoids | P21554 | 0.104626059 | 10 |
| 2779 | taraxerol | Triterpenoids | P22303 | 0.287353637 | 26 |
| 2780 | taraxerol | Triterpenoids | P23975 | 0.115012308 | 10 |
| 2781 | taraxerol | Triterpenoids | P24666 | 0.343119127 | 13 |
| 2782 | taraxerol | Triterpenoids | P27361 | 0.105473292 | 5 |
| 2783 | taraxerol | Triterpenoids | P28845 | 0.253303956 | 34 |
| 2784 | taraxerol | Triterpenoids | P29350 | 0.204438393 | 44 |
| 2785 | taraxerol | Triterpenoids | P30304 | 0.109145376 | 25 |
| 2786 | taraxerol | Triterpenoids | P30305 | 0.185734372 | 40 |
| 2787 | taraxerol | Triterpenoids | P31645 | 0.1201852 | 17 |
| 2788 | taraxerol | Triterpenoids | P33261 | 0.124903734 | 16 |
| 2789 | taraxerol | Triterpenoids | P48147 | 0.107233003 | 22 |
| 2790 | taraxerol | Triterpenoids | P49354 P49356 | 0.109904857 | 24 |
| 2791 | taraxerol | Triterpenoids | P51449 | 0.406390508 | 15 |
| 2792 | taraxerol | Triterpenoids | P60033 | 0.195084726 | 11 |
| 2793 | taraxerol | Triterpenoids | P80365 | 0.109471969 | 12 |
| 2794 | taraxerol | Triterpenoids | Q01469 | 0.111920896 | 10 |
| 2795 | taraxerol | Triterpenoids | Q03181 | 0.111670964 | 14 |
| 2796 | taraxerol | Triterpenoids | Q06124 | 0.211032909 | 40 |
| 2797 | taraxerol | Triterpenoids | Q08499 | 0.31881944 | 14 |
| 2798 | taraxerol | Triterpenoids | Q12772 | 0.216727786 | 7 |
| 2799 | taraxerol | Triterpenoids | Q13133 | 0.216325973 | 16 |
| 2800 | taraxerol | Triterpenoids | Q14534 | 0.110691545 | 6 |
| 2801 | taraxerol | Triterpenoids | Q14994 | 0.12030258 | 8 |
| 2802 | taraxerol | Triterpenoids | Q16850 | 0.152847021 | 14 |
| 2803 | taraxerol | Triterpenoids | Q92731 | 0.117586956 | 23 |
| 2804 | taraxerol | Triterpenoids | Q9UHC9 | 0.28130208 | 13 |
| 2805 | urs-12-ene-2α,3β,7β,16α-tetraol | Triterpenoids | O00311 | 0.105117399 | 6 |
| 2806 | urs-12-ene-2α,3β,7β,16α-tetraol | Triterpenoids | O00748 | 0.259386615 | 47 |
| 2807 | urs-12-ene-2α,3β,7β,16α-tetraol | Triterpenoids | O60218 | 0.499225177 | 13 |
| 2808 | urs-12-ene-2α,3β,7β,16α-tetraol | Triterpenoids | P03372 | 0.132037828 | 16 |
| 2809 | urs-12-ene-2α,3β,7β,16α-tetraol | Triterpenoids | P04035 | 0.166864971 | 20 |
| 2810 | urs-12-ene-2α,3β,7β,16α-tetraol | Triterpenoids | P04278 | 0.16719946 | 19 |
| 2811 | urs-12-ene-2α,3β,7β,16α-tetraol | Triterpenoids | P05093 | 0.148009819 | 20 |
| 2812 | urs-12-ene-2α,3β,7β,16α-tetraol | Triterpenoids | P06276 | 0.130116287 | 14 |
| 2813 | urs-12-ene-2α,3β,7β,16α-tetraol | Triterpenoids | P06746 | 0.307158581 | 16 |
| 2814 | urs-12-ene-2α,3β,7β,16α-tetraol | Triterpenoids | P08172 | 0.117318329 | 18 |
| 2815 | urs-12-ene-2α,3β,7β,16α-tetraol | Triterpenoids | P08185 | 0.113039089 | 17 |
| 2816 | urs-12-ene-2α,3β,7β,16α-tetraol | Triterpenoids | P09874 | 0.106818993 | 18 |
| 2817 | urs-12-ene-2α,3β,7β,16α-tetraol | Triterpenoids | P10275 | 0.132131799 | 34 |
| 2818 | urs-12-ene-2α,3β,7β,16α-tetraol | Triterpenoids | P10586 | 0.312345915 | 15 |
| 2819 | urs-12-ene-2α,3β,7β,16α-tetraol | Triterpenoids | P11511 | 0.139056613 | 29 |
| 2820 | urs-12-ene-2α,3β,7β,16α-tetraol | Triterpenoids | P18031 | 0.474772974 | 23 |
| 2821 | urs-12-ene-2α,3β,7β,16α-tetraol | Triterpenoids | P22303 | 0.287353637 | 26 |
| 2822 | urs-12-ene-2α,3β,7β,16α-tetraol | Triterpenoids | P23975 | 0.115012308 | 10 |
| 2823 | urs-12-ene-2α,3β,7β,16α-tetraol | Triterpenoids | P24666 | 0.343119127 | 13 |
| 2824 | urs-12-ene-2α,3β,7β,16α-tetraol | Triterpenoids | P28472 P18507 P14867 | 0.103855577 | 16 |
| 2825 | urs-12-ene-2α,3β,7β,16α-tetraol | Triterpenoids | P28472 P18507 P31644 | 0.103791839 | 19 |
| 2826 | urs-12-ene-2α,3β,7β,16α-tetraol | Triterpenoids | P28472 P34903 P18507 | 0.103855577 | 16 |
| 2827 | urs-12-ene-2α,3β,7β,16α-tetraol | Triterpenoids | P28845 | 0.253303956 | 34 |
| 2828 | urs-12-ene-2α,3β,7β,16α-tetraol | Triterpenoids | P29350 | 0.204438393 | 44 |
| 2829 | urs-12-ene-2α,3β,7β,16α-tetraol | Triterpenoids | P30305 | 0.185734372 | 40 |
| 2830 | urs-12-ene-2α,3β,7β,16α-tetraol | Triterpenoids | P31645 | 0.1201852 | 17 |
| 2831 | urs-12-ene-2α,3β,7β,16α-tetraol | Triterpenoids | P32246 | 0.109647095 | 6 |
| 2832 | urs-12-ene-2α,3β,7β,16α-tetraol | Triterpenoids | P33261 | 0.124903734 | 16 |
| 2833 | urs-12-ene-2α,3β,7β,16α-tetraol | Triterpenoids | P35398 | 0.130342003 | 12 |
| 2834 | urs-12-ene-2α,3β,7β,16α-tetraol | Triterpenoids | P35968 | 0.105052994 | 26 |
| 2835 | urs-12-ene-2α,3β,7β,16α-tetraol | Triterpenoids | P37023 | 0.111434828 | 4 |
| 2836 | urs-12-ene-2α,3β,7β,16α-tetraol | Triterpenoids | P41145 | 0.107282863 | 6 |
| 2837 | urs-12-ene-2α,3β,7β,16α-tetraol | Triterpenoids | P47869 P28472 P18507 | 0.104254314 | 15 |
| 2838 | urs-12-ene-2α,3β,7β,16α-tetraol | Triterpenoids | P49354 P49356 | 0.109904857 | 24 |
| 2839 | urs-12-ene-2α,3β,7β,16α-tetraol | Triterpenoids | P49840 | 0.108060801 | 5 |
| 2840 | urs-12-ene-2α,3β,7β,16α-tetraol | Triterpenoids | P49841 | 0.105823985 | 11 |
| 2841 | urs-12-ene-2α,3β,7β,16α-tetraol | Triterpenoids | P51449 | 0.406390508 | 15 |
| 2842 | urs-12-ene-2α,3β,7β,16α-tetraol | Triterpenoids | P52333 | 0.104243408 | 12 |
| 2843 | urs-12-ene-2α,3β,7β,16α-tetraol | Triterpenoids | P52732 | 0.104747724 | 9 |
| 2844 | urs-12-ene-2α,3β,7β,16α-tetraol | Triterpenoids | P60033 | 0.195084726 | 11 |
| 2845 | urs-12-ene-2α,3β,7β,16α-tetraol | Triterpenoids | Q12772 | 0.216727786 | 7 |
| 2846 | urs-12-ene-2α,3β,7β,16α-tetraol | Triterpenoids | Q13133 | 0.216325973 | 16 |
| 2847 | urs-12-ene-2α,3β,7β,16α-tetraol | Triterpenoids | Q14534 | 0.110691545 | 6 |
| 2848 | urs-12-ene-2α,3β,7β,16α-tetraol | Triterpenoids | Q14994 | 0.12030258 | 8 |
| 2849 | urs-12-ene-2α,3β,7β,16α-tetraol | Triterpenoids | Q16539 | 0.105438848 | 20 |
| 2850 | urs-12-ene-2α,3β,7β,16α-tetraol | Triterpenoids | Q16850 | 0.152847021 | 14 |
| 2851 | urs-12-ene-2α,3β,7β,16α-tetraol | Triterpenoids | Q92731 | 0.117586956 | 23 |
| 2852 | urs-12-ene-2α,3β,7β,16α-tetraol | Triterpenoids | Q9UHC9 | 0.28130208 | 13 |
| 2853 | urs-12-ene-2α,3β,7β,16α-tetraol | Triterpenoids | Q9Y233 | 0.108085897 | 14 |
| 2854 | ursolic acid | Triterpenoids | O00519 | 0.110352709 | 16 |
| 2855 | ursolic acid | Triterpenoids | O00748 | 0.259386615 | 47 |
| 2856 | ursolic acid | Triterpenoids | O00767 | 0.113596703 | 10 |
| 2857 | ursolic acid | Triterpenoids | O14684 | 0.116389783 | 19 |
| 2858 | ursolic acid | Triterpenoids | O14746 | 0.14728518 | 27 |
| 2859 | ursolic acid | Triterpenoids | O14842 | 0.110430092 | 4 |
| 2860 | ursolic acid | Triterpenoids | O60218 | 0.499225177 | 13 |
| 2861 | ursolic acid | Triterpenoids | P03372 | 0.132037828 | 16 |
| 2862 | ursolic acid | Triterpenoids | P04035 | 0.166864971 | 20 |
| 2863 | ursolic acid | Triterpenoids | P04054 | 0.395466092 | 11 |
| 2864 | ursolic acid | Triterpenoids | P04150 | 0.109500234 | 17 |
| 2865 | ursolic acid | Triterpenoids | P04278 | 0.16719946 | 19 |
| 2866 | ursolic acid | Triterpenoids | P05093 | 0.148009819 | 20 |
| 2867 | ursolic acid | Triterpenoids | P05413 | 0.111920896 | 10 |
| 2868 | ursolic acid | Triterpenoids | P06276 | 0.130116287 | 14 |
| 2869 | ursolic acid | Triterpenoids | P06401 | 0.108868706 | 15 |
| 2870 | ursolic acid | Triterpenoids | P06746 | 0.307158581 | 16 |
| 2871 | ursolic acid | Triterpenoids | P07148 | 0.115257869 | 10 |
| 2872 | ursolic acid | Triterpenoids | P08172 | 0.117318329 | 18 |
| 2873 | ursolic acid | Triterpenoids | P08185 | 0.113039089 | 17 |
| 2874 | ursolic acid | Triterpenoids | P08235 | 0.107998327 | 12 |
| 2875 | ursolic acid | Triterpenoids | P09917 | 0.106782208 | 14 |
| 2876 | ursolic acid | Triterpenoids | P10275 | 0.132131799 | 34 |
| 2877 | ursolic acid | Triterpenoids | P10586 | 0.312345915 | 15 |
| 2878 | ursolic acid | Triterpenoids | P11387 | 0.113455556 | 13 |
| 2879 | ursolic acid | Triterpenoids | P11413 | 0.113579529 | 12 |
| 2880 | ursolic acid | Triterpenoids | P11511 | 0.139056613 | 29 |
| 2881 | ursolic acid | Triterpenoids | P15090 | 0.111920896 | 10 |
| 2882 | ursolic acid | Triterpenoids | P17706 | 0.347170021 | 15 |
| 2883 | ursolic acid | Triterpenoids | P18031 | 0.474772974 | 23 |
| 2884 | ursolic acid | Triterpenoids | P20292 | 0.111722841 | 8 |
| 2885 | ursolic acid | Triterpenoids | P23141 | 0.275271523 | 31 |
| 2886 | ursolic acid | Triterpenoids | P23219 | 0.109524644 | 16 |
| 2887 | ursolic acid | Triterpenoids | P23975 | 0.115012308 | 10 |
| 2888 | ursolic acid | Triterpenoids | P24666 | 0.343119127 | 13 |
| 2889 | ursolic acid | Triterpenoids | P24723 | 0.11185669 | 11 |
| 2890 | ursolic acid | Triterpenoids | P27361 | 0.105473292 | 5 |
| 2891 | ursolic acid | Triterpenoids | P28845 | 0.253303956 | 34 |
| 2892 | ursolic acid | Triterpenoids | P29350 | 0.204438393 | 44 |
| 2893 | ursolic acid | Triterpenoids | P30304 | 0.109145376 | 25 |
| 2894 | ursolic acid | Triterpenoids | P30305 | 0.185734372 | 40 |
| 2895 | ursolic acid | Triterpenoids | P31213 | 0.110430092 | 4 |
| 2896 | ursolic acid | Triterpenoids | P31645 | 0.1201852 | 17 |
| 2897 | ursolic acid | Triterpenoids | P33261 | 0.124903734 | 16 |
| 2898 | ursolic acid | Triterpenoids | P35228 | 0.112440313 | 15 |
| 2899 | ursolic acid | Triterpenoids | P35354 | 0.113487837 | 23 |
| 2900 | ursolic acid | Triterpenoids | P35398 | 0.130342003 | 12 |
| 2901 | ursolic acid | Triterpenoids | P35408 | 0.113274443 | 9 |
| 2902 | ursolic acid | Triterpenoids | P37231 | 0.111742909 | 15 |
| 2903 | ursolic acid | Triterpenoids | P39086 | 0.110430092 | 4 |
| 2904 | ursolic acid | Triterpenoids | P43116 | 0.114821812 | 13 |
| 2905 | ursolic acid | Triterpenoids | P43119 | 0.112265692 | 10 |
| 2906 | ursolic acid | Triterpenoids | P48147 | 0.107233003 | 22 |
| 2907 | ursolic acid | Triterpenoids | P49354 P49356 | 0.109904857 | 24 |
| 2908 | ursolic acid | Triterpenoids | P51449 | 0.406390508 | 15 |
| 2909 | ursolic acid | Triterpenoids | P56817 | 0.108325485 | 12 |
| 2910 | ursolic acid | Triterpenoids | P60033 | 0.195084726 | 11 |
| 2911 | ursolic acid | Triterpenoids | P80365 | 0.109471969 | 12 |
| 2912 | ursolic acid | Triterpenoids | Q00987 | 0.107095673 | 11 |
| 2913 | ursolic acid | Triterpenoids | Q01469 | 0.111920896 | 10 |
| 2914 | ursolic acid | Triterpenoids | Q01959 | 0.107731401 | 12 |
| 2915 | ursolic acid | Triterpenoids | Q03181 | 0.111670964 | 14 |
| 2916 | ursolic acid | Triterpenoids | Q06124 | 0.211032909 | 40 |
| 2917 | ursolic acid | Triterpenoids | Q07869 | 0.112467055 | 11 |
| 2918 | ursolic acid | Triterpenoids | Q08499 | 0.31881944 | 14 |
| 2919 | ursolic acid | Triterpenoids | Q13002 | 0.110430092 | 4 |
| 2920 | ursolic acid | Triterpenoids | Q13133 | 0.216325973 | 16 |
| 2921 | ursolic acid | Triterpenoids | Q14994 | 0.12030258 | 8 |
| 2922 | ursolic acid | Triterpenoids | Q15722 | 0.111722841 | 8 |
| 2923 | ursolic acid | Triterpenoids | Q16850 | 0.152847021 | 14 |
| 2924 | ursolic acid | Triterpenoids | Q8TDU6 | 0.111722841 | 8 |
| 2925 | ursolic acid | Triterpenoids | Q92731 | 0.117586956 | 23 |
| 2926 | ursolic acid | Triterpenoids | Q96RI1 | 0.11050814 | 7 |
| 2927 | ursolic acid | Triterpenoids | Q99720 | 0.110060881 | 8 |
| 2928 | ursolic acid | Triterpenoids | Q9NR96 | 0.108367851 | 6 |
| 2929 | ursolic acid | Triterpenoids | Q9UHC9 | 0.28130208 | 13 |
| 2930 | uvaol | Triterpenoids | O00519 | 0.110352709 | 16 |
| 2931 | uvaol | Triterpenoids | O00748 | 0.259386615 | 47 |
| 2932 | uvaol | Triterpenoids | O00767 | 0.113596703 | 10 |
| 2933 | uvaol | Triterpenoids | O14684 | 0.116389783 | 19 |
| 2934 | uvaol | Triterpenoids | O14746 | 0.14728518 | 27 |
| 2935 | uvaol | Triterpenoids | O60218 | 0.499225177 | 13 |
| 2936 | uvaol | Triterpenoids | P03372 | 0.132037828 | 16 |
| 2937 | uvaol | Triterpenoids | P04035 | 0.166864971 | 20 |
| 2938 | uvaol | Triterpenoids | P04054 | 0.395466092 | 11 |
| 2939 | uvaol | Triterpenoids | P04278 | 0.16719946 | 19 |
| 2940 | uvaol | Triterpenoids | P05093 | 0.148009819 | 20 |
| 2941 | uvaol | Triterpenoids | P05413 | 0.111920896 | 10 |
| 2942 | uvaol | Triterpenoids | P06276 | 0.130116287 | 14 |
| 2943 | uvaol | Triterpenoids | P06746 | 0.307158581 | 16 |
| 2944 | uvaol | Triterpenoids | P07148 | 0.115257869 | 10 |
| 2945 | uvaol | Triterpenoids | P08172 | 0.117318329 | 18 |
| 2946 | uvaol | Triterpenoids | P08185 | 0.113039089 | 17 |
| 2947 | uvaol | Triterpenoids | P08235 | 0.107998327 | 12 |
| 2948 | uvaol | Triterpenoids | P10275 | 0.132131799 | 34 |
| 2949 | uvaol | Triterpenoids | P10586 | 0.312345915 | 15 |
| 2950 | uvaol | Triterpenoids | P11511 | 0.139056613 | 29 |
| 2951 | uvaol | Triterpenoids | P15090 | 0.111920896 | 10 |
| 2952 | uvaol | Triterpenoids | P17706 | 0.347170021 | 15 |
| 2953 | uvaol | Triterpenoids | P18031 | 0.474772974 | 23 |
| 2954 | uvaol | Triterpenoids | P21554 | 0.104626059 | 10 |
| 2955 | uvaol | Triterpenoids | P22303 | 0.287353637 | 26 |
| 2956 | uvaol | Triterpenoids | P23975 | 0.115012308 | 10 |
| 2957 | uvaol | Triterpenoids | P24666 | 0.343119127 | 13 |
| 2958 | uvaol | Triterpenoids | P28845 | 0.253303956 | 34 |
| 2959 | uvaol | Triterpenoids | P29350 | 0.204438393 | 44 |
| 2960 | uvaol | Triterpenoids | P30305 | 0.185734372 | 40 |
| 2961 | uvaol | Triterpenoids | P31645 | 0.1201852 | 17 |
| 2962 | uvaol | Triterpenoids | P33261 | 0.124903734 | 16 |
| 2963 | uvaol | Triterpenoids | P35354 | 0.113487837 | 23 |
| 2964 | uvaol | Triterpenoids | P48147 | 0.107233003 | 22 |
| 2965 | uvaol | Triterpenoids | P49354 P49356 | 0.109904857 | 24 |
| 2966 | uvaol | Triterpenoids | P51449 | 0.406390508 | 15 |
| 2967 | uvaol | Triterpenoids | P60033 | 0.195084726 | 11 |
| 2968 | uvaol | Triterpenoids | P80365 | 0.109471969 | 12 |
| 2969 | uvaol | Triterpenoids | Q01469 | 0.111920896 | 10 |
| 2970 | uvaol | Triterpenoids | Q03181 | 0.111670964 | 14 |
| 2971 | uvaol | Triterpenoids | Q08499 | 0.31881944 | 14 |
| 2972 | uvaol | Triterpenoids | Q12772 | 0.216727786 | 7 |
| 2973 | uvaol | Triterpenoids | Q13133 | 0.216325973 | 16 |
| 2974 | uvaol | Triterpenoids | Q14534 | 0.110691545 | 6 |
| 2975 | uvaol | Triterpenoids | Q14994 | 0.12030258 | 8 |
| 2976 | uvaol | Triterpenoids | Q16850 | 0.152847021 | 14 |
| 2977 | uvaol | Triterpenoids | Q92731 | 0.117586956 | 23 |
| 2978 | uvaol | Triterpenoids | Q9UHC9 | 0.28130208 | 13 |
| 2979 | euscaphic acid | Triterpenoids | O00519 | 0.110352709 | 16 |
| 2980 | euscaphic acid | Triterpenoids | O00748 | 0.259386615 | 47 |
| 2981 | euscaphic acid | Triterpenoids | O00767 | 0.113596703 | 10 |
| 2982 | euscaphic acid | Triterpenoids | O14684 | 0.116389783 | 19 |
| 2983 | euscaphic acid | Triterpenoids | O14746 | 0.14728518 | 27 |
| 2984 | euscaphic acid | Triterpenoids | O60218 | 0.499225177 | 13 |
| 2985 | euscaphic acid | Triterpenoids | O76074 | 0.103099774 | 12 |
| 2986 | euscaphic acid | Triterpenoids | P01375 | 0.108038789 | 7 |
| 2987 | euscaphic acid | Triterpenoids | P03372 | 0.132037828 | 16 |
| 2988 | euscaphic acid | Triterpenoids | P03951 | 0.106451353 | 3 |
| 2989 | euscaphic acid | Triterpenoids | P03956 | 0.109842942 | 8 |
| 2990 | euscaphic acid | Triterpenoids | P04035 | 0.166864971 | 20 |
| 2991 | euscaphic acid | Triterpenoids | P04054 | 0.395466092 | 11 |
| 2992 | euscaphic acid | Triterpenoids | P04150 | 0.109500234 | 17 |
| 2993 | euscaphic acid | Triterpenoids | P04278 | 0.16719946 | 19 |
| 2994 | euscaphic acid | Triterpenoids | P05093 | 0.148009819 | 20 |
| 2995 | euscaphic acid | Triterpenoids | P05413 | 0.111920896 | 10 |
| 2996 | euscaphic acid | Triterpenoids | P06401 | 0.108868706 | 15 |
| 2997 | euscaphic acid | Triterpenoids | P06746 | 0.307158581 | 16 |
| 2998 | euscaphic acid | Triterpenoids | P07148 | 0.115257869 | 10 |
| 2999 | euscaphic acid | Triterpenoids | P08185 | 0.113039089 | 17 |
| 3000 | euscaphic acid | Triterpenoids | P08253 | 0.106581489 | 9 |
| 3001 | euscaphic acid | Triterpenoids | P08254 | 0.109296209 | 8 |
| 3002 | euscaphic acid | Triterpenoids | P10275 | 0.132131799 | 34 |
| 3003 | euscaphic acid | Triterpenoids | P10586 | 0.312345915 | 15 |
| 3004 | euscaphic acid | Triterpenoids | P10827 | 0.110612204 | 3 |
| 3005 | euscaphic acid | Triterpenoids | P10828 | 0.110612204 | 3 |
| 3006 | euscaphic acid | Triterpenoids | P11413 | 0.113579529 | 12 |
| 3007 | euscaphic acid | Triterpenoids | P11511 | 0.139056613 | 29 |
| 3008 | euscaphic acid | Triterpenoids | P14902 | 0.110454469 | 24 |
| 3009 | euscaphic acid | Triterpenoids | P15090 | 0.111920896 | 10 |
| 3010 | euscaphic acid | Triterpenoids | P17706 | 0.347170021 | 15 |
| 3011 | euscaphic acid | Triterpenoids | P18031 | 0.474772974 | 23 |
| 3012 | euscaphic acid | Triterpenoids | P20292 | 0.111722841 | 8 |
| 3013 | euscaphic acid | Triterpenoids | P23219 | 0.109524644 | 16 |
| 3014 | euscaphic acid | Triterpenoids | P24666 | 0.343119127 | 13 |
| 3015 | euscaphic acid | Triterpenoids | P24723 | 0.11185669 | 11 |
| 3016 | euscaphic acid | Triterpenoids | P27487 | 0.104386227 | 3 |
| 3017 | euscaphic acid | Triterpenoids | P28845 | 0.253303956 | 34 |
| 3018 | euscaphic acid | Triterpenoids | P29350 | 0.204438393 | 44 |
| 3019 | euscaphic acid | Triterpenoids | P30304 | 0.109145376 | 25 |
| 3020 | euscaphic acid | Triterpenoids | P30305 | 0.185734372 | 40 |
| 3021 | euscaphic acid | Triterpenoids | P30556 | 0.113117188 | 7 |
| 3022 | euscaphic acid | Triterpenoids | P31645 | 0.1201852 | 17 |
| 3023 | euscaphic acid | Triterpenoids | P34995 | 0.114009578 | 9 |
| 3024 | euscaphic acid | Triterpenoids | P35228 | 0.112440313 | 15 |
| 3025 | euscaphic acid | Triterpenoids | P35354 | 0.113487837 | 23 |
| 3026 | euscaphic acid | Triterpenoids | P35398 | 0.130342003 | 12 |
| 3027 | euscaphic acid | Triterpenoids | P35408 | 0.113274443 | 9 |
| 3028 | euscaphic acid | Triterpenoids | P37231 | 0.111742909 | 15 |
| 3029 | euscaphic acid | Triterpenoids | P37268 | 0.110958418 | 7 |
| 3030 | euscaphic acid | Triterpenoids | P43088 | 0.115549923 | 5 |
| 3031 | euscaphic acid | Triterpenoids | P43115 | 0.11287548 | 5 |
| 3032 | euscaphic acid | Triterpenoids | P43116 | 0.114821812 | 13 |
| 3033 | euscaphic acid | Triterpenoids | P43119 | 0.112265692 | 10 |
| 3034 | euscaphic acid | Triterpenoids | P48147 | 0.107233003 | 22 |
| 3035 | euscaphic acid | Triterpenoids | P49354 P49356 | 0.109904857 | 24 |
| 3036 | euscaphic acid | Triterpenoids | P49840 | 0.108060801 | 5 |
| 3037 | euscaphic acid | Triterpenoids | P51449 | 0.406390508 | 15 |
| 3038 | euscaphic acid | Triterpenoids | P56817 | 0.108325485 | 12 |
| 3039 | euscaphic acid | Triterpenoids | P60033 | 0.195084726 | 11 |
| 3040 | euscaphic acid | Triterpenoids | P80365 | 0.109471969 | 12 |
| 3041 | euscaphic acid | Triterpenoids | Q01433 | 0.110908758 | 3 |
| 3042 | euscaphic acid | Triterpenoids | Q01469 | 0.111920896 | 10 |
| 3043 | euscaphic acid | Triterpenoids | Q03181 | 0.111670964 | 14 |
| 3044 | euscaphic acid | Triterpenoids | Q06124 | 0.211032909 | 40 |
| 3045 | euscaphic acid | Triterpenoids | Q07869 | 0.112467055 | 11 |
| 3046 | euscaphic acid | Triterpenoids | Q08499 | 0.31881944 | 14 |
| 3047 | euscaphic acid | Triterpenoids | Q13133 | 0.216325973 | 16 |
| 3048 | euscaphic acid | Triterpenoids | Q13258 | 0.113015591 | 4 |
| 3049 | euscaphic acid | Triterpenoids | Q13547 | 0.110937969 | 6 |
| 3050 | euscaphic acid | Triterpenoids | Q15722 | 0.111722841 | 8 |
| 3051 | euscaphic acid | Triterpenoids | Q16850 | 0.152847021 | 14 |
| 3052 | euscaphic acid | Triterpenoids | Q7Z2W7 | 0.106776816 | 5 |
| 3053 | euscaphic acid | Triterpenoids | Q8TDU6 | 0.111722841 | 8 |
| 3054 | euscaphic acid | Triterpenoids | Q92731 | 0.117586956 | 23 |
| 3055 | euscaphic acid | Triterpenoids | Q96RI1 | 0.11050814 | 7 |
| 3056 | euscaphic acid | Triterpenoids | Q99720 | 0.110060881 | 8 |
| 3057 | euscaphic acid | Triterpenoids | Q9UHC9 | 0.28130208 | 13 |
| 3058 | euscaphic acid | Triterpenoids | Q9Y5Y4 | 0.113671543 | 7 |
| 3059 | Methylene tanshinquinone (methylenetanshinone) | Diterpenoids | O00748 | 0.259386615 | 47 |
| 3060 | Methylene tanshinquinone (methylenetanshinone) | Diterpenoids | O14746 | 0.14728518 | 27 |
| 3061 | Methylene tanshinquinone (methylenetanshinone) | Diterpenoids | O75530 Q15022 Q15910 | 0.341964133 | 19 |
| 3062 | Methylene tanshinquinone (methylenetanshinone) | Diterpenoids | P00533 | 0.104151342 | 24 |
| 3063 | Methylene tanshinquinone (methylenetanshinone) | Diterpenoids | P08575 | 0.109148612 | 27 |
| 3064 | Methylene tanshinquinone (methylenetanshinone) | Diterpenoids | P10275 | 0.132131799 | 34 |
| 3065 | Methylene tanshinquinone (methylenetanshinone) | Diterpenoids | P11309 | 0.104805981 | 13 |
| 3066 | Methylene tanshinquinone (methylenetanshinone) | Diterpenoids | P11511 | 0.139056613 | 29 |
| 3067 | Methylene tanshinquinone (methylenetanshinone) | Diterpenoids | P11940 | 0.1046882 | 6 |
| 3068 | Methylene tanshinquinone (methylenetanshinone) | Diterpenoids | P12268 | 0.104524224 | 13 |
| 3069 | Methylene tanshinquinone (methylenetanshinone) | Diterpenoids | P15121 | 0.267155221 | 39 |
| 3070 | Methylene tanshinquinone (methylenetanshinone) | Diterpenoids | P16083 | 0.112425971 | 14 |
| 3071 | Methylene tanshinquinone (methylenetanshinone) | Diterpenoids | P21397 | 0.102816353 | 15 |
| 3072 | Methylene tanshinquinone (methylenetanshinone) | Diterpenoids | P22736 | 0.100039999 | 6 |
| 3073 | Methylene tanshinquinone (methylenetanshinone) | Diterpenoids | P23141 | 0.275271523 | 31 |
| 3074 | Methylene tanshinquinone (methylenetanshinone) | Diterpenoids | P25025 | 0.100320282 | 13 |
| 3075 | Methylene tanshinquinone (methylenetanshinone) | Diterpenoids | P27338 | 0.105021471 | 16 |
| 3076 | Methylene tanshinquinone (methylenetanshinone) | Diterpenoids | P29350 | 0.204438393 | 44 |
| 3077 | Methylene tanshinquinone (methylenetanshinone) | Diterpenoids | P30305 | 0.185734372 | 40 |
| 3078 | Methylene tanshinquinone (methylenetanshinone) | Diterpenoids | P30307 | 0.105546712 | 12 |
| 3079 | Methylene tanshinquinone (methylenetanshinone) | Diterpenoids | P35348 | 0.101281367 | 4 |
| 3080 | Methylene tanshinquinone (methylenetanshinone) | Diterpenoids | P35968 | 0.105052994 | 26 |
| 3081 | Methylene tanshinquinone (methylenetanshinone) | Diterpenoids | P48039 | 0.101354935 | 19 |
| 3082 | Methylene tanshinquinone (methylenetanshinone) | Diterpenoids | P48147 | 0.107233003 | 22 |
| 3083 | Methylene tanshinquinone (methylenetanshinone) | Diterpenoids | P49286 | 0.101354935 | 19 |
| 3084 | Methylene tanshinquinone (methylenetanshinone) | Diterpenoids | Q06124 | 0.211032909 | 40 |
| 3085 | Methylene tanshinquinone (methylenetanshinone) | Diterpenoids | Q07820 | 0.101122731 | 4 |
| 3086 | Methylene tanshinquinone (methylenetanshinone) | Diterpenoids | Q09028 Q16576 O75530 Q15022 Q15910 | 0.142197972 | 14 |
| 3087 | Methylene tanshinquinone (methylenetanshinone) | Diterpenoids | Q16539 | 0.105438848 | 20 |
| 3088 | Methylene tanshinquinone (methylenetanshinone) | Diterpenoids | Q86V86 | 0.100967138 | 7 |
| 3089 | Methylene tanshinquinone (methylenetanshinone) | Diterpenoids | Q99572 | 0.10437669 | 25 |
| 3090 | Methylene tanshinquinone (methylenetanshinone) | Diterpenoids | Q9P1W9 | 0.103344117 | 12 |
| 3091 | methylenedihydrotanshinone | Diterpenoids | B2RXH2 | 0.104871629 | 12 |
| 3092 | methylenedihydrotanshinone | Diterpenoids | O00748 | 0.259386615 | 47 |
| 3093 | methylenedihydrotanshinone | Diterpenoids | O95551 | 0.106334502 | 10 |
| 3094 | methylenedihydrotanshinone | Diterpenoids | P00533 | 0.104151342 | 24 |
| 3095 | methylenedihydrotanshinone | Diterpenoids | P04626 | 0.104502793 | 11 |
| 3096 | methylenedihydrotanshinone | Diterpenoids | P08575 | 0.109148612 | 27 |
| 3097 | methylenedihydrotanshinone | Diterpenoids | P11387 | 0.113455556 | 13 |
| 3098 | methylenedihydrotanshinone | Diterpenoids | P11388 | 0.106757964 | 8 |
| 3099 | methylenedihydrotanshinone | Diterpenoids | P14902 | 0.110454469 | 24 |
| 3100 | methylenedihydrotanshinone | Diterpenoids | P15121 | 0.267155221 | 39 |
| 3101 | methylenedihydrotanshinone | Diterpenoids | P17948 | 0.105554897 | 13 |
| 3102 | methylenedihydrotanshinone | Diterpenoids | P22303 | 0.287353637 | 26 |
| 3103 | methylenedihydrotanshinone | Diterpenoids | P23141 | 0.275271523 | 31 |
| 3104 | methylenedihydrotanshinone | Diterpenoids | P28472 P18507 P14867 | 0.103855577 | 16 |
| 3105 | methylenedihydrotanshinone | Diterpenoids | P28472 P18507 P31644 | 0.103791839 | 19 |
| 3106 | methylenedihydrotanshinone | Diterpenoids | P28472 P34903 P18507 | 0.103855577 | 16 |
| 3107 | methylenedihydrotanshinone | Diterpenoids | P28562 | 0.104003317 | 12 |
| 3108 | methylenedihydrotanshinone | Diterpenoids | P29350 | 0.204438393 | 44 |
| 3109 | methylenedihydrotanshinone | Diterpenoids | P35968 | 0.105052994 | 26 |
| 3110 | methylenedihydrotanshinone | Diterpenoids | P40763 | 0.268996335 | 13 |
| 3111 | methylenedihydrotanshinone | Diterpenoids | P42574 | 0.10400953 | 13 |
| 3112 | methylenedihydrotanshinone | Diterpenoids | P47869 P28472 P18507 | 0.104254314 | 15 |
| 3113 | methylenedihydrotanshinone | Diterpenoids | P51532 | 0.1046882 | 4 |
| 3114 | methylenedihydrotanshinone | Diterpenoids | P55210 | 0.10400953 | 13 |
| 3115 | methylenedihydrotanshinone | Diterpenoids | Q02083 | 0.102416978 | 6 |
| 3116 | methylenedihydrotanshinone | Diterpenoids | Q06124 | 0.211032909 | 40 |
| 3117 | methylenedihydrotanshinone | Diterpenoids | Q86U86 | 0.1046882 | 4 |
| 3118 | methylenedihydrotanshinone | Diterpenoids | Q9NR96 | 0.108367851 | 6 |
| 3119 | methylenedihydrotanshinone | Diterpenoids | Q9UDY8 | 0.107158923 | 10 |
| 3120 | salvinal (XH-14,5-(3-hydroxypropyl)-7-methoxy-2-(3′-methoxy-4′-hydroxyphenyl)-3-benzo[b]furancarbaldehyde) | Others | O00329 | 0.105574288 | 6 |
| 3121 | salvinal (XH-14,5-(3-hydroxypropyl)-7-methoxy-2-(3′-methoxy-4′-hydroxyphenyl)-3-benzo[b]furancarbaldehyde) | Others | O14757 | 0.106739614 | 8 |
| 3122 | salvinal (XH-14,5-(3-hydroxypropyl)-7-methoxy-2-(3′-methoxy-4′-hydroxyphenyl)-3-benzo[b]furancarbaldehyde) | Others | O60218 | 0.499225177 | 13 |
| 3123 | salvinal (XH-14,5-(3-hydroxypropyl)-7-methoxy-2-(3′-methoxy-4′-hydroxyphenyl)-3-benzo[b]furancarbaldehyde) | Others | O60674 | 0.103305945 | 11 |
| 3124 | salvinal (XH-14,5-(3-hydroxypropyl)-7-methoxy-2-(3′-methoxy-4′-hydroxyphenyl)-3-benzo[b]furancarbaldehyde) | Others | O96020 P24941 P24864 | 0.104035866 | 9 |
| 3125 | salvinal (XH-14,5-(3-hydroxypropyl)-7-methoxy-2-(3′-methoxy-4′-hydroxyphenyl)-3-benzo[b]furancarbaldehyde) | Others | P00533 | 0.104151342 | 24 |
| 3126 | salvinal (XH-14,5-(3-hydroxypropyl)-7-methoxy-2-(3′-methoxy-4′-hydroxyphenyl)-3-benzo[b]furancarbaldehyde) | Others | P00746 | 0.101536397 | 5 |
| 3127 | salvinal (XH-14,5-(3-hydroxypropyl)-7-methoxy-2-(3′-methoxy-4′-hydroxyphenyl)-3-benzo[b]furancarbaldehyde) | Others | P03956 | 0.109842942 | 8 |
| 3128 | salvinal (XH-14,5-(3-hydroxypropyl)-7-methoxy-2-(3′-methoxy-4′-hydroxyphenyl)-3-benzo[b]furancarbaldehyde) | Others | P04035 | 0.166864971 | 20 |
| 3129 | salvinal (XH-14,5-(3-hydroxypropyl)-7-methoxy-2-(3′-methoxy-4′-hydroxyphenyl)-3-benzo[b]furancarbaldehyde) | Others | P04629 | 0.107046621 | 8 |
| 3130 | salvinal (XH-14,5-(3-hydroxypropyl)-7-methoxy-2-(3′-methoxy-4′-hydroxyphenyl)-3-benzo[b]furancarbaldehyde) | Others | P04818 | 0.111100851 | 4 |
| 3131 | salvinal (XH-14,5-(3-hydroxypropyl)-7-methoxy-2-(3′-methoxy-4′-hydroxyphenyl)-3-benzo[b]furancarbaldehyde) | Others | P05186 | 0.104336352 | 9 |
| 3132 | salvinal (XH-14,5-(3-hydroxypropyl)-7-methoxy-2-(3′-methoxy-4′-hydroxyphenyl)-3-benzo[b]furancarbaldehyde) | Others | P06493 | 0.102732601 | 7 |
| 3133 | salvinal (XH-14,5-(3-hydroxypropyl)-7-methoxy-2-(3′-methoxy-4′-hydroxyphenyl)-3-benzo[b]furancarbaldehyde) | Others | P07333 | 0.104063456 | 9 |
| 3134 | salvinal (XH-14,5-(3-hydroxypropyl)-7-methoxy-2-(3′-methoxy-4′-hydroxyphenyl)-3-benzo[b]furancarbaldehyde) | Others | P07949 | 0.103471701 | 8 |
| 3135 | salvinal (XH-14,5-(3-hydroxypropyl)-7-methoxy-2-(3′-methoxy-4′-hydroxyphenyl)-3-benzo[b]furancarbaldehyde) | Others | P08253 | 0.106581489 | 9 |
| 3136 | salvinal (XH-14,5-(3-hydroxypropyl)-7-methoxy-2-(3′-methoxy-4′-hydroxyphenyl)-3-benzo[b]furancarbaldehyde) | Others | P08254 | 0.109296209 | 8 |
| 3137 | salvinal (XH-14,5-(3-hydroxypropyl)-7-methoxy-2-(3′-methoxy-4′-hydroxyphenyl)-3-benzo[b]furancarbaldehyde) | Others | P10275 | 0.132131799 | 34 |
| 3138 | salvinal (XH-14,5-(3-hydroxypropyl)-7-methoxy-2-(3′-methoxy-4′-hydroxyphenyl)-3-benzo[b]furancarbaldehyde) | Others | P11309 | 0.104805981 | 13 |
| 3139 | salvinal (XH-14,5-(3-hydroxypropyl)-7-methoxy-2-(3′-methoxy-4′-hydroxyphenyl)-3-benzo[b]furancarbaldehyde) | Others | P11362 | 0.105847024 | 4 |
| 3140 | salvinal (XH-14,5-(3-hydroxypropyl)-7-methoxy-2-(3′-methoxy-4′-hydroxyphenyl)-3-benzo[b]furancarbaldehyde) | Others | P11511 | 0.139056613 | 29 |
| 3141 | salvinal (XH-14,5-(3-hydroxypropyl)-7-methoxy-2-(3′-methoxy-4′-hydroxyphenyl)-3-benzo[b]furancarbaldehyde) | Others | P12268 | 0.104524224 | 13 |
| 3142 | salvinal (XH-14,5-(3-hydroxypropyl)-7-methoxy-2-(3′-methoxy-4′-hydroxyphenyl)-3-benzo[b]furancarbaldehyde) | Others | P14780 | 0.106689076 | 6 |
| 3143 | salvinal (XH-14,5-(3-hydroxypropyl)-7-methoxy-2-(3′-methoxy-4′-hydroxyphenyl)-3-benzo[b]furancarbaldehyde) | Others | P17252 | 0.102754312 | 7 |
| 3144 | salvinal (XH-14,5-(3-hydroxypropyl)-7-methoxy-2-(3′-methoxy-4′-hydroxyphenyl)-3-benzo[b]furancarbaldehyde) | Others | P17948 | 0.105554897 | 13 |
| 3145 | salvinal (XH-14,5-(3-hydroxypropyl)-7-methoxy-2-(3′-methoxy-4′-hydroxyphenyl)-3-benzo[b]furancarbaldehyde) | Others | P20248 P24941 | 0.112800203 | 4 |
| 3146 | salvinal (XH-14,5-(3-hydroxypropyl)-7-methoxy-2-(3′-methoxy-4′-hydroxyphenyl)-3-benzo[b]furancarbaldehyde) | Others | P20701 | 0.127026321 | 4 |
| 3147 | salvinal (XH-14,5-(3-hydroxypropyl)-7-methoxy-2-(3′-methoxy-4′-hydroxyphenyl)-3-benzo[b]furancarbaldehyde) | Others | P22303 | 0.287353637 | 26 |
| 3148 | salvinal (XH-14,5-(3-hydroxypropyl)-7-methoxy-2-(3′-methoxy-4′-hydroxyphenyl)-3-benzo[b]furancarbaldehyde) | Others | P22894 | 0.111100851 | 4 |
| 3149 | salvinal (XH-14,5-(3-hydroxypropyl)-7-methoxy-2-(3′-methoxy-4′-hydroxyphenyl)-3-benzo[b]furancarbaldehyde) | Others | P23458 | 0.102916912 | 11 |
| 3150 | salvinal (XH-14,5-(3-hydroxypropyl)-7-methoxy-2-(3′-methoxy-4′-hydroxyphenyl)-3-benzo[b]furancarbaldehyde) | Others | P24723 | 0.11185669 | 11 |
| 3151 | salvinal (XH-14,5-(3-hydroxypropyl)-7-methoxy-2-(3′-methoxy-4′-hydroxyphenyl)-3-benzo[b]furancarbaldehyde) | Others | P24941 P78396 P20248 | 0.108328679 | 5 |
| 3152 | salvinal (XH-14,5-(3-hydroxypropyl)-7-methoxy-2-(3′-methoxy-4′-hydroxyphenyl)-3-benzo[b]furancarbaldehyde) | Others | P27361 | 0.105473292 | 5 |
| 3153 | salvinal (XH-14,5-(3-hydroxypropyl)-7-methoxy-2-(3′-methoxy-4′-hydroxyphenyl)-3-benzo[b]furancarbaldehyde) | Others | P28482 | 0.111100851 | 4 |
| 3154 | salvinal (XH-14,5-(3-hydroxypropyl)-7-methoxy-2-(3′-methoxy-4′-hydroxyphenyl)-3-benzo[b]furancarbaldehyde) | Others | P29274 | 0.103202685 | 27 |
| 3155 | salvinal (XH-14,5-(3-hydroxypropyl)-7-methoxy-2-(3′-methoxy-4′-hydroxyphenyl)-3-benzo[b]furancarbaldehyde) | Others | P30291 | 0.103962076 | 3 |
| 3156 | salvinal (XH-14,5-(3-hydroxypropyl)-7-methoxy-2-(3′-methoxy-4′-hydroxyphenyl)-3-benzo[b]furancarbaldehyde) | Others | P30556 | 0.113117188 | 7 |
| 3157 | salvinal (XH-14,5-(3-hydroxypropyl)-7-methoxy-2-(3′-methoxy-4′-hydroxyphenyl)-3-benzo[b]furancarbaldehyde) | Others | P31639 | 0.109951316 | 5 |
| 3158 | salvinal (XH-14,5-(3-hydroxypropyl)-7-methoxy-2-(3′-methoxy-4′-hydroxyphenyl)-3-benzo[b]furancarbaldehyde) | Others | P34998 | 0.110755665 | 3 |
| 3159 | salvinal (XH-14,5-(3-hydroxypropyl)-7-methoxy-2-(3′-methoxy-4′-hydroxyphenyl)-3-benzo[b]furancarbaldehyde) | Others | P35916 | 0.105748254 | 5 |
| 3160 | salvinal (XH-14,5-(3-hydroxypropyl)-7-methoxy-2-(3′-methoxy-4′-hydroxyphenyl)-3-benzo[b]furancarbaldehyde) | Others | P35968 | 0.105052994 | 26 |
| 3161 | salvinal (XH-14,5-(3-hydroxypropyl)-7-methoxy-2-(3′-methoxy-4′-hydroxyphenyl)-3-benzo[b]furancarbaldehyde) | Others | P37023 | 0.111434828 | 4 |
| 3162 | salvinal (XH-14,5-(3-hydroxypropyl)-7-methoxy-2-(3′-methoxy-4′-hydroxyphenyl)-3-benzo[b]furancarbaldehyde) | Others | P42338 | 0.107541648 | 9 |
| 3163 | salvinal (XH-14,5-(3-hydroxypropyl)-7-methoxy-2-(3′-methoxy-4′-hydroxyphenyl)-3-benzo[b]furancarbaldehyde) | Others | P42345 | 0.10907142 | 6 |
| 3164 | salvinal (XH-14,5-(3-hydroxypropyl)-7-methoxy-2-(3′-methoxy-4′-hydroxyphenyl)-3-benzo[b]furancarbaldehyde) | Others | P43405 | 0.106351077 | 5 |
| 3165 | salvinal (XH-14,5-(3-hydroxypropyl)-7-methoxy-2-(3′-methoxy-4′-hydroxyphenyl)-3-benzo[b]furancarbaldehyde) | Others | P45452 | 0.106586322 | 6 |
| 3166 | salvinal (XH-14,5-(3-hydroxypropyl)-7-methoxy-2-(3′-methoxy-4′-hydroxyphenyl)-3-benzo[b]furancarbaldehyde) | Others | P45983 | 0.105088123 | 17 |
| 3167 | salvinal (XH-14,5-(3-hydroxypropyl)-7-methoxy-2-(3′-methoxy-4′-hydroxyphenyl)-3-benzo[b]furancarbaldehyde) | Others | P45984 | 0.10939337 | 4 |
| 3168 | salvinal (XH-14,5-(3-hydroxypropyl)-7-methoxy-2-(3′-methoxy-4′-hydroxyphenyl)-3-benzo[b]furancarbaldehyde) | Others | P55263 | 0.107485158 | 3 |
| 3169 | salvinal (XH-14,5-(3-hydroxypropyl)-7-methoxy-2-(3′-methoxy-4′-hydroxyphenyl)-3-benzo[b]furancarbaldehyde) | Others | P56373 | 0.107098941 | 5 |
| 3170 | salvinal (XH-14,5-(3-hydroxypropyl)-7-methoxy-2-(3′-methoxy-4′-hydroxyphenyl)-3-benzo[b]furancarbaldehyde) | Others | P78536 | 0.104291862 | 9 |
| 3171 | salvinal (XH-14,5-(3-hydroxypropyl)-7-methoxy-2-(3′-methoxy-4′-hydroxyphenyl)-3-benzo[b]furancarbaldehyde) | Others | Q00796 | 0.107710558 | 5 |
| 3172 | salvinal (XH-14,5-(3-hydroxypropyl)-7-methoxy-2-(3′-methoxy-4′-hydroxyphenyl)-3-benzo[b]furancarbaldehyde) | Others | Q02750 | 0.106463868 | 11 |
| 3173 | salvinal (XH-14,5-(3-hydroxypropyl)-7-methoxy-2-(3′-methoxy-4′-hydroxyphenyl)-3-benzo[b]furancarbaldehyde) | Others | Q05655 | 0.102598878 | 5 |
| 3174 | salvinal (XH-14,5-(3-hydroxypropyl)-7-methoxy-2-(3′-methoxy-4′-hydroxyphenyl)-3-benzo[b]furancarbaldehyde) | Others | Q08828 | 0.102451863 | 4 |
| 3175 | salvinal (XH-14,5-(3-hydroxypropyl)-7-methoxy-2-(3′-methoxy-4′-hydroxyphenyl)-3-benzo[b]furancarbaldehyde) | Others | Q13547 | 0.110937969 | 6 |
| 3176 | salvinal (XH-14,5-(3-hydroxypropyl)-7-methoxy-2-(3′-methoxy-4′-hydroxyphenyl)-3-benzo[b]furancarbaldehyde) | Others | Q13627 | 0.104383674 | 7 |
| 3177 | salvinal (XH-14,5-(3-hydroxypropyl)-7-methoxy-2-(3′-methoxy-4′-hydroxyphenyl)-3-benzo[b]furancarbaldehyde) | Others | Q16539 | 0.105438848 | 20 |
| 3178 | salvinal (XH-14,5-(3-hydroxypropyl)-7-methoxy-2-(3′-methoxy-4′-hydroxyphenyl)-3-benzo[b]furancarbaldehyde) | Others | Q16584 | 0.104189154 | 3 |
| 3179 | salvinal (XH-14,5-(3-hydroxypropyl)-7-methoxy-2-(3′-methoxy-4′-hydroxyphenyl)-3-benzo[b]furancarbaldehyde) | Others | Q16875 | 0.101683939 | 4 |
| 3180 | salvinal (XH-14,5-(3-hydroxypropyl)-7-methoxy-2-(3′-methoxy-4′-hydroxyphenyl)-3-benzo[b]furancarbaldehyde) | Others | Q86V86 | 0.100967138 | 7 |
| 3181 | salvinal (XH-14,5-(3-hydroxypropyl)-7-methoxy-2-(3′-methoxy-4′-hydroxyphenyl)-3-benzo[b]furancarbaldehyde) | Others | Q9HBH9 | 0.10939337 | 4 |
| 3182 | salvinal (XH-14,5-(3-hydroxypropyl)-7-methoxy-2-(3′-methoxy-4′-hydroxyphenyl)-3-benzo[b]furancarbaldehyde) | Others | Q9NWZ3 | 0.105136637 | 5 |
| 3183 | salvinal (XH-14,5-(3-hydroxypropyl)-7-methoxy-2-(3′-methoxy-4′-hydroxyphenyl)-3-benzo[b]furancarbaldehyde) | Others | Q9NZJ5 | 0.102919059 | 6 |
| 3184 | salvinal (XH-14,5-(3-hydroxypropyl)-7-methoxy-2-(3′-methoxy-4′-hydroxyphenyl)-3-benzo[b]furancarbaldehyde) | Others | Q9P1W9 | 0.103344117 | 12 |
| 3185 | salvinal (XH-14,5-(3-hydroxypropyl)-7-methoxy-2-(3′-methoxy-4′-hydroxyphenyl)-3-benzo[b]furancarbaldehyde) | Others | Q9UBN7 | 0.106238718 | 3 |
| 3186 | salvinal (XH-14,5-(3-hydroxypropyl)-7-methoxy-2-(3′-methoxy-4′-hydroxyphenyl)-3-benzo[b]furancarbaldehyde) | Others | Q9Y233 | 0.108085897 | 14 |

**Supplementary Table 3.** Compounds identified in lipophilic constituents in Salvia miltiorrhiza by UPLC/Q-TOF-MS

| No | t_R_(min) | identification | Formula | M+X | Expected (m/z) | Detected (m/z) | MS/MS | | | Error (ppm) |
| --- | --- | --- | --- | --- | --- | --- | --- | --- | --- | --- |
| C1 | 8.717 | 1,2,15,16-tetrahydrotanshiquinone (1,2,15,16-tetrahydrotanshinone I,Trijuganone B,Tetrahydro tanshinone I) | C_18_H_16_O_3_ | (M+H)^+^ | 281.1172 | 281.1171 | 263.1065(75.11)  235.1114(100.00)  192.0926(21.95) | | | 0.01 |
| C2 | 24.833 | salvinone | C_18_H_20_O_2_ | (M+H)+ | 269.1536 | 269.1570 |  | | | -0.23 |
| C3 | 22.479 | 1,2-Didehydrocryptotanshinone | C_19_H_18_O_3_ | (M+H)^+^ | 295.1329 | 295.1323 | 277.1214(67.54)  280.1073(31.15)  266.0937（37.15）  249.1267（100） | | | 3.57 |
| C4 | 19.677 | 1,2-Dihydrotanshinquinone (1,2-Dihydrotanshinone I,1,2-Dihydrotanshinone) | C_18_H_14_O_3_ | (M+H)^+^ | 279.1016 | 279.1024 |  | | | -2 |
| C5 | 21.517 | 15,16-dihydrotanshinol B | C_18_H_18_O_4_ | (M+H)^+^ | 299.1278 | 299.1287 |  | | | -2.81 |
| C6 | 5.126 | 17-hydroxycryptotanshinone | C_19_H_20_O_4_ | (M+H)^+^ | 313.1434 | 313.1431 | 295.1326（100.0）  277.1227（55.73）  267.1381（90.06） | | | 1.43 |
| C7 | 1.604 | 17-hydroxytanshindiol B | C_18_H_18_O_5_ | (M+H)^+^ | 315.1227 | 315.1230 | 251.1055（100）  223.1011（93.00） | | | -0.72 |
| C8 | 18.483 | 1-hydroxytaxinine A | C_26_H_36_O_9_ | (M+H)^+^ | 493.2432 | 493.2447 |  | | | -2.95 |
| C9 | 22.214 | 1-ketoaethiopinone | C_20_H_22_O_3_ | (M+H)^+^ | 311.1642 | 311.1647 | 267.1382（100.0）  252.1141（18.35） | | | -1.39 |
| C10 | 7.126 | 1-ketoisocryptotanshinone | C_19_H_18_O_4_ | (M+H)^+^ | 311.1278 | 311.1280 |  | | | -0.94 |
| C11 | 15.200 | 1-oxomiltirone | C_19_H_20_O_3_ | (M+H)^+^ | 297.1485 | 297.1494 |  | | | -2.19 |
| C12 | 19.030 | 1R-hydroxymiltirone | C_19_H_22_O_3_ | (M+H)^+^ | 299.1642 | 299.1646 |  | | | -1.56 |
| C13 | 22.495 | 2,3-Didehydrocryptotanshinone | C_19_H_18_O_3_ | (M+H)^+^ | 295.1329 | 295.1337 |  | | | -0.28 |
| C14 | 22.694 | 2alpha-acetoxysugiol | C_22_H_30_O_4_ | (M+H)^+^ | 359.2217 | 359.2216 |  | | | 0.22 |
| C15 | 6.645 | 2-hydroxydihydroisotanshinone I | C_18_H_14_O_4_ | (M+H)^+^ | 295.0965 | 295.0964 |  | | | -0.07 |
| C16 | 22.810 | 2-Isopropyl-8-methylphenanthrene-3,4-dione (Ro 09-0680,miltirone I similar) | C_18_H_16_O_2_ | (M+H)^+^ | 265.1223 | 265.1225 |  | | | -0.95 |
| C17 | 24.435 | 3-hydroxycyptotanshinone | C_19_H_20_O_4_ | (M+H)^+^ | 313.1434 | 313.1447 | 295.1304（15.44）  227.1053（100） | | | -3.64 |
| C18 | 7.822 | 3-hydroxymethylenetanshinquinone (hydroxymethylenetanshinone,3-beta-Hydroxymethylenetanshiquinone) | C_18_H_14_O_4_ | (M+H)^+^ | 295.0965 | 295.0968 | 277.0854（53.35）  249.0909（100.0） | | | -0.93 |
| C19 | 12.464 | 3-hydroxytanshinone | C_19_H_18_O_4_ | (M+H)^+^ | 311.1278 | 311.1278 |  | | | -1.48 |
| C20 | 10.757 | 3-hydroxytanshinone IIB | C_19_H_18_O_5_ | (M+H)^+^ | 327.1227 | 327.1221 | 313.1425（42.12）  272.1004（13.27）  251.1053（100.0） | | | 1.23 |
| C21 | 14.603 | 3-Oxosapriparaquinone | C_20_H_24_O_4_ | (M+H)^+^ | 329.1747 | 329.1743 | 279.1006（100）  55.0526（6.80） | | | 1.22 |
| C22 | 25.364 | 5,6-dehydrosugiol | C_20_H_26_O_2_ | (M+H)^+^ | 299.2006 | 299.2008 |  | | | -0.71 |
| C23 | 22.893 | 6,12-Dihydroxyabieta-5,8,11,13-tetraen-7-one (montbretol) | C_20_H_26_O_3_ | (M+H)^+^ | 315.1955 | 315.1954 |  | | | -0.79 |
| C24 | 18.815 | 7β-hydroxy-8,13-abietadiene-11,12-dione | C_20_H_28_O_3_ | (M+H)^+^ | 317.2111 | 317.2119 |  | | | -2.19 |
| C25 | 22.247 | cryptoacetalide | C_18_H_22_O_3_ | (M+H)^+^ | 287.1642 | 287.1648 |  | | | -2.23 |
| C26 | 18.068 | cryptotanshinone （15,17-Dihydrotanshinone IIA） | C_19_H_20_O_3_ | (M+H)^+^ | 297.1485 | 297.1493 | 253.1587（100.0）  251.1431（21.90）  225.1635（14.83） | | | -2.65 |
| C27 | 14.487 | danshenol a | C_21_H_20_O_4_ | (M+H)^+^ | 337.1434 |  | | 337.1432 | 1.04 | |
| C28 | 6.794 | danshenxinkun A (neotanshinone A,Tanshiquinone A) | C_18_H_16_O_4_ | (M+H)^+^ | 297.1121 | 297.1127 | 279.1021（100）  251.1089（19.33）  223.1121（5.13）  209.0868（20.65） | | | -1.61 |
| C29 | 14.034 | danshenxinkun B(tanshiquinone B,neotanshinone B) | C_18_H_16_O_3_ | (M+H)^+^ | 281.1172 | 281.1171 |  | | | 0.54 |
| C30 | 10.093 | danshenxinkun C (Neotanshinone C,tanshiquinone C) | C_16_H_12_O_3_ | (M+H)^+^ | 253.0859 | 253.0859 | 213.0917（92.97）  185.0920（100.0） | | | -0.09 |
| C31 | 12.381 | danshenxinkun D | C_21_H_20_O_4_ | (M+H)^+^ | 337.1434 | 337.1424 |  | | | 3.11 |
| C32 | 26.143 | Danshinspiroketallactone (Danshenspiroketallactone) | C_17_H_16_O_3_ | (M+H)^+^ | 269.1172 | 269.1172 |  | | | 0.19 |
| C33 | 12.481 | dehydromiltirone (1,2-didehydromiltirone,1-dehydromiltirone) | C_19_H_20_O_2_ | (M+H)^+^ | 281.1536 | 281.1541 | 253.1600（100）  221.0976（84.69）  263.1441（22.33） | | | -1.64 |
| C34 | 21.368 | Dehydrotanshinone II A (1,2-Didehydrotanshinone IIA,1,2-Dehydrotanshinone II A,delta1-Dehydrotanshinone II(A)) | C_19_H_16_O_3_ | (M+H)^+^ | 293.1172 | 293.1176 | 275.0934（100.0）  276.0956（18.32）  247.0985（14.97） | | | -1.33 |
| C35 | 13.625 | Dihydrotanshinone I (15,16-Dihydrotanshinone I) | C_18_H_14_O_3_ | (M+H)^+^ | 279.1016 | 279.1023 | 261.0909（100）  233.0967（83.60）  205.1014（34.85） | | | -2.53 |
| C36 | 19.511 | tanshinone I | C_18_H_12_O_3_ | (M+H)^+^ | 277.0859 | 277.0860 | 249.0907（100.0）  231.0802（7.5）  221.0962（8.8） | | | -0.61 |
| C37 | 24.153 | tanshinone II A | C_19_H_18_O_3_ | (M+H)^+^ | 295.1329 | 295.1330 | 277.1220（66.91）  262.0963（54.74）  252.0781（61.46）  249.1262（100.0）  206.1083（46.06） | | | -1.48 |
| C38 | 23.772 | demethylcryptojaponol (11-Hydroxysugiol) | C_20_H_28_O_3_ | (M+H)^+^ | 317.2111 | 317.2105 |  | | | 0.71 |
| C39 | 15.382 | deoxyneocryptotanshinone | C_19_H_22_O_3_ | (M+H)^+^ | 299.1642 | 299.1644 |  | | | -0.79 |
| C40 | 6.860 | dihydroisotanshinone I (isodihydrotanshinone) | C_18_H_14_O_3_ | (M+H)^+^ | 279.1016 | 279.1018 | 265.1065（75.11）  235.1114（100.0） | | | -0.7 |
| C41 | 13.675 | dihydroisotanshinone II | C_18_H_14_O_3_ | (M+H)^+^ | 279.1016 | 279.1024 |  | | | -3.49 |
| C42 | 5.468 | dihydronortanshinone | C_17_H_14_O_4_ | (M+H)^+^ | 283.0965 | 283.0969 |  | | | -1.28 |
| C43 | 22.247 | Epi-Cryptoacetalide | C_18_H_22_O_3_ | (M+H)^+^ | 287.1642 | 287.1648 |  | | | -2.23 |
| C44 | 26.143 | epidanshenspiroketallactone | C_17_H_16_O_3_ | (M+H)^+^ | 269.1172 | 269.1172 |  | | | 0.19 |
| C45 | 20.622 | epi-Danshenspiroketallactone | C_20_H_20_O_5_ | (M+H)^+^ | 341.1384 | 341.1384 |  | | | -0.89 |
| C46 | 20.622 | ferruginol | C_20_H_30_O | (M+H)^+^ | 287.2369 | 287.2368 | 271.1669（100.0）  69.0697（19.74） | | | 1.72 |
| C47 | 12.579 | hydroxytanshinone IIA (hydroxytanshinone) | C_19_H_18_O_4_ | (M+H)^+^ | 311.1278 | 311.1283 | 312.1589（100.0）  311.1279（71.81）  310.1341（16.38） | | | -1.68 |
| C48 | 12.332 | isocryptotanshinone(isodihydrotanshinone IIA) | C_19_H_20_O_3_ | (M+H)^+^ | 297.1485 | 297.1494 | 281.1169（100.0）  253.1211（7.08） | | | -2.73 |
| C49 | 19.511 | isotanshinone I | C_18_H_12_O_3_ | (M+H)^+^ | 277.0859 | 277.0860 | 261.0917（100）  189.0703（1.31） | | | -0.61 |
| C50 | 19.511 | isotanshinone II | C_18_H_12_O_3_ | (M+H)^+^ | 277.0859 | 277.0860 |  | | | -0.61 |
| C51 | 17.866 | isotanshinone IIA | C_19_H_18_O_3_ | (M+H)^+^ | 295.1329 | 295.1336 | 279.1401（97.97）  251.1429（100.0） | | | -2.29 |
| C52 | 13.111 | isotanshinone IIB (18-hydroxyisotanshinone IIA) | C_19_H_18_O_4_ | (M+H) | 311.1278 | 311.1269 | 279.0958 | | | 0.76 |
| C53 | 18.665 | methyl dihydronortanshinonate（methylcryptotanshinoate） | C_20_H_20_O_5_ | (M+H)^+^ | 341.1384 | 341.1394 |  | | | -2.89 |
| C54 | 14.471 | methylene dihydrotanshinone | C_18_H_16_O_3_ | (M+H)^+^ | 281.1172 | 281.1175 |  | | | -1.62 |
| C55 | 20.920 | Methylenetanshinquinone (methylenetanshinone) | C_18_H_14_O_3_ | (M+H)^+^ | 279.1016 | 279.1029 | 248.0826（20.97）  235.1129（100.0） | | | -4.62 |
| C56 | 24.551 | miltiodiol (Arucadiol) | C_19_H_22_O_3_ | (M+H)^+^ | 299.1642 | 299.1644 |  | | | -0.72 |
| C57 | 24.518 | miltionone I | C_19_H_20_O_4_ | (M+H)^+^ | 313.1434 | 313.1428 |  | | | 0.91 |
| C58 | 24.551 | miltionone II | C_19_H_20_O_4_ | (M+H)^+^ | 313.1434 | 313.1433 |  | | | 0.3 |
| C59 | 23.457 | miltiorin A (2alpha-Acetoxyabieta-8(14),9(11),12-triene-12-ol) | C_22_H_32_O_3_ | (M+H)^+^ | 345.2424 | 345.2415 |  | | | 2.68 |
| C60 | 15.946 | miltiorin B | C_22_H_30_O_5_ | (M+H)^+^ | 375.2116 | 375.2159 |  | | | 2.15 |
| C61 | 24.734 | miltiorin C | C_22_H_28_O_4_ | (M+H)^+^ | 357.206 | 357.2055 |  | | | 1.84 |
| C62 | 15.117 | miltiorin D | C_19_H_22_O_4_ | (M+H)^+^ | 315.1591 | 315.1591 |  | | | -0.34 |
| C63 | 7.540 | miltipolone | C_19_H_24_O_3_ | (M+H)^+^ | 301.1798 | 301.1791 |  | | | -0.7 |
| C64 | 22.810 | miltirone I | C_18_H_16_O_2_ | (M+H)^+^ | 265.1223 | 265.1225 |  | | | -0.95 |
| C65 | 19.776 | monodydroxytanshinone I | C_18_H_12_O_4_ | (M+H)^+^ | 293.0808 | 293.0805 |  | | | 1.31 |
| C66 | 10.508 | neocryptotanshinone （tanshinone V） | C_19_H_22_O_4_ | (M+H)^+^ | 315.1591 | 315.1598 | 297.1496（100.0）  279.1391（36.03） | | | -1.86 |
| C67 | 11.486 | neocryptotanshinone II | C_17_H_18_O_3_ | (M+H)^+^ | 271.1329 | 271.1327 |  | | | -0.82 |
| C68 | 24.435 | neosalvianen | C_21_H_21_NO_2_ | (M+H)^+^ | 320.1645 | 320.1643 | 317.1746（100.0）  305.1476（16.65） | | | -0.34 |
| C69 | 22.147 | neotanshinlactone | C_17_H_12_O_3_ | (M+H)^+^ | 265.0859 | 265.0856 |  | | | 0.53 |
| C70 | 8.850 | nortanshinone | C_17_H_12_O_4_ | (M+H)^+^ | 281.0808 | 281.0811 |  | | | -0.64 |
| C71 | 13.376 | paramiltioic acid | C_19_H_24_O_5_ | (M+H)^+^ | 333.1697 | 333.1696 |  | | | -0.38 |
| C72 | 13.194 | przewaquinone A | C_19_H_18_O_4_ | (M+H)^+^ | 311.1278 | 311.1284 | 292.1300（42.17）  277.1215（100.0）  249.1295（39.32） | | | -0.95 |
| C73 | 8.270 | przewaquinone B | C_18_H_12_O_4_ | (M+H)^+^ | 293.0808 | 293.0811 | 264.1218（100.0）  178.0847（8.94） | | | -0.51 |
| C74 | 15.184 | salviadione | C_19_H_19_NO_2_ | (M+H)^+^ | 294.1489 | 294.1495 |  | | | -1.64 |
| C75 | 16.493 | salviamone | C_18_H_14_O_4_ | (M+H)^+^ | 295.0965 | 295.0961 |  | | | -0.72 |
| C76 | 24.585 | salvianan | C_21_H_23_NO_2_ | (M+H)^+^ | 322.1802 | 322.1804 |  | | | -0.97 |
| C77 | 24.435 | salvianen | C_21_H_21_NO_2_ | (M+H)^+^ | 320.1645 | 320.1643 | 317.1746（100.0）  305.1476（16.65） | | | -0.34 |
| C78 | 21.816 | salviol （2-Hydroxyferruginol） | C_20_H_30_O_2_ | (M+H)^+^ | 303.2319 | 303.2317 |  | | | 0.65 |
| C79 | 15.399 | salviolone | C_18_H_20_O_2_ | (M+H)^+^ | 265.1536 | 265.1535 |  | | | 0.46 |
| C80 | 25.911 | sugiol | C_20_H_28_O_2_ | (M+H)^+^ | 301.2162 | 301.2162 | 285.1862（100） | | | 1.53 |
| C81 | 9.016 | tanshinaldehyde （tanshinaldehyde II） | C_19_H_18_O_4_ | (M+H)^+^ | 311.1278 | 311.1285 |  | | | -1.55 |
| C82 | 13.327 | tanshinaldehyde I （formyltanshinone） | C_18_H_10_O_4_ | (M+H)^+^ | 291.0652 | 291.0658 |  | | | -2.4 |
| C83 | 6.860 | tanshindiol A | C_18_H_16_O_5_ | (M+H)^+^ | 311.1071 | 311.1074 |  | | | -0.97 |
| C84 | 6.396 | tanshindiol B | C_18_H_16_O_5_ | (M+H)^+^ | 311.1071 | 311.1069 | 267.1013（100）  249.0896（55.82） | | | 0.35 |
| C85 | 6.396 | tanshindiol C | C_18_H_16_O_5_ | (M+H)^+^ | 311.1071 | 311.1069 | 295.1324（87.95）  267.1370（92.18） | | | 0.35 |
| C86 | 22.147 | tanshinlactone | C_17_H_12_O_3_ | (M+H)^+^ | 265.0859 | 265.0856 |  | | | 0.53 |
| C87 | 8.270 | tanshinol A (tanshinol I) | C_18_H_12_O_4_ | (M+H)^+^ | 293.0808 | 293.0811 |  | | | -0.51 |
| C88 | 13.161 | tanshinol B (Przewaquinone C) | C_18_H_16_O_4_ | (M+H)^+^ | 297.1121 | 297.1120 | 279.1021（51.07）  261.0916（100.0） | | | -0.6 |
| C89 | 13.277 | tanshinone II B | C_19_H_18_O_4_ | (M+H)^+^ | 311.1278 | 311.1284 | 283.1321（5.05）  279.0968（100.0）  261.0854（46.53） | | | -3.32 |
| C90 | 13.260 | tanshinone VI | C_18_H_16_O_4_ | (M+H)^+^ | 297.1121 | 297.1126 | 279.1021（100）  251.1089（19.33）  223.1121（5.13）  209.0868（20.65） | | | -2.16 |
| C91 | 19.295 | isocucurbitacin d | C_30_H_44_O_7_ | (M+H)^+^ | 517.316 | 517.3179 |  | | | -3.63 |
| C92 | 18.483 | daphneolone | C_17_H_18_O_3_ | (M+H)^+^ | 271.1329 | 271.1330 |  | | | -0.99 |
| C93 | 15.150 | isotenulin | C_17_H_22_O_5_ | (M+H)^+^ | 307.154 | 307.1537 |  | | | 1.88 |
| C94 | 5.534 | Rehmanone C((E)-4-[5-(hydroxymethyl)furan-2-yl]but-3-en-2-one) | C_9_H_10_O_3_ | (M+H)^+^ | 167.0703 | 167.0703 |  | | | 2.33 |
| C95 | 13.360 | salvinal (XH-14,5-(3-hydroxypropyl)-7-methoxy-2-(3′-methoxy-4′-hydroxyphenyl)-3-benzo[b]furancarbaldehyde) | C_20_H_20_O_6_ | (M+H)^+^ | 357.1333 | 357.1325 |  | | | -1.28 |
| C96 | 21.666 | barbinervic acid | C_30_H_48_O_5_ | (M+H)^+^ | 489.3575 | 489.3587 |  | | | -2.54 |
| C97 | 20.473 | Corosolic acid (colosolic acid,2alpha-hydroxyursolic acid) | C_30_H_48_O_4_ | (M+H)^+^ | 473.3625 | 473.3619 | 471.3457（53.57）  249.1848（16.59） | | | 1.37 |
| C98 | 22.860 | euscaphic acid （tormentic acid，Jacarandic acid,2beta-hydroxypomolic acid） | C_30_H_48_O_5_ | (M+H)^+^ | 489.3575 | 489.3586 |  | | | -2.43 |
| C99 | 21.069 | maslinic acid | C_30_H_48_O_4_ | (M+H)^+^ | 473.3525 | 473.3632 | 473.3618（48.45）  437.3434（52.58）  409.3463（67.65） | | | -1.58 |
| C100 | 12.398 | neotigogenin | C_27_H_44_O_3_ | (M+H)^+^ | 417.3363 | 417.3383 |  | | | -4.74- |
| C101 | 19.942 | pomolic acid | C_30_H_48_O_4_ | (M+K)^+^ | 473.3625 | 473.3636 |  | | | -2.33 |
| C102 | 18.732 | przewanoic acid A | C_30_H_46_O_4_ | (M+H)^+^ | 471.3469 | 471.3461 |  | | | 0.9 |
| C103 | 16.394 | przewanoic acid B | C_29_H_42_O_4_ | (M+H)^+^ | 455.3156 | 455.3151 |  | | | 0.27 |
| C104 | 23.092 | ursolic acid | C_30_H_48_O_3_ | (M+H)^+^ | 457.3676 | 457.3694 | 455.3186（93.53）  456.3220（38.06） | | | -3.85 |
| C105 | 26.143 | uvaol | C_30_H_50_O_2_ | (M+H)^+^ | 443.3884 | 443.3863 | 163.1475（1475）  121.0997（84.63） | | | 4.58 |
| C106 | 22.247 | O-methyltaxodine | C_20_H_29_NO_3_ | (M+H)^+^ | 332.222 | 332.2214 |  | | | 1.84 |
| C107 | 16.725 | ailanthoidol | C_19_H_18_O_5_ | (M+H)^+^ | 327.1227 | 327.1225 |  | | | 1.44 |
| C108 | 24.004 | Asiatic acid (Dammarolic acid,Asiantic acid,2alpha,23-Dihydroxyursolic acid) | C_30_H_48_O_5_ | (M+H)^+^ | 489.3575 | 489.3595 | 409.3464（100.0）  421.1610（2.91） | | | -4.21 |
| C109 | 11.486 | heteratisine | C_22_H_33_NO_5_ | (M+H)^+^ | 392.2431 | 392.2429 |  | | | 0.64 |
| C110 | 16.062 | methyl tanshinonate (methyltanshinoate) | C_20_H_18_O_5_ | (M+H)^+^ | 339.1227 | 339.1225 |  | | | 0.78 |
| C111 | 25.099 | miltirone | C_19_H_22_O_2_ | (M+H)^+^ | 283.1693 | 283.1693 | 265.1571（29.21）  223.1115（100.0）  241.1211（25.54） | | | 0.26 |

**Supplementary Table 4.** The potential target proteins involved in Jak-STAT signaling pathway

| Protein name | Uniprot ID | Gene name | target prediction related scores | number of target protein-related compounds |
| --- | --- | --- | --- | --- |
| Protein-tyrosine phosphatase 1C | P29350 | PTPN6 | 0.204438393 | 44 |
| Protein-tyrosine phosphatase 2C | Q06124 | PTPN11 | 0.211032909 | 40 |
| Epidermal growth factor receptor erbB1 | P00533 | EGFR | 0.104151342 | 24 |
| T-cell protein-tyrosine phosphatase | P17706 | PTPN2 | 0.347170021 | 15 |
| PI3-kinase p110-alpha subunit | P42336 | PIK3CA | 0.108042586 | 13 |
| Serine/threonine-protein kinase PIM1 | P11309 | PIM1 | 0.104805981 | 13 |
| Signal transducer and activator of transcription 3 | P40763 | STAT3 | 0.268996335 | 13 |
| Tyrosine-protein kinase JAK3 | P52333 | JAK3 | 0.104243408 | 12 |
| Tyrosine-protein kinase JAK1 | P23458 | JAK1 | 0.102916912 | 11 |
| Tyrosine-protein kinase JAK2 | O60674 | JAK2 | 0.103305945 | 11 |
| PI3-kinase p110-beta subunit | P42338 | PIK3CB | 0.107541648 | 9 |
| Serine/threonine-protein kinase mTOR | P42345 | MTOR | 0.10907142 | 6 |
| PI3-kinase p110-delta subunit | O00329 | PIK3CD | 0.105574288 | 6 |
| Cyclin-dependent kinase 4/cyclin D1 | P24385 P11802 | CCND1 CDK4 | 0.105464394 | 4 |
| Induced myeloid leukemia cell differentiation protein Mcl-1 | Q07820 | MCL1 | 0.101122731 | 4 |
| Serine/threonine-protein kinase AKT2 | P31751 | AKT2 | 0.110657683 | 3 |
| CREB-binding protein/p53 | Q92793 | CREBBP | 0.102416978 | 3 |
| Platelet-derived growth factor receptor alpha | P16234 | PDGFRA | 0.100367415 | 3 |
| Tyrosine-protein kinase TYK2 | P29597 | TYK2 | 0.106959422 | 3 |

**Supplementary Table 5.** The abbreviation list

| abbreviation | full name |
| --- | --- |
| HSCs | hepatic stellate cells |
| LS | Lipophilic constituents in Salvia miltiorrhiza |
| JAK1 | Janus Kinase 1 |
| P-JAK1 | Phosphorylated Janus Kinase 1 |
| STAT3 | Signal Transducer And Activator Of Transcription 3 |
| P-STAT3 | phosphorylated signal transducer and activator of transcription 3 |
| TCM | Traditional Chinese medicine |
| ECM | extracellular matrix |
| PBS | Phosphate buffer solution |
| DMEM | Dulbecco's Modified Eagle's Medium |
| FBS | Fetal Bovine Serum |
| TGFβ1 | transforming growth factor β1 |
| CCK8 | Cell Counting Kit-8 |
| UPLC | Ultra Performance Liquid Chromatography |
| DBE | double bond equivalent |
| PVDF | polyvinylidene difluoride |
| TBST | Tris-buffered saline with 0.1% Tween® 20 detergent |
| CMC-Na | Sodium carboxymethyl cellulose |
| LN | laminin |
| HA | hyaluronic acid |
| ALT | alanine aminotransferase |
| AST | aspartate aminotransferase |
| OD | optical density |
| Hyp | hydroxyproline |
| HE | hematoxylin-eosin |
| α-SMA | Alpha Smooth Muscle Actin |
| PTPN11 | Protein Tyrosine Phosphatase Non-Receptor Type 11 |
| PTPN2 | Protein Tyrosine Phosphatase Non-Receptor Type 2 |
| PTPN6 | Protein Tyrosine Phosphatase Non-Receptor Type 6 |
| PIK3CB | Phosphatidylinositol-4,5-Bisphosphate 3-Kinase Catalytic Subunit Beta |
| PIK3CD | Phosphatidylinositol-4,5-Bisphosphate 3-Kinase Catalytic Subunit Delta |
| PIM1 | Pim-1 Proto-Oncogene, Serine/Threonine Kinase |
| EGFR | epidermal growth factor receptor |

**Supplementary Figure 1.** The Screening process of active compounds in LS


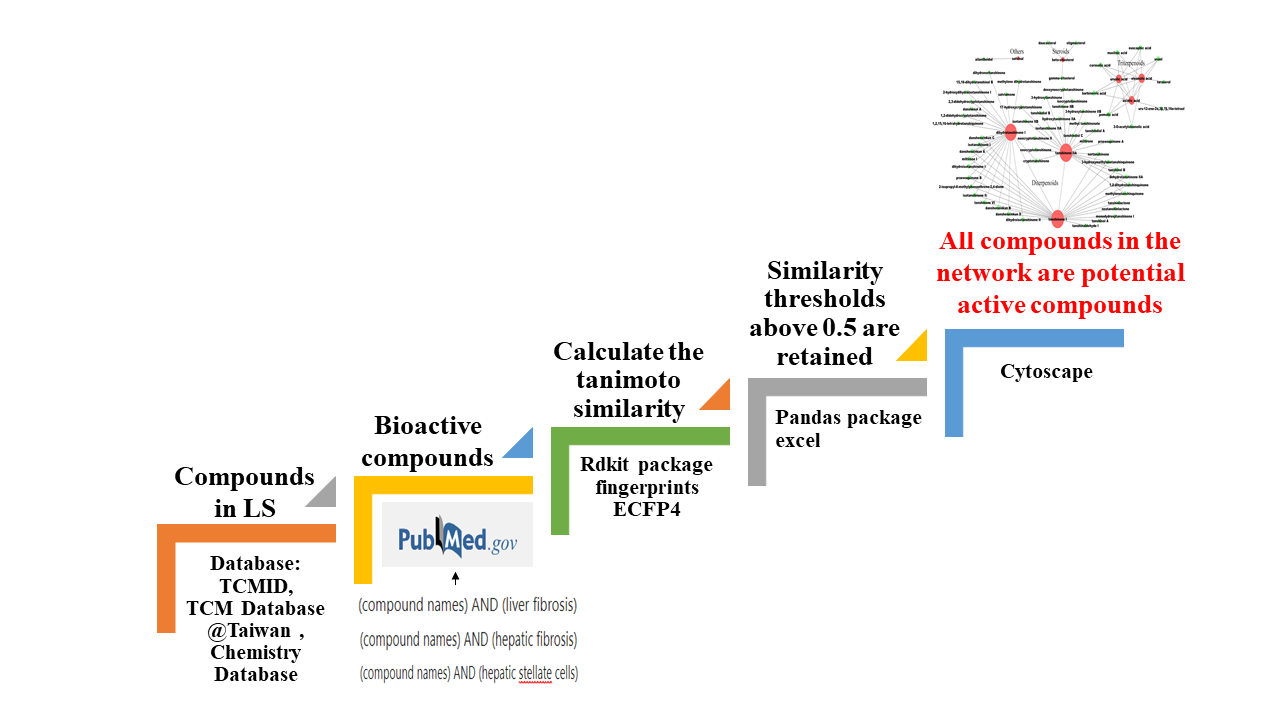


**Supplementary Figure 2.** The safety assessment of LS (30 g/kg/day)
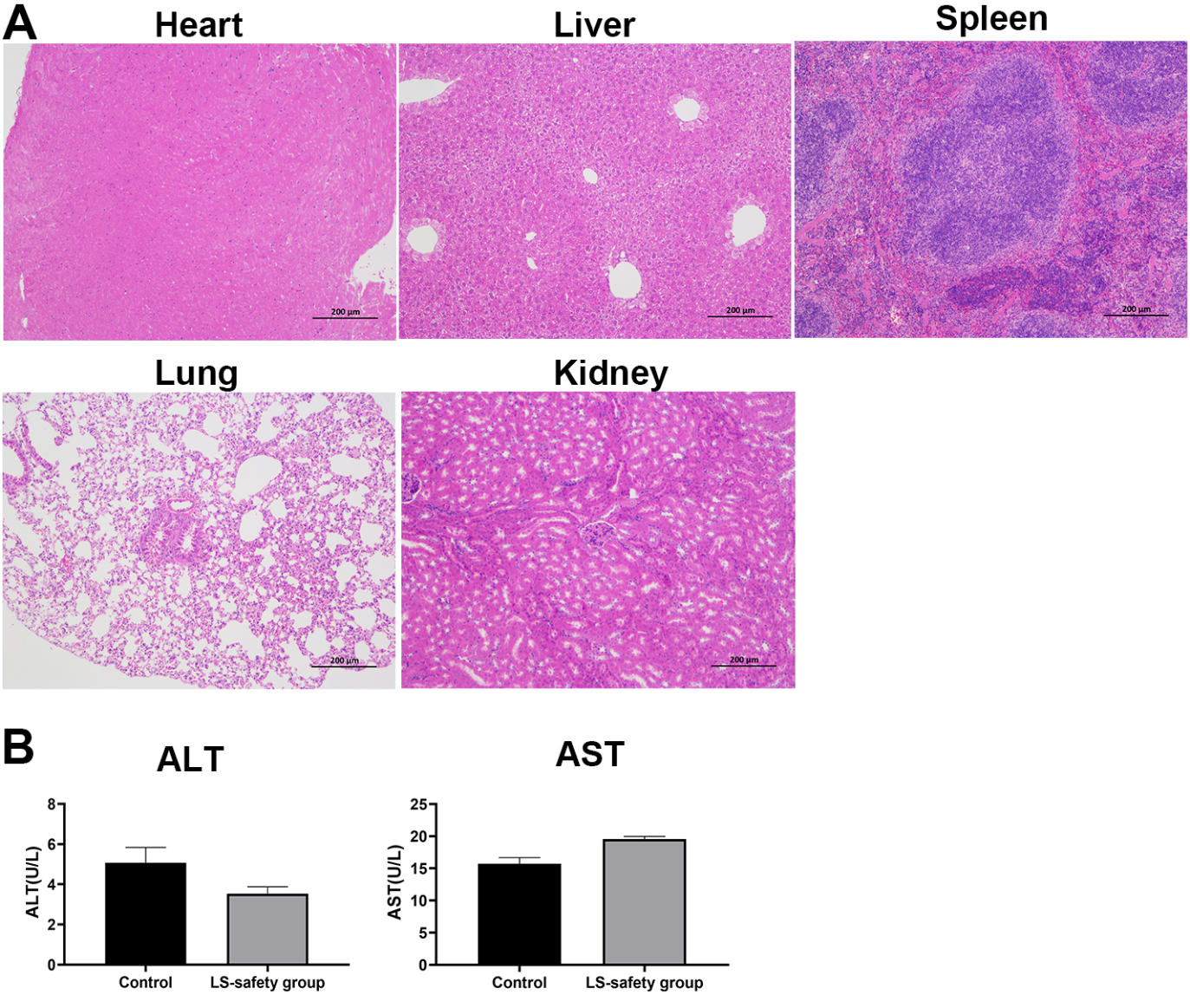

Supplement: Supplementary file 1 [file DataSheet1.docx]
